# Supplementary material for: Age-dependent seroprevalence of dengue and chikungunya: inference from a cross-sectional analysis in Esmeraldas Province in coastal Ecuador
Source: BMJ Open. 2020 Oct 16;10(10):e040735. doi: 10.1136/bmjopen-2020-040735 (PMC7569951; doi:10.1136/bmjopen-2020-040735)
Supplement: Supplementary data [file bmjopen-2020-040735supp001.pdf]

Supplementary Tables and figure captions

Supplementary Table 1. Comparison between serological sample and cohort with respect to area of residence and socioeconomic status of households.

Supplementary Table 2. Geographical distribution of households by parish across region of Esmeraldas (census), cohort and data sample. The data sample distribution is similar to that of the cohort and the post-stratification weights adjusted for a fair representation of the Esmeraldas Province. The weights represent essentially the ratios between the census proportions and data proportions across each region. This calibration also adjusted for urban/rural area of residence.

Supplementary Table 3. Total number of reported cases of dengue and chikungunya in Ecuador and Esmeraldas province in 2015 compared to the 2014 and 2016. Overall proportions of cases were calculated using Ecuador and Esmeraldas population total as denominator, respectively. Ecuador census data for 2010 were used and the totals were adjusted according to the annual predicted population growth.

Supplementary Table 4. Age distributions of Esmeraldas census population and serological sample from cohort. The weights used in modelling were the ratios between Esmeraldas census population distribution proportions and age-group proportions in the serological sample.

Supplementary Table 5 Average predicted seroprevalence (proportions) for each exposure category and their 95%Credible Intervals (CrI) for dengue and chikungunya and seroprevalence difference by sex (female [F] vs. males [M]), area of residence (urban [U] vs. rural [R]), and socioeconomic level (low [L] vs. high [H]). Exposure categories are: DENV+CHIKV- [D+C-]; DENV-CHIKV+ [D-C+]; and DENV+CHIKV+ [D+C+].

Supplementary Figure 1. Reported clinical cases of dengue and chikungunya in 2015 in Ecuador nationally and in the Province of Esmeraldas. Time-series graphs were derived from data publicly accessible (17, 18, 19, 20)

Supplementary Figure 2. Predicted age-dependent seroprevalence for exposure groups to dengue and chikungunya for categories of area of residence, sex, and household dispersal in study sample. Exposure categories are: DENV+CHIKV- [D+C-]; DENV-CHIKV+ [D-C+]; and DENV+CHIKV+ [D+C+]. Dashed lines represent the 95% Credible Intervals.

Supplementary Figure 3. Predicted age-dependent differences for exposure categories by sex (A), area of residence (B), and socioeconomic status (C). Exposure categories were DENV+CHIKV- [D+C-]; DENV-CHIKV+ [D-C+]; and DENV+CHIKV+ [D+C+]. Dashed lines represent 95% Credible Intervals.

Supplementary Figure 4 Age-dependent seroprevalence for exposure groups to dengue and chikungunya stratified by sex, socioeconomic status, and area of residence. Exposure categories were DENV+CHIKV- [D+C-]; DENV-CHIKV+ [D-C+]; and DENV+CHIKV+ [D+C+]. Dashed lines represent the 95% Credible Intervals of the predictions derived from the multivariable model

Supplementary Figure 5. Age-dependent seroprevalence for exposure groups to dengue and chikungunya stratified by area of residence, sex, and socioeconomic status. Exposure categories were DENV+CHIKV- [D+C-]; DENV-CHIKV+ [D-C+]; and DENV+CHIKV+ [D+C+]. Dashed lines represent the 95% Credible Intervals of the predictions derived from the multivariable model (Table 2).

Supplementary Figure 6. Age-dependent seroprevalence for exposure groups to dengue and chikungunya stratified by socioeconomic status, sex, and area of residence. Exposure categories were DENV+CHIKV- [D+C-]; DENV-CHIKV+ [D-C+]; and DENV+CHIKV+ [D+C+]. Dashed lines represent the 95% Credible Intervals of the predictions derived from the multivariable model (Table 2).

Supplementary Figure 7. Model goodness of fit: the observed seropositive numbers in each 5-year age group vs. the expected numbers calculated using average age-specific MCMC predictions and the total number in the corresponding age group: (a) for D+C-, (b) for D-C+ and (c) for D+C+.

Supplementary Figure 8 Model goodness of fit: the observed seropositive numbers in groups defined by geographical regions vs. the corresponding expected numbers calculated using MCMC predicted prevalence in that group and its total numbers: (a) for all DENV and (b) for all CHIKV.

Supplementary Figure 9. Parameter estimates for the most complex models - the MCMC traces for 100000 iterations.

Supplementary Figure 10. Parameter correlations based on the MCMC traces for 100000 iterations.

Supplementary Figure 11 Variance-covariance components (random effects) estimation associated with hierarchical structure of the data (family and ward).
