# Supplementary material for: Age-dependent seroprevalence of dengue and chikungunya: inference from a cross-sectional analysis in Esmeraldas Province in coastal Ecuador
Source: BMJ Open. 2020 Oct 16;10(10):e040735. doi: 10.1136/bmjopen-2020-040735 (PMC7569951; doi:10.1136/bmjopen-2020-040735)
Supplement: Supplementary data [file bmjopen-2020-040735supp002.pdf]

		Serological cohort 151 households	Ecuavida 2404 households	Serological cohort vs Ecuavida
**Variable**	**Category**	**Number/Prop**	**Number/Prop**	**One-sample test**
Administrative location	Urban	115 (76.2%)	1,796 (75%)	p=0.74
	Rural	36 (23.8%)	608 (25%)	
Socio Economic Status	High	45 (29.8%)	797 (33.2%)	P=0.46
	Low	106 (70.2%)	1,607(66.8%)	

Supplementary Table 1
