# Supplementary material for: Age-dependent seroprevalence of dengue and chikungunya: inference from a cross-sectional analysis in Esmeraldas Province in coastal Ecuador
Source: BMJ Open. 2020 Oct 16;10(10):e040735. doi: 10.1136/bmjopen-2020-040735 (PMC7569951; doi:10.1136/bmjopen-2020-040735)
Supplement: Supplementary data [file bmjopen-2020-040735supp003.pdf]

	ALL	ECUAVIDA	SAMPLE	Prop of ALL	Prop of ECUAVIDA	Prop of SAMPLE	PROP ECUAVIDA / PROP ALL	PROP SAMPLE / PROP ECUAVIDA	RATIO OF THE LAST	WEIGHTS= 1/RATIO
**Rosa Zarate**	16342	1578	102	0.353	0.656	0.675	1.862	1.029	1.916	**0.522**
**La Concord**	10662	217	13	0.230	0.090	0.086	0.392	0.954	0.374	**2.672**
**PuertoQuito**	5037	27	3	0.109	0.011	0.020	0.103	1.769	0.183	**5.469**
**La Unión**	4963	351	21	0.107	0.146	0.139	1.364	0.953	1.299	**0.770**
**Cube+Others**	4163	49	6	0.090	0.020	0.040	0.227	1.949	0.442	**2.260**
**Malimpia**	4004	81	3	0.086	0.034	0.020	0.390	0.590	0.230	**4.348**
**Viche**	1185	101	3	0.026	0.042	0.020	1.644	0.473	0.777	**1.287**
**Total**	46356	2404	151	1	1	1				

Supplementary Table 2
