# Supplementary material for: Age-dependent seroprevalence of dengue and chikungunya: inference from a cross-sectional analysis in Esmeraldas Province in coastal Ecuador
Source: BMJ Open. 2020 Oct 16;10(10):e040735. doi: 10.1136/bmjopen-2020-040735 (PMC7569951; doi:10.1136/bmjopen-2020-040735)
Supplement: Supplementary data [file bmjopen-2020-040735supp004.pdf]

Dengue and Chikungunya in Ecuador – Supplementary Statistical Inference

Supplementary: The statistical model and inference

The model

This is a model borrowed from econometrics (1) and recently used in the context of infectious diseases which occur simultaneously or are inherently influenced by correlated binary outcomes. We adapted the methodology in the framework of these two binary highly correlated outcomes which can be associated with a similar (or not) set of covariates and the presence of one is highly correlated with the other. A lay description of the approach is presented in the main text. Here, we give details on the mathematical equations and the statistical inference.

Let us denote by X and Y the two binary random variables indicating the presence/absence of an individual to DENV and CHIKV, respectively. This pair of responses define a binary bivariate outcome denoted by (X, Y).

Let us also denote

$R_i = 1 \times \boldsymbol{K}_X$ vector of variables associated with X

$\boldsymbol{\beta} = \boldsymbol{K}_X \times 1$ vector of unknown parameters which quantify the effect of variables associated with X

$Q_i = 1 \times \boldsymbol{K}_Y$ vector of variables associated with Y

$\boldsymbol{\gamma} = \boldsymbol{K}_Y \times 1$ vector of unknown parameters which quantify the effect of variables associated with Y,

$i = \overline{1,319}$, $j = \overline{1,151}$, $k = \overline{1{,}49}$

NB: We deliberately keep the generality of the model specification although in our scenario $\boldsymbol{K}_X = \boldsymbol{K}_Y$

Chis Ster I, *et al. BMJ Open* 2020; 10:e040735. doi: 10.1136/bmjopen-2020-040735

Then, the two simultaneous logistic equations which depict the nonlinear association with age and the rest of the other variables are written in terms of a system of conditional probabilities on the potential explanatory variables and their random effects:

$$\begin{cases} log\left(\frac{P(X=1|Y)}{P(X=0|Y)}\right)_{ijk} = \beta_{age} \times \log(\text{age}_{ijk}) + \boldsymbol{\beta} \times R_{ijk} + \boldsymbol{\alpha} \times Y_{ijk} + w_{ijk}^{age} + w_k^{ward} + v_{0k}^1 + u_{0jk}^1 \\ log\left(\frac{P(Y=1|X)}{P(Y=0|X)}\right)_{ijk} = \gamma_{age} \times \log(\text{age}_{ijk}) + \boldsymbol{\gamma} \times Q_{ijk} + \boldsymbol{\theta} \times X_{ijk} + w_{ijk}^{age} + w_k^{ward} + v_{0k}^2 + u_{0jk}^2 \end{cases}$$

(1)

The triple index (ijk) – denotes individual i in the family j and the ward k

w_{ijk}^{age} denote the age-group constants associated weights (see the subsequent section on Population adjusting weights)

w_k^{ward} denote the ward constants associated weights (see the subsequent section on Population adjusting weights)

$v_{0k}^{1,2}$ denote the random effect associated with the ward

$u_{0jk}^{1,2}$ denote the random effect associated with the family

The latter two sets of random effects are assumed to be drawn from a two normal bivariate normal distributions (MVN) which model the shared and unshared similarities and account for unmeasured aspects such as mosquitos' burden around the households or weather patterns for instance.

$$\begin{pmatrix} v_{0k}^1 \\ v_{0k}^2 \end{pmatrix} \sim MVN\left(\begin{pmatrix} 0 \\ 0 \end{pmatrix}, \sum\begin{pmatrix} (v_0^1)^2 & \rho_v v_0^1 v_0^2 \\ \rho_v v_0^1 v_0^2 & (v_0^2)^2 \end{pmatrix}\right)$$

Dengue and Chikungunya in Ecuador – Supplementary Statistical Inference

$$\begin{pmatrix} u_{0jk}^{1} \\ u_{0jk}^{2} \end{pmatrix} \sim MVN\left(\begin{pmatrix} 0 \\ 0 \end{pmatrix}, \sum \begin{pmatrix} (u_0^1)^2 & \rho_u u_0^1 u_0^2 \\ \rho_u u_0^1 u_0^2 & (u_0^2)^2 \end{pmatrix}\right)$$

On one hand side, from the first simultaneous equation specification (1):

$$\boldsymbol{\alpha} = log\left[\frac{P(X=1|Y=1)}{P(X=0|Y=1)}\right] - log\left[\frac{P(X=1|Y=0)}{P(X=0|Y=0)}\right] = log\frac{P(X=1,Y=1)/P(Y=1)}{P(X=0,Y=1)/P(Y=1)} - log\frac{P(X=1,Y=0)/P(Y=0)}{P(X=0,Y=0)/P(Y=0)} =$$

$$log\left[\frac{P(X=1,Y=1)\times P(X=0,Y=0)}{P(X=0,Y=1)\times P(X=1,Y=0)}\right] \quad (2)$$

From the second simultaneous equation specification:

$$\boldsymbol{\theta} = log\left[\frac{P(Y=1|X=1)}{P(Y=0|X=1)}\right] - log\left[\frac{P(Y=1|X=0)}{P(Y=0|X=0)}\right] = log\frac{P(X=1,Y=1)/P(X=1)}{P(X=0,Y=1)/P(X=1)} - log\frac{P(X=1,Y=0)/P(X=0)}{P(X=0,Y=0)/P(X=0)} =$$

$$log\left[\frac{P(X=1,Y=1)\times P(X=0,Y=0)}{P(X=0,Y=1)\times P(X=1,Y=0)}\right] \quad (3)$$

Hence, using the key assumption that $\boldsymbol{\alpha} = \boldsymbol{\theta}$ and keeping the fixed effects only for the sake of simplicity, the system can be written as

$$\begin{cases} log\left(\dfrac{P(X=1|Y)}{P(X=0|Y)}\right)_i = \boldsymbol{\beta} \times R_i + \boldsymbol{\alpha} \times Y_i \\ log\left(\dfrac{P(Y=1|X)}{P(Y=0|X)}\right)_i = \boldsymbol{\gamma} \times Q_i + \boldsymbol{\alpha} \times X_i \end{cases}$$

This implies that, if the occurrence of dengue makes more likely (or less likely) the occurrence of chikungunya, the converse is also necessarily true and $\boldsymbol{\alpha}$ is deemed as a nuisance parameter

This crucial assumption $(\boldsymbol{\alpha} = \theta)$ and elementary rules of conditioned probabilities leads to the following four statements derived from

Chis Ster I, *et al. BMJ Open* 2020; 10:e040735. doi: 10.1136/bmjopen-2020-040735

Dengue and Chikungunya in Ecuador – Supplementary Statistical Inference

$$\begin{cases} \frac{P(X=1|Y=0)}{P(X=0|Y=0)} = \frac{P(X=1,Y=0)}{P(X=0,Y=0)} = exp(\boldsymbol{\beta} \times \boldsymbol{R}) & (4) \\ \frac{P(X=1|Y=1)}{P(X=0|Y=1)} = \frac{P(X=1,Y=1)}{P(X=0,Y=1)} = exp(\boldsymbol{\beta} \times \boldsymbol{R} + \alpha) & (5) \end{cases}$$

$$\begin{cases} \frac{P(Y=1|X=0)}{P(Y=0|X=0)} = \frac{P(Y=1,X=0)}{P(Y=0,X=0)} = exp(\boldsymbol{\gamma} \times \boldsymbol{Q}) & (6) \\ \frac{P(Y=1|X=1)}{P(Y=0|X=1)} = \frac{P(Y=1,X=1)}{P(Y=0,X=1)} = exp(\boldsymbol{\gamma} \times \boldsymbol{Q} + \alpha) & (7) \end{cases}$$

Hence, combining either (4) + (7) (or (5) + (6)) by multiplication

$$\frac{P(X=1,Y=0)}{P(X=0,Y=0)} \times \frac{P(Y=1,X=1)}{P(Y=0,X=1)} =$$

$$\frac{P(Y=1,X=1)}{P(Y=0,X=0)} = exp(\boldsymbol{\beta} \times \boldsymbol{R}) \times \; exp(\boldsymbol{\gamma} \times \boldsymbol{Q} + \alpha) = exp(\boldsymbol{\beta} \times \boldsymbol{R} + \boldsymbol{\gamma} \times \boldsymbol{Q} + \alpha)$$

But all four probabilities situations ((1, 0), (0, 1), (1, 1), (0,0)) sum to 1:

$$P(X=1,Y=0) + P(X=0,Y=1) + P(Y=0,X=0) + P(Y=1,X=1) = 1$$

$$P(X=0,Y=0) + P(X=0,Y=0) \times exp(\boldsymbol{\gamma} \times Q) + P(Y=0,X=0) \times exp(\boldsymbol{\beta} \times \boldsymbol{R})$$
$$+ P(Y=0,X=0) \times exp(\boldsymbol{\beta} \times \boldsymbol{R} + \boldsymbol{\gamma} \times \boldsymbol{Q} + \alpha) = 1$$

So

$$P(X=0,Y=0) \times \left(1 + exp(\boldsymbol{\gamma} \times \boldsymbol{Q}) + exp(\boldsymbol{\beta} \times \boldsymbol{R}) + exp(\boldsymbol{\beta} \times \boldsymbol{R} + \boldsymbol{\gamma} \times \boldsymbol{Q} + \alpha)\right)=1$$

Therefore, the predicted joint probabilities can be derived as it follows:

$$P(X=0,Y=0) = \frac{1}{1 + exp(\boldsymbol{\gamma} \times \boldsymbol{Q}) + exp(\boldsymbol{\beta} \times \boldsymbol{R}) + exp(\boldsymbol{\beta} \times \boldsymbol{R} + \boldsymbol{\gamma} \times \boldsymbol{Q} + \alpha)}$$

$$P(X=0,Y=1) = \frac{exp(\boldsymbol{\gamma} \times \boldsymbol{Q})}{1 + exp(\boldsymbol{\gamma} \times \boldsymbol{Q}) + exp(\boldsymbol{\beta} \times \boldsymbol{R}) + exp(\boldsymbol{\beta} \times \boldsymbol{R} + \boldsymbol{\gamma} \times \boldsymbol{Q} + \alpha)}$$

$$P(X=1,Y=0)=\frac{exp(\boldsymbol{\beta}\times\boldsymbol{R})}{1+exp(\boldsymbol{\gamma}\times\boldsymbol{Q})+exp(\boldsymbol{\beta}\times\boldsymbol{R})+exp(\boldsymbol{\beta}\times\boldsymbol{R}+\boldsymbol{\gamma}\times\boldsymbol{Q}+\alpha)}$$

$$P(X=1,Y=1)=\frac{exp(\boldsymbol{\beta}\times\boldsymbol{R}+\boldsymbol{\gamma}\times\boldsymbol{Q}+\alpha)}{1+exp(\boldsymbol{\gamma}\times\boldsymbol{Q})+exp(\boldsymbol{\beta}\times\boldsymbol{R})+exp(\boldsymbol{\beta}\times\boldsymbol{R}+\boldsymbol{\gamma}\times\boldsymbol{Q}+\alpha)}$$

Denoting by

$$I_{ij}=\{k|X_k=i,Y_k=j\}\ where\ \ i,j=0,1$$

If the vector of the unknown parameters are denoted by $\phi=(\alpha,\boldsymbol{\beta},\boldsymbol{\gamma})$**,** the model likelihood can be formally written as

$$L(\phi|Y_k,X_k;\boldsymbol{R},\boldsymbol{Q})=\prod_{i=0,1}\prod_{j=0,1}\prod_{k\in I_{ij}}P(X_k=i,Y_k=j)$$

Bayesian inference consist of summarizing the posterior distributions of the model parameters and requires the joint probability distribution over all random quantities, parameters or observables. The marginal and conditional probability distributions of the vector of unknown parameters are denoted by $Pr(\cdot)$ and $Pr(\cdot\mid\cdot)$. The ϕ posterior distribution is proportional to the likelihood multiplied by their prior distributions – all assumed non informative as stated in the text. That is

$$Pr(\phi|Y_k,X_k;\boldsymbol{R},\boldsymbol{Q})\propto L(\phi|Y_k,X_k;\boldsymbol{R},\boldsymbol{Q})\times Pr(\phi)$$

Statistical Inference

The simultaneous equations systems (1) define a probability model which has been implemented in OpenBUGS - which makes this approach very appealing even for larger datasets (OpenBUGS program for the simplest model appended). A Bayesian framework for statistical inference using Markov chain Monte Carlo (MCMC) estimation methods naturally accommodates missing

responses (5%) in the data and allows straightforward assessment of uncertainties in nonlinear models under missing at random assumption. Sensitivities analyses to the missing data (by assuming all positives and all negatives) have been conducted but the results were not altered.

The posterior conditional odds ratios (OR) distributions are presented in Table 2. However, for an easily digestible reading, the summary statistics are presented in terms of means and standard deviations of the predicted joint probabilities by age and stratified by different socio-economic factors that we accounted for.

The MCMC estimation followed 1 million iterations and the corresponding chains (log scale) are displayed in Supplementary Figure 8 – including that of the nuisance parameter. Supplementary Figure 9 display the correlation between the parameters which are all lower than 0.5 in absolute values with the exception of that between the constant and age corresponding to the dengue equations with appear to be highly negatively correlated (-0.87).

Non-informative priors have been used for parameter estimation.

Population adjusting weights used in modelling

We used the 2010 Census data (the latest) adjusted for an average annual growth of in the Ecuador population. All statistical analyses should ultimately serve population predictions. The post-stratification weights, namely calibration, is a technique which corrects the estimation for population misrepresentation which often occurs in ad-hoc surveys or when researchers use already recruited participants. One set of weights are aimed at reducing the effect of Rosa Zarate where most of the data were collected and at inflating the influence of the misrepresented groups (La Union and 10-19 years) on the model likelihood. These weights are associated with each individual and are additive for the log scale model formulation of the model (see equations

above). The other set of weights are aimed at reducing the effect of those in young group and inflate the effects of older people less represented in the sample. The weights are displayed Supplementary Tables 3 and 4. Each observation is associated with a weight according to individual's age group and one by the household location and their additive effect is accounted for in the predictors (log scale so their effect on the actual odds ratios or odds would be multiplicative).

Goodness of fit

The model is developed using a fully parametric paradigm and inherits complexities including random effects for binary outcomes which are not associated with any classical or formal goodness of fit measures.

However, in reference to the basic independent binary outcomes, the only goodness-of-fit measure following a potential *individual based model* fit to these data is that of Hosmer-Lemeshow statistic. That invokes partition of the data *into groups* (https://thestatsgeek.com/2014/02/16/the-hosmer-lemeshow-goodness-of-fit-test-for-logistic-regression/). That is because the individual level raw observations are either 0 or 1 whilst the individual level predicted values can be anywhere between 0 and 1 and hence a one-to-one observed-predicted plot often used for continuous data loses its meaning.

Hence, our approach alludes to that of basic logistic regression and follows similar lines to those developed in Chis Ster, I. Journal of Applied Statistics, 2012. **39**(11): p. 2523-2542, (*Inference for serological surveys investigating past exposures to infections resulting in long-lasting immunity – an approach using finite mixture models with concomitant information)* within a

related and yet equally complex modelling framework.

We split the data into groups, by 5 years age-groups and geographical sub-regions, and calculated the average predicted probabilities and their 95%CrIs for the outcomes (((D+, C-), (D-, C+), (D+, C+) and also for all dengue and chikungunya) within each group. Then we calculated the expected number of positives corresponding each group and each outcome component by multiplying the corresponding average predicted probabilities with the total number of people within that group. We plotted the observed numbers vs. the predicted numbers in each group.

Given the full parametric, parsimonious nature of this model, and the average sized data – we deemed our model as a good fit to these data.

OpenBUGS code:

```
model{
    #model's likelihood
       for (i in 1:319){
              log_age[i]<-log(age_final_years[i]);
              log_dist[i]<-log(1+min_dist[i]/250);
              weights_final[i]<-weights[i]+weight_age[i];

              dengue[i]~dbern(p[i,1]);
              chick[i] ~dbern(p[i,2]);
              eta[i,1]<-beta1[1]+beta1[2]*log_age[i]+alpha*chick[i]+beta1[3]*gender[i]+
                                beta1[4]*ses_bin[i]+
                                weights_final[i]+
                                theta[family[i],1]+
                                ward_level[ward_index[i],1];

             eta[i,2]<- beta2[1]+beta2[2]*log_age[i]+alpha*dengue[i]+
                                beta2[3]*gender[i]+
                                beta2[4]*urbanrural[i]+beta2[5]*log_dist[i]+
                                weights_final[i]+
                                theta[family[i],2]+
                                ward_level[ward_index[i],2];
               logit(p[i,1])<-eta[i,1];
               logit(p[i,2])<-eta[i,2];
       }
       for (j in 1:151){
              theta[j,1:2] ~ dmnorm(mu.fam[],R_fam[,]);
        }
    for(j in 1:49){
         ward_level[j,1:2] ~ dmnorm(mu.ward[],R_ward[,]);
    }
    for (k in 1:2){
                    mu.fam[k] <-0.0;
                  mu.ward[k] <-0.0;
       }

    R_fam[1:2,1:2] ~dwish(Omega_fam[,],2);
    R_ward[1:2,1:2]~dwish(Omega_ward[,],2);
    Omega_fam[1,1]<-1; Omega_fam[1,2]<-0; Omega_fam[2,1]<-0; Omega_fam[2,2]<-1;
 Omega_ward[1,1]<-1; Omega_ward[1,2]<-0; Omega_ward[2,1]<-0; Omega_ward[2,2]<-1;
```


Chis Ster I, *et al. BMJ Open* 2020; 10:e040735. doi: 10.1136/bmjopen-2020-040735

Dengue and Chikungunya in Ecuador – Supplementary Statistical Inference

```
    Sigma.fam[1:2, 1:2] <-inverse(R_fam[,]);
    Sigma.ward[1:2, 1:2]<-inverse(R_ward[,]);
    for (j in 1:2){
        sigma.fam[j]<-sqrt(Sigma.fam[j,j]);
        sigma.ward[j]<-sqrt(Sigma.ward[j,j]);
        for(k in 1:j){
                rho_fam[j,k]<-0;
                 rho_ward[j,k]<-0;
         }
        for(k in (j+1):2){
               rho_fam[j,k]<-Sigma.fam[j,k]/(sigma.fam[j]*sigma.fam[k]);
               rho_ward[j,k]<-Sigma.ward[j,k]/(sigma.ward[j]*sigma.ward[k]);
        }
    }
    alpha ~ dnorm( 0.0, 0.00001);
    for (j in 1:4){
        beta1[j] ~ dnorm( 0.0, 0.00001);
        beta2[j] ~ dnorm( 0.0, 0.00001);
        exp_beta1[j]<-exp(beta1[j]);
         exp_beta2[j]<-exp(beta2[j]);
         eff1[j] <-step(beta1[j]);
         eff2[j] <-step(beta2[j]);
    }
           exp_alpha<-exp(alpha);
           beta2[5] ~ dnorm( 0.0, 0.00001);
           exp_beta2[5]<-exp(beta2[5]);
}
```

References

1. Schmidt P, Strauss RP. Estimation of Models with Jointly Dependent Qualitative Variables: A Simultaneous Logit Approach. Econometrica. 1975;43(4):745-55.
2. Ghebremichael M. Joint modeling of correlated binary outcomes: HIV-1 and HSV-2 co-infection. Journal of Applied Statistics. 2015 2015/10/03;42(10):2180-91.
3. Goldstein H. Multilevel Statistical Models. 4th Edition ed: JohnWiley and Sons, Ltd, Chichester; 2011.

Chis Ster I, *et al. BMJ Open* 2020; 10:e040735. doi: 10.1136/bmjopen-2020-040735
