# Supplementary material for: Age-dependent seroprevalence of dengue and chikungunya: inference from a cross-sectional analysis in Esmeraldas Province in coastal Ecuador
Source: BMJ Open. 2020 Oct 16;10(10):e040735. doi: 10.1136/bmjopen-2020-040735 (PMC7569951; doi:10.1136/bmjopen-2020-040735)
Supplement: Supplementary data [file bmjopen-2020-040735supp005.pdf]

Year	**Population**		**Dengue**		**Chickungunya**		**Zika**	
	**Ecuador**	**Esmeraldas**	**Ecuador**	**Esmeraldas**	**Ecuador**	**Esmeraldas**	**Ecuador**	**Esmeraldas**
2014	15,944,275	187,880	15, 584 (0.10%)	984 (0.5%)	39 (~0%)	0 (0%)	0 (0%)	0 (0%)
2015	16,144,368	196,350	42, 499 (0.26%)	3,482 (1.8%)	34,101 (0.2%)	10,477 (5.3%)	1 (0%)	0 (0%)
2016	16,385,068	204,820	13,612 (0.08%)	1,311 (0.6%)	1, 860 (0.01%)	301 (0.15%)	2946(0.02%)	192(0.09%)

Supplementary Table 3
