# Supplementary figures and images for: Age-dependent seroprevalence of dengue and chikungunya: inference from a cross-sectional analysis in Esmeraldas Province in coastal Ecuador
Source: BMJ Open. 2020 Oct 16;10(10):e040735. doi: 10.1136/bmjopen-2020-040735 (PMC7569951; doi:10.1136/bmjopen-2020-040735)

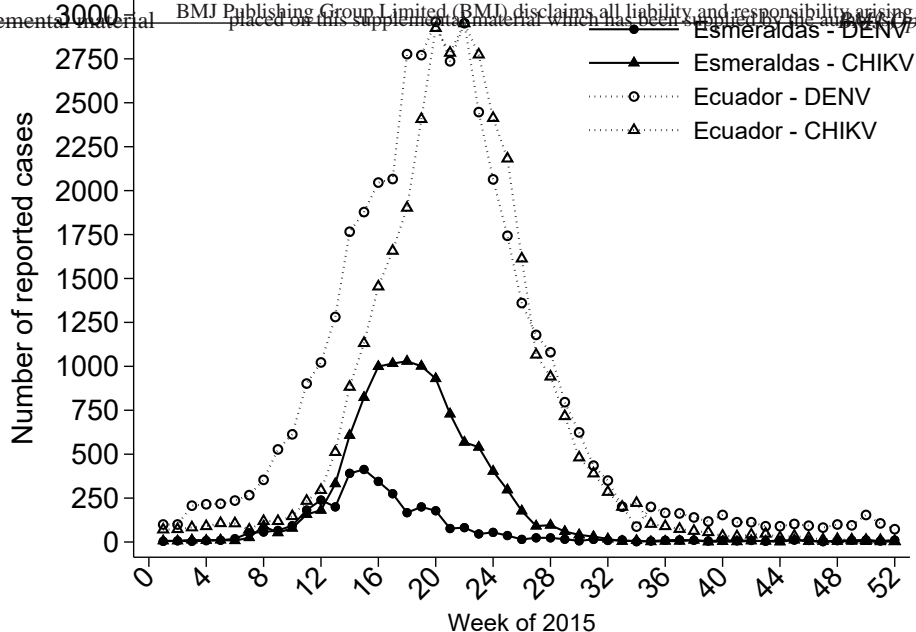

Supplement: Supplementary data [file bmjopen-2020-040735supp006.pdf]

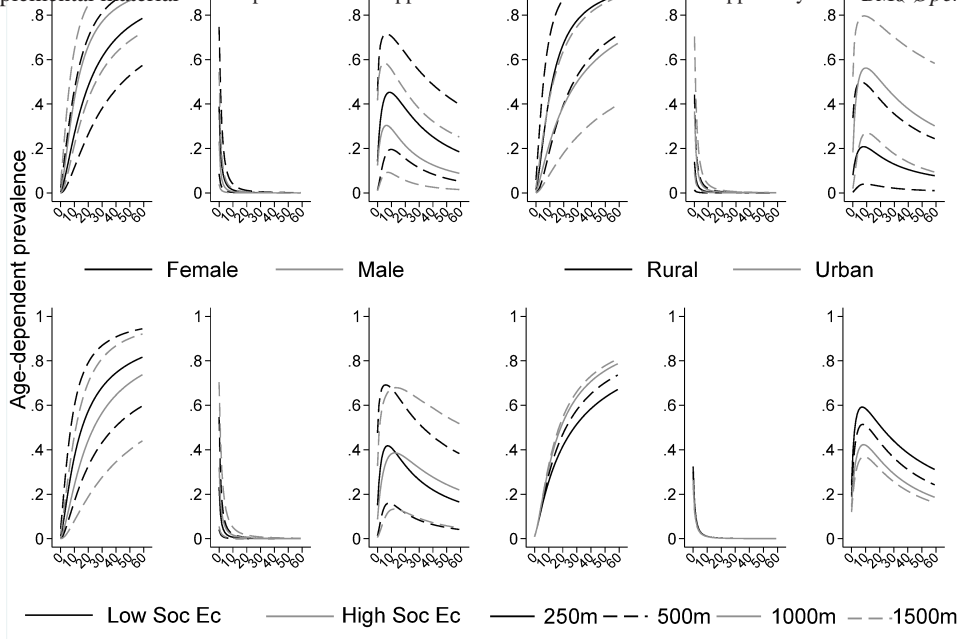

Supplement: Supplementary data [file bmjopen-2020-040735supp009.pdf]

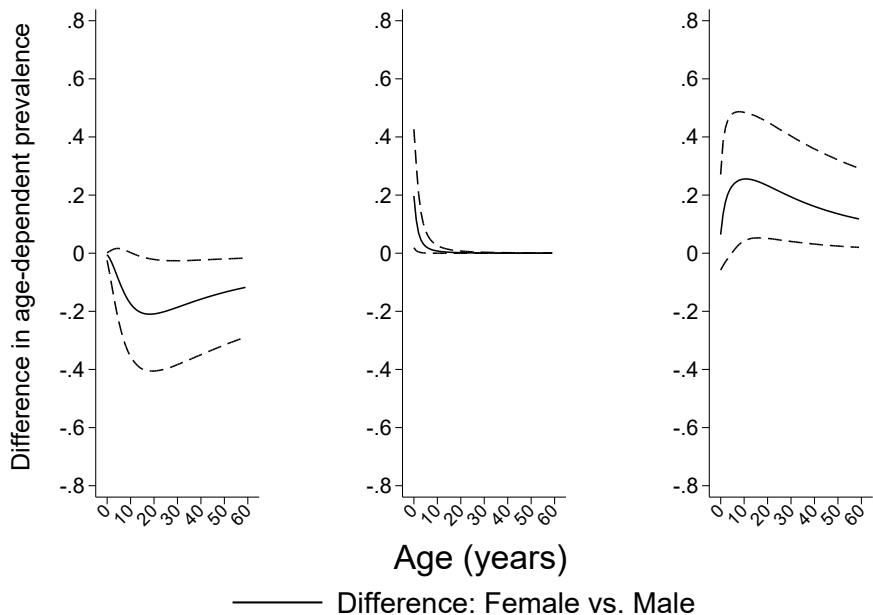

Supplement: Supplementary data [file bmjopen-2020-040735supp010.pdf]

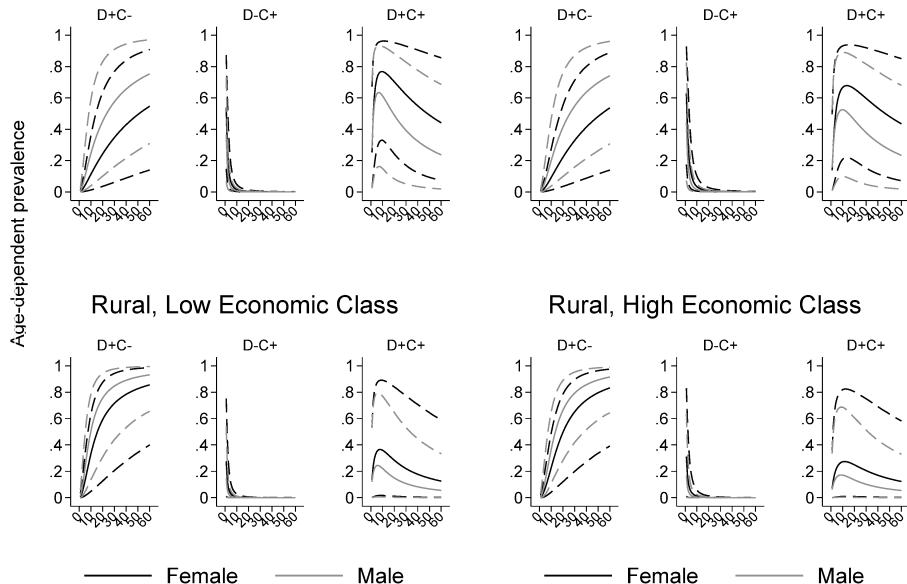

Supplement: Supplementary data [file bmjopen-2020-040735supp011.pdf]

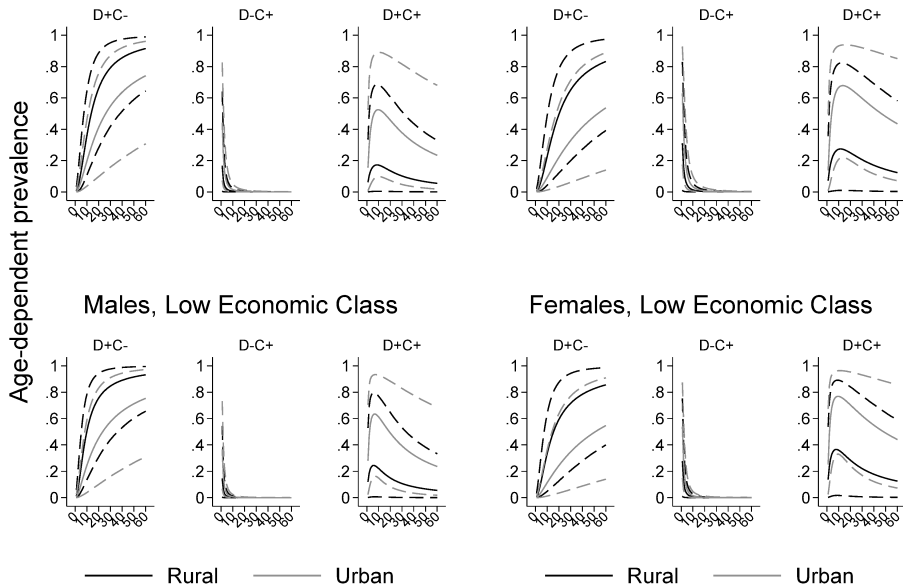

Supplement: Supplementary data [file bmjopen-2020-040735supp012.pdf]

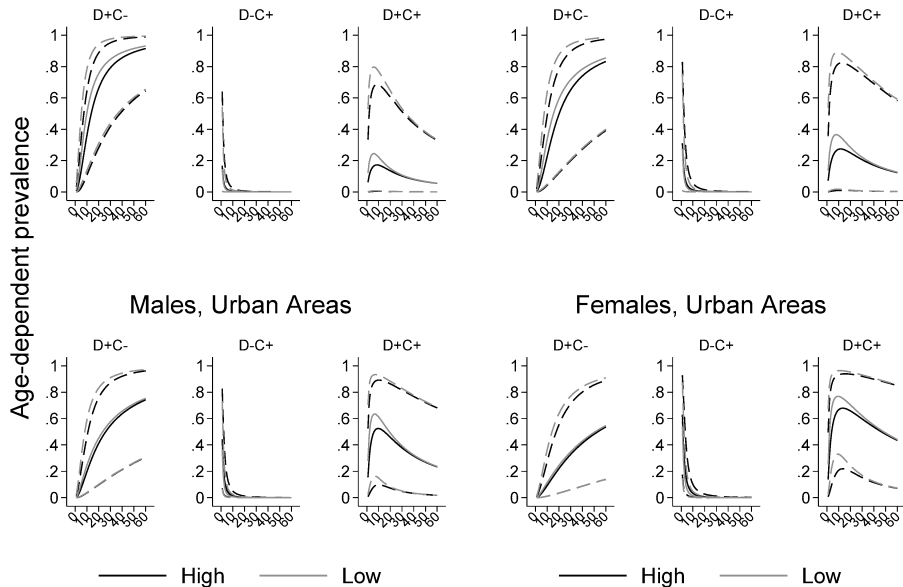

Supplement: Supplementary data [file bmjopen-2020-040735supp013.pdf]

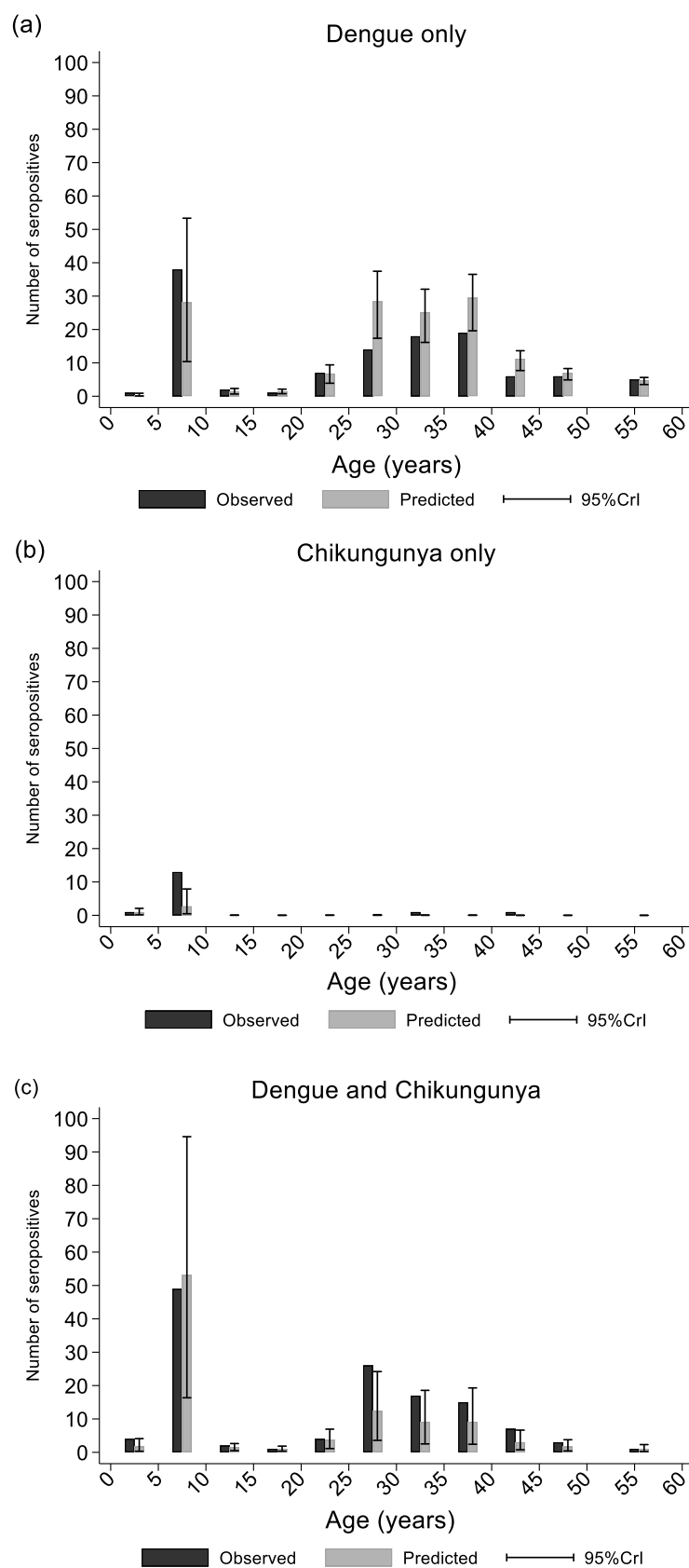

Supplement: Supplementary data [file bmjopen-2020-040735supp014.pdf]

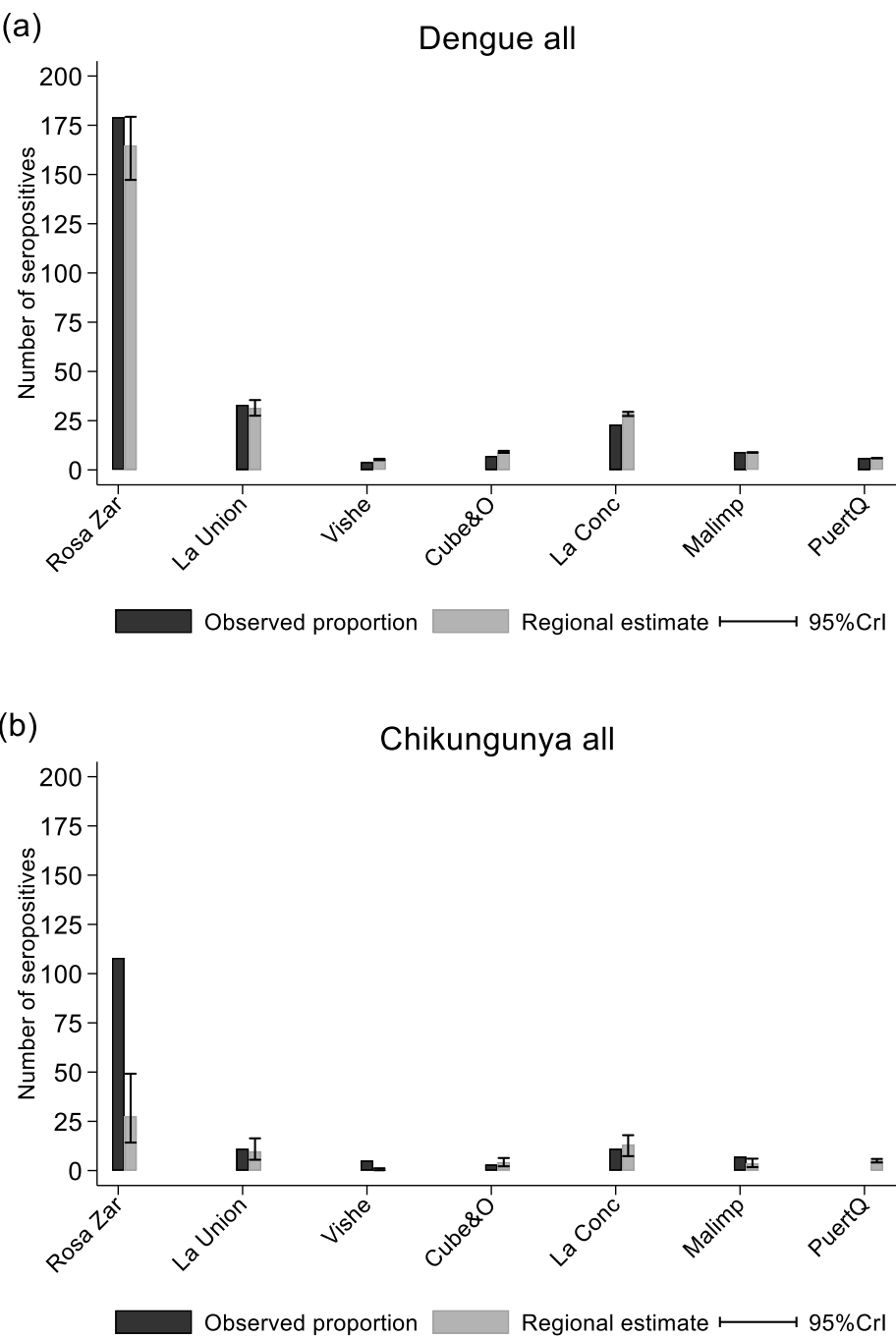

Supplement: Supplementary data [file bmjopen-2020-040735supp015.pdf]

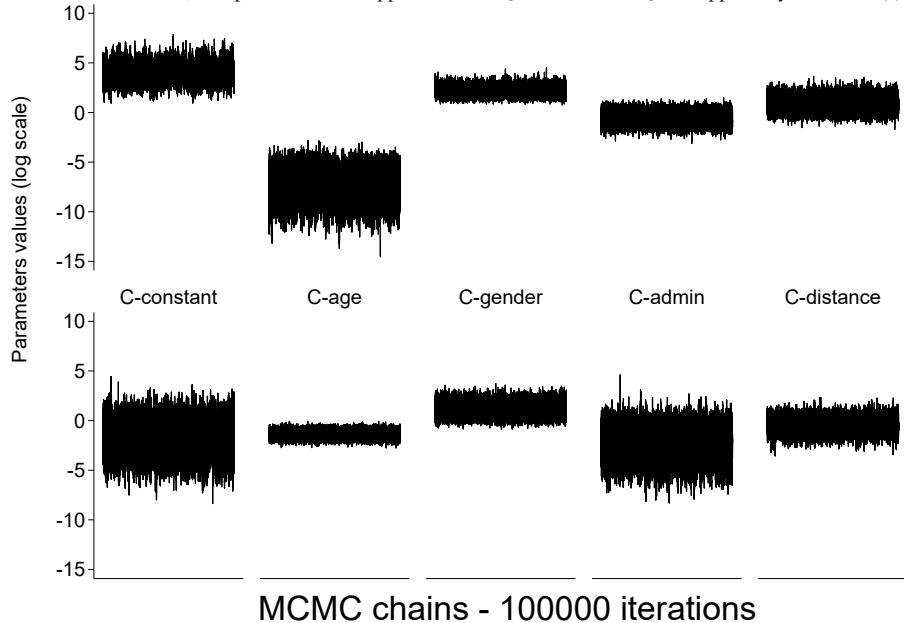

Supplement: Supplementary data [file bmjopen-2020-040735supp016.pdf]

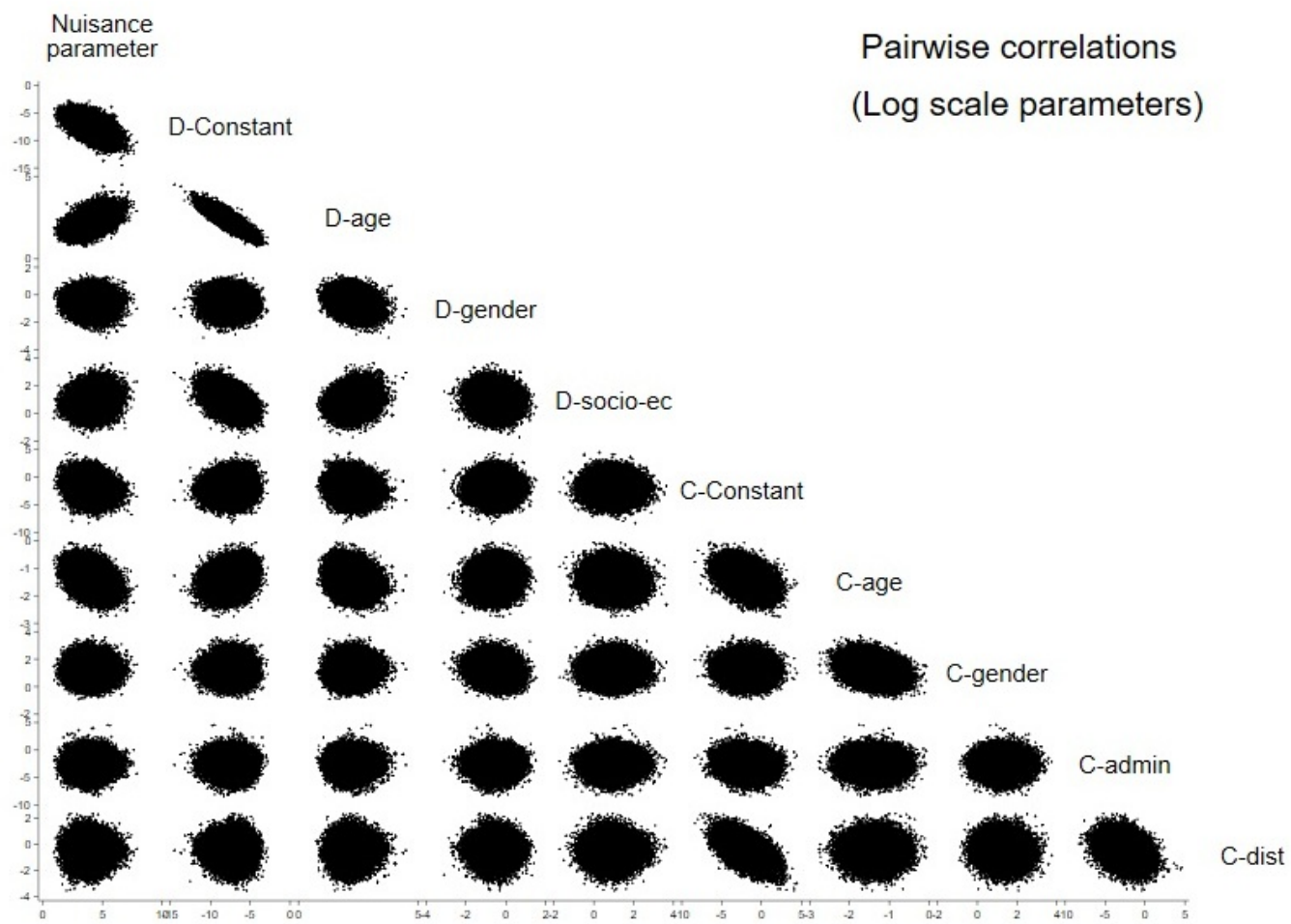

Supplement: Supplementary data [file bmjopen-2020-040735supp017.pdf]

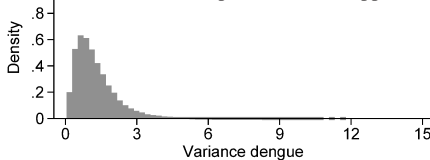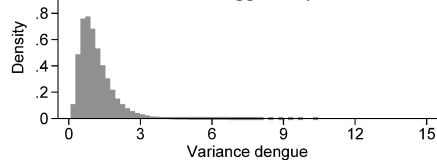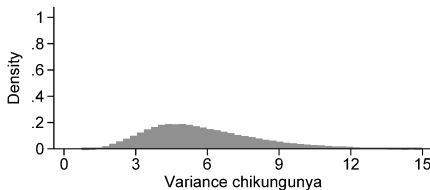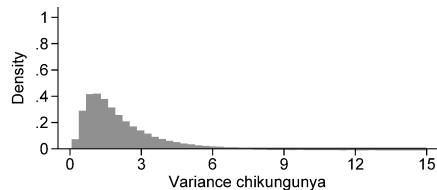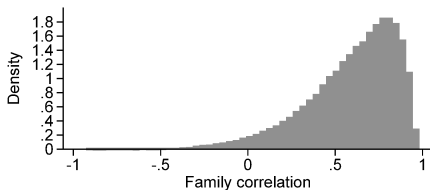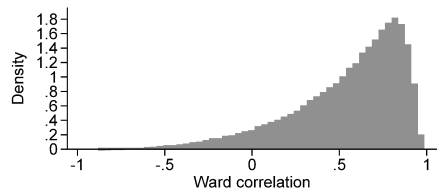

Supplement: Supplementary data [file bmjopen-2020-040735supp018.pdf]
