# Supplementary material for: Age-dependent seroprevalence of dengue and chikungunya: inference from a cross-sectional analysis in Esmeraldas Province in coastal Ecuador
Source: BMJ Open. 2020 Oct 16;10(10):e040735. doi: 10.1136/bmjopen-2020-040735 (PMC7569951; doi:10.1136/bmjopen-2020-040735)
Supplement: Supplementary data [file bmjopen-2020-040735supp007.pdf]

Age Group	Age distribution of census population in Esmeraldas		Age distribution of serological sample		Age-adjusted weights=
	Numbers	Proportions	Numbers	Proportions	
0-9	32452	0.211	147	0.461	**0.458**
10-19	33798	0.219	7	0.022	**9.999**
20-29	26507	0.172	46	0.144	**1.193**
30-39	20092	0.130	79	0.248	**0.527**
40-49	16477	0.107	33	0.103	**1.034**
50-59	12279	0.080	4	0.013	**6.357**
60+	12430	0.081	3	0.009	**8.581**

Supplementary Table 4
