# Supplementary material for: Age-dependent seroprevalence of dengue and chikungunya: inference from a cross-sectional analysis in Esmeraldas Province in coastal Ecuador
Source: BMJ Open. 2020 Oct 16;10(10):e040735. doi: 10.1136/bmjopen-2020-040735 (PMC7569951; doi:10.1136/bmjopen-2020-040735)
Supplement: Supplementary data [file bmjopen-2020-040735supp008.pdf]

OUTCOME	CATEG	MEAN	SD	LOWER 95% CrI	MEDIAN	UPPER 95% CrI
ALL						
		MEAN	SD	P025	MEDIAN	P975
**D+C-**	**ALL**	0.58	0.09	0.38	0.59	0.74
**D-C+**		0.01	0.01	0.00	0.01	0.03
**D+C+**		0.26	0.11	0.08	0.25	0.50
**D+**		0.84	0.04	0.75	0.84	0.91
**C+**		0.27	0.11	0.09	0.27	0.52
**BY SEX**						
**D+C-**	**MALE**	0.69	0.08	0.51	0.70	0.81
	**FEMALE**	0.53	0.10	0.32	0.54	0.72
**D-C+**	**MALE**	0.008	0.01	0.001	0.01	0.023
	**FEMALE**	0.018	0.01	0.004	0.02	0.04
**D+C+**	**MALE**	0.13	0.09	0.03	0.12	0.33
	**FEMALE**	0.32	0.12	0.11	0.31	0.57
**SEX DIFFERENCE**						
**D+C-**	**F - M**	-0.15	0.07	-0.31	-0.15	-0.018
**D-C+**		0.01	0.007	0.00	0.009	0.03
**D+C+**		0.18	0.09	0.04	0.18	0.37
**BY AREA OF RESIDENCE**						
**D+C-**	**URBAN**	0.46	0.12	0.23	0.47	0.68
	**RURAL**	0.68	0.09	0.39	0.70	0.86
**D-C+**	**URBAN**	0.017	0.01	0.004	0.015	0.04
	**RURAL**	0.006	0.005	0.00	0.005	0.02
**D+C+**	**URBAN**	0.39	0.14	0.13	0.38	0.68
	**RURAL**	0.21	0.13	0.03	0.18	0.53
**AREA OF RESIDENCE DIFFERENCES**						
**D+C-**	**U - R**	-0.21	0.16	-0.52	-0.22	0.13
**D-C+**		0.01	0.01	-0.004	0.01	0.03
**D+C+**		0.18	0.18	-0.21	0.18	0.53
**BY SOCIOECONOMIC STATUS**						
**D+C-**	**LOW**	0.61	0.11	0.37	0.63	0.79
	**HIGH**	0.51	0.13	0.24	0.52	0.73
**D-C+**	**LOW**	0.009	0.006	0.001	0.007	0.03
	**HIGH**	0.016	0.01	0.002	0.013	0.04
**D+C+**	**LOW**	0.26	0.12	0.07	0.24	0.52
	**HIGH**	0.28	0.15	0.06	0.26	0.61
**SOCIOECONOMIC STATUS DIFFERENCES**						
**D+C-**	**L-H**	0.10	0.14	-0.18	-0.10	0.39
**D-C+**		-0.006	0.01	-0.07	0.00	0.008
**D+C+**		-0.017	0.16	-0.35	0.02	0.29

Supplementary Table 5
