# Supplementary material for: Age-dependent seroprevalence of dengue and chikungunya: inference from a cross-sectional analysis in Esmeraldas Province in coastal Ecuador
Source: BMJ Open. 2020 Oct 16;10(10):e040735. doi: 10.1136/bmjopen-2020-040735 (PMC7569951; doi:10.1136/bmjopen-2020-040735)
Supplement: Author's manuscript [file bmjopen-2020-040735.draft_revisions.pdf]

BMJ Open

BMJ Open is committed to open peer review. As part of this commitment we make the peer review history of every article we publish publicly available.

When an article is published we post the peer reviewers' comments and the authors' responses online. We also post the versions of the paper that were used during peer review. These are the versions that the peer review comments apply to.

The versions of the paper that follow are the versions that were submitted during the peer review process. They are not the versions of record or the final published versions. They should not be cited or distributed as the published version of this manuscript.

BMJ Open is an open access journal and the full, final, typeset and author-corrected version of record of the manuscript is available on our site with no access controls, subscription charges or pay-per-view fees (http://bmjopen.bmj.com).

If you have any questions on BMJ Open's open peer review process please email info.bmjopen@bmj.com

Age-dependent seroprevalence of dengue and chikungunya in coastal Ecuador

Journal:	*BMJ Open*
Manuscript ID	bmjopen-2020-040735
Article Type:	Original research
Date Submitted by the Author:	21-May-2020

Complete List of Authors:	Chis Ster, Irina; St. George's University of London, ; St George's University of London<br>Rodriguez, Alejandro<br>Romero, Natalia; Universidad Internacional del Ecuador, Quito<br>Lopez, Andrea; Universidad Internacional del Ecuador, Quito<br>Chico, Martha; Universidad Internacional del Ecuador, Quito<br>Montgomery, Joel ; CDC<br>Cooper, Philip; St George's University of London, Institute of Infection and Immunity

Keywords:	Epidemiology < TROPICAL MEDICINE, STATISTICS & RESEARCH METHODS, PUBLIC HEALTH

SCHOLARONE™
Manuscripts

I, the Submitting Author has the right to grant and does grant on behalf of all authors of the Work (as defined in the below author licence), an exclusive licence and/or a non-exclusive licence for contributions from authors who are: i) UK Crown employees; ii) where BMJ has agreed a CC-BY licence shall apply, and/or iii) in accordance with the terms applicable for US Federal Government officers or employees acting as part of their official duties; on a worldwide, perpetual, irrevocable, royalty-free basis to BMJ Publishing Group Ltd ("BMJ") its licensees and where the relevant Journal is co-owned by BMJ to the co-owners of the Journal, to publish the Work in this journal and any other BMJ products and to exploit all rights, as set out in our licence.

The Submitting Author accepts and understands that any supply made under these terms is made by BMJ to the Submitting Author unless you are acting as an employee on behalf of your employer or a postgraduate student of an affiliated institution which is paying any applicable article publishing charge ("APC") for Open Access articles. Where the Submitting Author wishes to make the Work available on an Open Access basis (and intends to pay the relevant APC), the terms of reuse of such Open Access shall be governed by a Creative Commons licence – details of these licences and which Creative Commons licence will apply to this Work are set out in our licence referred to above.

Other than as permitted in any relevant BMJ Author's Self Archiving Policies, I confirm this Work has not been accepted for publication elsewhere, is not being considered for publication elsewhere and does not duplicate material already published. I confirm all authors consent to publication of this Work and authorise the granting of this licence.

For peer review only

Age-dependent seroprevalence of dengue and chikungunya in coastal Ecuador

Irina Chis Ster[1], Alejandro Rodriguez[2], Natalia Romero[2,5], Andrea Lopez[2], Martha E Chico[3], Joel Montgomery[4], Philip J Cooper[1-3].

[1]Institute of Infection and Immunity, *St George's University of London, London SW17 ORE*, United Kingdom; [2]School of Medicine, *Universidad Internacional del Ecuador, Quito, Ecuador*; [3]*Fundacion Ecuatoriana Para Investigacion en Salud, Quito, Ecuador*; [4]*Centers for Disease Control and Prevention*, Atlanta, Georgia, USA; [5]*Grups* de *Recerca d'America* i *Africa Llatines*.

For peer review only

Conflict of Interests: NONE declared.

Correspondence: Dr Irina Chis Ster, (ichisste@sgul.ac.uk)

Dr Irina Chis Ster

Senior Lecturer in Biostatistics

Institute of Infection and Immunity, St George's University of London

Cranmer Terrace | Tooting | London | SW17 0RE

Tel: +44 (0)208 725 4937

Email: i.chisster@sgul.ac.uk

Contributors:

For peer review only - http://bmjopen.bmj.com/site/about/guidelines.xhtml

Contributors: PJC and ICS designed the study. PJC and MEC supervised data and sample collection. AL did the laboratory assays. ICS developed and implemented the statistical methodology and conducted the literature research. AR provided data and contributed to spatial data analyses. ICS and PJC drafted the manuscript. NR and JM reviewed and provided critical comments on the manuscript. All authors reviewed and approved the final version of the manuscript. ICS is the paper guarantor.

For peer review only

For peer review only - http://bmjopen.bmj.com/site/about/guidelines.xhtml

Abstract

Objectives: There are few population-based estimates for prevalence of past exposure to dengue and chikungunya viruses despite common epidemiological features. Here, we have developed a novel statistical method to study patterns of age-dependent prevalence of immunity in a population following exposures to two viruses which share similar epidemiological features including mode of transmission and induce long-lasting immunity. This statistical technique accounted for socio-demographic characteristics associated with individuals and households.

Settings: The data represent a random sample from an ongoing longitudinal birth cohort set up in a tropical District in coastal Ecuador (Esmeraldas).

Participants: We collected data and blood samples from 319 individuals belonging to 152 households following epidemics of the infections in 2015 in Latin America.

Primary outcome: Plasma was tested for the presence of specific IgG antibodies to dengue and chikungunya viruses by commercial enzyme-linked immunosorbent assays and defined a bivariate binary outcome indicating individuals' past exposure status to dengue and chikungunya (i.e. presence/absence of IgG antibodies to dengue or chikungunya or both).

Results: Dengue seroprevalence increased rapidly with age reaching 97% (95%CrI 93%-99%) by 60 years. Chikungunya seroprevalence peaked at 42% (95%CrI 18%-66%) around 9 years of age and averaged 27% (95%CrI 8.7%-51.6%) for all ages. Rural areas were more likely to be associated with dengue only exposure while urban areas and shorter distance to the nearest household were associated with exposures to both. Females living in urban settings were more likely to be chikungunya seropositive while rural males were more likely to be dengue seropositive.

For peer review only - http://bmjopen.bmj.com/site/about/guidelines.xhtml

Conclusion: Dengue seroprevalence was strongly age-dependent consistent with endemic exposure while that of chikungunya peaked in childhood consistent with the recent emergence of the virus in the study area. Our findings will inform control strategies for the two arboviruses in Ecuador including recommendations by WHO on dengue vaccination.

Strengths and limitations of this study

- The arboviruses, dengue and chikungunya, are transmitted by the same vectors and share similar epidemiological characteristics. There are limited data on age-dependent seroprevalence for these arboviruses from the Americas and no previous population-based estimates on co-exposures in the same population in any region of Ecuador.
- The paper presents a novel and easily applicable (OpenBUGS code provided) statistical method which addresses the more general question of inferring age-dependent prevalence of past exposure to two related viruses which result in long lasting immunity. The method can easily be generalized to three exposures, given the more recent emergence of Zika virus, another arbovirus circulating in the Latin American region and transmitted by the same vectors.
- The estimates account for individual and household characteristics and reveal different patterns in the population regarding the past exposures to the two viruses
- Recent guidelines call for understanding local epidemiology of dengue prior to designing efficient control strategies including vaccination.
- More research using mathematical modelling is needed to assess the impact of various control strategies in the community using these estimates and their uncertainties

Ethics

Informed written consent was obtained from participants aged 18 or more, and from a parent or legal representative for a minor. Minor assent was obtained from children aged 8 years or

For peer review only - http://bmjopen.bmj.com/site/about/guidelines.xhtml

older. The study protocol was approved by the Ethics Committee of the Universidad Internacional del Ecuador, Quito, Ecuador.

Acknowledgements

We thank to all participants and their families who agreed to participate in the Ecuavida cohort.

Public benefit

The international steering committee of dengue virus called for studies to improve our understanding of the local epidemiology of dengue to guide the design of locally relevant and appropriate control programmes. This study provides estimates on age-dependent seroprevalence for dengue (and chikungunya) and epidemiological determinants of these exposures from a tropical region of coastal Ecuador. Our data are relevant to public health authorities locally and in similar regions in Latin America and will inform control strategies.

For peer review only

Introduction

Dengue virus (DENV) and chikungunya virus (CHIKV), arboviruses belonging to *Flaviviridae* and *Togaviridae* families, respectively, are endemic in many tropical and sub-tropical regions where *Aedes* vectors thrive. Although the two viruses belong to different arbovirus families *[1]*, human infections are associated with similar clinical manifestations and are a major cause of morbidity *[2-4]*. Over the past 15 years, the number of dengue cases reported in Latin America has increased dramatically *[5]* while chikungunya, which emerged in the Caribbean region in 2013, has spread rapidly through the region *[6]* causing an estimated 2.9 million cases *[7-9]*.

Global estimates of the burden of dengue and chikungunya are based on aggregate numbers of reported cases and likely to be biased by underreporting because of limited surveillance, high rates of asymptomatic infections, and non-specific clinical presentation *[10]*. Asymptomatic dengue, for example, can inflate the total number of cases by up to 40-fold *[11, 12]*.

Cross-sectional serological surveys can provide valuable information on arbovirus infection burdens and allow more accurate determination of numbers exposed. Serological surveys, thus, have a number of advantages over clinically classified, self-reported cases or other non-objective clinical diagnoses by providing information that are less sensitive to recall, underreporting, and misdiagnosis due to asymptomatic manifestations of related infections *[6, 13]*.

Ecuador is a middle-income country in Latin America where dengue has been endemic since the late 1980s *[14]*: dengue is thought to have re-emerged around 1988 following re-colonisation with *A. aegypti* after a thirty-year absence *[15, 16]*. Chikungunya first appeared

For peer review only - http://bmjopen.bmj.com/site/about/guidelines.xhtml

in the Caribbean region in 2013, reaching Ecuador in 2014 causing a large outbreak the following year *[16] [17]*.

Dengue transmission dynamics has been estimated from aggregated data in a series of studies *[18, 19]* with the latter suggesting that the picture provided by serology is very incomplete and limited when trying to characterize transmission across time and space both within and between countries and calling for local data analyses.

Most seroprevalence studies for DENV and CHIKV have examined exposures to each in isolation despite evidence *[20]* of co-circulation and shared risk factors *[6, 21, 22]*. There are no published serological surveys of dengue and chikungunya in Ecuador or studies of co-exposures to the two viruses from the Latin American region *[6]*, and limited information on the determinants of exposure risk. Such data are critical to estimate the burden of infection and inform public health strategies for control. The first licenced dengue vaccine (CYD-TDV) is now available in the Latin American region. Current WHO guidelines advise the implementation of cost-effective prevention strategies that consider local arbovirus epidemiology *[23]*. Recent studies indicated that targeting only seropositive individuals for vaccination might reduce global annual disease incidence by 20 to 30%, with the greatest impact in high-transmission settings *[24]*. Hence, understanding past exposure or seropositivity patterns in affected populations are key to implementing cost-effective control strategies.

In this study, we hypothesised that prevalence of past exposure to DENV and CHIKV in the population are associated with age, and individual and household risk factors. We estimated age-dependent seroprevalence of DENV, CHIKV and co-exposures with both arboviruses in a district in coastal Ecuador, and explored individual and household determinants of past exposure status.

For peer review only - http://bmjopen.bmj.com/site/about/guidelines.xhtml

Materials and methods

Population and study sample

We sampled children (and their households) that had been recruited into a birth cohort (ECUAVIDA) between 2005 and 2009 in the town of Quininde, Esmeraldas Province *[25]*. The District is largely rural in a tropical region of coastal Ecuador with a population of 88,000 living below 150 m altitude with mean annual temperature of 30ºC and relative humidity of 80%. Main sources of income are cultivation of palm oil and tropical fruits, and timber extraction. Cohort children reaching 8 years and household members were invited to attend a visit at the cohort clinic in Quininde between May and September 2016. Venous blood was collected into heparinized tubes (Vaccutainer, BD Systems), centrifuged and plasma was stored at -20C. Data on individual and household factors were collected from the child's mother by an investigator-administered questionnaire and the 2010 census population data was used to derive post-stratification weights such that the resulting estimates would represent those of the population *[26]*. Each household was visited and location determined by GPS (Garmin eTrex, KS, USA).

Laboratory testing

Plasma was analysed for specific IgG antibodies to DENV and CHIKV viruses using commercial ELISA kits (Human anti-Dengue IgG and human anti-Chikungunya IgG ELISA kits, Abcam, Cambridge UK) following manufacturer's instructions. Past exposure or seropositivity to DENV and CHIKV was defined by the presence of specific IgG antibodies.

Statistical methods

The presence or absence of DENV and CHIKV antibodies define a bivariate binary outcome indicating past exposure to DENV only denoted by D+C-, to CHICK only denoted by D-C+,

For peer review only - http://bmjopen.bmj.com/site/about/guidelines.xhtml

to both, D+C+, or to none. The key assumption of the model is that of long-lasting immunity *[18]* after infection with DENV and CHIKV.

We used two simultaneous multilevel logistic regression equations with shared random effects to model the presence/absence of antibodies for DENV or CHIKV or both. The basic idea of this model was developed initially in econometrics *[27]* and more recently in the context of two binary correlated outcomes indicating infections but using a distinct approach from the one used here *[28]*. Each equation modelled the presence of one arbovirus conditioned on the other, making the presence of DENV more or less likely in the presence of CHIKV (and vice versa). We explored the effects of age, sex, area of residence (urban vs. rural), socioeconomic level (grouped as low, medium, and high derived from tertiles of the first component of principal components analysis of 7 socio-economic variables as described in *[29]*), crowding (number of persons per sleeping room) and household dispersal (Euclidian distance to nearest household using GPS coordinates) on our bivariate binary outcome (dependent variable) defined above. The simplest model started with investigating the effect of age on the outcome which provided answers to the main question of interest, i.e. how the probabilities of past exposures to (D+C-, D-C+ and D+C+) varied with age in this population. The systematic trends with age in these probabilities are called *age-dependent prevalence of past exposure* to D+C-, D-C+ and D+C+. They are also predicted probabilities of past exposures. We then refined these findings by exploring the extent to which these overall associations between past exposure to D+C-, D-C+ and D+C+ and age are altered by the other variables – alone and in a multivariable model.

The model (Supplementary file) also embedded post-stratification weights, allowing estimates to be generalizable to the population from which the sample was derived *[26]*. Hierarchical structure *[30]* of the data (individuals within families within wards) was represented by shared random effects, accounting for unmeasured epidemiological factors such as biting rates or climate conditions. Because of the assumption of long-life immunity, the age-dependent

For peer review only - http://bmjopen.bmj.com/site/about/guidelines.xhtml

prevalence of past specific or co-exposures *[13]* can be also regarded as measures of immunity to these viruses in the population.

Bayesian inference was used for parameter estimation and to predict age-dependent probabilities of seropositivity and 95% credible intervals (CrI). In the absence of any formal goodness-of-fit measure, predicted age dependent prevalence was plotted against the observed proportions of positives within the same 5-year age group. Moreover, we also plotted the observed numbers of seropositive individuals across 5-year groupings and across geographical regions against expected numbers predicted by the model.

Detailed information on the statistical approach used with mathematical models, framework for statistical inference, estimated parameters and goodness of fit are provided as Supplementary files. Aggregated data at national and provincial levels for case reports of dengue/chikungunya from 2015 were retrieved from various sources *[15, 16, 31, 32]*. Given the assumption of life-long immunity, age-dependent seroprevalence and risk or probability of past exposures to DENV and CHICKV, are used interchangeably.

Results

Figure 1 shows the geographical locations of households of children in the background cohort and the locations of those sampled for this analysis (151). There was an almost 3-fold increase in the number of reported DENV cases (42,499 cases) in 2015 compared to 2014 (15,584) and 2016 (13,612). An outbreak of CHIKV of comparable size (34,101 cases) emerged in 2015 after no previously documented history of infection in the country before 2014: Esmeraldas province accounted for a third (10,477) of CHIKV cases in 2015 reported nationally (Supplementary Table 1 and Supplementary Figure 1 [showing monthly time-series of cases]).

For peer review only - http://bmjopen.bmj.com/site/about/guidelines.xhtml

Plasma from blood samples collected between September and December 2015 from 319 individuals (including children) belonging to 151 households from 49 wards (electoral sub-divisions at parish level) were analysed. Characteristics for subjects and their households are provided in Table 1. Of 319 individuals, 245 (76.8%) were seropositive for DENV and 145 (45%) were seropositive for CHIKV. High numbers/proportions were positive for both arboviruses: 129 (40%) individuals belonging to 86 (57%) families living in 24 (49%) wards. Proportions of households and wards with at least one seropositive individual were: 143 (95%) households in 46 (94%) wards for DENV and 93 (62%) households in 27 (55%) wards for CHIKV.

The sub-sample of the cohort used in this analysis appeared to represent the overall cohort well in terms of socioeconomic status and area of residence (Supplementary Table 2). Distributions by parish and age for the census population of Esmeraldas Province, cohort, and serological sample are shown in Supplementary Table 3 and Supplementary Table 4, respectively, along with stratum-specific weights subsequently used in modelling for population-level representativeness. Parameter estimates (conditional odds ratios) which measure the univariate and adjusted associations of the risk of testing positive for DENV, CHIKV and both with each potential risk factor (age, sex, area of residence, socio-economic status and distance to next door neighbour) are shown in Table 2. Given the relative complexity of this non-linear modelling, the results are best understood through a predicted average seroprevalence across the population, with reference to trends and patterns by age and household characteristics (Supplementary Table 5).

Average overall DENV seroprevalence (including those seropositive for both viruses) was 84% (95% CrI 75%-91%) but increased rapidly with age from 14% (95% CrI 1.5-45%) in infants to 97% (95% CrI 93-99%) in adults aged 60 years (*Figure 2*). The overall average seroprevalence

For peer review only - http://bmjopen.bmj.com/site/about/guidelines.xhtml

of CHIKV was 27% (95% CrI 8.7%-51.6%), peaked at 42% (95% CrI 18%-66%) in children aged 9 years, and declined to 18% (95% CrI (5%-38%) by 60 years (*Figure 2*).

Age-dependent prevalence curves for D+C-, D-C+, D+C+ and actual proportions from raw data (bars) for 5-year age groupings are shown *Figure 3*. The predicted curves fitted observed data well: raw proportions of positives were generally close to the 95% CrI predicted curves. Average seroprevalence of D+C- was 58% (95% CrI 38-74%), that of D-C+ was 1% (95% CrI 0-3%) and that of D+C+ was 26% (95% CrI 8-50%) (Supplementary Table 5). Seroprevalence increased rapidly following a power law (i.e. faster than exponential) with age for D+C-, declined rapidly for D-C+, and increased rapidly to a peak around 9 years (40%, 95% CrI 18%-64%) for D+C+, followed by a steady decline to 18% (95% CrI 6-37%) by 60 years.

Average seroprevalence by groups defined by co-exposures including sex, area of residence, and socioeconomic status are shown in Supplementary Table 5 and their sole effects on age-specific prevalence in Table 2 and Supplementary Figure 2. Females were more likely to be seropositive for CHIKV (D+C+ and D-C+) but less likely to be singly positive to DENV (D+C). The difference in D+C- seroprevalence between males and females was greatest in adolescence while that for D+C+ was greatest in childhood (*Supplementary Figure 3*). Individuals resident in rural areas were more likely to be D+C- while D+C+ was more frequent in urban areas; while shorter distance to nearest household (i.e. household dispersal) appeared to be a risk factor for D+C+.

Strength of associations were preserved in the multivariable model but estimates were less precise as more information was extracted, i.e. the CrIs were wider (Final Model in Table 2). Males remained more likely to be D+C- and less likely to be D+C+ (*Supplementary Figure 4*); individuals living in rural areas were more likely to be D+C- while those living in urban areas were more likely to be D+C+ (*Supplementary Figure 5*); and individuals of lower

For peer review only - http://bmjopen.bmj.com/site/about/guidelines.xhtml

economic status appeared to be more likely to have all outcomes across all ages although differences were greater among those aged less than 20 years (*Supplementary Figure 6*). *Supplementary Figure 7* and *Supplementary Figure 8* show further evidence for a good fit of the model to the numbers in the data rather than to proportions: group-specific defined by age and geographical regions predicted number of seropositive individuals were derived by averaging the age-specific proportions in that group and used to predict approximate average number of exposed individuals. All the parameter estimates MCMC traces for the most complex model (Table 2) and their correlations are presented in *Supplementary Figure 9 Supplementary Figure 9* and *Supplementary Figure 10*, respectively. The estimated variance-covariance matrices corresponding to the random effects associated with both ward and family structures can also be viewed in *Supplementary Figure 11*.

Discussion

Arbovirus infections are a growing public health threat in Latin America where DENV is endemic in tropical and sub-tropical regions, and CHIKV and Zika virus infections have recently emerged *[6]*. Despite evidence for co-circulation within the same geographic regions of more than one type of arbovirus, and in many regions all 3 viruses *[3, 9, 33]* that share the same vectors and cause similar symptomatology *[34]*, there are no studies addressing the epidemiology of co-exposures. Nevertheless, the epidemiological and statistical methodologies developed here can be made to accommodate relatively easily more than two concomitant exposures. Although DENV has been endemic in Ecuador since 1980, there are no previous seroprevalence estimates from Ecuador for either arbovirus *[6]*.

In the present study, we estimated that 84% (95%CrI 75%-91%) of individuals aged 0 to 60 years had been exposed to DENV in a tropical region of coastal Ecuador. However, DENV seroprevalence increased rapidly with age reaching 97% (95% CrI 93%-99%) by 60 years,

For peer review only

reflecting cumulative exposures over a 30-year period since DENV re-emerged in the region in the late 1980s *[17]*. Placing this estimate within a global context, it concurs with a previous systematic review *[6]* indicating a high average seroprevalence in the Caribbean and Latin American regions (~65%) compared to Asia (46%) and Africa (18%).

Mean chikungunya seroprevalence was 27% (95% CrI 8.7%-51.6%) in this population and is within the range of seroprevalence estimates from seven previous studies from Latin America: Puerto Rico (23.5%), Guadeloupe (48.1%), Martinique (41.9%), Saint Martin (16.9%), Nicaragua (32.8%) and Brazil (20%) *[35]*. A study of two urban populations in Brazil estimated CHIKV seroprevalence at 57.1% and 45.7%, respectively *[35]* while seroprevalence in a rural area of Chapada district (Brazil) was estimated at 20% (95% CrI, 15.4–35%) *[36]*. A seroprevalence of CHIKV of 75% was observed in urban children in Haiti and French Polynesia *[6]*. Our data showed a much flatter seroprevalence pattern by age with a peak around 9 years, likely reflecting a single intense exposure period during 2015.

In the present analysis, we developed statistical methodology to address analysis of exposures to co-circulating viruses which share strong epidemiological similarities. This method was applied to a bivariate binary outcome defined by the presence/absence of specific IgG antibodies in sera from a population sample weighted to represent the source population, and aimed at understanding how patterns of age-dependent seroprevalence vary by individual and contextual factors. The model allowed prediction of 4 possible outcomes (i.e. D+C-, D-C+, D+C-, and D-C- [used as reference group]). D+C- was the most prevalent group in this population followed by D+C+ and D-C+ (*Figure 3*). The latter group was small and young with imprecise estimates. This statistical methodology show here can be applied relatively easily to more than two co-exposures in future studies.

For peer review only - http://bmjopen.bmj.com/site/about/guidelines.xhtml

Males were more likely to have been exposed to DENV while females had greater exposure to CHIKV, effects that were observed across all ages. Differences in exposure by sex could reflect temporal changes in transmission sources related to accelerating urbanisation in the context of women maintaining traditional roles as home keepers (i.e. transmission is largely within and between households, particularly in an urbanizing/urban setting). The observation that CHIKV seroprevalence was inversely associated with distance between households (e.g. a marker of greater population densities and urbanism) suggest that CHIKV exposures are more intense in crowded places, an observation not seen for DENV in this study. These data are consistent with an outbreak in 2012 in Bangladesh that showed a reduced probability of transmission of CHIKV with increasing distance between households *[37]*. An increased risk of infection among females from the same study was attributed to greater time spent around the house *[37]*. The primary vector, *Ae. aegypti*, is well adapted to urban living and breeds wherever clean water is present, particularly in open recipients around households.

The non-linear age-dependence of DENV seroprevalence is not unexpected given the period over which exposures are likely to have occurred. Our data indicate that by 60 years of age almost everyone has been infected at least once with a dengue serotype. In contrast, exposure to CHIKV, which first appeared in Ecuador in 2014, possibly after a long absence *[7]*, was shorter: CHIKV caused a large outbreak in 2015 in coastal Ecuador, particularly in Esmeraldas Province. Thus, the age-seroprevalence profile for chikungunya, unlike dengue for which age can be viewed as a measure of length of exposure, may reflect age-dependent differences in exposures to *Aedes* vectors or susceptibility to infection.

Current global estimates of DENV and CHIKV prevalence are uncertain, being largely based on case reports to public health authorities often by other diagnostic mechanisms than laboratory analyses. Our data support the usefulness of serological surveys to inform evaluations of burden of infection within countries, particularly those with limited health

For peer review only - http://bmjopen.bmj.com/site/about/guidelines.xhtml

resources. Further, local serological surveys will be useful for planning novel population-level interventions against arbovirus transmission such as vaccination. Dengvaxia is only currently registered dengue vaccine that might be used in endemic populations *[23]*. There has been extensive debate over dengue vaccination planning in the endemic countries: either vaccination of populations with dengue seroprevalence rates above 80% or screening for dengue-specific antibodies and vaccinating only seropositive individuals. The WHO scientific advisory working group concluded that both "mass vaccination based on population seroprevalence criteria" and "pre-vaccination screening" are difficult to implement and neither can achieve herd immunity but favours a pre-vaccination strategy in which only seropositive persons are vaccinated. A better understanding of the local epidemiology of dengue will be essential for the evaluation of cost-effectiveness of such measures *[23]*. Recent research suggested that highlighting areas of high prevalence is important as recent analyses suggested that transmission-blocking interventions such as Wolbachia, even at intermediate efficacy (50% transmission reduction), might reduce global annual disease incidence by up to 90%. The newly licensed Sanofi-Pasteur vaccine, targeting only seropositive recipients, (namely those at risk of secondary infection which can result in severe dengue and subsequent death), might reduce global annual disease incidence by 20 to 30%, with the greatest impact in high-transmission settings [24].

Limitations of seroprevalence estimates from the literature are small sample size and poorly representative samples *[6]*. A recent review of seroprevalence studies for DENV, CHIKV, and Zika identified 185 studies of which the majority were specific populations or age sub-groups (60%), and just over a third (38.9%) were samples from the general population *[6]* although few were representative samples. About half (50.3%) of the 185 studies had sample sizes of less than 500 subjects *[6]*. The sample size in this study likely limited our ability to detect effects of individual and contextual factors. We have minimized the potential bias in

For peer review only - http://bmjopen.bmj.com/site/about/guidelines.xhtml

using a sample from a birth cohort rather than a general population sample, by assigning post-stratification weights based on the 2010 census *[26]*.

Our modelling strategy was based on assumptions that: 1) mortality caused by either infection is negligible- case fatality rate in the America region for dengue is approximately 2.5% annually while that of chikungunya is less than 0.1% *[38, 39]*; and 2) both arboviruses induce long-lasting immunity *[40]* - while likely to be true for CHIKV, relevance to DENV is less clear given that immunity is strain-specific and infections with 4 different strains are possible *[41]*. Molecular analysis of dengue strains in Ecuador have shown all 4 strains to be circulating since before 2013 *[42]*. Further, our data can shed little light on vector competence which may affect the transmission of one or other arbovirus (or DENV serotype/genotype) if the two are circulating in the same population at the same point in time – there is some evidence for adaptation of CHIKV to *A. aegypti* and for displacement of one arbovirus by another within populations of *A. aegypti [43]*. Seroprevalence estimates here likely reflect cumulative exposures to all four DENV strains. Given relatively low case fatality rates of 2.5% for DENV (mostly related to severe dengue) and 0.1% for CHIKV *[40, 44]*, this assumption is reasonable. Although the serological tests used were highly sensitive, sensitivity may vary depending on whether infection is primary or secondary *[45]*. In the context of the present study, there was limited evidence of reduced sensitivity in older subjects (more likely to have secondary infections) among whom seroprevalence to DENV reached almost 100% by 60 years. The serological test used for CHIKV and DENV were highly specific: the study was done before the emergence of Zika virus which first appeared in Ecuador in 2016 and in the study area in May 2016 with which serological tests for DENV share significant serological cross-reactivity [46].

For peer review only - http://bmjopen.bmj.com/site/about/guidelines.xhtml

Conclusions

Our data, from a household sample weighted to represent the general population, showed strong age-dependence of DENV seroprevalence but not CHIKV which emerged only in 2015 in the study population in a tropical region of coastal Ecuador. Our study will inform policy makers on relevant control strategies in Ecuador and decisions on cost–effectiveness of vaccination strategies for dengue *[23]*. Highlighting areas of high prevalence is important as recent analyses suggested that transmission-blocking interventions such as *Wolbachia*, even at intermediate efficacy (50% transmission reduction), might reduce global annual disease incidence by up to 90% *[24]*. The newly licensed Sanofi-Pasteur vaccine, targeting only *seropositive* recipients, (namely those at risk of secondary infection which can result in severe dengue and subsequent death), might reduce global annual disease incidence by 20 to 30%, with the greatest impact in high-transmission settings *[24]*.

For peer review only

Tables

Characteristic	N (%)
Individual characteristics	N=319
Sex	
Male	117(37%)
Female	202(63%)
Age (years)	
0-5	7(2%)
6-10	140(44%)
11-15	4(1%)
16-20	3(1%)
21-25	12(4%)
26-30	45(14%)
31-35	37(12%)
36-40	41(13%)
41-45	15(5%)
46-50	9(3%)
>50	6(2%)
Dengue seropositive	
No	74 (32.2%)
Yes	245 (76.8%)
Chikungunya seropositive	
No	174 (54.5%)
Yes	145 (45.5%)
Household characteristics	N=151
Area of residence	
Rural	47 (32%)
Urban	104 (68%)
Socioeconomic status	
Low and medium	106 (70%)
High	45 (30%)
Household dispersal (metres)	
<50	13 (8.6%)
51-250	63(41.7%)
251-500	34(22.5%)
501-1,000	12(8%)
>1,000	29(19%)

Table 1. Characteristics of 319 individuals from 151 households in the region of the District of Quininde, Esmeraldas province.

For peer review only - http://bmjopen.bmj.com/site/about/guidelines.xhtml

	DENGUE CONDITIONED ON CHIKUNGUNYA						CHIKUNGUNYA CONDITIONED ON DENGUE					
MODEL	MEAN	SD	MEDIAN	P025	P975	P(OR<=1)/P(OR>1)	MEAN	SD	MEDIAN	P025	P975	P(OR<=1)/P(OR>1)
1st MODELS												
AGE ((POWER*)	7.671	3.123	7.023	3.616	15.450		0.357	0.097	0.346	0.202	0.579	
2nd MODELS												
FEMALE vs. MALE	0.728	0.373	0.650	0.248	1.665	**0.822/0.178**	2.971	1.587	2.614	1.030	7.004	**0.017/0.983**
RURAL vs. URBAN	1.490	1.431	1.099	0.260	5.020	0.441/0.559	0.252	0.337	0.159	0.023	1.040	**0.975/0.025**
CROWDING	1.217	0.744	1.042	0.348	3.118	0.476/0.524	0.916	0.759	0.718	0.179	2.808	0.679/0.321
LOW vs. HIGH	3.302	2.472	2.671	0.828	9.518	**0.052/0.948**	0.916	0.858	0.675	0.142	3.148	0.684/0.316
HOUSEHOLD	0.825	0.314	0.772	0.380	1.585	0.771/0.229	0.711	0.384	0.628	0.225	1.685	**0.826/0.174**
FINAL MODELS												
AGE (POWER)	9.980	4.623	8.955	4.290	21.760		0.263	0.085	0.251	0.132	0.460	
FEMALE vs. MALE	0.651	0.347	0.577	0.210	1.528	**0.87/0.13**	3.804	2.356	3.238	1.152	9.831	**0.01/0.99**
RURAL vs. URBAN							0.218	0.479	0.099	0.008	1.167	**0.98/0.02**
LOW vs. HIGH	3.028	1.992	2.536	0.827	8.085	**0.05/0.95**						
HOUSEHOLD							0.794	0.561	0.657	0.186	2.202	0.76/0.24

Table 2. Parameter estimates derived after fitting simultaneous logistic models with increasing number of explanatory variables. 1st Models have age only as predictor. 2nd Models show effects of each of age, sex, area of residence, socio-economic status, household overcrowding and household dispersal on the Odds Ratio of past exposure vs. no exposure to the viruses. Final models included all variables for which the effect in the simpler models, measured as P(OR>1) (or P(OR<1)), was greater than 0.80 or less than 0.20 (shown in bold). Age-dependence is parameterized according to a power law (i.e. age^{power}) and the corresponding estimate represented the *power* of the age^{power} component.

For peer review only

Supplementary Table 1. Total number of reported cases of dengue and chikungunya in Ecuador and Esmeraldas province in 2015 compared to the 2014 and 2016. Overall proportions of cases were calculated using Ecuador and Esmeraldas population total as denominator, respectively. Ecuador census data for 2010 were used and the totals were adjusted according to the annual predicted population growth.

Supplementary Table 2. Comparison between serological sample and cohort with respect to area of residence and socioeconomic status of households.

Supplementary Table 3. Geographical distribution of households by parish across region of Esmeraldas (census), cohort and data sample. The data sample distribution is similar to that of the cohort and the post-stratification weights adjusted for a fair representation of the Esmeraldas Province. The weights represent essentially the ratios between the census proportions and data proportions across each region. This calibration also adjusted for urban/rural area of residence.

Supplementary Table 4. Age distributions of Esmeraldas census population and serological sample from cohort. The weights used in modelling were the ratios between Esmeraldas census population distribution proportions and age-group proportions in the serological sample.

Supplementary Table 5. Average predicted seroprevalence (proportions) for each exposure category and their 95%Credible Intervals (CrI) for dengue and chikungunya and seroprevalence difference by sex (female [F] vs. males [M]), area of residence (urban [U] vs. rural [R]), and socioeconomic level (low [L] vs. high [H]). Exposure categories are: DENV+CHIKV- [D+C-]; DENV-CHIKV+ [D-C+]; and DENV+CHIKV+ [D+C+].

For peer review only - http://bmjopen.bmj.com/site/about/guidelines.xhtml

Figures

Figure 1. Geographic distribution of the household locations of the background population (ECUAVIDA) and the serological sample.

Figure 2. Predicted age-dependent seroprevalence for dengue and chikungunya. Dashed lines represent the 95% Credible Intervals and the bars represent the observe proportions of seropositive participants.

Figure 3. Predicted age-dependent prevalence for exposure categories to dengue and chikungunya in the sample population plotted against observed proportions (in 5-year age groupings). Dashed lines represent 95% Credible Intervals.

Supplementary Figure 1. Reported clinical cases of dengue and chikungunya in 2015 in Ecuador nationally and in the Province of Esmeraldas. Time-series graphs were derived from data publicly accessible (17, 18, 19, 20)

Supplementary Figure 2. Predicted age-dependent seroprevalence for exposure groups to dengue and chikungunya for categories of area of residence, sex, and household dispersal in study sample. Exposure categories are: DENV+CHIKV- [D+C-]; DENV-CHIKV+ [D-C+]; and DENV+CHIKV+ [D+C+]. Dashed lines represent the 95% Credible Intervals.

For peer review only

Supplementary Figure 3. Predicted age-dependent differences for exposure categories by sex (A), area of residence (B), and socioeconomic status (C). Exposure categories were DENV+CHIKV- [D+C-]; DENV-CHIKV+ [D-C+]; and DENV+CHIKV+ [D+C+]. Dashed lines represent 95% Credible Intervals.

Supplementary Figure 4 Age-dependent seroprevalence for exposure groups to dengue and chikungunya stratified by sex, socioeconomic status, and area of residence. Exposure categories were DENV+CHIKV- [D+C-]; DENV-CHIKV+ [D-C+]; and DENV+CHIKV+ [D+C+]. Dashed lines represent the 95% Credible Intervals of the predictions derived from the multivariable model

Supplementary Figure 5. Age-dependent seroprevalence for exposure groups to dengue and chikungunya stratified by area of residence, sex, and socioeconomic status. Exposure categories were DENV+CHIKV- [D+C-]; DENV-CHIKV+ [D-C+]; and DENV+CHIKV+ [D+C+]. Dashed lines represent the 95% Credible Intervals of the predictions derived from the multivariable model (Table 2).

Supplementary Figure 6. Age-dependent seroprevalence for exposure groups to dengue and chikungunya stratified by socioeconomic status, sex, and area of residence. Exposure categories were DENV+CHIKV- [D+C-]; DENV-CHIKV+ [D-C+]; and DENV+CHIKV+ [D+C+]. Dashed lines represent the 95% Credible Intervals of the predictions derived from the multivariable model (Table 2).

For peer review only

Supplementary Figure 7. Model goodness of fit: the observed seropositive numbers in each 5-year age group vs. the expected numbers calculated using average age-specific MCMC predictions and the total number in the corresponding age group: (a) for D+C-, (b) for D-C+ and (c) for D+C+.

Supplementary Figure 8 Model goodness of fit: the observed seropositive numbers in groups defined by geographical regions vs. the corresponding expected numbers calculated using MCMC predicted prevalence in that group and its total numbers: (a) for all DENV and (b) for all CHIKV.

Supplementary Figure 9. Parameter estimates for the most complex models - the MCMC traces for 100000 iterations.

Supplementary Figure 10. Parameter correlations based on the MCMC traces for 100000 iterations.

Supplementary Figure 11 Variance-covariance components (random effects) estimation associated with hierarchical structure of the data (family and ward).

Financial support

The project was supported by the Wellcome Trust (088862/Z/09/Z) and the Universidad Internacional del Ecuador.

Data sharing statement

For peer review only - http://bmjopen.bmj.com/site/about/guidelines.xhtml

The data were from a longitudinal birth cohort study and are available upon request to pcooper@sgul.ac.uk

Patient and Public Involvement statement

Informed written consent and minor assent was obtained to collect data and samples from study participants. At the time of consent, the public health benefits of the study were explained to participants.

For peer review only

References

1. Torres, M.C., et al., *Re-introduction of dengue virus serotype 2 in the state of Rio de Janeiro after almost a decade of epidemiological silence.* PLOS ONE, 2019. **14**(12): p. e0225879.
2. Brathwaite Dick, O., et al., *The History of Dengue Outbreaks in the Americas.* The American Journal of Tropical Medicine and Hygiene, 2012. **87**(4): p. 584-593.
3. Ding, F., et al., *Mapping the spatial distribution of Aedes aegypti and Aedes albopictus.* Acta Tropica, 2018. **178**: p. 155-162.
4. Kraemer, M.U.G., et al., *The global distribution of the arbovirus vectors Aedes aegypti and Ae. albopictus.* eLife, 2015. **4**: p. e08347.
5. Brady, O.J., et al., *Refining the Global Spatial Limits of Dengue Virus Transmission by Evidence-Based Consensus.* PLOS Neglected Tropical Diseases, 2012. **6**(8): p. e1760.
6. Fritzell, C., et al., *Current challenges and implications for dengue, chikungunya and Zika seroprevalence studies worldwide: A scoping review.* PLoS Neglected Tropical Diseases, 2018. **12**(7).
7. Scott, B.H., *Reappearance of Chikungunya, Formerly Called Dengue, in the Americas.* Emerging Infectious Disease journal, 2015. **21**(4): p. 561.
8. Yactayo, S., et al., *Epidemiology of Chikungunya in the Americas.* The Journal of infectious diseases, 2016. **214**(suppl 5): p. S441-S445.
9. Wahid, B., et al., *Global expansion of chikungunya virus: mapping the 64-year history.* International Journal of Infectious Diseases, 2017. **58**: p. 69-76.
10. Romero-Sandoval, N., et al., *Critical analysis of the definition of severe dengue, based on the Ecuador outbreak in 2012*. Vol. 04. 2014. 36-38.
11. Balmaseda, A., et al., *Trends in patterns of dengue transmission over four years of a pediatric cohort study in Nicaragua.* The Journal of infectious diseases, 2010. **201**(1): p. 5-14.
12. Morrison, A.C., et al., *Epidemiology of Dengue Virus in Iquitos, Peru 1999 to 2005: Interepidemic and Epidemic Patterns of Transmission.* PLOS Neglected Tropical Diseases, 2010. **4**(5): p. e670.
13. Chis Ster, I., *Inference for serological surveys investigating past exposures to infections resulting in long-lasting immunity – an approach using finite mixture models with concomitant information.* Journal of Applied Statistics, 2012. **39**(11): p. 2523-2542.
14. CDC. *International Notes Dengue Epidemic -- Ecuador*. 1988; Available from: https://www.cdc.gov/mmwr/preview/mmwrhtml/00001411.htm.
15. PÚBLICA, S.N.D.V.-M.D.S. *Dengue in Ecuador*. Available from: https://www.salud.gob.ec/wp-content/uploads/2013/02/Gecata-Dengue-SE-15.pdf.
16. PÚBLICA, S.N.D.V.-M.D.S. *Chickungunya in Ecuador*. Available from: https://www.salud.gob.ec/wp-content/uploads/2017/07/GACETA-VECTORES-SE-1.pdf.
17. Stewart-Ibarra, A.M., et al., *The high burden of dengue and chikungunya in southern coastal Ecuador: Epidemiology, clinical presentation, and phylogenetics from a prospective study in Machala in 2014 and 2015.* bioRxiv, 2017.
18. Imai, N., et al., *Estimating Dengue Transmission Intensity from Sero-Prevalence Surveys in Multiple Countries.* PLOS Neglected Tropical Diseases, 2015. **9**(4): p. e0003719.
19. Rodriguez-Barraquer, I., H. Salje, and D.A. Cummings, *Opportunities for improved surveillance and control of dengue from age-specific case data.* eLife, 2019. **8**: p. e45474.
20. Paixão, E.S., M.G. Teixeira, and L.C. Rodrigues, *Zika, chikungunya and dengue: the causes and threats of new and re-emerging arboviral diseases.* BMJ Global Health, 2018. **3**(Suppl 1): p. e000530.
21. Beltrán-Silva, S.L., et al., *Clinical and differential diagnosis: Dengue, chikungunya and Zika.* Revista Médica del Hospital General de México, 2016.
22. Rodríguez-Barraquer, I., et al., *The Hidden Burden of Dengue and Chikungunya in Chennai, India.* PLoS Negl Trop Dis, 2015. **9**(7): p. e0003906.

For peer review only - http://bmjopen.bmj.com/site/about/guidelines.xhtml

23. Wilder-Smith, A., et al., *Deliberations of the Strategic Advisory Group of Experts on Immunization on the use of CYD-TDV dengue vaccine.* The Lancet Infectious Diseases, 2019. **19**(1): p. e31-e38.
24. Cattarino, L., et al., *Mapping global variation in dengue transmission intensity.* Science Translational Medicine, 2020. **12**(528): p. eaax4144.
25. Cooper, P.J., et al., *Cohort Profile: The Ecuador Life (ECUAVIDA) study in Esmeraldas Province, Ecuador.* International Journal of Epidemiology, 2015. **44**(5): p. 1517-1527.
26. Little, R.J.A., *Post-Stratification: A Modeler's Perspective.* Journal of the American Statistical Association, 1993. **88**(423): p. 1001-1012.
27. Schmidt, P. and R.P. Strauss, *Estimation of Models with Jointly Dependent Qualitative Variables: A Simultaneous Logit Approach.* Econometrica, 1975. **43**(4): p. 745-755.
28. Ghebremichael, M., *Joint modeling of correlated binary outcomes: HIV-1 and HSV-2 co-infection.* Journal of Applied Statistics, 2015. **42**(10): p. 2180-2191.
29. Menzies, S.K., et al., *Risk factors for soil-transmitted helminth infections during the first 3 years of life in the tropics; findings from a birth cohort.* PLoS Negl Trop Dis, 2014. **8**(2): p. e2718.
30. Goldstein, H., *Multilevel Statistical Models*. 4th Edition ed. 2011: JohnWiley and Sons, Ltd, Chichester.
31. PÚBLICA, S.N.D.V.-M.D.S. *Dengue in Ecuador - Esmeraldas*. Available from: https://www.salud.gob.ec/wp-content/uploads/2013/02/DENGUE-GACETA-SE28.pdf.
32. PÚBLICA, S.N.D.V.-M.D.S. *Chikungunya in Ecuador - Esmeraldas*. Available from: https://www.salud.gob.ec/wp-content/uploads/2014/09/GACETA-CHIKUNGUNYA-SE-52.pdf.
33. Leta, S., et al., *Global risk mapping for major diseases transmitted by Aedes aegypti and Aedes albopictus.* International journal of infectious diseases : IJID : official publication of the International Society for Infectious Diseases, 2018. **67**: p. 25-35.
34. Mardekian, S.K. and A.L. Roberts, *Diagnostic Options and Challenges for Dengue and Chikungunya Viruses.* BioMed research international, 2015. **2015**: p. 834371-834371.
35. Dias, J.P., et al., *Seroprevalence of Chikungunya Virus after Its Emergence in Brazil.* Emerging infectious diseases, 2018. **24**(4): p. 617-624.
36. Cunha, R.V., et al., *Seroprevalence of Chikungunya Virus in a Rural Community in Brazil.* PLOS Neglected Tropical Diseases, 2017. **11**(1): p. e0005319.
37. Salje, H., et al., *How social structures, space, and behaviors shape the spread of infectious diseases using chikungunya as a case study.* Proc Natl Acad Sci U S A, 2016. **113**(47): p. 13420-13425.
38. WHO. *Dengue and severe dengue*. Available from: https://www.who.int/news-room/fact-sheets/detail/dengue-and-severe-dengue.
39. WHO. *Chikungunya*. Available from: https://www.who.int/news-room/fact-sheets/detail/chikungunya.
40. Torres, J.R., et al., *Epidemiological Characteristics of Dengue Disease in Latin America and in the Caribbean: A Systematic Review of the Literature.* Journal of tropical medicine, 2017. **2017**: p. 8045435-8045435.
41. Andraud, M., et al., *Dynamic Epidemiological Models for Dengue Transmission: A Systematic Review of Structural Approaches.* PLOS ONE, 2012. **7**(11): p. e49085.
42. Ramos-Castañeda, J., et al., *Dengue in Latin America: Systematic Review of Molecular Epidemiological Trends.* PLoS neglected tropical diseases, 2017. **11**(1): p. e0005224-e0005224.
43. Souza-Neto, J.A., J.R. Powell, and M. Bonizzoni, *Aedes aegypti vector competence studies: A review.* Infection, Genetics and Evolution, 2019. **67**: p. 191-209.

For peer review only - http://bmjopen.bmj.com/site/about/guidelines.xhtml

44. Moraes, G.H., E. de Fátima Duarte, and E.C. Duarte, *Determinants of mortality from severe dengue in Brazil: a population-based case-control study.* The American journal of tropical medicine and hygiene, 2013. **88**(4): p. 670-676.
45. Simmons, C.P., et al., *Dengue.* New England Journal of Medicine, 2012. **366**(15): p. 1423-1432.
46. Priyamvada, L., et al., *Human antibody responses after dengue virus infection are highly cross-reactive to Zika virus.* Proc Natl Acad Sci U S A, 2016. **113**(28): p. 7852-7.

For peer review only

For peer review only - http://bmjopen.bmj.com/site/about/guidelines.xhtml

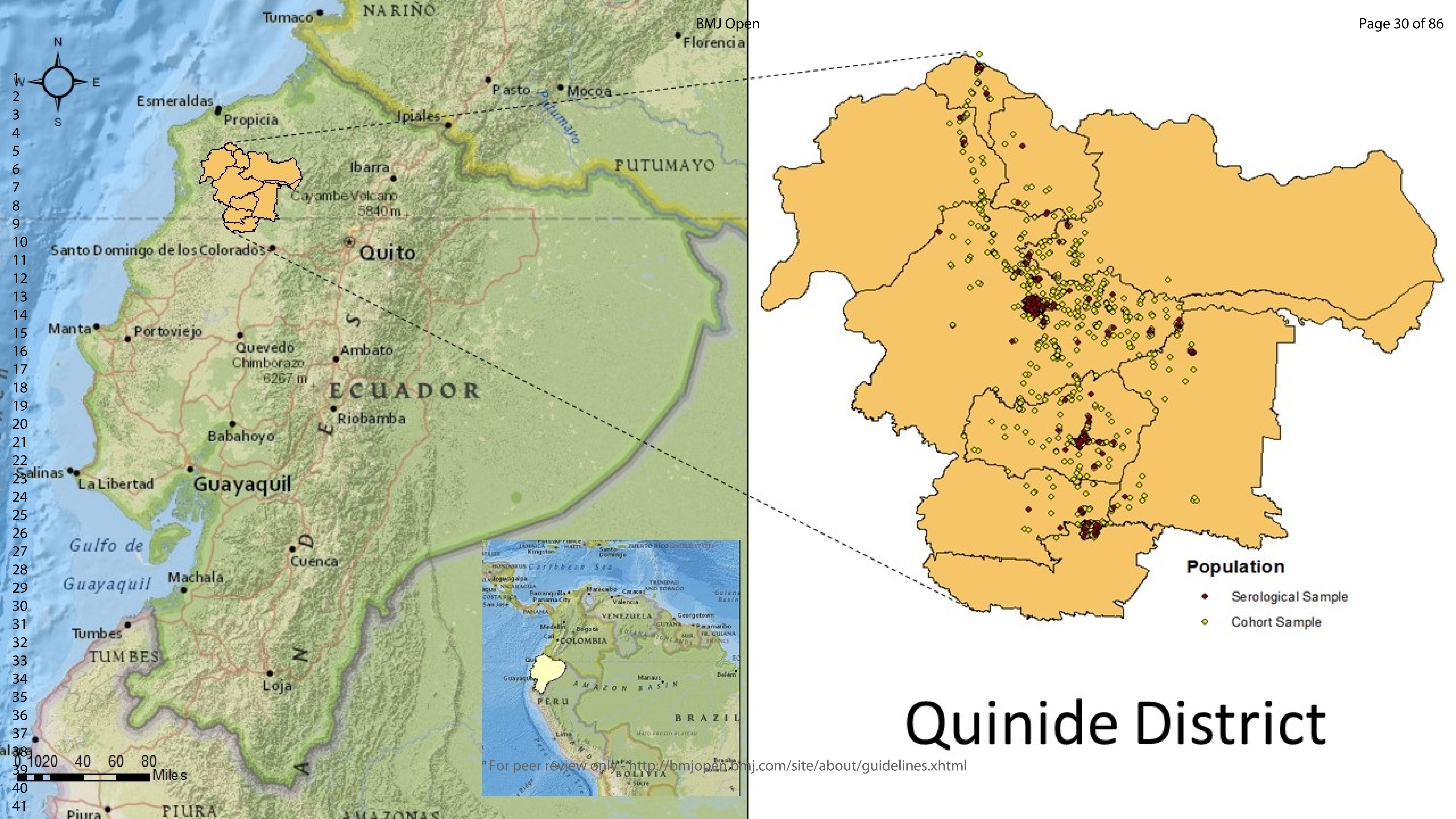
Tumaco
NARIÑO
Florencia
N
W
E
S
Esmeraldas
Propicia
Pasto
Mocoa
Putumayo
Ipiales
PUTUMAYO
Ibarra
Cayambe Volcano
5840 m
Santo Domingo de los Colorados
Quito
Manta
Portoviejo
Quevedo
Chimborazo
6267 m
Ambato
ECUADOR
Riobamba
Babahoyo
Salinas
La Libertad
Guayaquil
Gulfo de
Guayaquil
Cuenca
Machala
Tumbes
TUMBES
Loja
0 1020 40 60 80
Miles
Piura
PIURA
AMAZONAS
COLOMBIA
VENEZUELA
PERU
BRAZIL
Population
Serological Sample
Cohort Sample
Quinide District

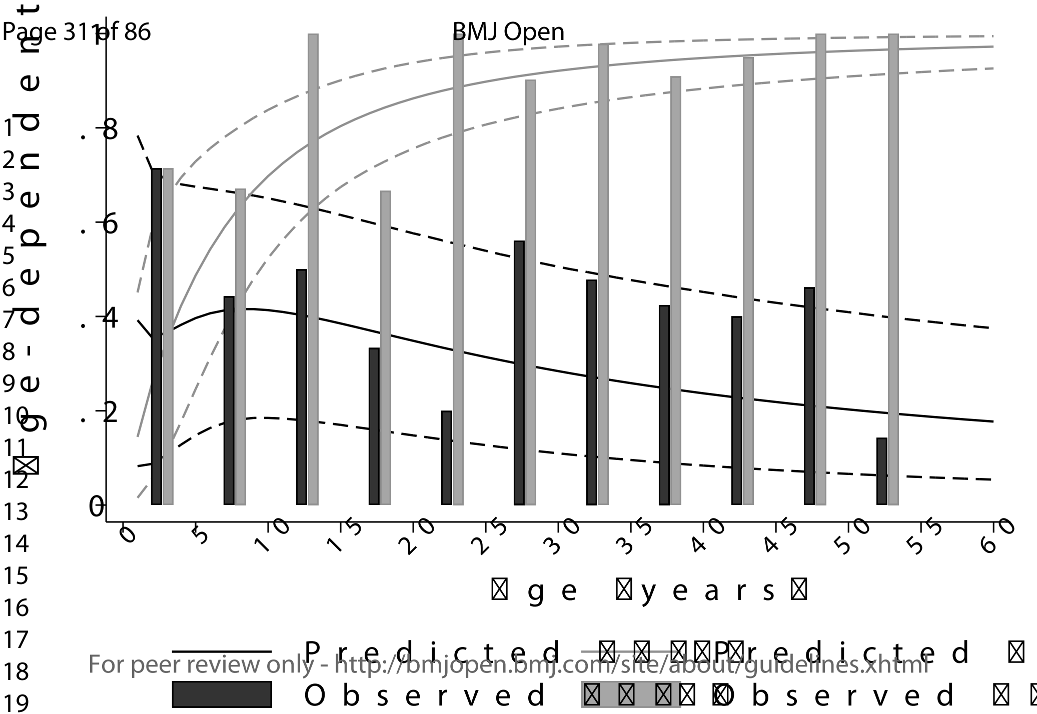

For peer review only - http://bmjopen.bmj.com/site/about/guidelines.xhtml

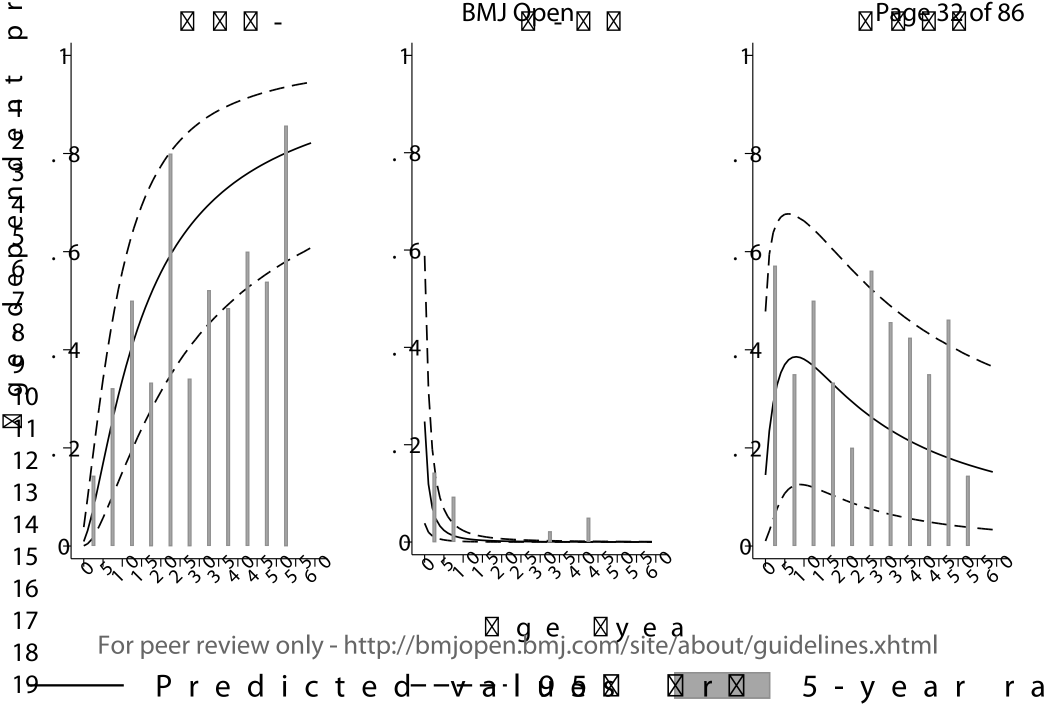
1
.8
.6
.4
.2
0
Predicted value

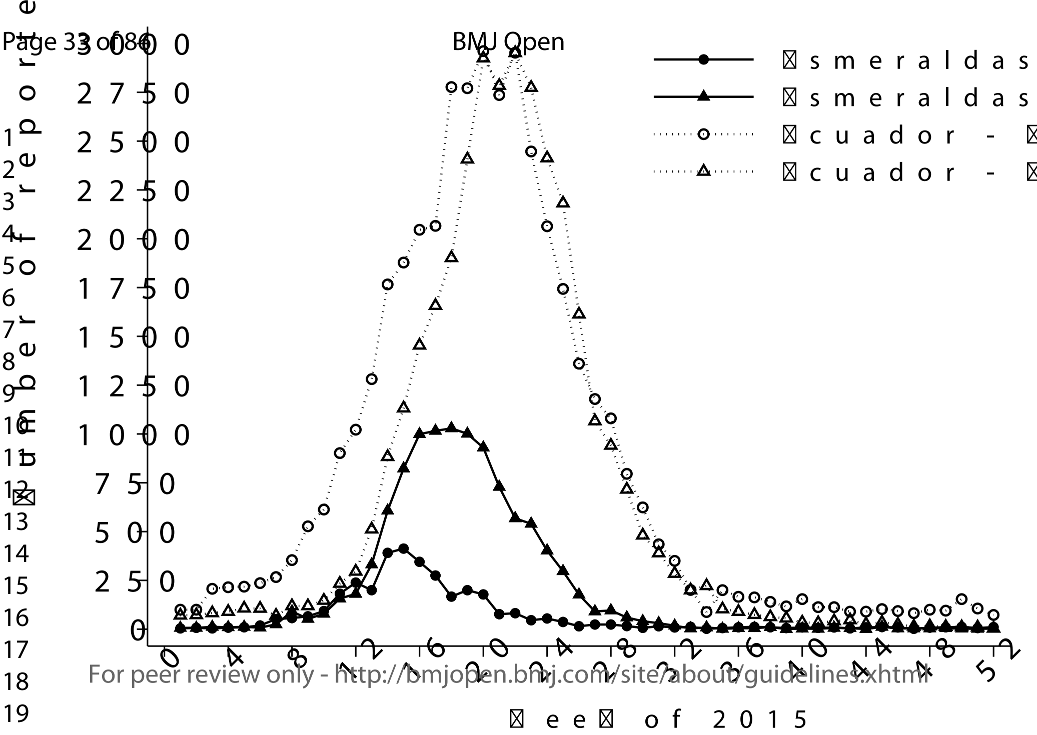

For peer review only - http://bmjopen.bmj.com/site/about/guidelines.xhtml

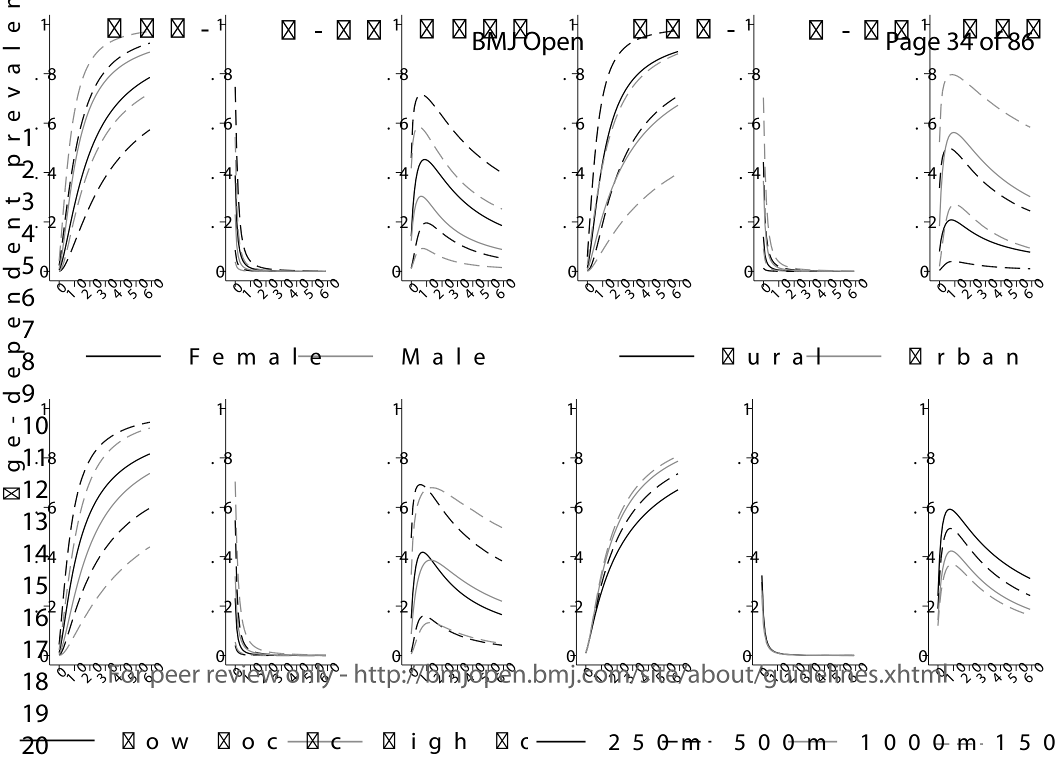

For peer review only - http://bmjopen.bmj.com/site/about/guidelines.xhtml

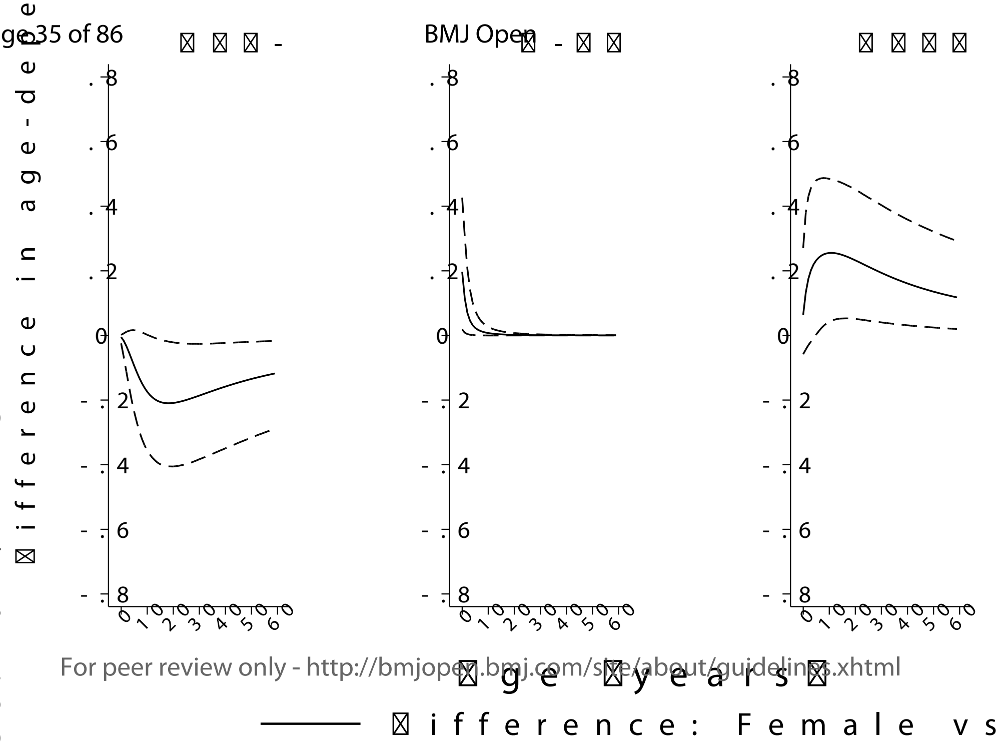
ifference in age-dep
.8
.6
.4
.2
0
-.2
-.4
-.6
-.8
0 10 20 30 40 50 60
ge years
ifference: Female vs

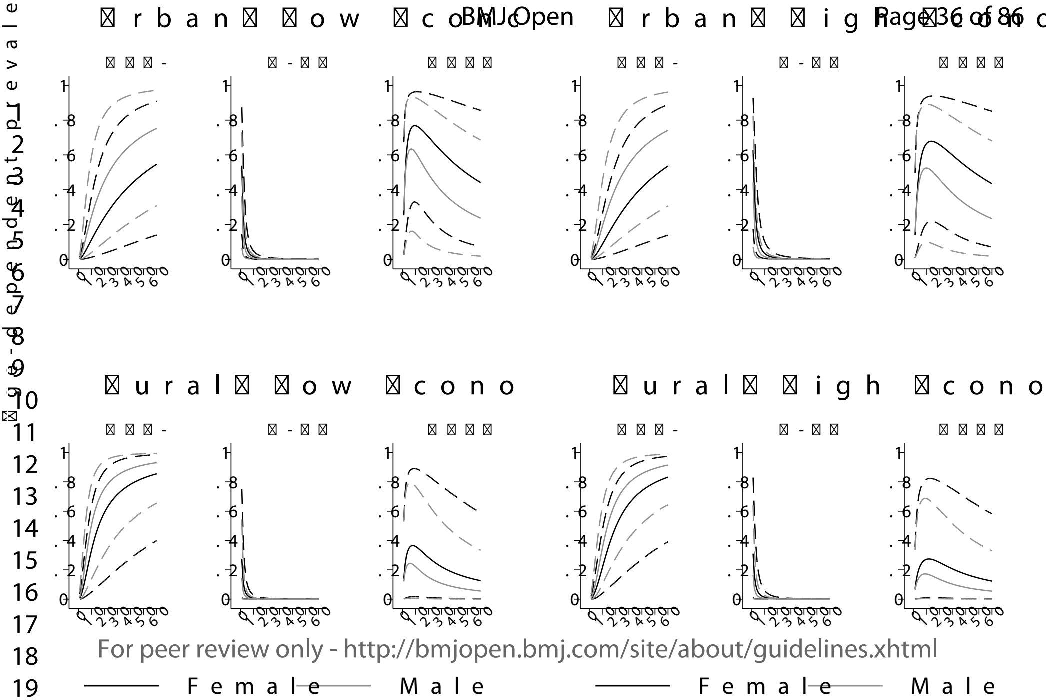

For peer review only - http://bmjopen.bmj.com/site/about/guidelines.xhtml

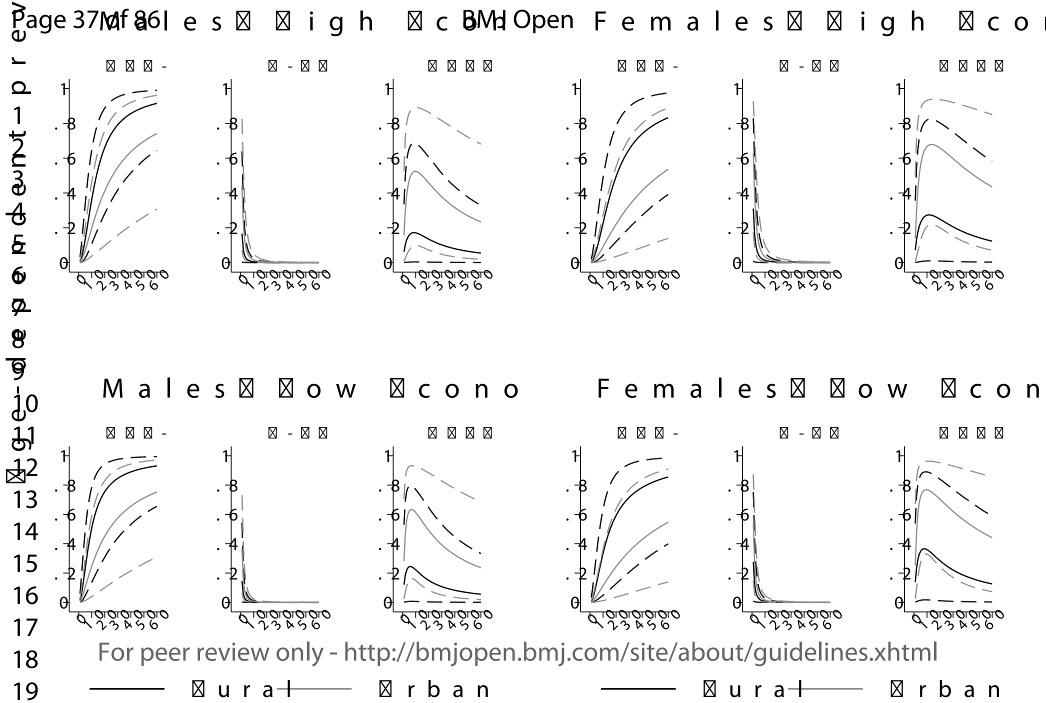

For peer review only - http://bmjopen.bmj.com/site/about/guidelines.xhtml

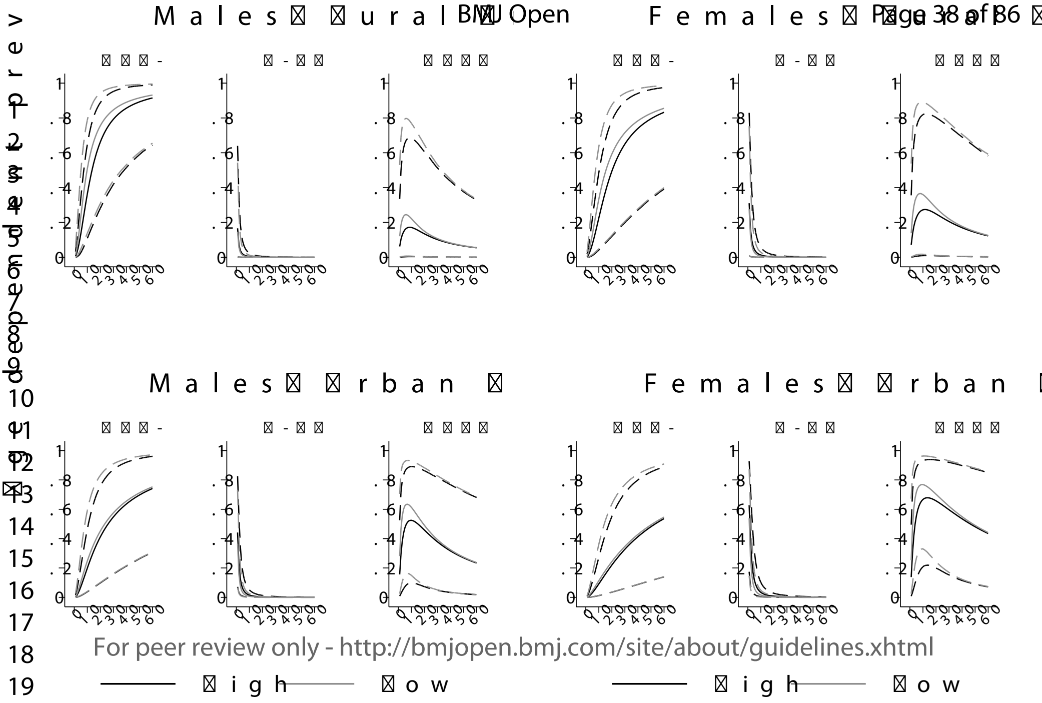

For peer review only - http://bmjopen.bmj.com/site/about/guidelines.xhtml

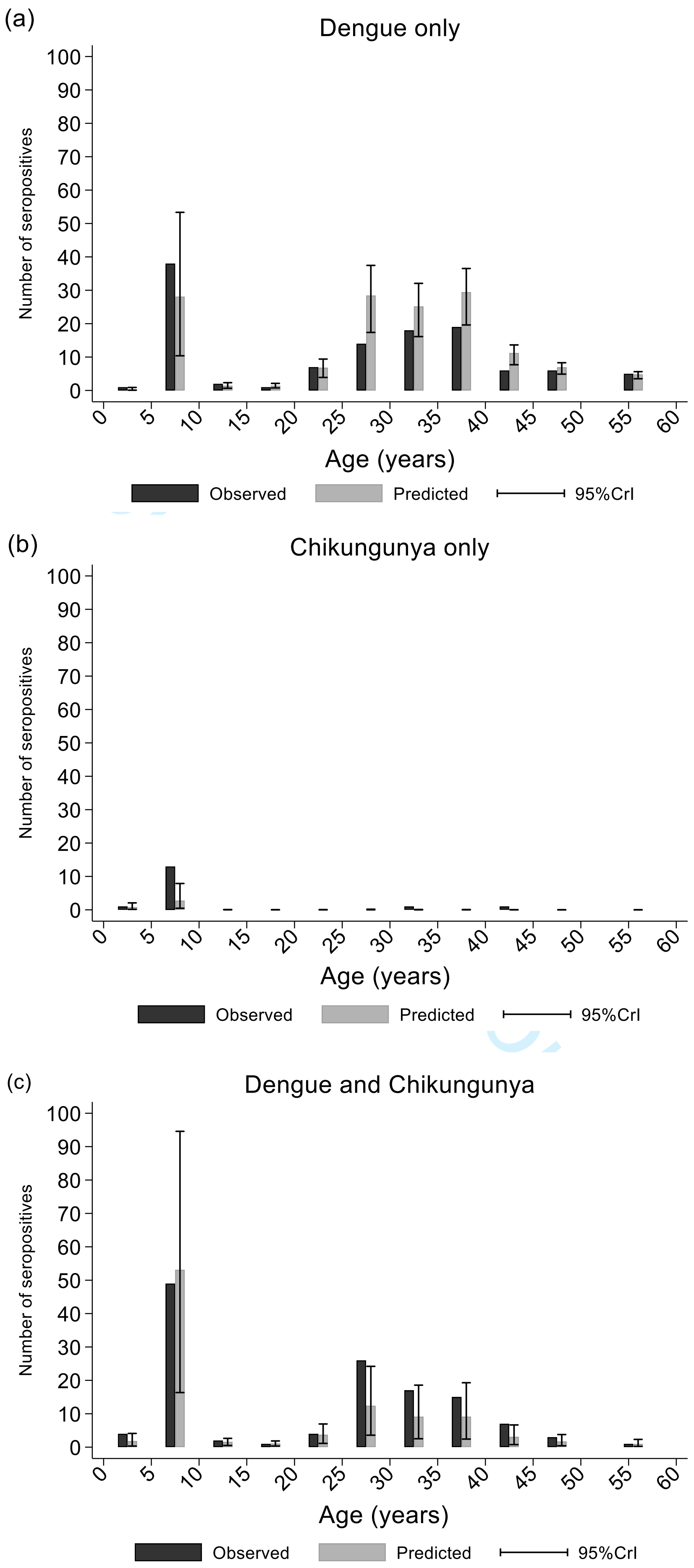
(a)
Dengue only
Number of seropositives
Age (years)
Observed
Predicted
95%CrI
(b)
Chikungunya only
Number of seropositives
Age (years)
Observed
Predicted
95%CrI
(c)
Dengue and Chikungunya
Number of seropositives
Age (years)
Observed
Predicted
95%CrI

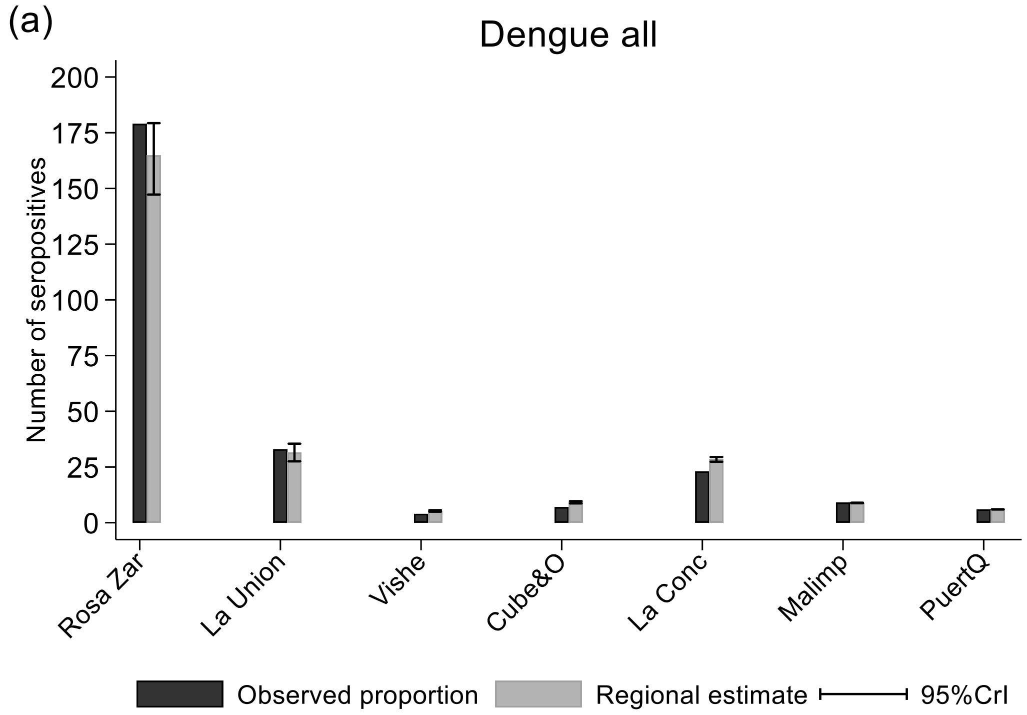
(a)
Dengue all
Number of seropositives
200
175
150
125
100
75
50
25
0
Rosa Zar
La Union
Vishe
Cube&O
La Conc
Malimp
PuertQ
Observed proportion
Regional estimate
95%CrI

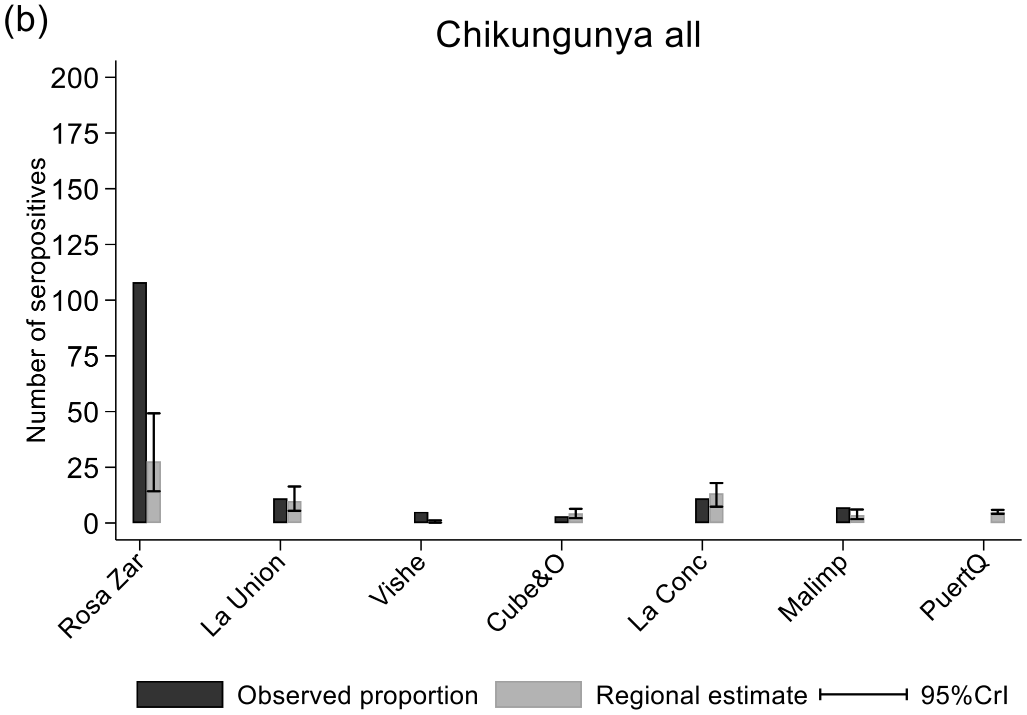
(b)
Chikungunya all
Number of seropositives
200
175
150
125
100
75
50
25
0
Rosa Zar
La Union
Vishe
Cube&O
La Conc
Malimp
PuertQ
Observed proportion
Regional estimate
95%CrI

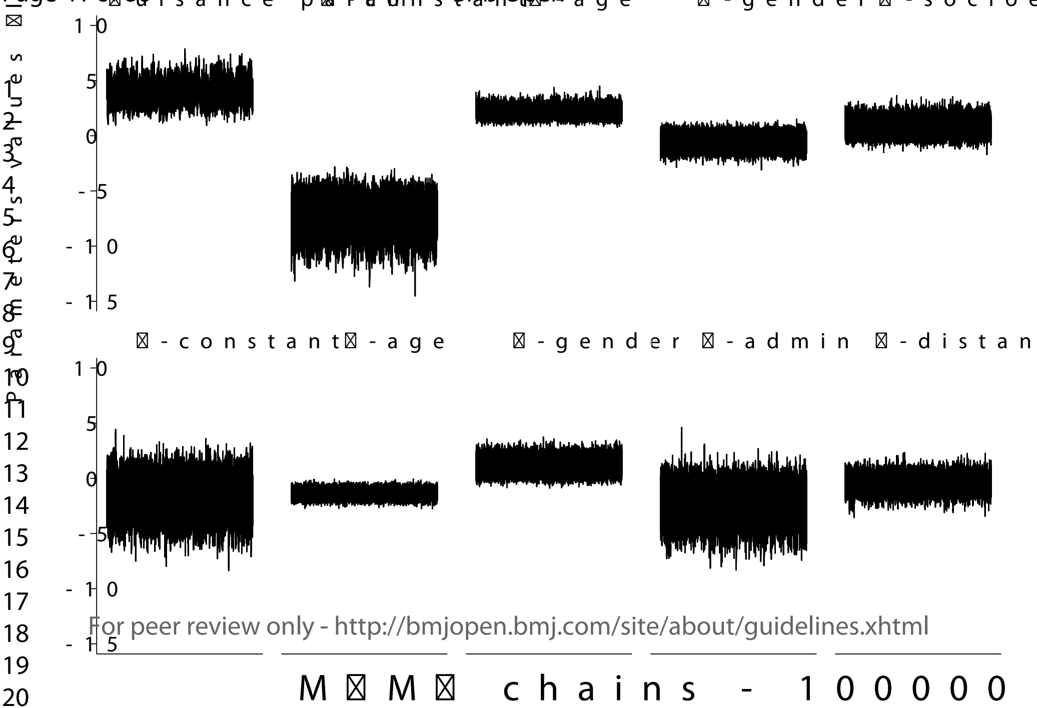

For peer review only - http://bmjopen.bmj.com/site/about/guidelines.xhtml

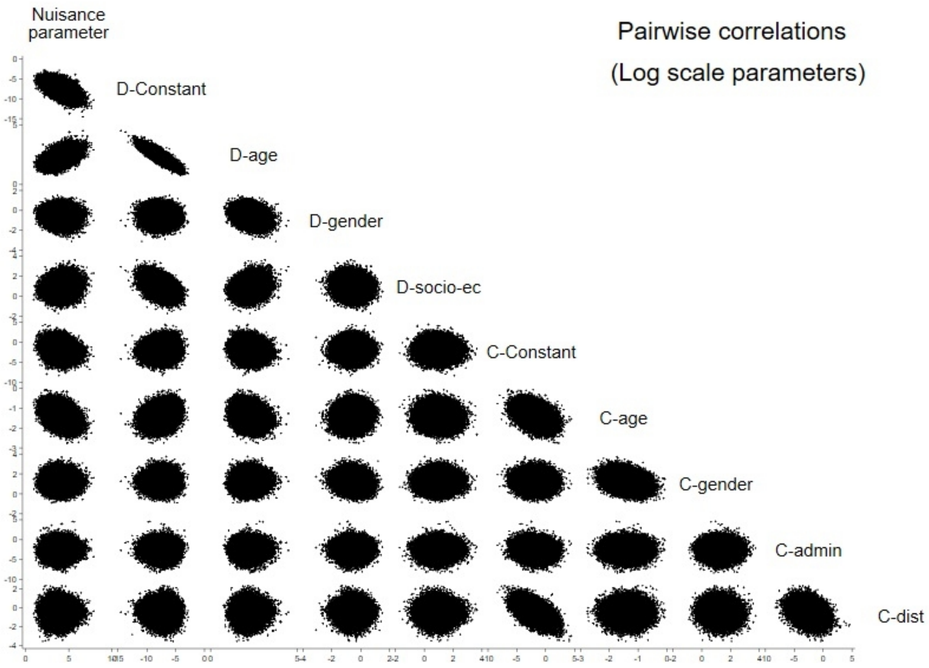

224x162mm (96 x 96 DPI)

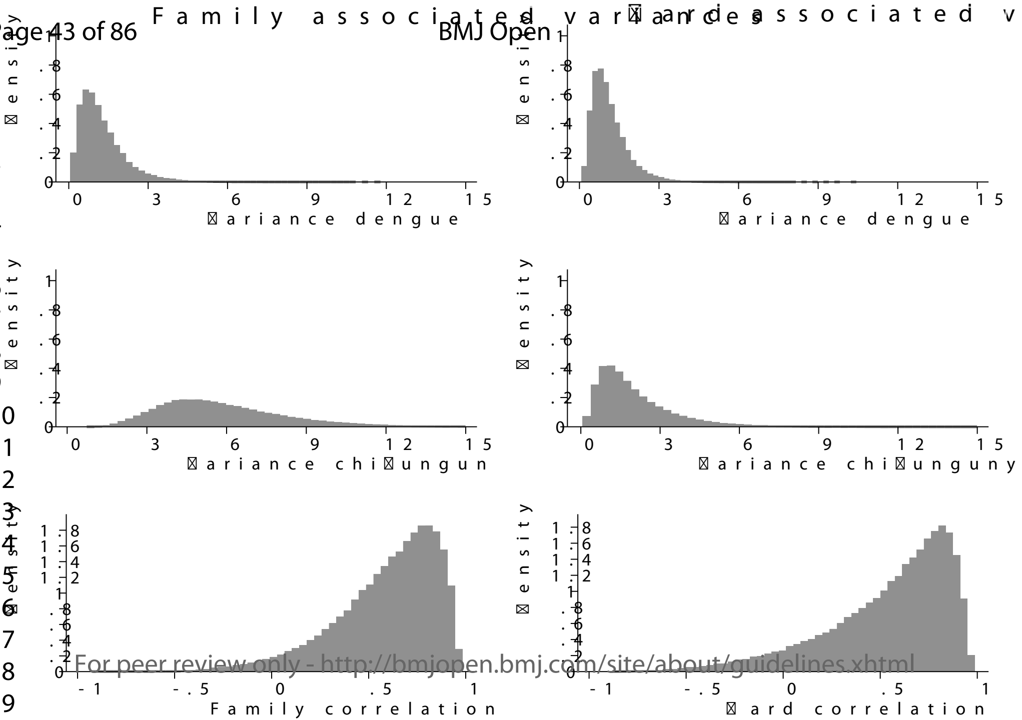

For peer review only - http://bmjopen.bmj.com/site/about/guidelines.xhtml

	Census population		Reported cases of dengue		Reported cases of chikungunya	
	**Ecuador**	**Esmeraldas**	**Ecuador**	**Esmeraldas**	**Ecuador**	**Esmeraldas**
2014	15,944,275	187,880	15, 584 (0.10%)	984 (0.5%)	39 (~0%)	0 (0%)
2015	16,144,368	196,350	42, 499 (0.26%)	3,482 (1.8%)	34,101 (0.2%)	10,477 (5.3%)
2016	16,385,068	204,820	13,612 (0.08%)	1,311 (0.6%)	1, 860 (0.01%)	301 (0.15%)

Supplementary Table 1

For peer review only

Variable	Category	Serological sample 151 households	ECUAVIDA 2404 households	Serological cohort vs. ECUAVIDA
		N (%)	N (%)	One-sample test
Area of residence	Urban	115 (76.2%)	1,796 (75%)	p=0.74
	Rural	36 (23.8%)	608 (25%)	
Socioeconomic Status	High	45 (29.8%)	797 (33.2%)	P=0.46
	Low	106 (70.2%)	1,607(66.8%)	

Supplementary Table 2

For peer review only

Parish	Census pop. Numbers	ECUAVIDA Numbers	Data Numbers	Census pop. Distribution	ECUAVIDA Distribution	Data Distribution	ECUAVIDA Distribution/ Census Distribution	Data Distribution/ ECUAVIDA Distribution	Data Distribution/ Census distribution	WEIGHTS= 1/RATIO
**Rosa Zarate**	16342	1578	102	0.353	0.656	0.675	1.862	1.029	**1.916**	**0.522**
**La Concord**	10662	217	13	0.230	0.090	0.086	0.392	0.954	**0.374**	**2.672**
**PuertoQuito**	5037	27	3	0.109	0.011	0.020	0.103	1.769	**0.183**	**5.469**
**La Unión**	4963	351	21	0.107	0.146	0.139	1.364	0.953	**1.299**	**0.770**
**Cube+Others**	4163	49	6	0.090	0.020	0.040	0.227	1.949	**0.442**	**2.260**
**Malimpia**	4004	81	3	0.086	0.034	0.020	0.390	0.590	**0.230**	**4.348**
**Viche**	1185	101	3	0.026	0.042	0.020	1.644	0.473	**0.777**	**1.287**
**Total**	46356	2404	151	1	1	1				

Supplementary Table 3

For peer review only

Age Group	Age distribution of census population in Esmeraldas		Age distribution of serological sample		Age-adjusted weights=
	Numbers	Proportions	Numbers	Proportions	
0-9	32452	0.211	147	0.461	**0.458**
10-19	33798	0.219	7	0.022	**9.999**
20-29	26507	0.172	46	0.144	**1.193**
30-39	20092	0.130	79	0.248	**0.527**
40-49	16477	0.107	33	0.103	**1.034**
50-59	12279	0.080	4	0.013	**6.357**
60+	12430	0.081	3	0.009	**8.581**

Supplementary Table 4

For peer review only

OUTCOME	CATEG	MEAN	SD	LOWER 95% CrI	MEDIAN	UPPER 95% CrI
ALL						
		MEAN	SD	P025	MEDIAN	P975
D+C-	ALL	0.58	0.09	0.38	0.59	0.74
D-C+		0.01	0.01	0.00	0.01	0.03
D+C+		0.26	0.11	0.08	0.25	0.50
D+		0.84	0.04	0.75	0.84	0.91
C+		0.27	0.11	0.09	0.27	0.52
BY SEX						
D+C-	MALE	0.69	0.08	0.51	0.70	0.81
	FEMALE	0.53	0.10	0.32	0.54	0.72
D-C+	MALE	0.008	0.01	0.001	0.01	0.023
	FEMALE	0.018	0.01	0.004	0.02	0.04
D+C+	MALE	0.13	0.09	0.03	0.12	0.33
	FEMALE	0.32	0.12	0.11	0.31	0.57
SEX DIFFERENCE						
D+C-	F - M	-0.15	0.07	-0.31	-0.15	-0.018
D-C+		0.01	0.007	0.00	0.009	0.03
D+C+		0.18	0.09	0.04	0.18	0.37
BY AREA OF RESIDENCE						
D+C-	URBAN	0.46	0.12	0.23	0.47	0.68
	RURAL	0.68	0.09	0.39	0.70	0.86
D-C+	URBAN	0.017	0.01	0.004	0.015	0.04
	RURAL	0.006	0.005	0.00	0.005	0.02
D+C+	URBAN	0.39	0.14	0.13	0.38	0.68
	RURAL	0.21	0.13	0.03	0.18	0.53
AREA OF RESIDENCE DIFFERENCES						
D+C-	U - R	-0.21	0.16	-0.52	-0.22	0.13
D-C+		0.01	0.01	-0.004	0.01	0.03
D+C+		0.18	0.18	-0.21	0.18	0.53
BY SOCIOECONOMIC STATUS						
D+C-	LOW	0.61	0.11	0.37	0.63	0.79
	HIGH	0.51	0.13	0.24	0.52	0.73
D-C+	LOW	0.009	0.006	0.001	0.007	0.03
	HIGH	0.016	0.01	0.002	0.013	0.04
D+C+	LOW	0.26	0.12	0.07	0.24	0.52
	HIGH	0.28	0.15	0.06	0.26	0.61
SOCIOECONOMIC STATUS DIFFERENCES						
D+C-	L-H	0.10	0.14	-0.18	-0.10	0.39
D-C+		-0.006	0.01	-0.07	0.00	0.008
D+C+		-0.017	0.16	-0.35	0.02	0.29

Supplementary Table 5

For peer review only

Supplementary: The statistical model and inference

The model

This is a model borrowed from econometrics (1) and recently used in the context of infectious diseases which occur simultaneously or are inherently influenced by correlated binary outcomes. We adapted the methodology in the framework of these two binary highly correlated outcomes which can be associated with a similar (or not) set of covariates and the presence of one is highly correlated with the other. A lay description of the approach is presented in the main text. Here, we give details on the mathematical equations and the statistical inference.

Let us denote by X and Y the two binary random variables indicating the presence/absence of an individual to DENV and CHIKV, respectively. This pair of responses define a binary bivariate outcome denoted by (X, Y).

Let us also denote

$R_i = 1 \times \boldsymbol{K}_X$ vector of variables associated with X

$\boldsymbol{\beta} = \boldsymbol{K}_X \times 1$ vector of unknown parameters which quantify the effect of variables associated with X

$Q_i = 1 \times \boldsymbol{K}_Y$ vector of variables associated with Y

$\boldsymbol{\gamma} = \boldsymbol{K}_Y \times 1$ vector of unknown parameters which quantify the effect of variables associated with Y,

$i = \overline{1, 319}$, $j = \overline{1, 151}$, $k = \overline{1,49}$

For peer review only - http://bmjopen.bmj.com/site/about/guidelines.xhtml

NB: We deliberately keep the generality of the model specification although in our scenario $\boldsymbol{K}_X = \boldsymbol{K}_Y$

Then, the two simultaneous logistic equations which depict the nonlinear association with age and the rest of the other variables are written in terms of a system of conditional probabilities on the potential explanatory variables and their random effects:

$$\begin{cases} log\left(\frac{P(X=1|Y)}{P(X=0|Y)}\right)_{ijk} = \beta_{age} \times \log(\text{age}_{ijk}) + \boldsymbol{\beta} \times R_{ijk} + \boldsymbol{\alpha} \times Y_{ijk} + w_{ijk}^{age} + w_k^{ward} + v_{0k}^1 + u_{0jk}^1 \\ log\left(\frac{P(Y=1|X)}{P(Y=0|X)}\right)_{ijk} = \gamma_{age} \times \log(\text{age}_{ijk}) + \boldsymbol{\gamma} \times Q_{ijk} + \boldsymbol{\theta} \times X_{ijk} + w_{ijk}^{age} + w_k^{ward} + v_{0k}^2 + u_{0jk}^2 \end{cases} \quad (1)$$

The triple index (ijk) – denotes individual i in the family j and the ward k

w_{ijk}^{age} denote the age-group constants associated weights (see the subsequent section on Population adjusting weights)

w_k^{ward} denote the ward constants associated weights (see the subsequent section on Population adjusting weights)

$v_{0k}^{1,2}$ denote the random effect associated with the ward

$u_{0jk}^{1,2}$ denote the random effect associated with the family

The latter two sets of random effects are assumed to be drawn from a two normal bivariate normal distributions (MVN) which model the shared and unshared similarities and account for unmeasured aspects such as mosquitos' burden around the households or weather patterns for instance.

$$\begin{pmatrix} v_{0k}^1 \\ v_{0k}^2 \end{pmatrix} \sim MVN\left(\begin{pmatrix} 0 \\ 0 \end{pmatrix}, \sum \begin{pmatrix} (v_0^1)^2 & \rho_v v_0^1 v_0^2 \\ \rho_v v_0^1 v_0^2 & (v_0^2)^2 \end{pmatrix}\right)$$

For peer review only - http://bmjopen.bmj.com/site/about/guidelines.xhtml

$$\begin{pmatrix} u_{0jk}^{1} \\ u_{0jk}^{2} \end{pmatrix} \sim MVN\left(\begin{pmatrix} 0 \\ 0 \end{pmatrix}, \sum\begin{pmatrix} (u_0^1)^2 & \rho_u u_0^1 u_0^2 \\ \rho_u u_0^1 u_0^2 & (u_0^2)^2 \end{pmatrix}\right)$$

On one hand side, from the first simultaneous equation specification (1):

$$\boldsymbol{\alpha} = log\left[\frac{P(X=1|Y=1)}{P(X=0|Y=1)}\right] - log\left[\frac{P(X=1|Y=0)}{P(X=0|Y=0)}\right] = log\frac{P(X=1,Y=1)/P(Y=1)}{P(X=0,Y=1)/P(Y=1)} - log\frac{P(X=1,Y=0)/P(Y=0)}{P(X=0,Y=0)/P(Y=0)} = log\left[\frac{P(X=1,Y=1)\times P(X=0,Y=0)}{P(X=0,Y=1)\times P(X=1,Y=0)}\right] \quad (2)$$

From the second simultaneous equation specification:

$$\boldsymbol{\theta} = log\left[\frac{P(Y=1|X=1)}{P(Y=0|X=1)}\right] - log\left[\frac{P(Y=1|X=0)}{P(Y=0|X=0)}\right] = log\frac{P(X=1,Y=1)/P(X=1)}{P(X=0,Y=1)/P(X=1)} - log\frac{P(X=1,Y=0)/P(X=0)}{P(X=0,Y=0)/P(X=0)} = log\left[\frac{P(X=1,Y=1)\times P(X=0,Y=0)}{P(X=0,Y=1)\times P(X=1,Y=0)}\right] \quad (3)$$

Hence, using the key assumption that $\boldsymbol{\alpha} = \boldsymbol{\theta}$ and keeping the fixed effects only for the sake of simplicity, the system can be written as

$$\begin{cases} log\left(\frac{P(X=1|Y)}{P(X=0|Y)}\right)_i = \boldsymbol{\beta} \times R_i + \boldsymbol{\alpha} \times Y_i \\ log\left(\frac{P(Y=1|X)}{P(Y=0|X)}\right)_i = \boldsymbol{\gamma} \times Q_i + \boldsymbol{\alpha} \times X_i \end{cases}$$

This implies that, if the occurrence of dengue makes more likely (or less likely) the occurrence of chikungunya, the converse is also necessarily true and $\boldsymbol{\alpha}$ is deemed as a nuisance parameter

This crucial assumption ($\boldsymbol{\alpha} = \theta$) and elementary rules of conditioned probabilities leads to the following four statements derived from

For peer review only - http://bmjopen.bmj.com/site/about/guidelines.xhtml

$$\begin{cases} \dfrac{P(X=1|Y=0)}{P(X=0|Y=0)} = \dfrac{P(X=1,Y=0)}{P(X=0,Y=0)} = exp(\boldsymbol{\beta} \times \boldsymbol{R}) \quad (4) \\ \dfrac{P(X=1|Y=1)}{P(X=0|Y=1)} = \dfrac{P(X=1,Y=1)}{P(X=0,Y=1)} = exp(\boldsymbol{\beta} \times \boldsymbol{R} + \alpha) \quad (5) \end{cases}$$

$$\begin{cases} \dfrac{P(Y=1|X=0)}{P(Y=0|X=0)} = \dfrac{P(Y=1,X=0)}{P(Y=0,X=0)} = exp(\boldsymbol{\gamma} \times \boldsymbol{Q}) \quad (6) \\ \dfrac{P(Y=1|X=1)}{P(Y=0|X=1)} = \dfrac{P(Y=1,X=1)}{P(Y=0,X=1)} = exp(\boldsymbol{\gamma} \times \boldsymbol{Q} + \alpha) \quad (7) \end{cases}$$

Hence, combining either (4) + (7) (or (5) + (6)) by multiplication

$$\frac{P(X=1,Y=0)}{P(X=0,Y=0)} \times \frac{P(Y=1,X=1)}{P(Y=0,X=1)} =$$

$$\frac{P(Y=1,X=1)}{P(Y=0,X=0)} = exp(\boldsymbol{\beta} \times \boldsymbol{R}) \times \ exp(\boldsymbol{\gamma} \times \boldsymbol{Q} + \alpha) = exp(\boldsymbol{\beta} \times \boldsymbol{R} + \boldsymbol{\gamma} \times \boldsymbol{Q} + \alpha)$$

But all four probabilities situations ((1, 0), (0, 1), (1, 1), (0,0)) sum to 1:

$$P(X=1,Y=0) + P(X=0,Y=1) + P(Y=0,X=0) + P(Y=1,X=1) = 1$$

$$P(X=0,Y=0) + P(X=0,Y=0) \times exp(\boldsymbol{\gamma} \times Q) + P(Y=0,X=0) \times exp(\boldsymbol{\beta} \times \boldsymbol{R}) + P(Y=0,X=0) \times exp(\boldsymbol{\beta} \times \boldsymbol{R} + \boldsymbol{\gamma} \times \boldsymbol{Q} + \alpha) = 1$$

So

$$P(X=0,Y=0) \times (1 + exp(\boldsymbol{\gamma} \times \boldsymbol{Q}) + exp(\boldsymbol{\beta} \times \boldsymbol{R}) + exp(\boldsymbol{\beta} \times \boldsymbol{R} + \boldsymbol{\gamma} \times \boldsymbol{Q} + \alpha))=1$$

Therefore, the predicted joint probabilities can be derived as it follows:

$$P(X=0,Y=0) = \frac{1}{1 + exp(\boldsymbol{\gamma} \times \boldsymbol{Q}) + exp(\boldsymbol{\beta} \times \boldsymbol{R}) + exp(\boldsymbol{\beta} \times \boldsymbol{R} + \boldsymbol{\gamma} \times \boldsymbol{Q} + \alpha)}$$

$$P(X=0,Y=1) = \frac{exp(\boldsymbol{\gamma} \times \boldsymbol{Q})}{1 + exp(\boldsymbol{\gamma} \times \boldsymbol{Q}) + exp(\boldsymbol{\beta} \times \boldsymbol{R}) + exp(\boldsymbol{\beta} \times \boldsymbol{R} + \boldsymbol{\gamma} \times \boldsymbol{Q} + \alpha)}$$

For peer review only - http://bmjopen.bmj.com/site/about/guidelines.xhtml

$$P(X=1,Y=0)=\frac{exp(\boldsymbol{\beta}\times\boldsymbol{R})}{1+exp(\boldsymbol{\gamma}\times\boldsymbol{Q})+exp(\boldsymbol{\beta}\times\boldsymbol{R})+exp(\boldsymbol{\beta}\times\boldsymbol{R}+\boldsymbol{\gamma}\times\boldsymbol{Q}+\alpha)}$$

$$P(X=1,Y=1)=\frac{exp(\boldsymbol{\beta}\times\boldsymbol{R}+\boldsymbol{\gamma}\times\boldsymbol{Q}+\alpha)}{1+exp(\boldsymbol{\gamma}\times\boldsymbol{Q})+exp(\boldsymbol{\beta}\times\boldsymbol{R})+exp(\boldsymbol{\beta}\times\boldsymbol{R}+\boldsymbol{\gamma}\times\boldsymbol{Q}+\alpha)}$$

Denoting by

$$I_{ij}=\{k|X_k=i,Y_k=j\}\ where\ \ i,j=0,1$$

If the vector of the unknown parameters are denoted by $\phi=(\alpha,\boldsymbol{\beta},\boldsymbol{\gamma})$, the model likelihood can be formally written as

$$L(\phi|Y_k,X_k;\boldsymbol{R},\boldsymbol{Q})=\prod_{i=0,1}\prod_{j=0,1}\prod_{k\in I_{ij}}P(X_k=i,Y_k=j)$$

Bayesian inference consist of summarizing the posterior distributions of the model parameters and requires the joint probability distribution over all random quantities, parameters or observables. The marginal and conditional probability distributions of the vector of unknown parameters are denoted by $Pr(\cdot)$ and $Pr(\cdot|\cdot)$. The ϕ posterior distribution is proportional to the likelihood multiplied by their prior distributions – all assumed non informative as stated in the text. That is

$$Pr(\phi|Y_k,X_k;\boldsymbol{R},\boldsymbol{Q})\propto L(\phi|Y_k,X_k;\boldsymbol{R},\boldsymbol{Q})\times Pr(\phi)$$

Statistical Inference

The simultaneous equations systems (1) define a probability model which has been implemented in OpenBUGS - which makes this approach very appealing even for larger datasets (OpenBUGS program for the simplest model appended). A Bayesian framework for statistical inference using Markov chain Monte Carlo (MCMC) estimation methods naturally accommodates missing

For peer review only - http://bmjopen.bmj.com/site/about/guidelines.xhtml

responses (5%) in the data and allows straightforward assessment of uncertainties in nonlinear models under missing at random assumption. Sensitivities analyses to the missing data (by assuming all positives and all negatives) have been conducted but the results were not altered.

The posterior conditional odds ratios (OR) distributions are presented in Table 2. However, for an easily digestible reading, the summary statistics are presented in terms of means and standard deviations of the predicted joint probabilities by age and stratified by different socio-economic factors that we accounted for.

The MCMC estimation followed 1 million iterations and the corresponding chains (log scale) are displayed in Supplementary Figure 8 – including that of the nuisance parameter. Supplementary Figure 9 display the correlation between the parameters which are all lower than 0.5 in absolute values with the exception of that between the constant and age corresponding to the dengue equations with appear to be highly negatively correlated (-0.87).

Non-informative priors have been used for parameter estimation.

Population adjusting weights used in modelling

We used the 2010 Census data (the latest) adjusted for an average annual growth of in the Ecuador population. All statistical analyses should ultimately serve population predictions. The post-stratification weights, namely calibration, is a technique which corrects the estimation for population misrepresentation which often occurs in ad-hoc surveys or when researchers use already recruited participants. One set of weights are aimed at reducing the effect of Rosa Zarate where most of the data were collected and at inflating the influence of the misrepresented groups (La Union and 10-19 years) on the model likelihood. These weights are associated with each

For peer review only

individual and are additive for the log scale model formulation of the model (see equations above). The other set of weights are aimed at reducing the effect of those in young group and inflate the effects of older people less represented in the sample. The weights are displayed Supplementary Tables 3 and 4. Each observation is associated with a weight according to individual's age group and one by the household location and their additive effect is accounted for in the predictors (log scale so their effect on the actual odds ratios or odds would be multiplicative).

Goodness of fit

The model is developed using a fully parametric paradigm and inherits complexities including random effects for binary outcomes which are not associated with any classical or formal goodness of fit measures.

However, in reference to the basic independent binary outcomes, the only goodness-of-fit measure following a potential *individual based model* fit to these data is that of Hosmer-Lemeshow statistic. That invokes partition of the data *into groups* (https://thestatsgeek.com/2014/02/16/the-hosmer-lemeshow-goodness-of-fit-test-for-logistic-regression/). That is because the individual level raw observations are either 0 or 1 whilst the individual level predicted values can be anywhere between 0 and 1 and hence a one-to-one observed-predicted plot often used for continuous data loses its meaning.

Hence, our approach alludes to that of basic logistic regression and follows similar lines to those developed in Chis Ster, I. Journal of Applied Statistics, 2012. **39**(11): p. 2523-2542, (*Inference for serological surveys investigating past exposures to infections resulting in long-lasting*

For peer review only - http://bmjopen.bmj.com/site/about/guidelines.xhtml

immunity – an approach using finite mixture models with concomitant information) within a related and yet equally complex modelling framework.

We split the data into groups, by 5 years age-groups and geographical sub-regions, and calculated the average predicted probabilities and their 95%CrIs for the outcomes (((D+, C-), (D-, C+), (D+, C+) and also for all dengue and chikungunya) within each group. Then we calculated the expected number of positives corresponding each group and each outcome component by multiplying the corresponding average predicted probabilities with the total number of people within that group. We plotted the observed numbers vs. the predicted numbers in each group.

Given the full parametric, parsimonious nature of this model, and the average sized data – we deemed our model as a good fit to these data.

OpenBUGS code:

```
model{
    #model's likelihood
       for (i in 1:319){
              log_age[i]<-log(age_final_years[i]);
              log_dist[i]<-log(1+min_dist[i]/250);
              weights_final[i]<-weights[i]+weight_age[i];

              dengue[i]~dbern(p[i,1]);
              chick[i] ~dbern(p[i,2]);
              eta[i,1]<-beta1[1]+beta1[2]*log_age[i]+alpha*chick[i]+beta1[3]*gender[i]+
                              beta1[4]*ses_bin[i]+
                              weights_final[i]+
                              theta[family[i],1]+
                              ward_level[ward_index[i],1];

             eta[i,2]<- beta2[1]+beta2[2]*log_age[i]+alpha*dengue[i]+
                              beta2[3]*gender[i]+
                              beta2[4]*urbanrural[i]+beta2[5]*log_dist[i]+
                              weights_final[i]+
                              theta[family[i],2]+
                              ward_level[ward_index[i],2];
               logit(p[i,1])<-eta[i,1];
              logit(p[i,2])<-eta[i,2];
       }
       for (j in 1:151){
              theta[j,1:2] ~ dmnorm(mu.fam[],R_fam[,]);
        }
    for(j in 1:49){
         ward_level[j,1:2] ~ dmnorm(mu.ward[],R_ward[,]);
    }
    for (k in 1:2){

                    mu.fam[k] <-0.0;
                  mu.ward[k] <-0.0;
       }
```


For peer review only - http://bmjopen.bmj.com/site/about/guidelines.xhtml

```
     R_fam[1:2,1:2] ~dwish(Omega_fam[,],2);
     R_ward[1:2,1:2]~dwish(Omega_ward[,],2);
     Omega_fam[1,1]<-1; Omega_fam[1,2]<-0; Omega_fam[2,1]<-0; Omega_fam[2,2]<-1;
  Omega_ward[1,1]<-1; Omega_ward[1,2]<-0; Omega_ward[2,1]<-0; Omega_ward[2,2]<-1;


    Sigma.fam[1:2, 1:2] <-inverse(R_fam[,]);
    Sigma.ward[1:2, 1:2]<-inverse(R_ward[,]);
    for (j in 1:2){
        sigma.fam[j]<-sqrt(Sigma.fam[j,j]);
        sigma.ward[j]<-sqrt(Sigma.ward[j,j]);
        for(k in 1:j){
                rho_fam[j,k]<-0;
                 rho_ward[j,k]<-0;
         }
        for(k in (j+1):2){
                rho_fam[j,k]<-Sigma.fam[j,k]/(sigma.fam[j]*sigma.fam[k]);
                rho_ward[j,k]<-Sigma.ward[j,k]/(sigma.ward[j]*sigma.ward[k]);
        }
    }
    alpha ~ dnorm( 0.0, 0.00001);
    for (j in 1:4){
        beta1[j] ~ dnorm( 0.0, 0.00001);
        beta2[j] ~ dnorm( 0.0, 0.00001);
        exp_beta1[j]<-exp(beta1[j]);
         exp_beta2[j]<-exp(beta2[j]);
         eff1[j] <-step(beta1[j]);
         eff2[j] <-step(beta2[j]);
    }
           exp_alpha<-exp(alpha);
           beta2[5] ~ dnorm( 0.0, 0.00001);
           exp_beta2[5]<-exp(beta2[5]);
}
```

References

1. Schmidt P, Strauss RP. Estimation of Models with Jointly Dependent Qualitative Variables: A Simultaneous Logit Approach. Econometrica. 1975;43(4):745-55.
2. Ghebremichael M. Joint modeling of correlated binary outcomes: HIV-1 and HSV-2 co-infection. Journal of Applied Statistics. 2015 2015/10/03;42(10):2180-91.
3. Goldstein H. Multilevel Statistical Models. 4th Edition ed: JohnWiley and Sons, Ltd, Chichester; 2011.

For peer review only - http://bmjopen.bmj.com/site/about/guidelines.xhtml

We would like to thank both reviewers for their detailed comments on the manuscript – they helped to improve the narrative of the paper. Neither reviewer flagged flaws in the science of the manuscript and complimented it on being "*interesting and diligently conducted*" (Reviewer 1). We believe that the interest shown by the reviewers and their detailed analyses of the manuscript likely will translate into significant interest by the readership of your journal, both by scientists and policy makers.

In response to the reviewers' comments, we have done more analyses to support our findings and modified the text in response to reviewers' comments on our resubmission. May we add that we did not agree with all the comments and have provided robust rebuttals where necessary. We feel that some of the comments were not reasonable and have tried to remedy areas of the text and analyses that we feel have not been understood clearly. We now provide further clarity in the text and further detailed analyses in the supplementary files, particularly in response to the goodness of fit questions.

We would also like to reiterate 3 areas of novelty for your attention and consideration that makes the manuscript of likely interest to readers with different scientific backgrounds:

1. There are limited data on dengue and chikungunya virus infections from Ecuador and other Pacific regions of Latin America apart from aggregated data communicated to international bodies (PAHO/WHO/CDC);
2. There are limited methodologies available to address the issue of co-exposures to co-circulating infections with considerable epidemiological and clinical similarities, a criticism highlighted in recent systematic review based on 147 studies (page 5 in Fritzell, C. et al., *Current challenges and implications for dengue, chikungunya and Zika seroprevalence studies worldwide: A scoping review.* PLoS Neglected Tropical Diseases, 2018. **12**(7))
3. Our manuscript also provides a novel solution (i.e. post-stratification weighting) to another criticism emphasised in the above review relating to imperfect sampling of populations used to draw inferences at population level (page 17: "*potential biases such sampling introduces must be considered during the analysis process"*).

Below we provide point-by-point responses to the reviewer's comments

For peer review only - http://bmjopen.bmj.com/site/about/guidelines.xhtml

Reviewer 1

Reviewer Name: Lorenzo Cattarino

Institution and Country: Imperial College London, UK

Comment 1**:** *The authors examined patterns of age-dependent seroprevalence of dengue and chikungunya in a tropical district of coastal Ecuador and investigated potential determinants of risk of exposure to the two viruses (sex, urban vs rural area of residence, socio-economic level, etc.). They first conducted a serology surveys of 319 individuals in 151 households using IgG ELISA. They then fitted logistic regression models to individual-level seroprevalence data using different sets of explanatory variables, including sex, area of residence, socio-economic level, a measure of crowding and one of potential human dispersal.*

The authors fitted separate models for data samples tested positive to dengue (tested negative for chikungunya), data samples tested positive for chikungunya (tested negative for dengue) and for data samples tested positive to both viruses.
Overall, ***I find the modelling work interesting, diligently conducted, model code is provided which is always good and the manuscript is well written.*** *Seroprevalence studies like this one are an invaluable source of information to better characterize transmission of arboviral diseases. However, I have a couple of comments related to the modelling approach used and study conclusions.*

Response 1: We thank the reviewer for his positive general comments on the manuscript.

Comment 2: *I wonder whether the model fits the data well, particularly the dengue one (Figure 2 in the manuscript). If I read the paper correctly (and I am not quite sure I fully understand the methodology) the authors fitted the model to the raw, individual-level seroprevalence data (i.e. the fitted response variable is the binary outcome of the serology test of each individual, i.e. 1 or 0). They then asses model fit by comparing model predictions to the observed seroprevalence data, aggregated in 5-year age groups.*

A fairer comparison, in my opinion, would be to compare the individual-level predictions with the individual level observations.

No such plot is provided (a plot of predicted individual-level seropositivity by age with overlaid the observed individual-level seropositivity values), although I would be really interested in seeing it. I suspect that such a plot would look quite noisy (due to variation in mosquito population, climatic conditions, endemic nature of dengue epidemics).

Response 2: To clarify, we did *not* fit two separate logistic models to the data – it is just one model using two mixed effects logistic regressions coupled through their random effects and linked by a nuisance parameter to account for co-exposures to the two viruses, their similarities of transmission and clinical manifestations. As mentioned in the manuscript (page 10, second paragraph), there is no formal goodness-of-fit associated with a complex

statistical model that additionally involved mixed effects. If we just take the simplest scenario of a basic logistic regression, the only goodness-of-fit measure following an *individual based model* is that using the Hosmer-Lemeshow statistic. That invokes partition of the data *into groups*. That is because the individual level observations are either 0 or 1 whilst the individual level predictions can be anywhere between 0 and 1 (https://thestatsgeek.com/2014/02/16/the-hosmer-lemeshow-goodness-of-fit-test-for-logistic-regression/). Hence, it is not clear how such a method of goodness-of-fit would, as proposed by the reviewer, might actually work. These one-to-one, observed-predicted comparisons are associated with continuous not with binary data. The goodness-of-fit presented in the manuscript alludes to that of a basic logistic regression (i.e. involving data grouping and calculating the expected number of positives using the average predicted probabilities across groups and, using that and the total number in that group, to calculate the expected number of infections). Our original submission *did* address goodness-of-fit in this manner in supplementary Figure 7 using groups defined by age.

However, to improve clarity we have added now further explanations in both main text and in the supplementary material. Namely, we responded to this reviewer's request in 3 ways:

1. We have added a separate section in which we have explained in detail the goodness of fit methodology as below and which is now on page 7 of the Supplementary Material
2. We have altered Figure 2 as this reviewer requested by also age-group observed bars, also displayed below for your perusal
3. We have added observed-predicted plots across geographical regions (Supplementary Figure 8) and for numbers of all dengue and all chikungunya seropositive participants - also appended below.

1 Goodness of fit

The model is developed using a fully parametric paradigm and inherits complexities including random effects for binary outcomes which are not associated with any classical or formal goodness of fit measures.

However, in reference to the univariate independent binary outcomes, the only goodness-of-fit measure following a potential *individual based model* fit to these data is that of Hosmer-Lemeshow statistic. That invokes partition of the data *into groups* (https://thestatsgeek.com/2014/02/16/the-hosmer-lemeshow-goodness-of-fit-test-for-logistic-regression/). That is because the individual-level raw observations are either 0 or 1 whilst the individual level predicted values can be anywhere between 0 and 1 and hence a one-to-one observed-predicted plot often used for continuous data loses its meaning. Hence, our approach alludes to that of basic logistic regression and follows similar lines to those developed in Chis Ster, I. Journal of Applied Statistics, 2012. **39**(11): p. 2523-2542, (*Inference for serological surveys investigating past exposures to infections resulting in long-lasting immunity – an approach using finite mixture models with concomitant information)* within a related and equally complex modelling framework.

For peer review only - http://bmjopen.bmj.com/site/about/guidelines.xhtml

We split the data into groups, by 5 years age-groups and geographical sub-regions, and calculated the average predicted probabilities and their 95% CrIs for the outcomes ((D+, C-), (D-, C+), (D+, C+) and also for all dengue and chikungunya) within each group. Then we calculated the expected number of positives corresponding each group and each outcome component by multiplying the corresponding average predicted probabilities with the total number of people within that group. We plotted the observed numbers vs. the predicted numbers in each group.

Given the full parametric, parsimonious nature of this model, and the average sized data – we deemed our model as a good fit to these data.

2 The new Figure 2 as requested by the Reviewer has been redone as below:

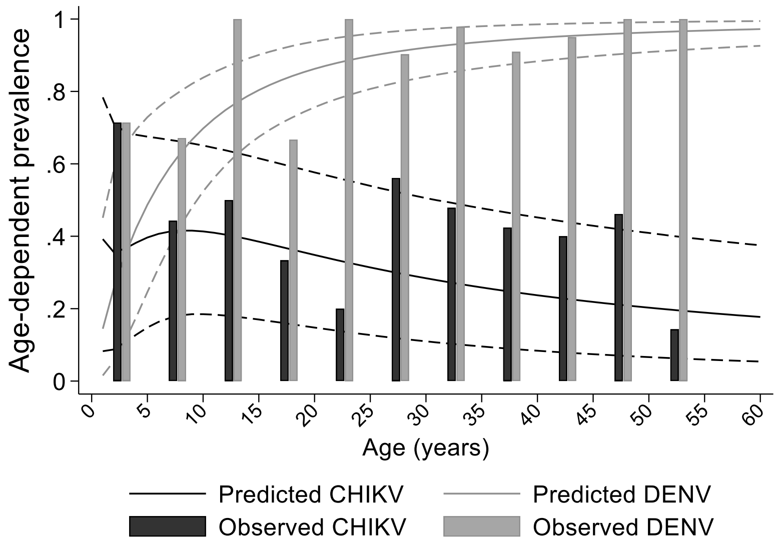

3 We also added new Figures regarding the spatial goodness of fit as requested by the reviewer. The represent the observed vs. expected numbers of all dengue and all chikungunya by geographical sub-region and reflect a reasonable goodness of fit of the model to the data.

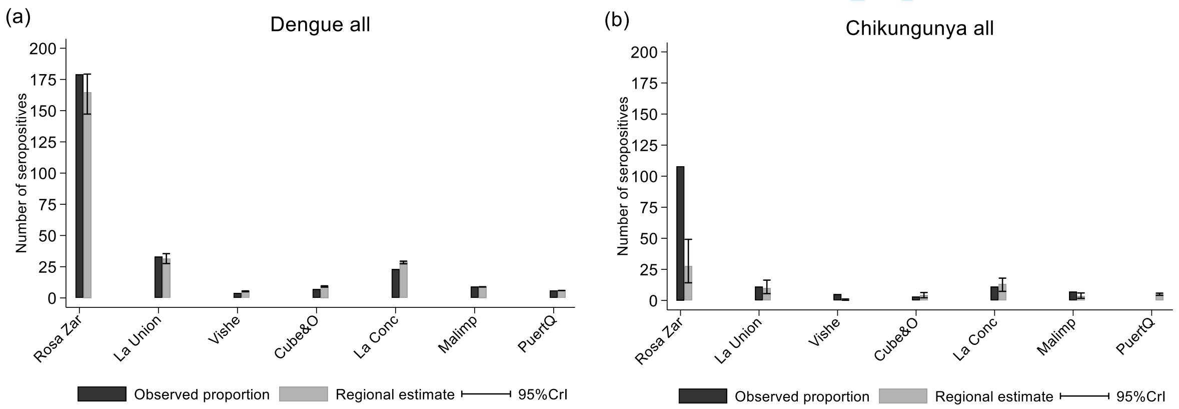

For peer review only - http://bmjopen.bmj.com/site/about/guidelines.xhtml

<u>Comment 3</u>: *An alternative to the individual-level fit presented here is to stratify the response by 5-year age group, calculate the proportion of seropositive individuals in each age group, and fit a catalytic model of force of infection to age-stratified seroprevalence data (not individual-level as currently done by the authors). This approach might help smoothing out variations in seroprevalence with age and possibly improving model fit. The approach I am referring to is explained in detail in several papers, including:*

N. Imai, I. Dorigatti, S. Cauchemez, N. M. Ferguson, Estimating Dengue Transmission Intensity from Sero-Prevalence Surveys in Multiple Countries. PLoS neglected tropical diseases 9, e0003719 (2015).

I. Rodriguez-Barraquer, H. Salje, D. Cummings, Opportunities for improved surveillance and control of infectious diseases from age-specific case data: insight from dengue. eLife 8, e45474 (2019).

<u>Response 3</u>: We thank the reviewer for these suggestions. There are, of course, many interesting questions to address regarding dengue and chikungunya and that includes its transmission dynamics which is a different body of work which would make a piece of research on its own. We feel that the question addressed here - which was inspired by the availability of individual detailed data - regarding age-dependent prevalence of past exposure to these co-circulating and related viruses is equally important for the local policy makers and a wide range of scientists. That is another criticism highlighted in the discussion of the December 2018 comprehensive review cited above (Fritzell, C. et al., *Current challenges and implications for dengue, chikungunya and Zika seroprevalence studies worldwide: A scoping review*. PLoS Neglected Tropical Diseases, 2018. **12**(7))

There is certainly value in fitting models to aggregated data - *in the absence of individual level data* and if the grouping correctly reflects the structure of the population to which the inference is made.

However, we feel that in our data set, besides having to ignore valuable individual information (such as further refinement by socio-demographic variables), this strategy will *not* allow interpretation of the results at the individual level (we refer to the *ecological fallacy* concept when inference from aggregated data is wrongly used to draw conclusions at individual level). In our approach, each individual is assigned a population-specific weight according to the age and to the location of their household. Our method uses population level demographics to address the issue of inference at population level in the context of field data collection.

For these reasons, we do not believe that a fit to aggregated data is a preferable approach answers our question or offers a better solution to the goodness of fit. In a previous scientific paper published in an applied statistical journal (Chis Ster, I., *Inference for serological surveys investigating past exposures to infections resulting in long-lasting immunity – an approach using finite mixture models with concomitant information*. Journal of Applied

For peer review only - http://bmjopen.bmj.com/site/about/guidelines.xhtml

Statistics, 2012. 39(11): p. 2523-2542), we used a similar goodness-of-fit approach to address a similar question – our approach was found to be acceptable by the journal after review by three professional statisticians.

One of the papers to which the reviewer refers was included in our original submission (Reference #24, now#18). This and the other paper to which the reviewer refers (now included in our resubmission as Reference #19), addressed the question of global transmission of dengue using available *aggregated not individual* data, highlighted a high level of heterogeneity and *calling for local data collection and analyses*. The 2nd paper (Rodriguez-Barraquer et al) concluded that ***"… the picture provided by serology is very incomplete and limited when trying to characterize transmission across time and space both within and between countries*** ”an assertion with which we fully agree. Moreover, we believe that serological data suits better our research question which not related to infection transmission/dynamics but that of age-dependent levels of past exposure in the study population. We also agree with these authors' statement regarding high spatial heterogeneity of dengue transmission. Clearly, climate and geography play crucial roles in the dengue life-cycle and the original supplementary material stated the ward level random effects are meant to capture these unmeasured features.

Our inference, in this study, is confined to a population from coastal Ecuador and can only be generalized to a country level after considering several important limitations that are discussed in the submission. Finally, our manuscript addresses a different question to that of arbovirus transmission – there are no local data on epidemiology of *Aedes* spp. from our study area – but rather we have chosen to focus our research question on a relatively rich and unique source of data at individual level from a birth cohort to understand age-dependent seroprevalence for co-exposures to DENV and CHICKV and their determinants at individual and household levels.

Comment 4: *It would be interesting to see if such a model fits better, or not, the data. Regardless, I think that model goodness of fit should be evaluated between predictions and the actual disaggregated observations used to fit the model.*

Response 4: Please see our Responses to Comments 2 and 3 above, and also our general responses to the reviewer below for further clarification. We feel that there is some confusion in the Reviewers' requests with respect to goodness-of-fit: on one hand the reviewer advocates the use of aggregated data methods to address a different question which are clearly not an optimal approach for our individual level data (which serves to understand patterns in our populations and which the reviewer stated was of interest) and, on the other hand, asks for goodness of fit methods tailored to disaggregated data. We have provided population aggregated data in Table 1. Also, the frequencies of the serological cases can be seen in the goodness of fit Supplementary Figures 8 and 9 where they are plotted by age group and by geographical sub-region.

Comment 5: *I am struggling to appreciate why it is important to fit a combined model (dengue + /chikungunya +). The dengue and chikungunya viruses are known to potentially cross-react and it might be difficult to make useful conclusions from the combined model.*

For peer review only - http://bmjopen.bmj.com/site/about/guidelines.xhtml

Maybe the authors can expand on the rationale for this in the introduction and explain a bit more the limitations of the approach in the discussion. Related to the used of the combined model and to the broader research question, I think the justification for looking at cocirculation of the two arboviruses could be clarified and strengthened in the introduction.

Response 5: There can be issues of cross reactivity due to cross-reactivity of the distinct serum-complexes to which dengue (DENV) and chikungunya (CHIKV) viruses belong. However, for the IgG ELISA assays used in this study this is not considered to be a problem. The DENV assays used purified DENV-2 derived protein as coating which is sufficiently cross-reactive across the 4 principal DENV serotypes as to be useful for measurement of exposure to these serotypes, and which may also cause significant antibody cross-reactivity to other flaviviruses such as Zika virus but not to alphaviruses such as CHIKV (Ohst et al, Adv Exp Med Biol 2018: 1062:19-43). The antigen used for coating CHIKV plates was the recombinant envelope protein 2 of CHIKV which is highly specific for the detection of this virus (Kam et al. PLoS Negl Trop Dis 2015; 9: e3445; Fumagalli et al Virol J 2018; 15: 112). Further there was no evidence of a correlation between OD (optical density) values for IgG responses to DENV and CHIKV (r=-0.0201), illustrated by the quite distinct age-dependent seroprevalence curves for DENV and CHIKV (Figure 2). Further a random sample of 92 plasma samples from the same cohort taken in 2013 (i.e. before the appearance of CHIKV in Ecuador) showed no positive reactions using the same commercial assay, indicating sufficient specificity in reference to DENV. Our data clearly show the differing epidemiology of the two viruses in this population - the endemic nature of DENV as opposed to the ‘stochastic’ age-seroprevalence profile for CHICKV – justifying the approach to address co-exposures and their epidemiological determinants. This approach allowed us to make observations specific to each virus that, although they share the same vector, differ with respect to numbers of human infections caused year-on-year depending on unknown factors such as vector competence, viral interference within vectors, and the density of susceptible humans.

Comment 6: *Previous studies have looked at estimating seroprevalence by age in settings with co-circulating arboviruses (e.g. I. Rodriguez-Barraquer, S. S. Solomon, P. Kuganantham, A. K. Srikrishnan, C. K. Vasudevan, S. H. Iqbal, P. Balakrishnan, S. Solomon, S. H. Mehta, D. A. Cummings, The Hidden Burden of Dengue and Chikungunya in Chennai, India. PLoS neglected tropical diseases 9, e0003906 (2015).).*

What is the novel research question this paper is addressing, relative to previous studies? This could be clarified in the manuscript.

Response 6: We thank the reviewer for highlighting this interesting reference which we overlooked in our previous submission. Of course, the study to which the reviewer refers was done in a different population in a different geographic region (Chennai, India) with different infection dynamics. This paper by Rodriguez-Barraquer et al., addressed different study questions (i.e. key infection dynamics parameters such as force of infection and R_0) using serological data. The authors use survey data to fit two *separate* models to the two outcomes (DENV and CHIKV) where there was a clear underlying correlation between the two outcomes. The use of the novel analytic approach described in our manuscript offers clear

advantages over such an approach. However, this study has now been included (Reference 22) in our resubmitted manuscript.

<u>Comment 7</u>: *The results regarding the determinates of risk of past exposure could be given more attention in the paper main text, as I find them quite interesting. Perhaps the authors can add a summary table with odd ratios for each risk factor (e.g. I. Rodriguez-Barraquer, S. S. Solomon, P. Kuganantham, A. K. Srikrishnan, C. K. Vasudevan, S. H. Iqbal, P. Balakrishnan, S. Solomon, S. H. Mehta, D. A. Cummings, The Hidden Burden of Dengue and Chikungunya in Chennai, India. PLoS neglected tropical diseases 9, e0003906 (2015).)*

<u>Response 7:</u> The information which the reviewer requests was provided in Table 2 of the original submission. We thought that these conditional ORs might be difficult to conceptualize for the non-specialist reader and, in addition, provided predicted prevalence across the groups in the population in the supplementary and use that interpretation of the results for the general reader.

<u>Comment 8</u>: *It would also be interesting to see how the predicted seroprevalence varies spatially in the study region. Dengue transmission is highly spatially heterogenous and a map of predicted seroprevalence might give some idea of the sources of this heterogeneity. Please see for example the Rodriguez-Barraquer papers above cited.*

<u>Response 8</u>: We thank the reviewer for this suggestion. We have we added region associated numbers and their corresponding predictions in the last figure associated with the *<u>Response 2</u>* to the *<u>Comment 2</u>* above. as mentioned above. However, the Rodriguez-Barraquer study was done on a much larger geographic area with refined predictions possible given large numbers. We do not believe that such an approach would be appropriate in our own given the much low numbers per spatial location. However, we have added relevant figures corresponding to goodness-of-fit as described in detail above.

<u>Comment 9</u>: *It would be also useful to highlight more the implications of the study for development of control strategies against dengue and chikungunya. How can we use the results of this study (predicted seroprevalence by age) to inform how effective alternative control strategies can be? It might be difficult, due to the observed variation in seroprevalence with age, which is also picked up by the model used by the authors. One advantage of fitting age-stratified data is that it allows to calculate the force of infection (per capita rate at which susceptible individual acquire infection). Force of infection can be used to infer the reproduction number* ***<u>R0 (average number of infections caused by one infectious individual)</u>****, which can be used to predict the potential impact of control strategies, such as vaccination (recently addressed in L. Cattarino, I. Rodriguez-Barraquer, N. Imai, D. A. T. Cummings, N. M. Ferguson, Mapping global variation in dengue transmission intensity. Sci. Transl. Med. 12, (2020).)*

For peer review only - http://bmjopen.bmj.com/site/about/guidelines.xhtml

Response 9: These comments regarding fitting a model to aggregated age-stratified data have been addressed in our previous responses - we reiterate our aim at making full use of our valuable individual-level information.

We agree that the key epidemiological measures to understand infection dynamics is the force of infection – all other measures including transmission parameters are derived from it. We are also aware about the definition and relevance of the R_0 as key dynamic descriptor of an epidemic. However, **dengue is not transmitted between people this is a well-known vector-transmitted disease and definition of R_0 is** ***incorrect*** (Estimating R_0 in this context of vector borne transmission involves extra complexities requiring data that are unavailable in our setting such as information on mosquito density and competence in the population).

Summarising: the question of disease dynamics is just different from our study question and we feel that the request to conduct a different body of work which is enough to generate a separate research communication on its own, is unreasonable.

We did add this new reference (Ref no 22 in the current version) stating in the discussion that "*Highlighting areas of high prevalence is important as recent analyses suggested that transmission-blocking interventions such as Wolbachia, even at intermediate efficacy (50% transmission reduction), might reduce global annual disease incidence by up to 90%. The newly licensed Sanofi-Pasteur vaccine, targeting only seropositive recipients, (namely those at risk of secondary infection which can result in severe dengue and subsequent death), might reduce global annual disease incidence by 20 to 30%, with the greatest impact in high-transmission settings*"

Additional comments in response to Reviewer 1:

The request of fitting a model to aggregated data (i.e. data grouped by age) is somewhat at odds with seeing a goodness-of-fit by means of a "*plot of predicted individual-level seropositivity by age with overlaid the observed individual-level seropositivity values*". Individual-level inferences made using models fitted to aggregated data can be deeply flawed as recognised by the *ecological fallacy*.

Given our individual-level information in the data, we feel that an aggregated modelling approach would waste valuable information that allows us to draw inferences.

As discussed in the previous research mentioned by this reviewer (Rodriguez-Barraquer et. al. above), serological data might be the best answer to the question of disease dynamics in this context of vector transmitted virus but we believe that it is very well suited to answer the question of prevalence of past exposure in the population given the long-lasting immunity

For peer review only - http://bmjopen.bmj.com/site/about/guidelines.xhtml

these viruses following exposure. The method developed by our paper is robust, account for the potential effect of unmeasured variables and embeds population-adjusted weighting which allow generalization of the results to the region of coastal Ecuador.

Further, such "*plot of predicted individual-level seropositivity by age with overlaid the observed individual-level seropositivity values*" would not allow us to achieve evidence of goodness of fit as the outcomes are binary with predicted probabilities between 0 and 1. We have clarified this issue by starting with the simplest case of a logistic regression where the Hosmer-Lemeshow measure of goodness of fit involves grouping the data and calculating the expected numbers of positive individuals across groups – however, the classical method applied to independent data no longer holds for hierarchical data. We had addressed goodness of fit in a Hosmer-Lemeshow manner in our original submission by plotting raw numbers for each type of response (D+C-, D-C+, D+C+) against expected numbers by age group, which might, perhaps, have been overlooked by the reviewer. We have added in this resubmission, a section dedicated to goodness of fit in supplementary files with further clarifications, and have more added also observed vs. predicted plots including by geographical region within the study district. We show that our model provides a good fit to the data considering the inherent limitations of a fully parametric parsimonious model fitted to our data set. We have added also group bars using raw data in Figure 2 as requested by the reviewer.

Reviewer 2 generic comments:
Reviewer Name: M. Celeste Torres
Institution and Country: Oswaldo Cruz Institute - FIOCRUZ, Brazil
Please state any competing interests or state 'None declared': None declared

Comment 1: The majority of my comments are included in the attached file. But there are particular main points that I feel are necessary to reinforce. English needs to be improved. Through this, the manuscript comprehension would be significantly facilitated. Spelling must be checked as well.

The objectives and hypotheses are not clearly defined. The methodology is not entirely clear, nor the study population correctly described. The methods and results are very mixed. It needs to be restructured.

For peer review only - http://bmjopen.bmj.com/site/about/guidelines.xhtml

<u>Response 1</u>: We have edited the English in the manuscript to improve clarity as requested and corrected some typos present in the previous version. We also provided a clear description of the hypothesis underlying this analysis in the final paragraph of the Introduction which reads now:

"In this study, we hypothesised that prevalence of past exposure to DENV and CHIKV in the population are associated with age, and individual and household risk factors. We estimated age-dependent seroprevalence of DENV, CHIKV and co-exposures with both arboviruses in a district in coastal Ecuador, and explored individual and household determinants of past exposure status."

A deliberate decision was made to separate the somewhat complex statistical modelling into supplementary files while maintaining the epidemiological question relating to co-exposures to the two arboviruses in the 'core' manuscript. In this way, the non-specialist reader can follow the basic analytic methodology and key results and refer if necessary to supplementary files. The novel but somewhat complex statistical methodology provided in the supplementary files are more likely to be of interest to advanced statistical modellers who may wish to use the methods described in their own research as well as using the code. We have addressed all the reviewer's comments with corresponding changes in a tracked revision of the modified manuscript.

<u>Comment 2</u>: *About the serological tests, I am very concerned about the level of confidence that can be achieved, particularly for dengue. As I commented in the manuscript, cross-reaction for flaviviruses is well known and a big issue for these techniques when it comes to diagnosis, for example. There is a commercial kit that crosses even between DENV and CHIKV. At anyway, ZiKV and YFV can be influential for DENV indirect serological tests. Besides, in low-prevalent scenarios (as can be for CHIKV in 2016), the positive predictive value of the test may decrease, leading to a less confident result. In this regard, and considering as well that serological results would be introduced into the modelling, shouldn't the authors be 100% sure that results obtained are true positives?*

<u>Response 2</u>. We agree that there is likely to be at least one (of many commercial kits available) for which cross-reactivity between DENV and CHIKV is an issue. Please also see Response 5 to Reviewer 1 above. The commercial assays we used have been validated and show minimal cross-reactivity between flaviviruses and alphaviruses. Particularly for DENV there may be issues of cross-reactivity with other flaviviruses such as Zika and yellow fever. However, the study was done before the emergence of Zika virus in this coastal Province and where there have been no reported human cases of yellow fever for over 50 years. The antigen used for coating plates for DENV was purified Dengue 2 virus proteins from strain 16681, cross-reactive across dengue serotypes and likely also to be cross-reactive with other flaviviruses but not the alphavirus CHIKV. Plates for detection of CHIKV specific-IgG were coated with recombinant envelope protein 2 of CHIKV which appears to be highly specific for the detection of this virus even among other alphaviruses (Kam et al. PLoS Negl Trop Dis 2015; 9: e3445; Fumagalli et al Virol J 2018; 15: 112). Further there was no evidence of a correlation between OD values for the IgG responses to DENV and CHIKV (r=-0.0201),

For peer review only - http://bmjopen.bmj.com/site/about/guidelines.xhtml

illustrated by the quite distinct age-dependent seroprevalence curves for DENV and CHIKV (Figure 2) and limited cross-reactivity. Further, testing of samples from the same cohort from 2013 (i.e. before the appearance of CHIKV in Ecuador) showed no positive tests for CHIKV but a similarly high prevalence of DENV, making significant cross-reactivity between DENV and CHIKV unlikely. Unfortunately, few if any serological tests are 100% specific. There will always be a small proportion of false positives (likely to be low as indicated by the previous observations). Such measurement errors, however, are unlikely to be systematic and will reduce the precision of the measurements and power to show an effect, but they do not invalidate the effects that can be shown.

Comment 3: Concerning the model itself, I could not understand in detail how was it applied. It would be interesting to better explain at least its fundamentals in the methodology section. Besides, being these arboviruses infections multifactorial, I am wondering if this modelling should not also include other variables concerning ecological (climate, vector, etc.) and clinical (fatality rate, asymptomatic estimations, etc.) parameters.

Response 3: We agree that the statistical modelling approach is complex which is why we have provided more complex methods in the supplement (see Response 1). We have changed the presentation in the statistical methods which we hope are now much clearer and have added further clarifications to the supplementary material.

Unmeasured variables that may influence the prevalence of past exposures including climate and vector-related factors are accounted for in the random effects. Unmeasured asymptomatic infections are captured within seroprevalence data given that these viruses induce long-lasting immunity: thus, a strength of using serological data. Most modelling papers on DENV and CHIKV viruses make this assumption given the low figures for case fatality (death ration among positive individuals). Furthermore, fatality ratios are related to severe dengue (references [35,36,40,41] are very informative in this sense). In a study in Brazil, only 1% were diagnosed with severe dengue out of which only 4% died (that is less than 1% of all, i.e. 0.04%).

The assumptions underlying our modelling approach are reasonable and not new in relation to this type of modelling. Moreover, the ability to account for mortality (a rare event) is only possible with data from very large samples. The same is true to a lesser extent of individual clinical characteristics, not of relevance to our specific study question. Only the most important parameters which drive an epidemiological process could be robustly estimated from our data (e.g. prevalence of exposure by age and relevant individual/household). Our data provide useful information for policy makers with respect to, for example, the potential feasibility of dengue vaccination.

Comment 4: The exposed results appear to be in line with those raised in methodology; however, as stated above, it is necessary to make adjustments on them to improve their

interpretation. Particular comments can be found on the attached file.

Response 4: Please see below our complete responses to the specific comments below and in the resubmitted manuscript.

Comment 5: *Although the discussion assessed the main results and limitations of this study, I feel it is necessary to make a more in-depth analysis of the results obtained in this study, including a potential comparison with different serotypes of DENV and other arboviruses in the region (they make a brief mention that is not enough). To base all conclusions just on these two viruses, and not considering DENV as four different serotypes, I do not believe that it correctly reflects what is actually happening. I also feel that they need to be further elaborated on regarding the public health significance of these results and how vaccine implementation could interfere (or not) in the results here presented.*

Response 5: We thank the reviewer for acknowledging our discussion of the main results and limitations of this study. We agree that an analysis of DENV-serotype responses would be of interest in this population but such an analysis is beyond the scope of the aims of the present study. The four DENV serotypes have been reported to be present in Ecuador since 2000 (Ramos-Castañeda et al. PLoS Negl Trop Dis 2017), and different outbreaks in space and time are likely to be caused by different serotypes/genotypes. The analysis of serotype-specific responses, not directly relevant to the specific aims of our project, would require a much larger sample to be assessed adequately, a different type of sample relevant to this specific question, and use of more specialized and specific serologic assays (plaque reduction neutralization assays) that are not presently done by any laboratory in Ecuador. Our aim in the present study was to define seroprevalence of DENV irrespective of serotype to allow us to estimate overall exposure to DENV. We are not aware of the circulation of other arboviruses in this region at the time this study was conducted. Zika virus appeared in the region after sample collection was done; the first cases of Mayaro virus infection in coastal Ecuador were identified in 2019 (https://www.salud.gob.ec/se-confirman-los-5-primeros-casos-con-el-virus-mayaro-en-el-pais/); and the first case of Oropouche virus in Esmeraldas Province was identified in 2016 (Wise et al., Emerg Infect Dis 2016). These observations don't indicate significant circulation of other arboviruses in the study area at the time we conducted the study. We address the vaccination issue with additional comments and references. We have appended a new paragraph (below) to the discussion in the light of a novel (2020) publication added to the references list (#22 in the list). The research presented there did not account for different DENV serotype either.

"Highlighting areas of high prevalence is important as recent analyses suggested that transmission-blocking interventions such as *Wolbachia*, even at intermediate efficacy (50% transmission reduction), might reduce global annual disease incidence by up to 90%. The newly licensed Sanofi-Pasteur vaccine, targeting only *seropositive* recipients, (namely those at risk of secondary infection which can result in severe dengue and subsequent death), might

For peer review only - http://bmjopen.bmj.com/site/about/guidelines.xhtml

reduce global annual disease incidence by 20 to 30%, with the greatest impact in high-transmission settings"

Reviewer 2 detailed comments from the pdf file annotated by the reviewer

Please note that the comments are identified by the page number, as labelled by the journal editorial on the top of the page, alternatively on the right/left corner, and line number, as labelled by the journal editorial on the left of that page.

Page 4, Line 19-20:

Authors meant the statistical method?
Response: Yes, we have change as requested – it is now "…the statistical technique"

Page 6, Line 6:

This might not be consistent with the last paragraph of the manuscript. Please, check it up.
Response: We have changed the last statement for consistency with this paragraph. We have now introduced a Patient and Public Involvement statement which reads:

"The cohort was initially set up to understand how early childhood infections affect the development of inflammatory diseases in childhood. Participants were clearly explained the public health benefits of the study prior to recruitment and informed written consent was obtained prior to recruitment and at several stages during the course of this longitudinal study (e.g. at 5 and 8 years also)."

We have also altered the Ethics statement which reads:
"Informed written consent was obtained from participants aged 18 or more, and from a parent or legal representative for a minor. Minor assent was obtained from children aged 8 years or older. The study protocol was approved by the Ethics Committee of the Universidad Internacional del Ecuador, Quito, Ecuador."

Page 7, Line 11:

dengue=disease
DENV= dengue virus
chikungunya=disease
CHIKV= chikungunya virus

It would be interesting to denote as well, that even though they are both arboviruses, they belong to different families and genres.
Response: Thank you – the terminology is important. We have added to the text the viral families to which these arboviruses belong and an additional reference [1]. The paragraph is changed to:

"Dengue virus (DENV) and chikungunya virus (CHIKV), arboviruses belonging to *Flaviviridae* and *Togaviridae* families, respectively, are endemic in many tropical and sub-tropical regions where *Aedes* vectors thrive."

For peer review only - http://bmjopen.bmj.com/site/about/guidelines.xhtml

Page 7, Line 40:

Case notifications may also include cases detected by other diagnosis techniques, or just clinically, etc. Unless serosurveys get implemented and widely applied to a population, I don´t see how they can be comparable to case notifications.

Response: Thank you We hope we added some clarity to this statement. We changed the terminology from case-notification to clinically classified. The paragraph reads now:

"Cross-sectional serological surveys can provide valuable information on arbovirus infection burdens and allow more accurate determination of numbers exposed. Serological surveys, thus, have a number of advantages over clinically classified, self-reported cases or other non-objective clinical diagnoses by providing information that are less sensitive to recall, underreporting, and misdiagnosis due to asymptomatic manifestations of related infections".

Page 8, Line 43:

It is understood from this statement that your particular sub-cohort included children recruited during the period 2006-2009.

Consider re-writing this section. Go from the general to particular... otherwise, it gets really confusing.

Response: [illegible]

Page 9, Line 13:

Please add a different subheading for this paragraph

Response: We have now added a paragraph dedicated to laboratory testing and name it as such, just before the Statistical methods section.

Page 9, Line 13

If they were already abbreviated before, then use DENV and CHIKV

Response: We have addressed that.

Page 9, Line 16-18 (repeated comment)

Can exposure be correlated directly to seropositivity?

What about non-responders? Incubation window?

What about seropositivity due to cross-reaction? Even more considering flaviviruses, zikv infection for exm.

Response: The serum data collection took place in the aftermath of the 2015 epidemics in Ecuador. We have defined seropositivity by the means of presence of viruses' specific antibodies and subsequently assumed that seropositivity indicated past exposure to those viruses. There is a great body of literature in modelling these viruses which are based on the assumptions of long-life immunity following exposure, many of which are cited in this paper.

For peer review only

Page 9, Line 29-30

Presence/absence of DENV or CHIKV would be referring to the direct detection of the virus, which is not the case. Please be aware of this. I guess authors meant antibodies against DENV or CHIKV, or evidence of past dengue or chikungunya infection. This should be clarified.

Response: This is precisely the case - we have modified the paragraph in the light of this comment and started with the definition of the statistical outcome or the dependent variable as we call it. The beginning of the Statistical Methods section reads now as:

"The presence or absence of DENV and CHIKV antibodies define a bivariate binary outcome indicating past exposure to DENV only (D+C-), to CHICK only (D-C+), to both (D+C+) or to none. The key assumption of the model is that of long-lasting immunity [25] after infection with DENV and CHIKV."

Page 9, Line 29-34

Not clear to me. Which model? The multilevel logistic regression? So, actually, it is an existing model you are adapting to arbovirus infections modelling.

Response: The very basic model we refer to can be found in the reference [26]. Our model is not a simple adaptation of that model. Adding two levels of correlated random effects associated with potentially unmeasured variables at household and ward level, including mosquitos' burden and bites' intensity, water presence around the house, weather, or ward population dynamics in general brings a series of complexities in the approach.

Page 9, Line 41

Which ones? It will be clearer if you present first what are the variables you are taking into account in the model. It is difficult to understand what you are trying to determine using this model otherwise.

Response: We have changed the statistical methods section to quite a greater extent hoping that it flows better. We have moved the variables taken into account in the model after clarifying the definition of the outcome (the dependent variable).

Page 9, Line 50

It is not clear to me, age-dependent prevalence isn´t one of the parameters being estimated, using the serology and household info? How is it that it can infer co-exposures in the past if CHIKV was introduced in 2015?
Moreover, how are you managing the different DENV serotypes infections and the respective serological response?

Response: Age dependent prevalence is a concept in epidemiology which refers to the risk of disease (infection) according to age (can be age groups) in the population. It

For peer review only - http://bmjopen.bmj.com/site/about/guidelines.xhtml

usually answers the question of how the disease risk varies with age and what are the factors which confound this potential association between disease and age.

In this particular context, of viral infections which result in long-life immunity, we are talking about the age-dependent prevalence of past exposure to those viruses. This can be thought as the age-dependent prevalence of immunity to those viruses in the population. This is a very important aspect to understand at population level as various control measures can be implemented effectively, i.e. targeting groups at most risk.

Page 10, Line 3-13

It is not clear at all for this reviewer what are you infering from this. You have the serological test result plus patients age information. These are not being infered. Which predictions were plotted?

Response: I think there is some confusion on what inference concept means in statistics. Indeed, we have serological test indicating past exposure to DENV/CHIKV results and patients age information. Our model develops a systematic trend, numerically quantified association between age and the risk of past exposure to these viruses as explained above. Moreover, proper statistical inference assigns uncertainties (the 95% credible intervals) around the numbers which express numerically this association. Accounting for the rest of the variables can change this association and the associated uncertainty.

Predictions are precisely those curves in Figures 2 and 3 - that is how our model infers numerically the risk of past exposure (or the prevalence of immunity) in the population.

Page 10, Line 20

This paragraph is crucial. It should be presented before.

Response: We reshuffled the statistical methods paragraph to a quite great extent.

Page 10, Line 35-39

If applying this model to DENV/CHIKV infection is the key point that authors intend to show, they should consider including the model parameters and the approach employed in the main text, leaving the accessory processing for the supplementary information.

Response: We are not sure if we can make sense of this request. But we think the paper flows better with a lay presentation of the methodology and the mathematics and associated technicalities presented in the Supplementary information.

For peer review only - http://bmjopen.bmj.com/site/about/guidelines.xhtml

Page 10, Line 35-39

This is not the same. To be at risk doesn't necessarily mean to get infected and thus, to seroconvert.

Response: This is not what we state at all, i.e. **to be at risk**. Here we are talking about the risk of past exposure to these viruses which is a very different concept from that of being at risk. The sentence has changed for clarification to: "Given the assumption of life-long immunity, age-dependent seroprevalence and risk of past exposures to DENV and CHICKV, are used interchangeably."

Page 10, Line 52

Please reformulate. There were 2404 children enrolled in the birth cohort, but only some of them participated in this particular study. This sub-group plus their relatives sharing household represent the 319 individuals of this study. Am I right? Population under study is not clear and needs to be better defined. Since the birth cohort is not the main focus of this analysis, I would recommend avoiding its continuous citing, cause it is confusing.

Response: We did reformulate. We think that it is important to cite the background cohort – it is one of the important features of this research. It now reads as "**Error! Reference source not found.** shows the geographical locations of households of children in the background cohort and the locations of those sampled for this analysis (151)."

Page 10, Line 56

Did the authors determine this information? If they did, then how was it determined? If they didn't, then please consider moving it from this section to the introduction, or discussion, and adequately reference it.

Response: This is part of the results and represent the cumulative number derived by summing weekly reported infection using publicly available websites. The numbers are not part of our modelling strategy but it gives the readers perspective into the extent of the 2015 epidemics.

Page 11, Line 8

There is no need to present the information as a table and as a graph. I would strongly recommend choosing one of them.

Response: Table 1 presents cumulative numbers and Figure 1 presents the epidemic curves, i.e. the weekly number of cases. The total (cumulative number) is not informative enough for infections of relatively short duration and short dynamics as it would be in case of chronic diseases outcomes. Weekly incidence is necessary to be reported too so the reader can understand the course of the epidemic.

For peer review only - http://bmjopen.bmj.com/site/about/guidelines.xhtml

Page 11, Line 11

319 children? or 319 children+household contacts?
Response*:* The question has been addressed above.

Page 11, Line 11

from 49 wards?

Response*:* yes – that has been clarified in the text.

Page 11, Line 13

The word baseline seemed out of place here. Or do the characteristics change at any point?
Response*:* We have removed it.

Page 11, Line 18

High proportions of...? Consider removing vague terms. Use instead real numbers.
Response: We have changed it accordingly and referred to numbers too.

Page 11, Line 42-49

Unclear. Please reformulate. By estimations, authors mean the calculated probability? It is still confusing for me what are the authors trying to model.
Response: The ORs (the estimates) are measures of association between the presence of DENV or CHICK antibodies (past exposures to these viruses) with age, sex, etc. The sentence changed to: "*Parameter estimates (conditional odds ratios) which measure the univariate and adjusted associations of the risk of testing positive for DENV, CHIKV and both with each potential risk factor (age, sex, area of residence, socio-economic status and distance to next door neighbour) are shown in Table 2.*"

Page 11, Line 49-55

Difficult to understand.
Besides, why are the authors talking about "predicted seroprevalence"? Didn't they obtain that information directly from the ELISA test?
Response: We think the confusion stems for similar terminology used in laboratory environment and statistics. In this context, the Elisa tests (probably by some prediction methodology which has a completely very different meaning from that of statistical prediction) provided the raw information which defined our binary bivariate outcome or dependent variable as we call it.

Page 11, Line 58

For peer review only - http://bmjopen.bmj.com/site/about/guidelines.xhtml

Abbreviations must be explained the first time they are used. CrI= confidence interval? It is commonly abbreviated as CI, as authors mentioned in the methodology section. Please be consistent, CrI or CI.
On the other side, by average, you meant the mean? The median? How did you get to the percentage value? Did the authors suggest that 84% of the individuals of this cohort tested positive for dengue? If so, the word average is confusing. Consider removing it.
Moreover, which ages define what an infant is? 5 years-intervals stratified ages.
Response: In Bayesian inference, the credible intervals play a similar role as confidence intervals in classical statistical inference – that of assessing the uncertainty of an estimate. Confidence Intervals (usually denoted by CI) are however different concepts from Credible Intervals. We clarified that we referred to credible intervals usually denoted by CrI.

Page 12, Line 6

Not obvious from the graph. Seemed to be before 10.
Response: We have corrected that to around 9 years.

Page 12, Line 11-14

What's the difference between Fig2 and Fig3 D+C- / D-C+??
Response: Figure 2 presents the predicted (from the model) age-dependent prevalence of all DENV and CHIKV past exposures whilst Figure 3 presents the predicted (from the model) age-dependent prevalence for past exposure to DENV only, to CHIKV only and to both only. The y-values on continuous line in Figure 2 are the sum between the y-values between the first and the third panel in Figure 3 (DENV) and the sum between the y-values between the first and the second panel in Figure 3. Both are of epidemiological interest.

Page 12, Line 14-21

I must assume that serological test results were introduced in your model, and then you compare estimations with real serological testing results. However, this is not clear through the manuscript. I would strongly recommend to improve the writing and reformulate the methodology section so to clarify how this model is applied and which is its purpose.
On the other hand, how is it possible to be predicting a probability lower than 3% for D-C+ but between 8-50% for D+C+?
Response: The reviewer is referring essentially to the goodness of fit. We have romped up the goodness of fit tailored to this model, inspired by the basic logistic regression. We add a section in the Supplementary and provided extra clarity on this.

Regarding the second request, the results regarding CHIKV proves that figures associated with past exposure to CHIKV only are actually low. The D+C+ figures of 8-50% reflects the high burden of co-circulation of the two viruses in 2015.

For peer review only - http://bmjopen.bmj.com/site/about/guidelines.xhtml

Page 12, Line 52

vague term

Response: "Less precise" is a term used in statistics when the uncertainty (CrI) is larger. Usually, the larger the number of variables involved in an epidemiological process, the larger the uncertainty. In other words, more information but less precise. The uncertainty associated with an estimated parameter is equally important as the parameter itself.

Page 13, Line 13

Which one?

Response: All of them – the sentence has been changed.

Page 13, Line 17

No y-axis title in suppl fig 8 nor 9.

Response: That has been fixed.

Page 13, Line 42-44

Which ones? Not clear what authors meant to say.

Response: This is a generic statement, i.e. "Nevertheless, the epidemiological and statistical methodologies can straightforwardly accommodate for more multiple concomitant exposures". We did not have to mention which – we wanted to highlight the flexibility of the methodology to extend to outcomes which can reflect past exposures to multiple related viruses.

Page 13, Line 48

This reference doesn't correspond to the stated information
Response: We think it does. The work contains worldwide maps with countries where serological studies have been conducted and the Ecuador map indicates no serological information. (Fritzell, C., et al., *Current challenges and implications for dengue, chikungunya and Zika seroprevalence studies worldwide: A scoping review*. PLoS Neglected Tropical Diseases, 2018. **12**(7))

Page 13, Line 51-54

Seems to be somehow expectable considering that DENV is endemic in Ecuador and 0 to 60 yo is a wide range.
Response: We are glad that our findings reflect those of empirical observations. As mentioned above, estimates and associated uncertainties are important to inform effective control strategies.

For peer review only - http://bmjopen.bmj.com/site/about/guidelines.xhtml

Page 14, Line 13

Interesting. Please cite all corresponding studies.
Response: the references are correctly assigned. We are bounded to a max number of 40 references and already exceeded it. We chose references which provided a comprehensive review of the existing literature.

Page 14, Line 27

This is not the correct reference. Please cite the real ones referencing the mentioned studies.
Response: Please see above

Page 14, Line 50-51

It is expected to find a lower prevalence for CHIKV exposure (compared to DENV), since the first outbreak was evidenced in 2015 and sampling of this study was between May and Sep 2016.

Considering this, how can the authors trust in the results obtained for Chikv+ estimations?
Response: Exposure to CHIKV results in the presence of specific antibodies and therefore a seropositive individual was considered as exposed to CHIKV at some point in time prior to data collection.

Page 14, Line 56

Also, consider that the region under study was defined as a rural area. Thus, it would be expected to detect a difference for male gender due to field activities.
Response: Table 1 indicated quite the contrary, 68% of households were deemed as urban and the rest were rural. We think that the interpretation of the results is informative.

Page 15, Line 10-13

Two comments in this regard:
**Vectorial competence should be considered in this study since it is focusing on a short period when two simultaneous outbreaks were taking place. Mosquitoes populations could have been more competent to transmit CHIKV rather than DENV.*
**On the other side, in the region under study DENV cases were considerable lower than CHIKV ones. A previously immune population would likely difficult or at least decrease DENV transmission (considering same DENV serotype).*
It would be interesting to extend the epidemiological data presented about these two viruses, and other relevant arboviruses as well.

For peer review only - http://bmjopen.bmj.com/site/about/guidelines.xhtml

As an example, while Brazil was experiencing a huge DENV2 outbreak last year, the state of Rio de Janeiro presented just a few cases, but a massive chikungunya outbreak instead. Several studies had proposed/demonstrated how vectorial competence is involved in different arboviruses cycles, together with the immune status of the population.

Response:

Evaluation of vectorial competence would require collections of vectors and molecular analyses during the outbreaks for the presence of the two viruses, as well as more sophisticated laboratory-based studies using A. aegypti. In this analysis, we used a study sample with blood collection after and not during the outbreaks. We measured levels of IgG that, after a first exposure, would require a 30-days or so to reach maximal levels and which would tell us about past exposure. Although we agree that studies of vector competence during simultaneous outbreaks of DENV and CHIKV are of scientific interest, they are beyond the scope of the present analysis and study sampling. We have added a statement to the Discussion on the value of studies of vector competence t our understanding of the evolution of two infection occurring simultaneously in time and through the same vector.

Page 15, Line 46

This is a strong statement.

Response: Replaced “unreliable” with “uncertain”.

Page 15, Line 48-53

Authors should take care with this kind of statement, and consider toning it down since serological surveys are extremely useful to determine the prevalence of a disease. Still, they may not entirely represent the impact/burden that the disease may represent. To start with, dengue, for example, presents high levels of subclinical infection. Moreover, a vaccine against DENV was implemented in different countries, which can raise the population seropositivity.

Otherwise, please provide justifying evidence.

Response: We are not entirely sure if we understand this comment. We stand by our original statement which advocates the usefulness of serological surveys as a powerful tool to understand population past exposure to viruses. In case of subsequent long-lasting immunity, they provide a measure of immunity in the population.

On the other hand, to our best knowledge there has been a long debate regarding most effective strategies for DENV control including vaccination. There are very new guidelines in this sense – two very novel and informative papers [20, 21] published in 2019 and 2020 are cited here. One of them (The Lancet) was cited in the original submission.

Page 16, Line 42-53

This information should be presented in the methodology.

For peer review only - http://bmjopen.bmj.com/site/about/guidelines.xhtml

The authors mentioned that this modelling would contribute to these arboviruses burden estimations. However, if the fatality rate is dismissed even for denv infection, wouldn´t the final predictions be biased, or underestimated?
It seems to me that both asymptomatic infections and fatality cases should be taken into account.

Response: The case fatality ratios appear to be low in the population as presented in references [35, 36]. Most modelling papers on these viruses made this assumption of negligible mortality given these low figures for case fatality ratios. Furthermore, fatality ratios are related to severe dengue (references [40,41] are very informative in this sense). In a study in Brazil, only 1% were diagnosed with severe dengue out of which only 4% died (that is less than 1% of all, i.e. 0.04%)

We clearly set out our assumptions which are reasonable and not new in relation to this type of modelling.

Having said this, unless operating with very large numbers of individual data, it is impossible to account for mortality, DENV serotypes and all otherwise important biological aspects. No matter how complex an epidemiological process in a population, only the most important parameters which drive that process can be robustly estimated from the data. In our research setting, that is the prevalence of past exposure by age of two important co-circulating viruses and a series of important population characteristics which would inform policy makers for effective vaccination.

Page 16, Line 53

Even more, I feel that the four serotypes should be modelled separately. Perhaps, immune response for heterotypic denv infections should be considered too, since non-neutralizing antibodies when in a certain titer, may cause ADE. The following publications could be helpful:
**Katzelnick LC, Gresh L, Halloran ME, et al. Antibody-dependent enhancement of severe dengue disease in humans. Science. 2017;358(6365):929–932. DOI:10.1126/science.aan6836*
**Salje H, Cummings DAT, Rodriguez-Barraquer I, et al. Reconstruction of antibody dynamics and infection histories to evaluate dengue risk. Nature. 2018;557(7707):719–723. DOI:10.1038/s41586-018-0157-4*

Response: This question has been addressed above to some extent. Population studies are different from laboratory studies. Serological studies combine them but it is impossible to accurately identify patterns in the populations accounting for all biological aspects in the lab. Large amounts of detailed laboratory data can be logistically difficult particularly in low-middle income countries. But this is what statistics do – from a relatively small sample which represents a population, through appropriate modelling, derives conclusions valid for that population. Only the most

For peer review only - http://bmjopen.bmj.com/site/about/guidelines.xhtml

important questions can be estimated with reasonable precision. For example, some estimate of a proportion of 0.5 with a CI between 0.01 and 0.99 is equivalent with *no information* at population level on what that proportion can represent.

Page 16, Line 58-60

Not clear.

Response: The reference [40] is very informative for case fatality ratios.

Page 17, Line 17-20

This is not a minor issue. I strongly recommend the authors to include epidemiological data of ZIKV in Ecuador; even better if there is any from the region under study. Serological cross-reaction is one of the main concerns about these tests. It should be taking into account. Mostly for flaviviruses. What about YFV?

Response: Our methodology accommodates for past exposure to a third related virus. At the time we started analyses of this data we did not hold serological results associated with Zika virus. Having said this – it is not straightforward to analyse samples in the lab and to re-analyse the data. It would be another year of work at least. But we do have plans in these regards.

Page 17, Line 30-34

But this information comes from the serological tests + patient's data and seems to be representing the mere epidemiology of both, DENV and CHIKV infections.

Response: In the light of the new vaccination guidelines, simply said, we put numbers to assess this dependence. This is always important control strategies may be costly and in the absence of population level predictions choosing the most effective strategy is impossible.

Page 18, Line 34

Please adjust font and size to correlate with Age column

Response: We fixed that.

Page 18, Line 54

How did the authors calculate this? Which is the reference point? Total n should not be 151? Didn't the authors take each house as a reference and measure the distance to the next most proximal one?

Response: We made the necessary corrections.

Page 20, Line 6 (in the Table 2)

For peer review only - http://bmjopen.bmj.com/site/about/guidelines.xhtml

What does power mean?

Response: We added clarity in the Table 2 legend. The power is a parameter to be estimated of the age ^{power} component of the model. For example, that can be age ^{2}. We estimated the power as 7.6 – that is translated in a very fast increase of past exposure to DENV with age - see Figures and Table 2.

Page 20, Line 56

this would be the prevalence....

Response: We disagree on the relevance of a proportion of cases on a particular year associated with this type of infections.

Page 22, Line 4

I would recommend to re-write this figure title

Response: Spatial distributions of the household locations of the background population (ECUAVIDA) and the serological sample.

Page 22, Line 31-36

17-20

Response: We left each reference in case the original order is changed.

Page 25, Line 8-9

Patient's samples and data are involved. Do you have a written consent from them? Since this study includes children, a legal representant must allow for using all this information. If waived, please inform it. Also authors would need to include a statement referring to the ethical comitee aproval.

Response: We have now introduced a Patient and Public Involvement statement which reads:

"The cohort was initially set up to understand how early childhood infections affect the development of inflammatory diseases in childhood. Participants were clearly explained the public health benefits of the study prior to recruitment and informed written consent was obtained prior to recruitment and at several stages during the course of this longitudinal study (e.g. at 5 and 8 years also)."

We have also altered the Ethics statement which reads:

"Informed written consent was obtained from participants aged 18 or more, and from a parent or legal representative for a minor. Minor assent was obtained from children aged 8 years or older. The study protocol was approved by the Ethics Committee of the Universidad Internacional del Ecuador, Quito, Ecuador."

Page 34 (Figure)

y axis?

For peer review only - http://bmjopen.bmj.com/site/about/guidelines.xhtml

Response: We fixed that

Page 38 (Figure)

x axis?

Response: We fixed that

Page 47, Line 29

variables need to be define at first instance.... what is R, what is K, etc. What are they representing?

Response: We think that the supplementary information reads very well   tthere are clear statements on what R, K represent.

For peer review only

STROBE 2007 (v4) checklist of items to be included in reports of observational studies in epidemiology*
Checklist for cohort, case-control, and cross-sectional studies (combined)

Section/Topic	Item #	Recommendation	Reported on page #
**Title and abstract**	1	(a) Indicate the study's design with a commonly used term in the title or the abstract	#1 Title:Age-dependent prevalence
		(b) Provide in the abstract an informative and balanced summary of what was done and what was found	#3 Abstract
**Introduction**			
Background/rationale	2	Explain the scientific background and rationale for the investigation being reported	#6 Introduction
Objectives	3	State specific objectives, including any pre-specified hypotheses	#7 Introduction
**Methods**			
Study design	4	Present key elements of study design early in the paper	#8 Materials and methods
Setting	5	Describe the setting, locations, and relevant dates, including periods of recruitment, exposure, follow-up, and data collection	#8 Materials and methods
Participants	6	Cross-sectional study—Give the eligibility criteria, and the sources and methods of selection of participants	#8 Materials and methods
		(b) Cohort study—For matched studies, give matching criteria and number of exposed and unexposed<br>Case-control study—For matched studies, give matching criteria and the number of controls per case	
Variables	7	Clearly define all outcomes, exposures, predictors, potential confounders, and effect modifiers. Give diagnostic criteria, if applicable	#8-9 Statistical Methods
Data sources/ measurement	8*	For each variable of interest, give sources of data and details of methods of assessment (measurement). Describe comparability of assessment methods if there is more than one group	#8 Materials and methods
Bias	9	Describe any efforts to address potential sources of bias	#6, #16
Study size	10	Explain how the study size was arrived at	#8 – random sample from an existing cohort
Quantitative variables	11	Explain how quantitative variables were handled in the analyses. If applicable, describe which groupings were chosen and why	#8 Materials and methods
Statistical methods	12	(a) Describe all statistical methods, including those used to control for confounding	#8-9 Statistical Methods

For peer review only

		(b) Describe any methods used to examine subgroups and interactions	#8-9 Statistical Methods
		(c) Explain how missing data were addressed	NA
		(d) *Cohort study*—If applicable, explain how loss to follow-up was addressed<br>*Case-control study*—If applicable, explain how matching of cases and controls was addressed<br>*Cross-sectional study*—If applicable, describe analytical methods taking account of sampling strategy	#8-9 Statistical Methods Random sample from an existing cohort. Weighting adjustments to represent the population
		(e) Describe any sensitivity analyses	NA
**Results**			
Participants	13*	(a) Report numbers of individuals at each stage of study—eg numbers potentially eligible, examined for eligibility, confirmed eligible, included in the study, completing follow-up, and analysed	319
		(b) Give reasons for non-participation at each stage	NA
		(c) Consider use of a flow diagram	NA
Descriptive data	14*	(a) Give characteristics of study participants (eg demographic, clinical, social) and information on exposures and potential confounders	#18 Table 1
		(b) Indicate number of participants with missing data for each variable of interest	NA
		(c) *Cohort study*—Summarise follow-up time (eg, average and total amount)	NA
Outcome data	15*	*Cohort study*—Report numbers of outcome events or summary measures over time	
		Case-control study—Report numbers in each exposure category, or summary measures of exposure	
		Cross-sectional study—Report numbers of outcome events or summary measures	#18 -19 Table 1, Table 2
Main results	16	(a) Give unadjusted estimates and, if applicable, confounder-adjusted estimates and their precision (eg, 95% confidence interval). Make clear which confounders were adjusted for and why they were included	#10 Results
		(b) Report category boundaries when continuous variables were categorized	#18 -19 Table 1
		(c) If relevant, consider translating estimates of relative risk into absolute risk for a meaningful time period	#10 Results – Supplementary Tables
Other analyses	17	Report other analyses done—eg analyses of subgroups and interactions, and sensitivity analyses	#10 Results<br>#13-16 Discussion
**Discussion**			
Key results	18	Summarise key results with reference to study objectives	#13-16 Discussion
Limitations	19	Discuss limitations of the study, taking into account sources of potential bias or imprecision. Discuss both direction and magnitude of any potential bias	#4 Strength and limitations

For peer review only

			#13-16 Discussion
Interpretation	20	Give a cautious overall interpretation of results considering objectives, limitations, multiplicity of analyses, results from similar studies, and other relevant evidence	#13-16 Discussion
Generalisability	21	Discuss the generalisability (external validity) of the study results	#13-16 Discussion
**Other information**			
Funding	22	Give the source of funding and the role of the funders for the present study and, if applicable, for the original study on which the present article is based	#24 Wellcome Trust (088862/Z/09/Z)

*Give information separately for cases and controls in case-control studies and, if applicable, for exposed and unexposed groups in cohort and cross-sectional studies.

Note: An Explanation and Elaboration article discusses each checklist item and gives methodological background and published examples of transparent reporting. The STROBE checklist is best used in conjunction with this article (freely available on the Web sites of PLoS Medicine at http://www.plosmedicine.org/, Annals of Internal Medicine at http://www.annals.org/, and Epidemiology at http://www.epidem.com/). Information on the STROBE Initiative is available at www.strobe-statement.org.

For peer review only

BMJ Open

Age-dependent seroprevalence of dengue and chikungunya: inference from a cross-sectional analysis in Esmeraldas Province in coastal Ecuador

Journal:	*BMJ Open*
Manuscript ID	bmjopen-2020-040735.R1
Article Type:	Original research
Date Submitted by the Author:	19-Jul-2020
Complete List of Authors:	Chis Ster, Irina; St. George's University of London, ; St George's University of London<br>Rodriguez, Alejandro<br>Romero, Natalia; Universidad Internacional del Ecuador, Quito<br>Lopez, Andrea; Universidad Internacional del Ecuador, Quito<br>Chico, Martha; Universidad Internacional del Ecuador, Quito<br>Montgomery, Joel ; CDC<br>Cooper, Philip; St George's University of London, Institute of Infection and Immunity
**Primary Subject Heading**:	Infectious diseases
Secondary Subject Heading:	Epidemiology
Keywords:	Epidemiology < TROPICAL MEDICINE, STATISTICS & RESEARCH METHODS, PUBLIC HEALTH

SCHOLARONE™
Manuscripts

I, the Submitting Author has the right to grant and does grant on behalf of all authors of the Work (as defined in the below author licence), an exclusive licence and/or a non-exclusive licence for contributions from authors who are: i) UK Crown employees; ii) where BMJ has agreed a CC-BY licence shall apply, and/or iii) in accordance with the terms applicable for US Federal Government officers or employees acting as part of their official duties; on a worldwide, perpetual, irrevocable, royalty-free basis to BMJ Publishing Group Ltd ("BMJ") its licensees and where the relevant Journal is co-owned by BMJ to the co-owners of the Journal, to publish the Work in this journal and any other BMJ products and to exploit all rights, as set out in our licence.

The Submitting Author accepts and understands that any supply made under these terms is made by BMJ to the Submitting Author unless you are acting as an employee on behalf of your employer or a postgraduate student of an affiliated institution which is paying any applicable article publishing charge ("APC") for Open Access articles. Where the Submitting Author wishes to make the Work available on an Open Access basis (and intends to pay the relevant APC), the terms of reuse of such Open Access shall be governed by a Creative Commons licence – details of these licences and which Creative Commons licence will apply to this Work are set out in our licence referred to above.

Other than as permitted in any relevant BMJ Author's Self Archiving Policies, I confirm this Work has not been accepted for publication elsewhere, is not being considered for publication elsewhere and does not duplicate material already published. I confirm all authors consent to publication of this Work and authorise the granting of this licence.

For peer review only

Age-dependent seroprevalence of dengue and chikungunya: inference from a cross-sectional analysis in Esmeraldas Province in coastal Ecuador

For peer review only

Irina Chis Ster[1], Alejandro Rodriguez[2], Natalia Romero[2,5], Andrea Lopez[2], Martha E Chico[3], Joel Montgomery[4], Philip J Cooper[1-3].

[1]Institute of Infection and Immunity, *St George's University of London, London SW17 ORE*, United Kingdom; [2]School of Medicine, *Universidad Internacional del Ecuador, Quito, Ecuador*; [3]*Fundacion Ecuatoriana Para Investigacion en Salud, Quito, Ecuador*; [4]*Centers for Disease Control and Prevention*, Atlanta, Georgia, USA; [5]*Grups* de *Recerca d'America* i *Africa Llatines*.

Conflict of Interests: NONE declared.

Correspondence: Dr Irina Chis Ster, (ichisste@sgul.ac.uk)

Dr Irina Chis Ster

Senior Lecturer in Biostatistics

Institute of Infection and Immunity, St George's University of London

For peer review only - http://bmjopen.bmj.com/site/about/guidelines.xhtml

Dengue and Chikungunya in Ecuador

Cranmer Terrace | Tooting | London | SW17 0RE

Tel: +44 (0)208 725 4937

Email: i.chisster@sgul.ac.uk

Contributors:

Contributors: PJC and ICS designed the study. PJC and MEC supervised data and sample collection. AL did the laboratory assays. ICS developed and implemented the statistical methodology and conducted the literature research. AR provided data and contributed to spatial data analyses. ICS and PJC drafted the manuscript. NR and JM reviewed and provided critical comments on the manuscript. All authors reviewed and approved the final version of the manuscript. ICS is the paper guarantor.

For peer review only

Abstract

Objectives: There are few population-based estimates for prevalence of past exposure to dengue and chikungunya viruses despite common epidemiological features. Here, we have developed a novel statistical method to study patterns of age-dependent prevalence of immunity in a population following exposures to two viruses which share similar epidemiological features including mode of transmission and induce long-lasting immunity. This statistical technique accounted for socio-demographic characteristics associated with individuals and households.

Settings: The data consist of a representative sample from an ongoing longitudinal birth cohort set up in a tropical District in coastal Ecuador (Esmeraldas).

Participants: We collected data and blood samples from 319 individuals belonging to 152 households following epidemics of the infections in 2015 in Latin America.

Primary outcome: Plasma was tested for the presence of specific IgG antibodies to dengue and chikungunya viruses by commercial enzyme-linked immunosorbent assays and defined a bivariate binary outcome indicating individuals' past exposure status to dengue and chikungunya (i.e. presence/absence of IgG antibodies to dengue or chikungunya or both).

Results: Dengue seroprevalence increased rapidly with age reaching 97% (95%CrI 93%-99%) by 60 years. Chikungunya seroprevalence peaked at 42% (95%CrI 18%-66%) around 9 years of age and averaged 27% (95%CrI 8.7%-51.6%) for all ages. Rural areas were more likely to be associated with dengue only exposure while urban areas and shorter distance to the nearest household were associated with exposures to both. Females living in urban settings were more likely to be chikungunya seropositive while rural males were more likely to be dengue seropositive.

For peer review only - http://bmjopen.bmj.com/site/about/guidelines.xhtml

For peer review only

Conclusion: Dengue seroprevalence was strongly age-dependent consistent with endemic exposure while that of chikungunya peaked in childhood consistent with the recent emergence of the virus in the study area. Our findings will inform control strategies for the two arboviruses in Ecuador including recommendations by WHO on dengue vaccination.

Strengths and limitations of this study

- This is the first serological study presenting population-based estimates on age-dependent prevalence of dengue and chikungunya in a region of Ecuador.
- The paper describes a novel statistical method and associated OpenBUGS code.
- The estimates account for individual and household characteristics
- The paper responds to recent WHO call for understanding local epidemiology of dengue prior to designing efficient control strategies.
- More research is needed to assess the impact of various control strategies in the community using these estimates and their uncertainties.

Ethics

Informed written consent was obtained from participants aged 18 or more, and from a parent or legal representative for a minor. Minor assent was obtained from children aged 8 years or older. The study protocol was approved by the Ethics Committee of the Universidad Internacional del Ecuador, Quito, Ecuador.

Acknowledgements

We thank to all participants and their families who agreed to participate in the Ecuavida cohort.

For peer review only - http://bmjopen.bmj.com/site/about/guidelines.xhtml

For peer review only

For peer review only - http://bmjopen.bmj.com/site/about/guidelines.xhtml

Introduction

Dengue virus (DENV) and chikungunya virus (CHIKV), arboviruses belonging to *Flaviviridae* and *Togaviridae* families, respectively, are endemic in many tropical and sub-tropical regions where *Aedes* vectors thrive. Although the two viruses belong to different arbovirus families *[1]*, human infections are associated with similar clinical manifestations and are a major cause of morbidity *[2-4]*. Over the past 15 years, the number of dengue cases reported in Latin America has increased dramatically *[5]* while chikungunya, which emerged in the Caribbean region in 2013, has spread rapidly through the region *[6]* causing an estimated 2.9 million cases *[7-9]*.

Global estimates of the burden of dengue and chikungunya are based on aggregate numbers of reported cases and likely to be biased by underreporting because of limited surveillance, high rates of asymptomatic infections, and non-specific clinical presentation *[10]*. Asymptomatic dengue, for example, can inflate the total number of cases by up to 40-fold *[11-13]*.

Cross-sectional serological surveys can provide valuable information on arbovirus infection burdens and allow more accurate determination of numbers exposed. Serological surveys, thus, have a number of advantages over clinically classified, self-reported cases or other non-objective clinical diagnoses by providing information that are less sensitive to recall, underreporting, and misdiagnosis due to asymptomatic manifestations of related infections *[6, 12-14]*.

Ecuador is a middle-income country in Latin America where dengue has been endemic since the late 1980s *[15]*: dengue is thought to have re-emerged around 1988 following re-colonisation with *A. aegypti* after a thirty-year absence *[16, 17]*. Chikungunya first appeared

For peer review only - http://bmjopen.bmj.com/site/about/guidelines.xhtml

in the Caribbean region in 2013, reaching Ecuador in 2014 causing a large outbreak the following year *[17] [18]*.

Dengue transmission dynamics has been estimated from aggregated data in a series of studies *[19, 20]* with the latter suggesting that the picture provided by serology is very incomplete and limited when trying to characterize transmission across time and space both within and between countries and calling for local data analyses.

Most seroprevalence studies for DENV and CHIKV have examined exposures to each in isolation despite evidence *[21]* of co-circulation and shared risk factors *[6, 22, 23]*. There are no published serological surveys of dengue and chikungunya in Ecuador or studies of co-exposures to the two viruses from the Latin American region *[6]*, and limited information on the determinants of exposure risk. Such data are critical to estimate the burden of infection and inform public health strategies for control. The first licenced dengue vaccine (CYD-TDV) is now available in the Latin American region. Current WHO guidelines advise the implementation of cost-effective prevention strategies that consider local arbovirus epidemiology *[24]*. Recent studies indicated that targeting only seropositive individuals for vaccination might reduce global annual disease incidence by 20 to 30%, with the greatest impact in high-transmission settings *[25]*. Hence, understanding past exposure or seropositivity patterns in affected populations are key to implementing cost-effective control strategies.

In this study, we hypothesised that prevalence of past exposure to DENV and CHIKV in the population are associated with age, and individual and household risk factors. We estimated age-dependent seroprevalence of DENV, CHIKV and co-exposures with both arboviruses in a district in coastal Ecuador, and explored individual and household determinants of past exposure status.

For peer review only - http://bmjopen.bmj.com/site/about/guidelines.xhtml

Materials and methods

Population and study sample

The data consist of a representative sample of households including children (and other household members) recruited into the ECUAVIDA birth cohort between 2005 and 2009 in the town of Quininde, Esmeraldas Province [26]. The District is largely rural in a tropical region of coastal Ecuador with a population of 88,000 living below 150 m altitude with mean annual temperature of 30ºC and relative humidity of 80%. Main sources of income are cultivation of palm oil and tropical fruits, and timber extraction. Children (and all other members of the household to which they belonged) due to attend the cohort clinic in Quininde for routine follow up visits between October 2015 and February 2016 were invited to participate in this study according to the dates of their scheduled attendance. This approach resulted in the selection of a representative sample of ECUAVIDA cohort households (*Supplementary Table 1*). As is frequent for such ad-hoc surveys, post-stratification weightings [27] using the census population distributions were used to adjust the estimates to population-generalizable values (details in supplementary information and weights shown in *Supplementary Table 2.*

Venous blood was collected into heparinized tubes (Vaccutainer, BD Systems), centrifuged and plasma was stored at -20ºC. Data on individual and household factors were collected from the child's mother by an investigator-administered questionnaire and the 2010 census population data was used to derive post-stratification weights such that the resulting estimates would represent those of the population *[27]*. Each household was visited and location determined by GPS (Garmin eTrex, KS, USA).

For peer review only - http://bmjopen.bmj.com/site/about/guidelines.xhtml

Laboratory testing

Plasma was analysed for specific IgG antibodies to DENV and CHIKV viruses using commercial ELISA kits (Human anti-Dengue IgG and human anti-Chikungunya IgG ELISA kits, Abcam, Cambridge UK) following manufacturer's instructions. Past exposure or seropositivity to DENV and CHIKV was defined by the presence of specific IgG antibodies.

Statistical methods

The presence or absence of DENV and CHIKV antibodies define a bivariate binary outcome indicating past exposure to DENV only denoted by D+C-, to CHICK only denoted by D-C+, to both, D+C+, or to none. The key assumption of the model is that of long-lasting immunity *[19]* after infection with DENV and CHIKV.

We used two simultaneous multilevel logistic regression equations with shared random effects to model the presence/absence of antibodies for DENV or CHIKV or both. The basic idea of this model was developed initially in econometrics *[28]* and more recently in the context of two binary correlated outcomes indicating infections but using a distinct approach from the one used here *[29]*. Each equation modelled the presence of one arbovirus conditioned on the other, making the presence of DENV more or less likely in the presence of CHIKV (and vice versa). We explored the effects of age, sex, area of residence (urban vs. rural), socioeconomic level (grouped as low, medium, and high derived from tertiles of the first component of principal components analysis of 7 socio-economic variables as described in *[30]*), crowding (number of persons per sleeping room) and household dispersal (Euclidian distance to nearest household using GPS coordinates) on our bivariate binary outcome (dependent variable) defined above. The simplest model started with investigating the effect of age on the outcome which provided answers to the main question of interest, i.e. how the probabilities of past exposures to (D+C-, D-C+ and D+C+) varied with age in this population. The systematic trends with age in these probabilities are called *age-dependent prevalence of past exposure* to D+C-, D-C+ and D+C+.

For peer review only - http://bmjopen.bmj.com/site/about/guidelines.xhtml

They are also predicted probabilities of past exposures. We then refined these findings by exploring the extent to which these overall associations between past exposure to D+C-, D-C+ and D+C+ and age are altered by the other variables – alone and in a multivariable model.

The model (*Supplementary file*) also embedded post-stratification weights, allowing estimates to be generalizable to the population from which the sample was derived *[27]*. Hierarchical structure *[31]* of the data (individuals within families within wards) was represented by shared random effects, accounting for unmeasured epidemiological factors such as biting rates or climate conditions. Because of the assumption of long-life immunity, the age-dependent prevalence of past specific or co-exposures *[14]* can be also regarded as measures of immunity to these viruses in the population.

Bayesian inference was used for parameter estimation and to predict age-dependent probabilities of seropositivity and 95% credible intervals (CrI). In the absence of any formal goodness-of-fit measure, predicted age dependent prevalence was plotted against the observed proportions of positives within the same 5-year age group. Moreover, we also plotted the observed numbers of seropositive individuals across 5-year groupings and across geographical regions against expected numbers predicted by the model.

Detailed information on the statistical approach used with mathematical models, framework for statistical inference, estimated parameters and goodness of fit are provided as Supplementary files. Aggregated data at national and provincial levels for case reports of dengue/chikungunya from 2015 were retrieved from various sources *[16, 17, 32, 33]*. Given the assumption of life-long immunity, age-dependent seroprevalence and risk or probability of past exposures to DENV and CHICKV, are used interchangeably.

For peer review only - http://bmjopen.bmj.com/site/about/guidelines.xhtml

Results

Figure 1 shows the geographical locations of households of children in the background cohort and the locations of those sampled for this analysis (151). There was an almost 3-fold increase in the number of reported DENV cases (42,499 cases) in 2015 compared to 2014 (15,584) and 2016 (13,612). An outbreak of CHIKV of comparable size (34,101 cases) emerged in 2015 after no previously documented history of infection in the country before 2014: Esmeraldas province accounted for a third (10,477) of CHIKV cases in 2015 reported nationally (*Supplementary Table 3* and *Supplementary Figure 1* [showing monthly time-series of cases]). Subsequent (2016) Zika epidemic cumulative numbers are also presented for reference.

Plasma from blood samples collected between September and December 2015 from 319 individuals (including children) belonging to 151 households from 49 wards (electoral sub-divisions at parish level) were analysed. Characteristics for subjects and their households are provided in Table 1. Of 319 individuals, 245 (76.8%) were seropositive for DENV and 145 (45%) were seropositive for CHIKV. High numbers/proportions were positive for both arboviruses: 129 (40%) individuals belonging to 86 (57%) families living in 24 (49%) wards. Proportions of households and wards with at least one seropositive individual were: 143 (95%) households in 46 (94%) wards for DENV and 93 (62%) households in 27 (55%) wards for CHIKV.

The sub-sample of the cohort used in this analysis appeared to represent the overall cohort well in terms of socioeconomic status and area of residence *(Supplementary Table 1*). Distributions by parish and age for the census population of Esmeraldas Province, cohort, and serological sample are shown in *Supplementary Table 2* and *Supplementary Table 3*, respectively, along with stratum-specific weights subsequently used in modelling for population-level

representativeness. Parameter estimates (conditional odds ratios) which measure the univariate and adjusted associations of the risk of testing positive for DENV, CHIKV and both with each potential risk factor (age, sex, area of residence, socio-economic status and distance to next door neighbour) are shown in Table 2. Given the relative complexity of this non-linear modelling, the results are best understood through a predicted average seroprevalence across the population, with reference to trends and patterns by age and household characteristics (*Supplementary Table 4*).

Average overall DENV seroprevalence (including those seropositive for both viruses) was 84% (95% CrI 75%-91%) but increased rapidly with age from 14% (95% CrI 1.5-45%) in infants to 97% (95% CrI 93-99%) in adults aged 60 years (*Figure 2*). The overall average seroprevalence of CHIKV was 27% (95% CrI 8.7%-51.6%), peaked at 42% (95% CrI 18%-66%) in children aged 9 years, and declined to 18% (95% CrI (5%-38%) by 60 years (*Figure 2*).

Age-dependent prevalence curves for D+C-, D-C+, D+C+ and actual proportions from raw data (bars) for 5-year age groupings are shown *Figure 3*. The predicted curves fitted observed data well: raw proportions of positives were generally close to the 95% CrI predicted curves. Average seroprevalence of D+C- was 58% (95% CrI 38-74%), that of D-C+ was 1% (95% CrI 0-3%) and that of D+C+ was 26% (95% CrI 8-50%) (*Supplementary Table 5*). Seroprevalence increased rapidly following a power law (i.e. faster than exponential) with age for D+C-, declined rapidly for D-C+, and increased rapidly to a peak around 9 years (40%, 95% CrI 18%-64%) for D+C+, followed by a steady decline to 18% (95% CrI 6-37%) by 60 years.

Average seroprevalence by groups defined by co-exposures including sex, area of residence, and socioeconomic status are shown in *Supplementary Table 5* and their sole effects on age-specific prevalence in Table 2 and *Supplementary Figure 2.* Females were more likely to be seropositive for CHIKV (D+C+ and D-C+) but less likely to be singly positive to DENV

For peer review only - http://bmjopen.bmj.com/site/about/guidelines.xhtml

(D+C). The difference in D+C- seroprevalence between males and females was greatest in adolescence while that for D+C+ was greatest in childhood (*Supplementary Figure 3*). Individuals resident in rural areas were more likely to be D+C- while D+C+ was more frequent in urban areas; while shorter distance to nearest household (i.e. household dispersal) appeared to be a risk factor for D+C+.

Strength of associations were preserved in the multivariable model but estimates were less precise as more information was extracted, i.e. the CrIs were wider (Final Model in Table 2). Males remained more likely to be D+C- and less likely to be D+C+ (*Supplementary Figure 4*); individuals living in rural areas were more likely to be D+C- while those living in urban areas were more likely to be D+C+ (*Supplementary Figure 5*); and individuals of lower economic status appeared to be more likely to have all outcomes across all ages although differences were greater among those aged less than 20 years (*Supplementary Figure 6*). *Supplementary Figure 7* and *Supplementary Figure 8* show further evidence for a good fit of the model to the numbers in the data rather than to proportions: group-specific defined by age and geographical regions predicted number of seropositive individuals were derived by averaging the age-specific proportions in that group and used to predict approximate average number of exposed individuals. All the parameter estimates MCMC traces for the most complex model (Table 2) and their correlations are presented in *Supplementary Figure 9 Supplementary Figure 9* and *Supplementary Figure 10*, respectively. The estimated variance-covariance matrices corresponding to the random effects associated with both ward and family structures can also be viewed in *Supplementary Figure 11*.

Discussion

Arbovirus infections are a growing public health threat in Latin America where DENV is endemic in tropical and sub-tropical regions, and CHIKV and Zika virus infections have recently emerged *[6]*. Despite evidence for co-circulation within the same geographic regions

For peer review only - http://bmjopen.bmj.com/site/about/guidelines.xhtml

of more than one type of arbovirus, and in many regions all 3 viruses *[3, 9, 34]* that share the same vectors and cause similar symptomatology *[35]*, there are no studies addressing the epidemiology of co-exposures. Nevertheless, the epidemiological and statistical methodologies developed here can be made to accommodate relatively easily more than two concomitant exposures. Although DENV has been endemic in Ecuador since 1980, there are no previous seroprevalence estimates from Ecuador for either arbovirus *[6]*. Recent research in Machala in southern coastal Ecuador has highlighted the need for integrated surveillance [13] and predictions in the same region have been discussed in the context of El Niño Southern Oscillation climatic phenomena [36, 37].In the present study, we estimated that 84% (95%CrI 75%-91%) of individuals aged 0 to 60 years had been exposed to DENV in a tropical region of coastal Ecuador. However, DENV seroprevalence increased rapidly with age reaching 97% (95% CrI 93%-99%) by 60 years, reflecting cumulative exposures over a 30-year period since DENV re-emerged in the region in the late 1980s *[18]*. Placing this estimate within a global context, it concurs with a previous systematic review *[6]* indicating a high average seroprevalence in the Caribbean and Latin American regions (~65%) compared to Asia (46%) and Africa (18%).

Mean chikungunya seroprevalence was 27% (95% CrI 8.7%-51.6%) in this population and is within the range of seroprevalence estimates from seven previous studies from Latin America: Puerto Rico (23.5%), Guadeloupe (48.1%), Martinique (41.9%), Saint Martin (16.9%), Nicaragua (32.8%) and Brazil (20%) *[38]*. A study of two urban populations in Brazil estimated CHIKV seroprevalence at 57.1% and 45.7%, respectively *[38]* while seroprevalence in a rural area of Chapada district (Brazil) was estimated at 20% (95% CrI, 15.4–35%) *[39]*. A seroprevalence of CHIKV of 75% was observed in urban children in Haiti and French Polynesia *[6]*. Our data showed a much flatter seroprevalence pattern by age with a peak around 9 years, likely reflecting a single intense exposure period during 2015.

For peer review only - http://bmjopen.bmj.com/site/about/guidelines.xhtml

In the present analysis, we developed statistical methodology to address analysis of exposures to co-circulating viruses which share strong epidemiological similarities. This method was applied to a bivariate binary outcome defined by the presence/absence of specific IgG antibodies in sera from a population sample weighted to represent the source population, and aimed at understanding how patterns of age-dependent seroprevalence vary by individual and contextual factors. The model allowed prediction of 4 possible outcomes (i.e. D+C-, D-C+, D+C-, and D-C- [used as reference group]). D+C- was the most prevalent group in this population followed by D+C+ and D-C+ (*Figure 3*). The latter group was small and young with imprecise estimates. This statistical methodology show here can be applied relatively easily to more than two co-exposures in future studies.

Males were more likely to have been exposed to DENV while females had greater exposure to CHIKV, effects that were observed across all ages. Differences in exposure by sex could reflect temporal changes in transmission sources related to accelerating urbanisation in the context of women maintaining traditional roles as home keepers (i.e. transmission is largely within and between households, particularly in an urbanizing/urban setting). The observation that CHIKV seroprevalence was inversely associated with distance between households (e.g. a marker of greater population densities and urbanism) suggest that CHIKV exposures are more intense in crowded places, an observation not seen for DENV in this study. These data are consistent with an outbreak in 2012 in Bangladesh that showed a reduced probability of transmission of CHIKV with increasing distance between households *[40]*. An increased risk of infection among females from the same study was attributed to greater time spent around the house *[40]*. The primary vector, *Ae. aegypti*, is well adapted to urban living and breeds wherever clean water is present, particularly in open recipients around households.

The non-linear age-dependence of DENV seroprevalence is not unexpected given the period over which exposures are likely to have occurred. Our data indicate that by 60 years of age

For peer review only - http://bmjopen.bmj.com/site/about/guidelines.xhtml

almost everyone has been infected at least once with a dengue serotype. In contrast, exposure to CHIKV, which first appeared in Ecuador in 2014, possibly after a long absence *[7]*, was shorter: CHIKV caused a large outbreak in 2015 in coastal Ecuador, particularly in Esmeraldas Province. Thus, the age-seroprevalence profile for chikungunya, unlike dengue for which age can be viewed as a measure of length of exposure, may reflect age-dependent differences in exposures to *Aedes* vectors or susceptibility to infection.

Current global estimates of DENV and CHIKV prevalence are uncertain, being largely based on case reports to public health authorities often by other diagnostic mechanisms than laboratory analyses. Our data support the usefulness of serological surveys to inform evaluations of burden of infection within countries, particularly those with limited health resources. Further, local serological surveys will be useful for planning novel population-level interventions against arbovirus transmission such as vaccination. Dengvaxia is only currently registered dengue vaccine that might be used in endemic populations *[24]* and it is not licensed for use in Ecuador. There has been extensive debate over dengue vaccination planning in the endemic countries: either vaccination of populations with dengue seroprevalence rates above 80% or screening for dengue-specific antibodies and vaccinating only seropositive individuals. The WHO scientific advisory working group concluded that both "mass vaccination based on population seroprevalence criteria" and "pre-vaccination screening" are difficult to implement and neither can achieve herd immunity but favours a pre-vaccination strategy in which only seropositive persons are vaccinated. A better understanding of the local epidemiology of dengue will be essential for the evaluation of cost-effectiveness of such measures *[24]*. Recent research suggested that highlighting areas of high prevalence is important as recent analyses suggested that transmission-blocking interventions such as Wolbachia, even at intermediate efficacy (50% transmission reduction), might reduce global annual disease incidence by up to 90%. The newly licensed Sanofi-

For peer review only - http://bmjopen.bmj.com/site/about/guidelines.xhtml

Pasteur vaccine, targeting only seropositive recipients, (namely those at risk of secondary infection which can result in severe dengue and subsequent death), might reduce global annual disease incidence by 20 to 30%, with the greatest impact in high-transmission settings [25].

Limitations of seroprevalence estimates from the literature are small sample size and poorly representative samples *[6]*. A recent review of seroprevalence studies for DENV, CHIKV, and Zika identified 185 studies of which the majority were specific populations or age sub-groups (60%), and just over a third (38.9%) were samples from the general population *[6]* although few were representative samples. About half (50.3%) of the 185 studies had sample sizes of less than 500 subjects *[6]*. The sample size in this study likely limited our ability to detect effects of individual and contextual factors. We have minimized the potential bias in using a sample from a birth cohort rather than a general population sample, by assigning post-stratification weights based on the 2010 census *[27]*.

Our modelling strategy was based on assumptions that: 1) mortality caused by either infection is negligible- case fatality rate in the America region for dengue is approximately 2.5% annually while that of chikungunya is less than 0.1% *[41, 42]*; and 2) both arboviruses induce long-lasting immunity *[43]* - while likely to be true for CHIKV, relevance to DENV is less clear given that immunity is strain-specific and infections with 4 different strains are possible *[44]*. Molecular analysis of dengue strains in Ecuador have shown all 4 strains to be circulating since before 2013 *[45]*. Further, our data can shed little light on vector competence which may affect the transmission of one or other arbovirus (or DENV serotype/genotype) if the two are circulating in the same population at the same point in time – there is some evidence for adaptation of CHIKV to *A. aegypti* and for displacement of one arbovirus by another within populations of *A. aegypti [46]*. Seroprevalence estimates here likely reflect cumulative exposures to all four DENV strains. Given relatively low case

For peer review only - http://bmjopen.bmj.com/site/about/guidelines.xhtml

fatality rates of 2.5% for DENV (mostly related to severe dengue) and 0.1% for CHIKV *[43, 47]*, this assumption is reasonable. Although the serological tests used were highly sensitive, sensitivity may vary depending on whether infection is primary or secondary *[48]*. In the context of the present study, there was limited evidence of reduced sensitivity in older subjects (more likely to have secondary infections) among whom seroprevalence to DENV reached almost 100% by 60 years. The serological test used for CHIKV and DENV were highly specific: the study was done before the emergence of Zika virus which first appeared in Ecuador in 2016 and in the study area in May 2016 with which serological tests for DENV share significant serological cross-reactivity [49].

Conclusions

Our data, from a household sample weighted to represent the general population, showed strong age-dependence of DENV seroprevalence but not CHIKV which emerged only in 2015 in the study population in a tropical region of coastal Ecuador. Our study will inform policy makers on relevant control strategies in Ecuador and decisions on cost–effectiveness of vaccination strategies for dengue *[24]*. Highlighting areas of high prevalence is important as recent analyses suggested that transmission-blocking interventions such as *Wolbachia*, even at intermediate efficacy (50% transmission reduction), might reduce global annual disease incidence by up to 90% *[25]*. The newly licensed Sanofi-Pasteur vaccine, targeting only *seropositive* recipients, (namely those at risk of secondary infection which can result in severe dengue and subsequent death), might reduce global annual disease incidence by 20 to 30%, with the greatest impact in high-transmission settings *[25]*.

For peer review only - http://bmjopen.bmj.com/site/about/guidelines.xhtml

MODEL	DENGUE CONDITIONED ON CHIKUNGUNYA MEAN	SD	MEDIAN	P025	P975	P(OR<=1)/P(OR>1)	CHIKUNGUNYA CONDITIONED ON DENGUE MEAN	SD	MEDIAN	P025	P975	P(OR<=1)/P(OR>1)
1st MODELS												
AGE ((POWER*)	7.671	3.123	7.023	3.616	15.450		0.357	0.097	0.346	0.202	0.579	
2nd MODELS												

Tables

Characteristic	N (%)
**Individual characteristics**	**N=319**
Sex	
Male	117(37%)
Female	202(63%)
Age (years)	
0-5	7(2%)
6-10	140(44%)
11-15	4(1%)
16-20	3(1%)
21-25	12(4%)
26-30	45(14%)
31-35	37(12%)
36-40	41(13%)
41-45	15(5%)
46-50	9(3%)
>50	6(2%)
Dengue seropositive	
No	74 (32.2%)
Yes	245 (76.8%)
Chikungunya seropositive	
No	174 (54.5%)
Yes	145 (45.5%)
**Household characteristics**	**N=151**
Area of residence	
Rural	47 (32%)
Urban	104 (68%)
Socioeconomic status	
Low and medium	106 (70%)
High	45 (30%)
Household dispersal (metres)	
<50	13 (8.6%)
51-250	63(41.7%)
251-500	34(22.5%)
501-1,000	12(8%)
>1,000	29(19%)

Table 1. Characteristics of 319 individuals from 151 households in the region of the District of Quininde, Esmeraldas province.

For peer review only

For peer review only - http://bmjopen.bmj.com/site/about/guidelines.xhtml

FEMALE vs. MALE	0.728	0.373	0.650	0.248	1.665	**0.822/0.178**	2.971	1.587	2.614	1.030	7.004	**0.017/0.983**
RURAL vs. URBAN	1.490	1.431	1.099	0.260	5.020	0.441/0.559	0.252	0.337	0.159	0.023	1.040	**0.975/0.025**
CROWDING	1.217	0.744	1.042	0.348	3.118	0.476/0.524	0.916	0.759	0.718	0.179	2.808	0.679/0.321
LOW vs. HIGH	3.302	2.472	2.671	0.828	9.518	**0.052/0.948**	0.916	0.858	0.675	0.142	3.148	0.684/0.316
HOUSEHOLD	0.825	0.314	0.772	0.380	1.585	0.771/0.229	0.711	0.384	0.628	0.225	1.685	**0.826/0.174**
FINAL MODELS												
AGE (POWER)	9.980	4.623	8.955	4.290	21.760		0.263	0.085	0.251	0.132	0.460	
FEMALE vs. MALE	0.651	0.347	0.577	0.210	1.528	**0.87/0.13**	3.804	2.356	3.238	1.152	9.831	**0.01/0.99**
RURAL vs. URBAN							0.218	0.479	0.099	0.008	1.167	**0.98/0.02**
LOW vs. HIGH	3.028	1.992	2.536	0.827	8.085	**0.05/0.95**						
HOUSEHOLD							0.794	0.561	0.657	0.186	2.202	0.76/0.24

Table 2. Parameter estimates derived after fitting simultaneous logistic models with increasing number of explanatory variables. 1st Models have age only as predictor. 2nd Models show effects of each of age, sex, area of residence, socio-economic status, household overcrowding and household dispersal on the Odds Ratio of past exposure vs. no exposure to the viruses. Final models included all variables for which the effect in the simpler models, measured as P(OR>1) (or P(OR<1)), was greater than 0.80 or less than 0.20 (shown in bold). Age-dependence is parameterized according to a power law (i.e. age^{power}) and the corresponding estimate represented the *power* of the age^{power} component.

For peer review only

Supplementary Tables

Supplementary Table 1. Comparison between serological sample and cohort with respect to area of residence and socioeconomic status of households.

Supplementary Table 2. Geographical distribution of households by parish across region of Esmeraldas (census), cohort and data sample. The data sample distribution is similar to that of the cohort and the post-stratification weights adjusted for a fair representation of the Esmeraldas Province. The weights represent essentially the ratios between the census proportions and data proportions across each region. This calibration also adjusted for urban/rural area of residence.

Supplementary Table 3. Total number of reported cases of dengue and chikungunya in Ecuador and Esmeraldas province in 2015 compared to the 2014 and 2016. Overall proportions of cases were calculated using Ecuador and Esmeraldas population total as denominator, respectively. Ecuador census data for 2010 were used and the totals were adjusted according to the annual predicted population growth.

Supplementary Table 4. Age distributions of Esmeraldas census population and serological sample from cohort. The weights used in modelling were the ratios between Esmeraldas census population distribution proportions and age-group proportions in the serological sample.

Supplementary Table 5 Average predicted seroprevalence (proportions) for each exposure category and their 95%Credible Intervals (CrI) for dengue and chikungunya and seroprevalence difference by sex (female [F] vs. males [M]), area of residence (urban [U] vs. rural [R]), and socioeconomic level (low [L] vs. high [H]). Exposure categories are: DENV+CHIKV- [D+C-]; DENV-CHIKV+ [D-C+]; and DENV+CHIKV+ [D+C+].

Figures

For peer review only - http://bmjopen.bmj.com/site/about/guidelines.xhtml

Figure 1. Geographic distribution of the household locations of the background population (ECUAVIDA) and the serological sample.

Figure 2. Predicted age-dependent seroprevalence for dengue and chikungunya. Dashed lines represent the 95% Credible Intervals and the bars represent the observe proportions of seropositive participants.

Figure 3. Predicted age-dependent prevalence for exposure categories to dengue and chikungunya in the sample population plotted against observed proportions (in 5-year age groupings). Dashed lines represent 95% Credible Intervals.

Supplementary Figure 1. Reported clinical cases of dengue and chikungunya in 2015 in Ecuador nationally and in the Province of Esmeraldas. Time-series graphs were derived from data publicly accessible (17, 18, 19, 20)

Supplementary Figure 2. Predicted age-dependent seroprevalence for exposure groups to dengue and chikungunya for categories of area of residence, sex, and household dispersal in study sample. Exposure categories are: DENV+CHIKV- [D+C-]; DENV-CHIKV+ [D-C+]; and DENV+CHIKV+ [D+C+]. Dashed lines represent the 95% Credible Intervals.

Supplementary Figure 3. Predicted age-dependent differences for exposure categories by sex (A), area of residence (B), and socioeconomic status (C). Exposure categories were

For peer review only

DENV+CHIKV- [D+C-]; DENV-CHIKV+ [D-C+]; and DENV+CHIKV+ [D+C+]. Dashed lines represent 95% Credible Intervals.

Supplementary Figure 4 Age-dependent seroprevalence for exposure groups to dengue and chikungunya stratified by sex, socioeconomic status, and area of residence. Exposure categories were DENV+CHIKV- [D+C-]; DENV-CHIKV+ [D-C+]; and DENV+CHIKV+ [D+C+]. Dashed lines represent the 95% Credible Intervals of the predictions derived from the multivariable model

Supplementary Figure 5. Age-dependent seroprevalence for exposure groups to dengue and chikungunya stratified by area of residence, sex, and socioeconomic status. Exposure categories were DENV+CHIKV- [D+C-]; DENV-CHIKV+ [D-C+]; and DENV+CHIKV+ [D+C+]. Dashed lines represent the 95% Credible Intervals of the predictions derived from the multivariable model (Table 2).

Supplementary Figure 6. Age-dependent seroprevalence for exposure groups to dengue and chikungunya stratified by socioeconomic status, sex, and area of residence. Exposure categories were DENV+CHIKV- [D+C-]; DENV-CHIKV+ [D-C+]; and DENV+CHIKV+ [D+C+]. Dashed lines represent the 95% Credible Intervals of the predictions derived from the multivariable model (Table 2).

Supplementary Figure 7. Model goodness of fit: the observed seropositive numbers in each 5-year age group vs. the expected numbers calculated using average age-specific MCMC

For peer review only - http://bmjopen.bmj.com/site/about/guidelines.xhtml

predictions and the total number in the corresponding age group: (a) for D+C-, (b) for D-C+ and (c) for D+C+.

Supplementary Figure 8 Model goodness of fit: the observed seropositive numbers in groups defined by geographical regions vs. the corresponding expected numbers calculated using MCMC predicted prevalence in that group and its total numbers: (a) for all DENV and (b) for all CHIKV.

Supplementary Figure 9. Parameter estimates for the most complex models - the MCMC traces for 100000 iterations.

Supplementary Figure 10. Parameter correlations based on the MCMC traces for 100000 iterations.

Supplementary Figure 11 Variance-covariance components (random effects) estimation associated with hierarchical structure of the data (family and ward).

For peer review only

Financial support

The project was supported by the Wellcome Trust (088862/Z/09/Z) and the Universidad Internacional del Ecuador.

Data sharing statement

The data were from a longitudinal birth cohort study and are available upon request to pcooper@sgul.ac.uk

Patient and Public Involvement statement

Informed written consent and minor assent was obtained to collect data and samples from study participants. At the time of consent, the public health benefits of the study were explained to participants.

For peer review only

References

1. Torres, M.C., et al., *Re-introduction of dengue virus serotype 2 in the state of Rio de Janeiro after almost a decade of epidemiological silence.* PLOS ONE, 2019. **14**(12): p. e0225879.
2. Brathwaite Dick, O., et al., *The History of Dengue Outbreaks in the Americas.* The American Journal of Tropical Medicine and Hygiene, 2012. **87**(4): p. 584-593.
3. Ding, F., et al., *Mapping the spatial distribution of Aedes aegypti and Aedes albopictus.* Acta Tropica, 2018. **178**: p. 155-162.
4. Kraemer, M.U.G., et al., *The global distribution of the arbovirus vectors Aedes aegypti and Ae. albopictus.* eLife, 2015. **4**: p. e08347.
5. Brady, O.J., et al., *Refining the Global Spatial Limits of Dengue Virus Transmission by Evidence-Based Consensus.* PLOS Neglected Tropical Diseases, 2012. **6**(8): p. e1760.
6. Fritzell, C., et al., *Current challenges and implications for dengue, chikungunya and Zika seroprevalence studies worldwide: A scoping review.* PLoS Neglected Tropical Diseases, 2018. **12**(7).
7. Scott, B.H., *Reappearance of Chikungunya, Formerly Called Dengue, in the Americas.* Emerging Infectious Disease journal, 2015. **21**(4): p. 561.
8. Yactayo, S., et al., *Epidemiology of Chikungunya in the Americas.* The Journal of infectious diseases, 2016. **214**(suppl 5): p. S441-S445.
9. Wahid, B., et al., *Global expansion of chikungunya virus: mapping the 64-year history.* International Journal of Infectious Diseases, 2017. **58**: p. 69-76.
10. Romero-Sandoval, N., et al., *Critical analysis of the definition of severe dengue, based on the Ecuador outbreak in 2012*. Vol. 04. 2014. 36-38.
11. Balmaseda, A., et al., *Trends in patterns of dengue transmission over four years of a pediatric cohort study in Nicaragua.* The Journal of infectious diseases, 2010. **201**(1): p. 5-14.
12. Morrison, A.C., et al., *Epidemiology of Dengue Virus in Iquitos, Peru 1999 to 2005: Interepidemic and Epidemic Patterns of Transmission.* PLOS Neglected Tropical Diseases, 2010. **4**(5): p. e670.
13. Vitale, M., et al., *A comparison of passive surveillance and active cluster-based surveillance for dengue fever in southern coastal Ecuador.* BMC Public Health, 2020. **20**(1): p. 1065.
14. Chis Ster, I., *Inference for serological surveys investigating past exposures to infections resulting in long-lasting immunity – an approach using finite mixture models with concomitant information.* Journal of Applied Statistics, 2012. **39**(11): p. 2523-2542.
15. CDC. *International Notes Dengue Epidemic -- Ecuador*. 1988; Available from: https://www.cdc.gov/mmwr/preview/mmwrhtml/00001411.htm.
16. PÚBLICA, S.N.D.V.-M.D.S. *Dengue in Ecuador*. Available from: https://www.salud.gob.ec/wp-content/uploads/2013/02/Gecata-Dengue-SE-15.pdf.
17. PÚBLICA, S.N.D.V.-M.D.S. *Chickungunya in Ecuador*. Available from: https://www.salud.gob.ec/wp-content/uploads/2017/07/GACETA-VECTORES-SE-1.pdf.
18. Stewart-Ibarra, A.M., et al., *The high burden of dengue and chikungunya in southern coastal Ecuador: Epidemiology, clinical presentation, and phylogenetics from a prospective study in Machala in 2014 and 2015.* bioRxiv, 2017.
19. Imai, N., et al., *Estimating Dengue Transmission Intensity from Sero-Prevalence Surveys in Multiple Countries.* PLOS Neglected Tropical Diseases, 2015. **9**(4): p. e0003719.
20. Rodriguez-Barraquer, I., H. Salje, and D.A. Cummings, *Opportunities for improved surveillance and control of dengue from age-specific case data.* eLife, 2019. **8**: p. e45474.
21. Paixão, E.S., M.G. Teixeira, and L.C. Rodrigues, *Zika, chikungunya and dengue: the causes and threats of new and re-emerging arboviral diseases.* BMJ Global Health, 2018. **3**(Suppl 1): p. e000530.
22. Beltrán-Silva, S.L., et al., *Clinical and differential diagnosis: Dengue, chikungunya and Zika.* Revista Médica del Hospital General de México, 2016.

For peer review only - http://bmjopen.bmj.com/site/about/guidelines.xhtml

23. Rodríguez-Barraquer, I., et al., *The Hidden Burden of Dengue and Chikungunya in Chennai, India.* PLoS Negl Trop Dis, 2015. **9**(7): p. e0003906.
24. Wilder-Smith, A., et al., *Deliberations of the Strategic Advisory Group of Experts on Immunization on the use of CYD-TDV dengue vaccine.* The Lancet Infectious Diseases, 2019. **19**(1): p. e31-e38.
25. Cattarino, L., et al., *Mapping global variation in dengue transmission intensity.* Science Translational Medicine, 2020. **12**(528): p. eaax4144.
26. Cooper, P.J., et al., *Cohort Profile: The Ecuador Life (ECUAVIDA) study in Esmeraldas Province, Ecuador.* International Journal of Epidemiology, 2015. **44**(5): p. 1517-1527.
27. Little, R.J.A., *Post-Stratification: A Modeler's Perspective.* Journal of the American Statistical Association, 1993. **88**(423): p. 1001-1012.
28. Schmidt, P. and R.P. Strauss, *Estimation of Models with Jointly Dependent Qualitative Variables: A Simultaneous Logit Approach.* Econometrica, 1975. **43**(4): p. 745-755.
29. Ghebremichael, M., *Joint modeling of correlated binary outcomes: HIV-1 and HSV-2 co-infection.* Journal of Applied Statistics, 2015. **42**(10): p. 2180-2191.
30. Menzies, S.K., et al., *Risk factors for soil-transmitted helminth infections during the first 3 years of life in the tropics; findings from a birth cohort.* PLoS Negl Trop Dis, 2014. **8**(2): p. e2718.
31. Goldstein, H., *Multilevel Statistical Models*. 4th Edition ed. 2011: JohnWiley and Sons, Ltd, Chichester.
32. PÚBLICA, S.N.D.V.-M.D.S. *Dengue in Ecuador - Esmeraldas*. Available from: https://www.salud.gob.ec/wp-content/uploads/2013/02/DENGUE-GACETA-SE28.pdf.
33. PÚBLICA, S.N.D.V.-M.D.S. *Chikungunya in Ecuador - Esmeraldas*. Available from: https://www.salud.gob.ec/wp-content/uploads/2014/09/GACETA-CHIKUNGUNYA-SE-52.pdf.
34. Leta, S., et al., *Global risk mapping for major diseases transmitted by Aedes aegypti and Aedes albopictus.* International journal of infectious diseases : IJID : official publication of the International Society for Infectious Diseases, 2018. **67**: p. 25-35.
35. Mardekian, S.K. and A.L. Roberts, *Diagnostic Options and Challenges for Dengue and Chikungunya Viruses.* BioMed research international, 2015. **2015**: p. 834371-834371.
36. Petrova D, R.X., Sippy R, Ballester J, Mejia R, Beltran-Ayala E, Borbor-Cordova MJ, Vallejo GM, Olmedo AA, Stewart-Ibarra AM, Lowe R. , *he 2018-2019 weak El Niño: predicting the risk of a dengue outbreak in Machala, Ecuador.* International Journal of Climatology, 2020. **In Press**.
37. Petrova, D., et al., *Sensitivity of large dengue epidemics in Ecuador to long-lead predictions of El Niño.* Climate Services, 2019. **15**: p. 100096.
38. Dias, J.P., et al., *Seroprevalence of Chikungunya Virus after Its Emergence in Brazil.* Emerging infectious diseases, 2018. **24**(4): p. 617-624.
39. Cunha, R.V., et al., *Seroprevalence of Chikungunya Virus in a Rural Community in Brazil.* PLOS Neglected Tropical Diseases, 2017. **11**(1): p. e0005319.
40. Salje, H., et al., *How social structures, space, and behaviors shape the spread of infectious diseases using chikungunya as a case study.* Proc Natl Acad Sci U S A, 2016. **113**(47): p. 13420-13425.
41. WHO. *Dengue and severe dengue*. Available from: https://www.who.int/news-room/fact-sheets/detail/dengue-and-severe-dengue.
42. WHO. *Chikungunya*. Available from: https://www.who.int/news-room/fact-sheets/detail/chikungunya.
43. Torres, J.R., et al., *Epidemiological Characteristics of Dengue Disease in Latin America and in the Caribbean: A Systematic Review of the Literature.* Journal of tropical medicine, 2017. **2017**: p. 8045435-8045435.

For peer review only - http://bmjopen.bmj.com/site/about/guidelines.xhtml

44. Andraud, M., et al., *Dynamic Epidemiological Models for Dengue Transmission: A Systematic Review of Structural Approaches.* PLOS ONE, 2012. **7**(11): p. e49085.
45. Ramos-Castañeda, J., et al., *Dengue in Latin America: Systematic Review of Molecular Epidemiological Trends.* PLoS neglected tropical diseases, 2017. **11**(1): p. e0005224-e0005224.
46. Souza-Neto, J.A., J.R. Powell, and M. Bonizzoni, *Aedes aegypti vector competence studies: A review.* Infection, Genetics and Evolution, 2019. **67**: p. 191-209.
47. Moraes, G.H., E. de Fátima Duarte, and E.C. Duarte, *Determinants of mortality from severe dengue in Brazil: a population-based case-control study.* The American journal of tropical medicine and hygiene, 2013. **88**(4): p. 670-676.
48. Simmons, C.P., et al., *Dengue.* New England Journal of Medicine, 2012. **366**(15): p. 1423-1432.
49. Priyamvada, L., et al., *Human antibody responses after dengue virus infection are highly cross-reactive to Zika virus.* Proc Natl Acad Sci U S A, 2016. **113**(28): p. 7852-7.

For peer review only

For peer review only - http://bmjopen.bmj.com/site/about/guidelines.xhtml

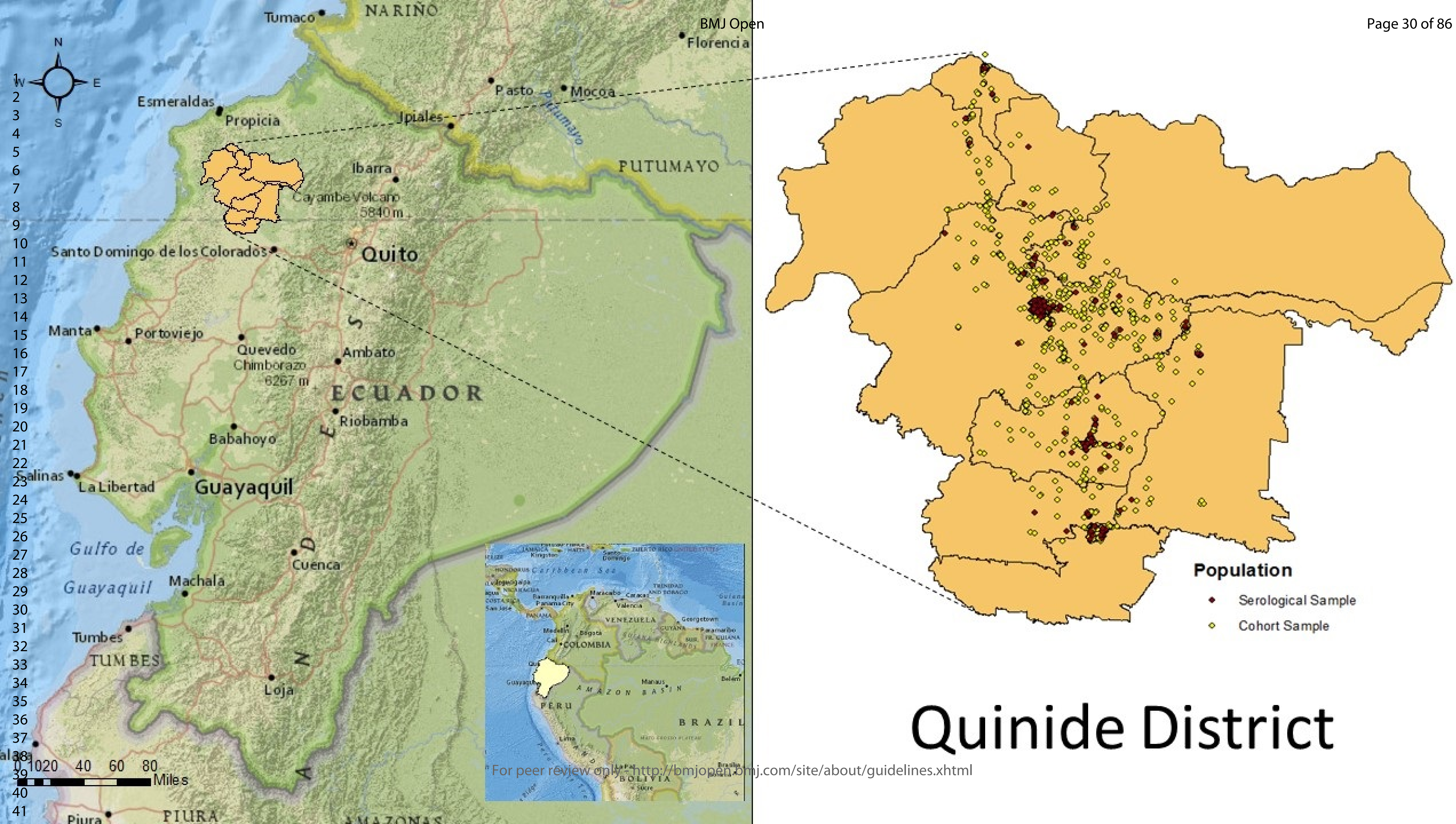

For peer review only - http://bmjopen.bmj.com/site/about/guidelines.xhtml

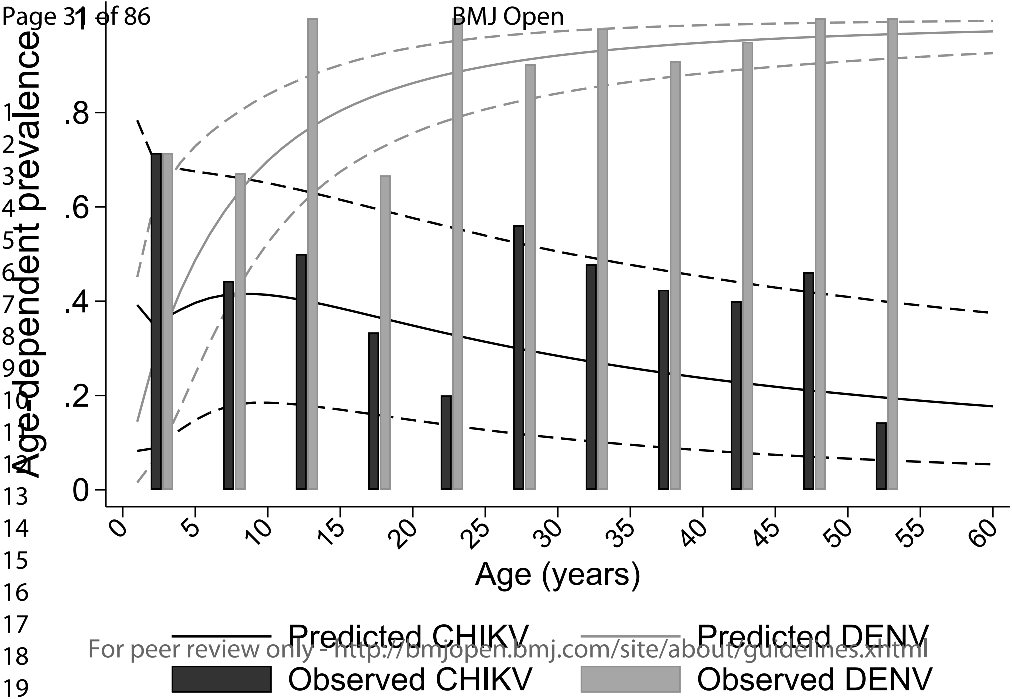

For peer review only - http://bmjopen.bmj.com/site/about/guidelines.xhtml

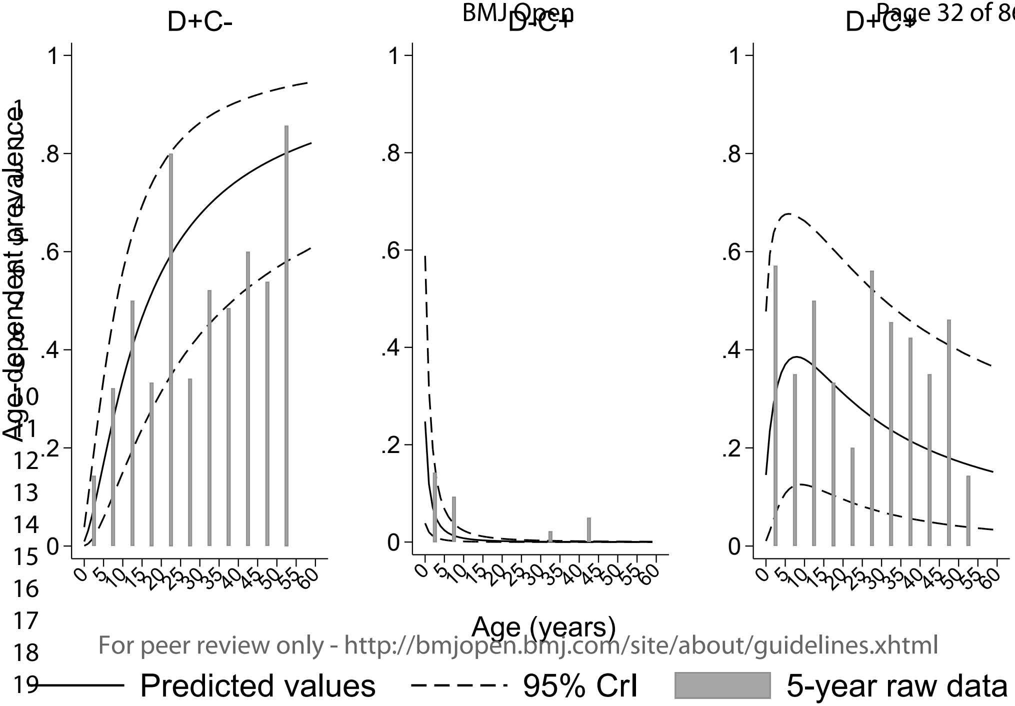
D+C-
D-C+
D+C+
Age-dependent prevalence
1
.8
.6
.4
.2
0
0 5 10 15 20 25 30 35 40 45 50 55 60
Age (years)
Predicted values
95% CrI
5-year raw data

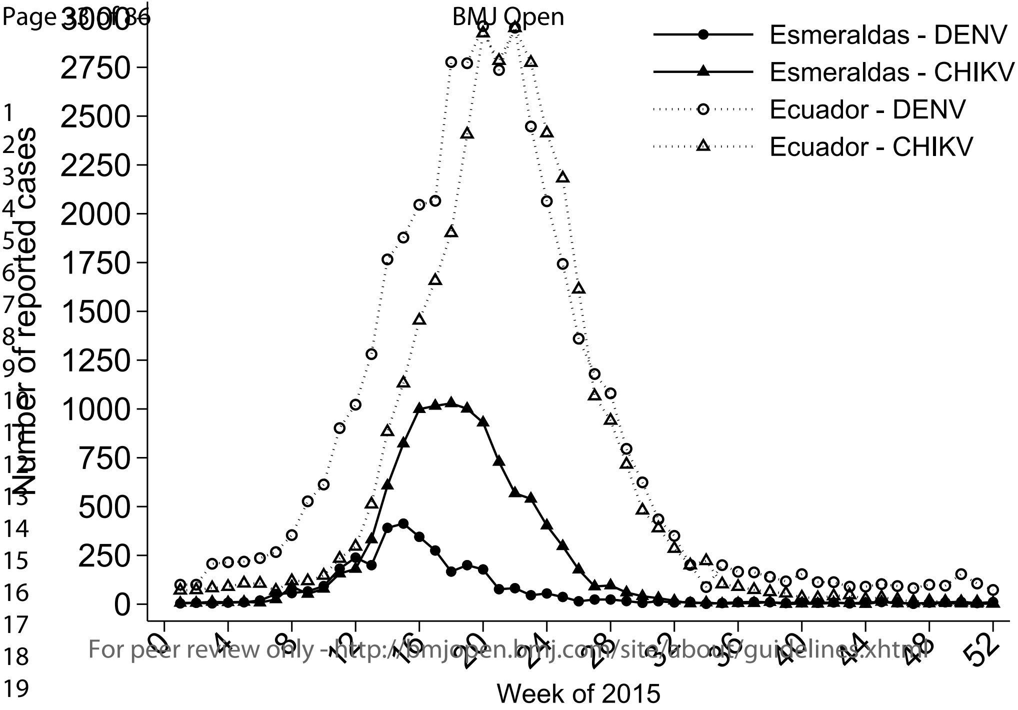

For peer review only - http://bmjopen.bmj.com/site/about/guidelines.xhtml

1
2
3
4
5
6
7
8
9
10
11
12
13
14
15
16
17

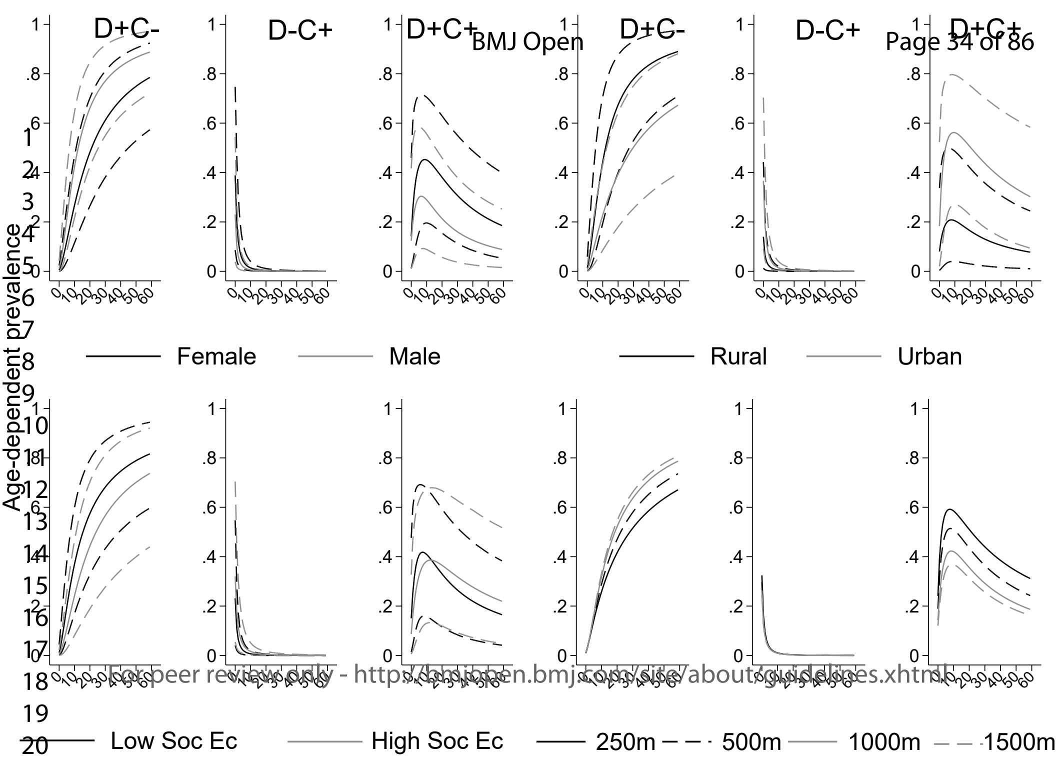

For peer review only - http://bmjopen.bmj.com/site/about/guidelines.xhtml

18
19
20

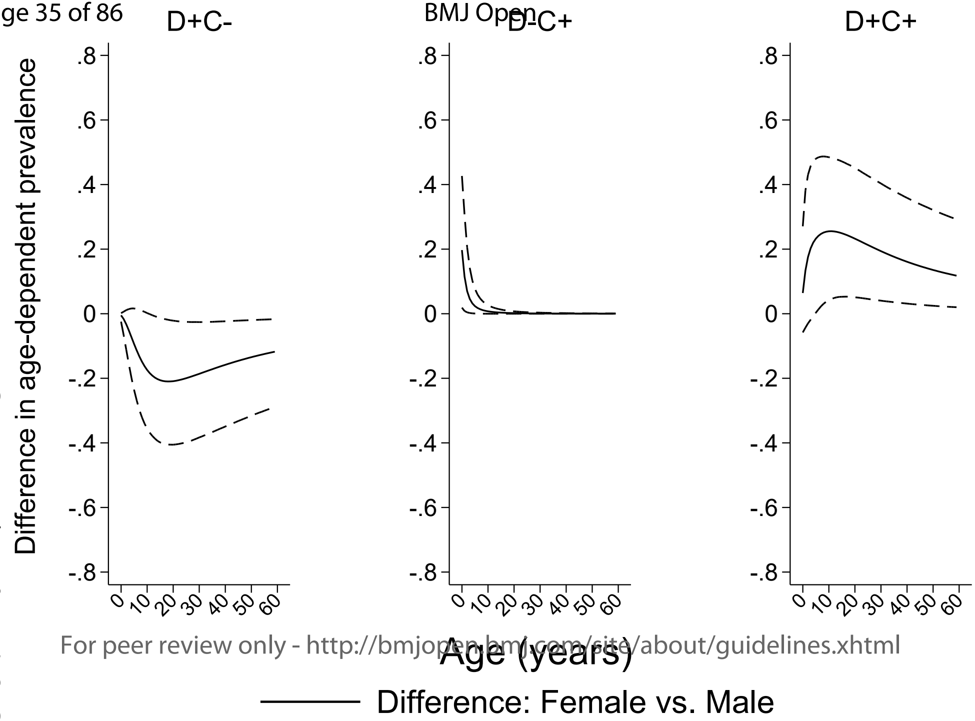

For peer review only - http://bmjopen.bmj.com/site/about/guidelines.xhtml

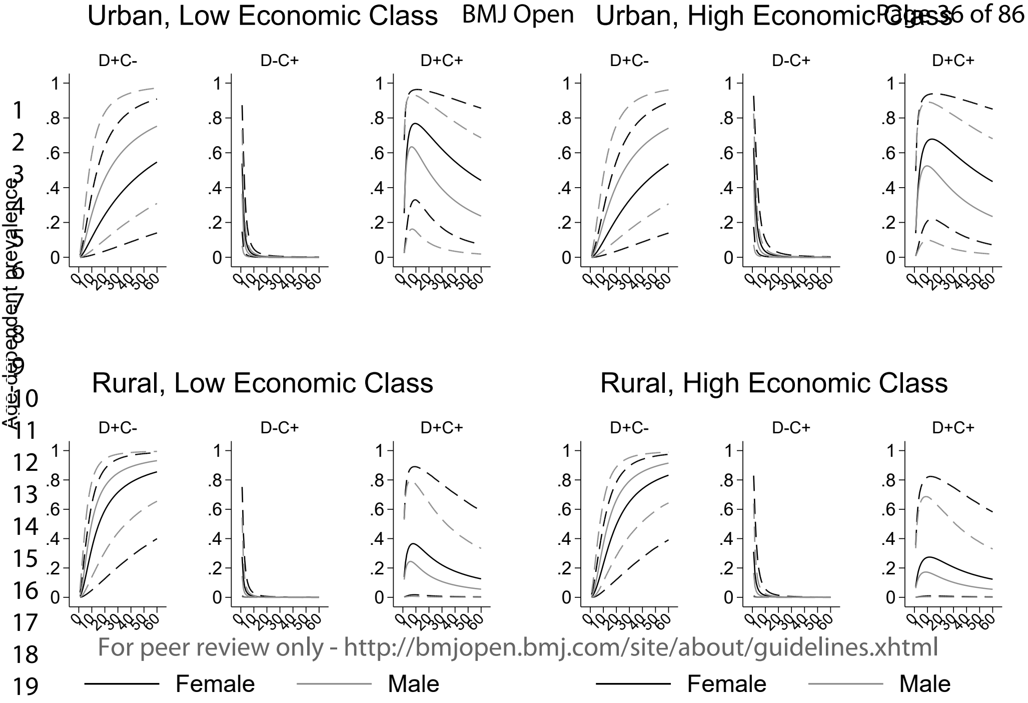

For peer review only - http://bmjopen.bmj.com/site/about/guidelines.xhtml

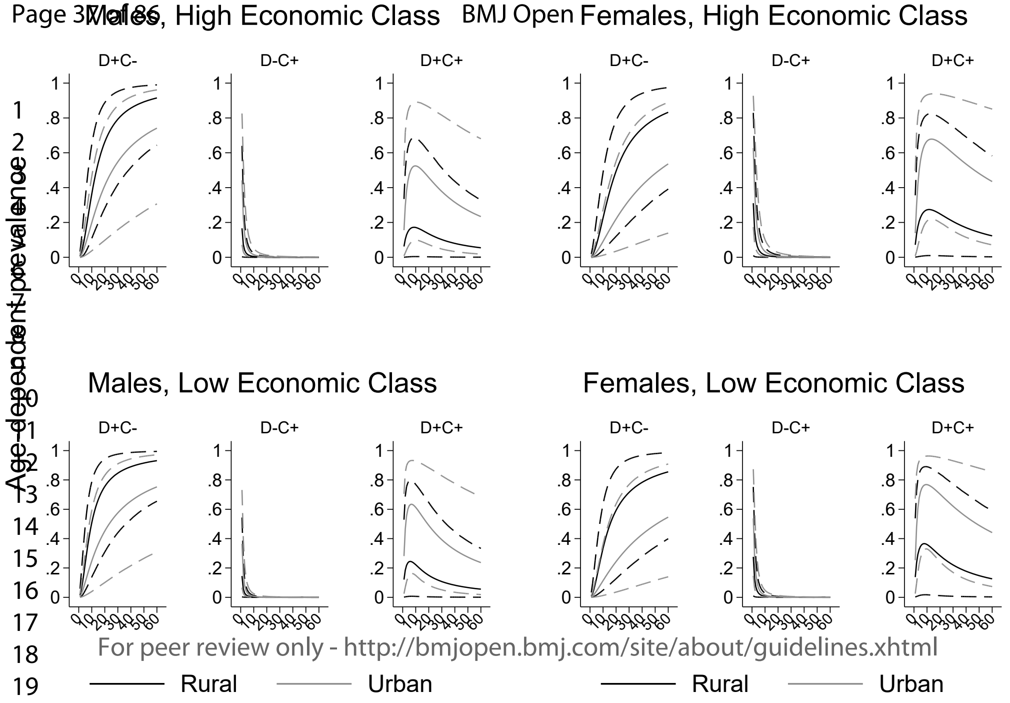
Males, High Economic Class
Females, High Economic Class
Males, Low Economic Class
Females, Low Economic Class
D+C-
D-C+
D+C+
Age-dependent prevalence
0
10
20
30
40
50
60
0
.2
.4
.6
.8
1
Rural
Urban

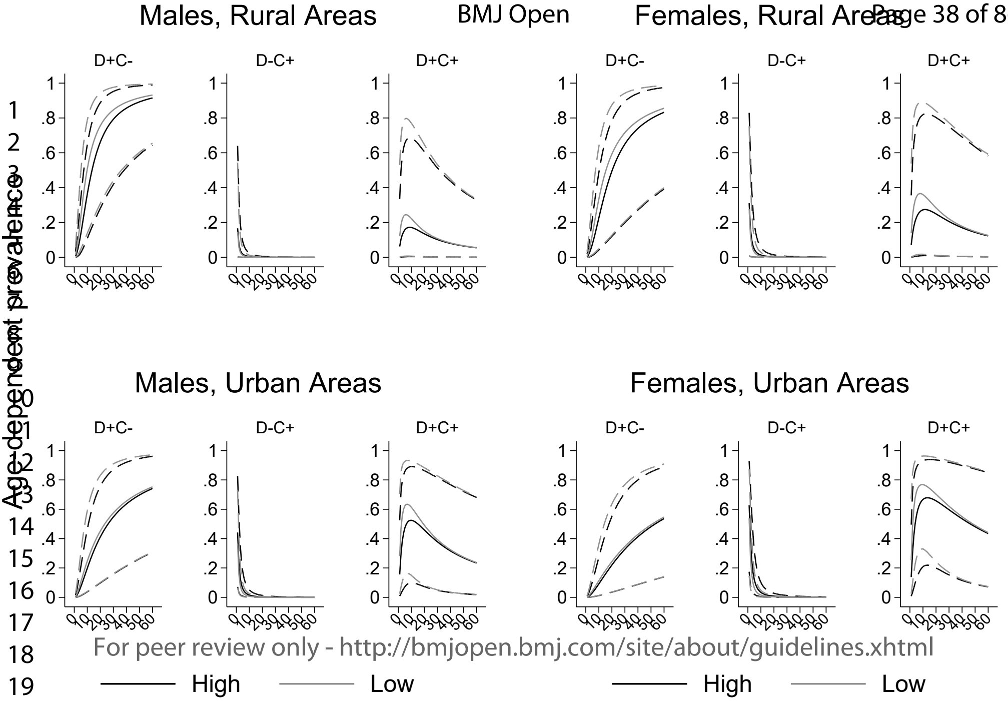

For peer review only - http://bmjopen.bmj.com/site/about/guidelines.xhtml

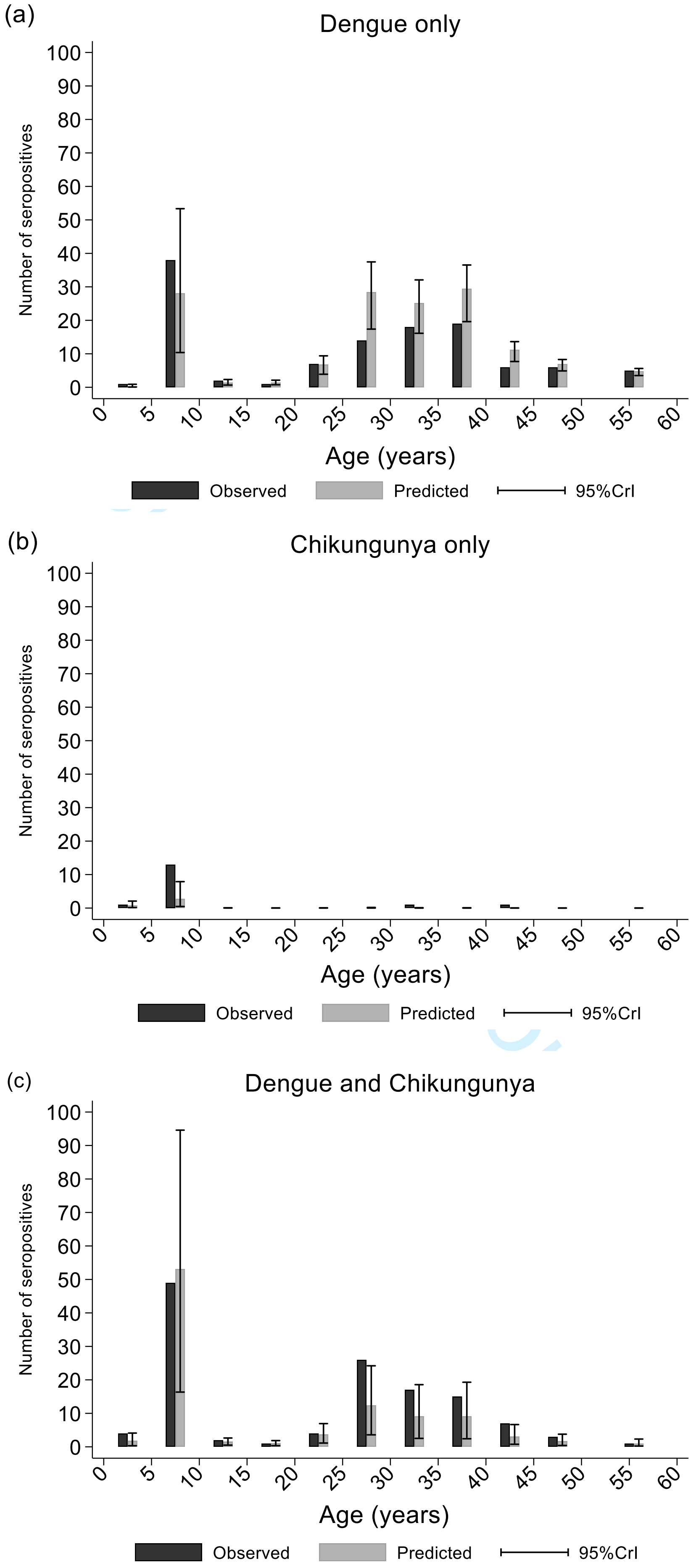
(a)
Dengue only
Number of seropositives
0 10 20 30 40 50 60 70 80 90 100
0 5 10 15 20 25 30 35 40 45 50 55 60
Age (years)
Observed
Predicted
95%CrI
(b)
Chikungunya only
Number of seropositives
0 10 20 30 40 50 60 70 80 90 100
0 5 10 15 20 25 30 35 40 45 50 55 60
Age (years)
Observed
Predicted
95%CrI
(c)
Dengue and Chikungunya
Number of seropositives
0 10 20 30 40 50 60 70 80 90 100
0 5 10 15 20 25 30 35 40 45 50 55 60
Age (years)
Observed
Predicted
95%CrI

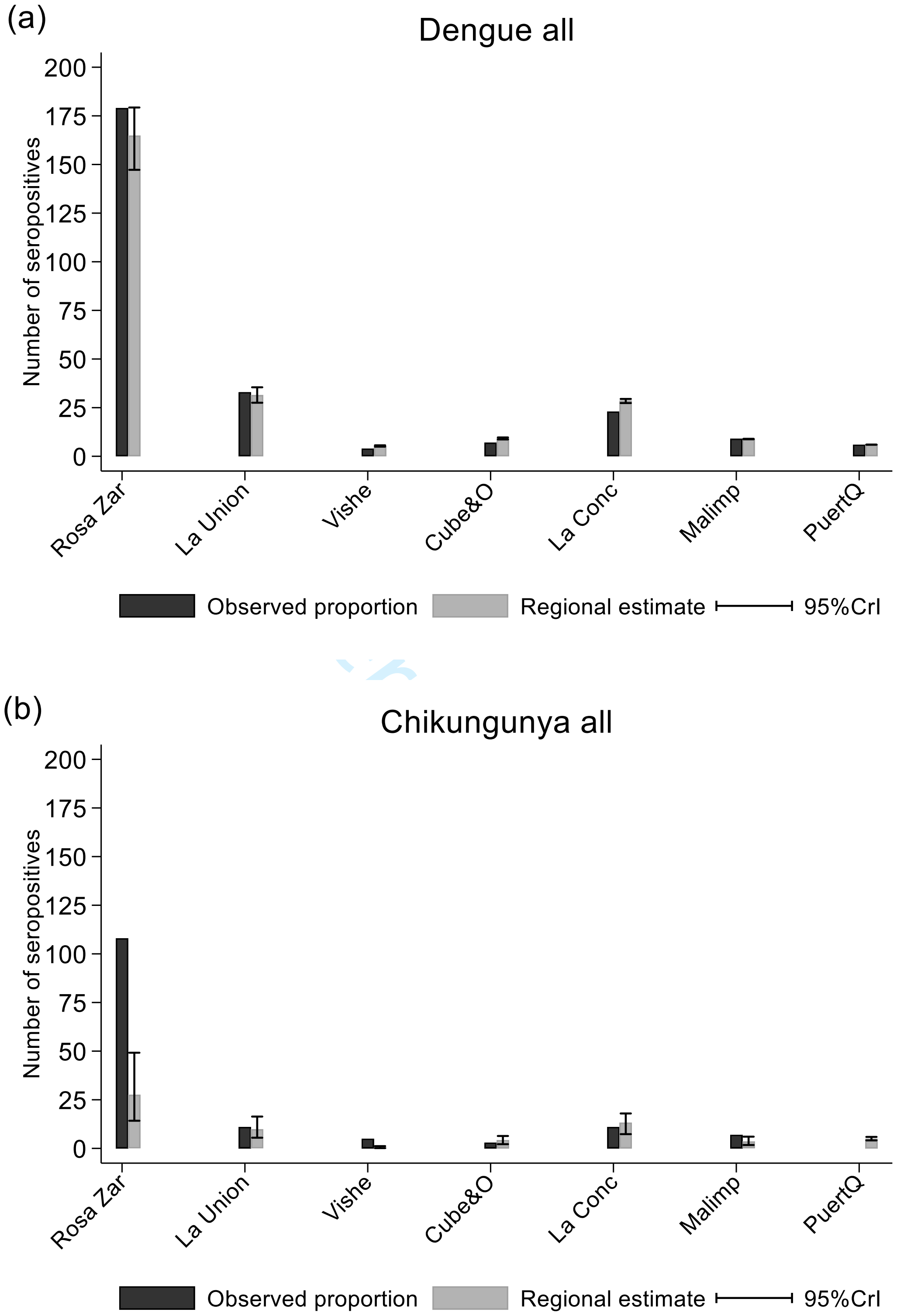
(a)
Dengue all
Number of seropositives
200
175
150
125
100
75
50
25
0
Rosa Zar
La Union
Vishe
Cube&O
La Conc
Malimp
PuertQ
Observed proportion
Regional estimate
95%CrI
(b)
Chikungunya all
Number of seropositives
200
175
150
125
100
75
50
25
0
Rosa Zar
La Union
Vishe
Cube&O
La Conc
Malimp
PuertQ
Observed proportion
Regional estimate
95%CrI

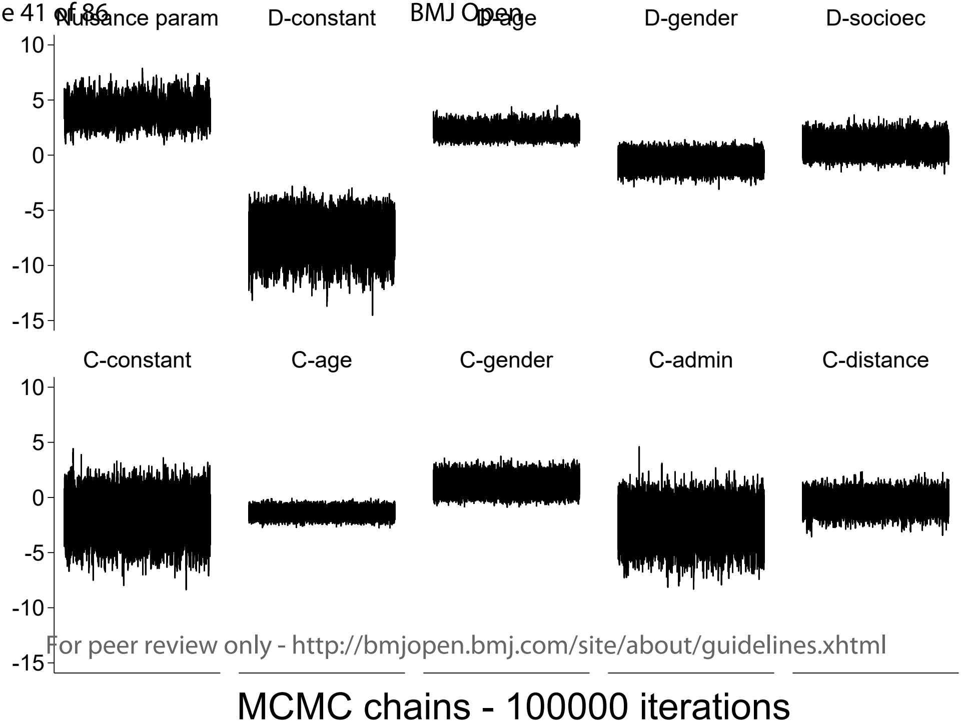
Nuisance param
D-constant
D-age
D-gender
D-socioec
C-constant
C-age
C-gender
C-admin
C-distance
10
5
0
-5
-10
-15
Parameters values (log scale)
MCMC chains - 100000 iterations

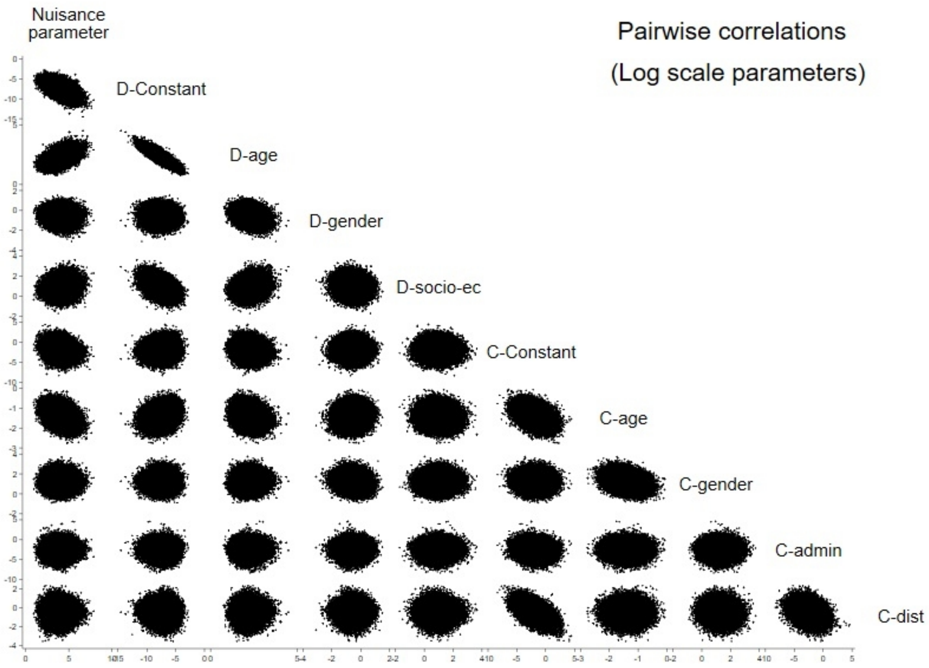

224x162mm (96 x 96 DPI)

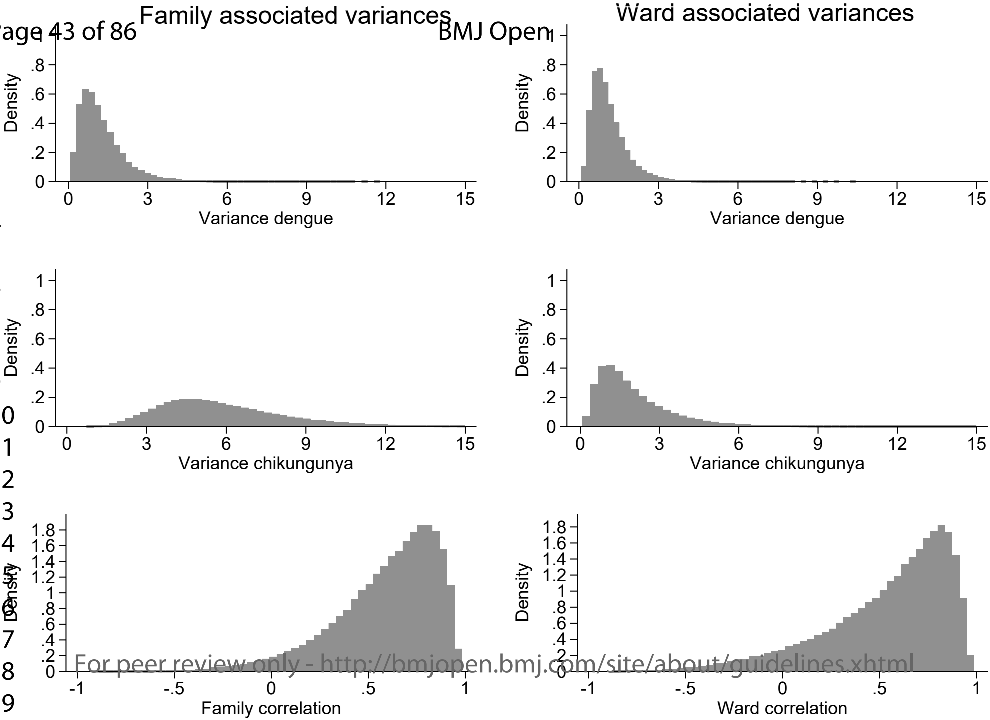

For peer review only - http://bmjopen.bmj.com/site/about/guidelines.xhtml

		Serological cohort 151 households	Ecuavida 2404 households	Serological cohort vs Ecuavida
**Variable**	**Category**	**Number/Prop**	**Number/Prop**	**One-sample test**
Administrative location	Urban	115 (76.2%)	1,796 (75%)	p=0.74
	Rural	36 (23.8%)	608 (25%)	
Socio Economic Status	High	45 (29.8%)	797 (33.2%)	P=0.46
	Low	106 (70.2%)	1,607(66.8%)	

Supplementary Table 1

For peer review only

	ALL	ECUAVIDA	SAMPLE	Prop of ALL	Prop of ECUAVIDA	Prop of SAMPLE	PROP ECUAVIDA / PROP ALL	PROP SAMPLE / PROP ECUAVIDA	RATIO OF THE LAST	WEIGHTS= 1/RATIO
**Rosa Zarate**	16342	1578	102	0.353	0.656	0.675	1.862	1.029	1.916	**0.522**
**La Concord**	10662	217	13	0.230	0.090	0.086	0.392	0.954	0.374	**2.672**
**PuertoQuito**	5037	27	3	0.109	0.011	0.020	0.103	1.769	0.183	**5.469**
**La Unión**	4963	351	21	0.107	0.146	0.139	1.364	0.953	1.299	**0.770**
**Cube+Others**	4163	49	6	0.090	0.020	0.040	0.227	1.949	0.442	**2.260**
**Malimpia**	4004	81	3	0.086	0.034	0.020	0.390	0.590	0.230	**4.348**
**Viche**	1185	101	3	0.026	0.042	0.020	1.644	0.473	0.777	**1.287**
**Total**	46356	2404	151	1	1	1				

Supplementary Table 2

For peer review only

Year	Population		Dengue		Chickungunya		Zika	
	**Ecuador**	**Esmeraldas**	**Ecuador**	**Esmeraldas**	**Ecuador**	**Esmeraldas**	**Ecuador**	**Esmeraldas**
2014	15,944,275	187,880	15, 584 (0.10%)	984 (0.5%)	39 (~0%)	0 (0%)	0 (0%)	0 (0%)
2015	16,144,368	196,350	42, 499 (0.26%)	3,482 (1.8%)	34,101 (0.2%)	10,477 (5.3%)	1 (0%)	0 (0%)
2016	16,385,068	204,820	13,612 (0.08%)	1,311 (0.6%)	1, 860 (0.01%)	301 (0.15%)	2946(0.02%)	192(0.09%)

Supplementary Table 3

For peer review only

Age Group	Age distribution of census population in Esmeraldas		Age distribution of serological sample		Age-adjusted weights=
	Numbers	Proportions	Numbers	Proportions	
0-9	32452	0.211	147	0.461	**0.458**
10-19	33798	0.219	7	0.022	**9.999**
20-29	26507	0.172	46	0.144	**1.193**
30-39	20092	0.130	79	0.248	**0.527**
40-49	16477	0.107	33	0.103	**1.034**
50-59	12279	0.080	4	0.013	**6.357**
60+	12430	0.081	3	0.009	**8.581**

Supplementary Table 4

For peer review only

OUTCOME	CATEG	MEAN	SD	LOWER 95% CrI	MEDIAN	UPPER 95% CrI
				**ALL**		
		MEAN	SD	P025	MEDIAN	P975
**D+C-**	**ALL**	0.58	0.09	0.38	0.59	0.74
**D-C+**		0.01	0.01	0.00	0.01	0.03
**D+C+**		0.26	0.11	0.08	0.25	0.50
**D+**		0.84	0.04	0.75	0.84	0.91
**C+**		0.27	0.11	0.09	0.27	0.52
				**BY SEX**		
**D+C-**	**MALE**	0.69	0.08	0.51	0.70	0.81
	**FEMALE**	0.53	0.10	0.32	0.54	0.72
**D-C+**	**MALE**	0.008	0.01	0.001	0.01	0.023
	**FEMALE**	0.018	0.01	0.004	0.02	0.04
**D+C+**	**MALE**	0.13	0.09	0.03	0.12	0.33
	**FEMALE**	0.32	0.12	0.11	0.31	0.57
				**SEX DIFFERENCE**		
**D+C-**	**F - M**	-0.15	0.07	-0.31	-0.15	-0.018
**D-C+**		0.01	0.007	0.00	0.009	0.03
**D+C+**		0.18	0.09	0.04	0.18	0.37
				**BY AREA OF RESIDENCE**		
**D+C-**	**URBAN**	0.46	0.12	0.23	0.47	0.68
	**RURAL**	0.68	0.09	0.39	0.70	0.86
**D-C+**	**URBAN**	0.017	0.01	0.004	0.015	0.04
	**RURAL**	0.006	0.005	0.00	0.005	0.02
**D+C+**	**URBAN**	0.39	0.14	0.13	0.38	0.68
	**RURAL**	0.21	0.13	0.03	0.18	0.53
				**AREA OF RESIDENCE DIFFERENCES**		
**D+C-**	**U - R**	-0.21	0.16	-0.52	-0.22	0.13
**D-C+**		0.01	0.01	-0.004	0.01	0.03
**D+C+**		0.18	0.18	-0.21	0.18	0.53
				**BY SOCIOECONOMIC STATUS**		
**D+C-**	**LOW**	0.61	0.11	0.37	0.63	0.79
	**HIGH**	0.51	0.13	0.24	0.52	0.73
**D-C+**	**LOW**	0.009	0.006	0.001	0.007	0.03
	**HIGH**	0.016	0.01	0.002	0.013	0.04
**D+C+**	**LOW**	0.26	0.12	0.07	0.24	0.52
	**HIGH**	0.28	0.15	0.06	0.26	0.61
				**SOCIOECONOMIC STATUS DIFFERENCES**		
**D+C-**	**L-H**	0.10	0.14	-0.18	-0.10	0.39
**D-C+**		-0.006	0.01	-0.07	0.00	0.008
**D+C+**		-0.017	0.16	-0.35	0.02	0.29

Supplementary Table 5

For peer review only

Supplementary: The statistical model and inference

The model

This is a model borrowed from econometrics (1) and recently used in the context of infectious diseases which occur simultaneously or are inherently influenced by correlated binary outcomes. We adapted the methodology in the framework of these two binary highly correlated outcomes which can be associated with a similar (or not) set of covariates and the presence of one is highly correlated with the other. A lay description of the approach is presented in the main text. Here, we give details on the mathematical equations and the statistical inference.

Let us denote by X and Y the two binary random variables indicating the presence/absence of an individual to DENV and CHIKV, respectively. This pair of responses define a binary bivariate outcome denoted by (X, Y).

Let us also denote

$R_i = 1 \times \boldsymbol{K}_X$ vector of variables associated with X

$\boldsymbol{\beta} = \boldsymbol{K}_X \times 1$ vector of unknown parameters which quantify the effect of variables associated with X

$Q_i = 1 \times \boldsymbol{K}_Y$ vector of variables associated with Y

$\boldsymbol{\gamma} = \boldsymbol{K}_Y \times 1$ vector of unknown parameters which quantify the effect of variables associated with Y,

$i = \overline{1, 319}$, $j = \overline{1, 151}$, $k = \overline{1{,}49}$

For peer review only - http://bmjopen.bmj.com/site/about/guidelines.xhtml

NB: We deliberately keep the generality of the model specification although in our scenario $\boldsymbol{K}_X = \boldsymbol{K}_Y$

Then, the two simultaneous logistic equations which depict the nonlinear association with age and the rest of the other variables are written in terms of a system of conditional probabilities on the potential explanatory variables and their random effects:

$$\begin{cases} log\left(\frac{P(X=1|Y)}{P(X=0|Y)}\right)_{ijk} = \beta_{age} \times \log(\text{age}_{ijk}) + \boldsymbol{\beta} \times R_{ijk} + \boldsymbol{\alpha} \times Y_{ijk} + w_{ijk}^{age} + w_k^{ward} + v_{0k}^1 + u_{0jk}^1 \\ log\left(\frac{P(Y=1|X)}{P(Y=0|X)}\right)_{ijk} = \gamma_{age} \times \log(\text{age}_{ijk}) + \boldsymbol{\gamma} \times Q_{ijk} + \boldsymbol{\theta} \times X_{ijk} + w_{ijk}^{age} + w_k^{ward} + v_{0k}^2 + u_{0jk}^2 \end{cases} \quad (1)$$

The triple index (ijk) – denotes individual i in the family j and the ward k

w_{ijk}^{age} denote the age-group constants associated weights (see the subsequent section on Population adjusting weights)

w_k^{ward} denote the ward constants associated weights (see the subsequent section on Population adjusting weights)

$v_{0k}^{1,2}$ denote the random effect associated with the ward

$u_{0jk}^{1,2}$ denote the random effect associated with the family

The latter two sets of random effects are assumed to be drawn from a two normal bivariate normal distributions (MVN) which model the shared and unshared similarities and account for unmeasured aspects such as mosquitos' burden around the households or weather patterns for instance.

$$\begin{pmatrix} v_{0k}^1 \\ v_{0k}^2 \end{pmatrix} \sim MVN\left(\begin{pmatrix} 0 \\ 0 \end{pmatrix}, \sum \begin{pmatrix} (v_0^1)^2 & \rho_v v_0^1 v_0^2 \\ \rho_v v_0^1 v_0^2 & (v_0^2)^2 \end{pmatrix}\right)$$

For peer review only - http://bmjopen.bmj.com/site/about/guidelines.xhtml

$$\begin{pmatrix} u_{0jk}^{1} \\ u_{0jk}^{2} \end{pmatrix} \sim MVN\left(\begin{pmatrix} 0 \\ 0 \end{pmatrix}, \sum\begin{pmatrix} (u_0^1)^2 & \rho_u u_0^1 u_0^2 \\ \rho_u u_0^1 u_0^2 & (u_0^2)^2 \end{pmatrix}\right)$$

On one hand side, from the first simultaneous equation specification (1):

$$\boldsymbol{\alpha} = log\left[\frac{P(X=1|Y=1)}{P(X=0|Y=1)}\right] - log\left[\frac{P(X=1|Y=0)}{P(X=0|Y=0)}\right] = log\frac{P(X=1,Y=1)/P(Y=1)}{P(X=0,Y=1)/P(Y=1)} - log\frac{P(X=1,Y=0)/P(Y=0)}{P(X=0,Y=0)/P(Y=0)} = log\left[\frac{P(X=1,Y=1)\times P(X=0,Y=0)}{P(X=0,Y=1)\times P(X=1,Y=0)}\right] \quad (2)$$

From the second simultaneous equation specification:

$$\boldsymbol{\theta} = log\left[\frac{P(Y=1|X=1)}{P(Y=0|X=1)}\right] - log\left[\frac{P(Y=1|X=0)}{P(Y=0|X=0)}\right] = log\frac{P(X=1,Y=1)/P(X=1)}{P(X=0,Y=1)/P(X=1)} - log\frac{P(X=1,Y=0)/P(X=0)}{P(X=0,Y=0)/P(X=0)} = log\left[\frac{P(X=1,Y=1)\times P(X=0,Y=0)}{P(X=0,Y=1)\times P(X=1,Y=0)}\right] \quad (3)$$

Hence, using the key assumption that $\boldsymbol{\alpha} = \boldsymbol{\theta}$ and keeping the fixed effects only for the sake of simplicity, the system can be written as

$$\begin{cases} log\left(\dfrac{P(X=1|Y)}{P(X=0|Y)}\right)_i = \boldsymbol{\beta} \times R_i + \boldsymbol{\alpha} \times Y_i \\ log\left(\dfrac{P(Y=1|X)}{P(Y=0|X)}\right)_i = \boldsymbol{\gamma} \times Q_i + \boldsymbol{\alpha} \times X_i \end{cases}$$

This implies that, if the occurrence of dengue makes more likely (or less likely) the occurrence of chikungunya, the converse is also necessarily true and $\boldsymbol{\alpha}$ is deemed as a nuisance parameter

This crucial assumption ($\boldsymbol{\alpha} = \theta$) and elementary rules of conditioned probabilities leads to the following four statements derived from

For peer review only - http://bmjopen.bmj.com/site/about/guidelines.xhtml

$$\begin{cases} \dfrac{P(X=1|Y=0)}{P(X=0|Y=0)} = \dfrac{P(X=1,Y=0)}{P(X=0,Y=0)} = exp(\boldsymbol{\beta} \times \boldsymbol{R}) & (4) \\ \dfrac{P(X=1|Y=1)}{P(X=0|Y=1)} = \dfrac{P(X=1,Y=1)}{P(X=0,Y=1)} = exp(\boldsymbol{\beta} \times \boldsymbol{R} + \alpha) & (5) \end{cases}$$

$$\begin{cases} \dfrac{P(Y=1|X=0)}{P(Y=0|X=0)} = \dfrac{P(Y=1,X=0)}{P(Y=0,X=0)} = exp(\boldsymbol{\gamma} \times \boldsymbol{Q}) & (6) \\ \dfrac{P(Y=1|X=1)}{P(Y=0|X=1)} = \dfrac{P(Y=1,X=1)}{P(Y=0,X=1)} = exp(\boldsymbol{\gamma} \times \boldsymbol{Q} + \alpha) & (7) \end{cases}$$

Hence, combining either (4) + (7) (or (5) + (6)) by multiplication

$$\frac{P(X=1,Y=0)}{P(X=0,Y=0)} \times \frac{P(Y=1,X=1)}{P(Y=0,X=1)} =$$

$$\frac{P(Y=1,X=1)}{P(Y=0,X=0)} = exp(\boldsymbol{\beta} \times \boldsymbol{R}) \times\ exp(\boldsymbol{\gamma} \times \boldsymbol{Q} + \alpha) = exp(\boldsymbol{\beta} \times \boldsymbol{R} + \boldsymbol{\gamma} \times \boldsymbol{Q} + \alpha)$$

But all four probabilities situations ((1, 0), (0, 1), (1, 1), (0,0)) sum to 1:

$$P(X=1,Y=0) + P(X=0,Y=1) + P(Y=0,X=0) + P(Y=1,X=1) = 1$$

$$P(X=0,Y=0) + P(X=0,Y=0) \times exp(\boldsymbol{\gamma} \times Q) + P(Y=0,X=0) \times exp(\boldsymbol{\beta} \times \boldsymbol{R}) + P(Y=0,X=0) \times exp(\boldsymbol{\beta} \times \boldsymbol{R} + \boldsymbol{\gamma} \times \boldsymbol{Q} + \alpha) = 1$$

So

$$P(X=0,Y=0) \times (1 + exp(\boldsymbol{\gamma} \times \boldsymbol{Q}) + exp(\boldsymbol{\beta} \times \boldsymbol{R}) + exp(\boldsymbol{\beta} \times \boldsymbol{R} + \boldsymbol{\gamma} \times \boldsymbol{Q} + \alpha))=1$$

Therefore, the predicted joint probabilities can be derived as it follows:

$$P(X=0,Y=0) = \frac{1}{1 + exp(\boldsymbol{\gamma} \times \boldsymbol{Q}) + exp(\boldsymbol{\beta} \times \boldsymbol{R}) + exp(\boldsymbol{\beta} \times \boldsymbol{R} + \boldsymbol{\gamma} \times \boldsymbol{Q} + \alpha)}$$

$$P(X=0,Y=1) = \frac{exp(\boldsymbol{\gamma} \times \boldsymbol{Q})}{1 + exp(\boldsymbol{\gamma} \times \boldsymbol{Q}) + exp(\boldsymbol{\beta} \times \boldsymbol{R}) + exp(\boldsymbol{\beta} \times \boldsymbol{R} + \boldsymbol{\gamma} \times \boldsymbol{Q} + \alpha)}$$

For peer review only - http://bmjopen.bmj.com/site/about/guidelines.xhtml

$$P(X=1,Y=0)=\frac{exp(\boldsymbol{\beta}\times\boldsymbol{R})}{1+exp(\boldsymbol{\gamma}\times\boldsymbol{Q})+exp(\boldsymbol{\beta}\times\boldsymbol{R})+exp(\boldsymbol{\beta}\times\boldsymbol{R}+\boldsymbol{\gamma}\times\boldsymbol{Q}+\alpha)}$$

$$P(X=1,Y=1)=\frac{exp(\boldsymbol{\beta}\times\boldsymbol{R}+\boldsymbol{\gamma}\times\boldsymbol{Q}+\alpha)}{1+exp(\boldsymbol{\gamma}\times\boldsymbol{Q})+exp(\boldsymbol{\beta}\times\boldsymbol{R})+exp(\boldsymbol{\beta}\times\boldsymbol{R}+\boldsymbol{\gamma}\times\boldsymbol{Q}+\alpha)}$$

Denoting by

$$I_{ij}=\{k|X_k=i,Y_k=j\}\ where\ \ i,j=0,1$$

If the vector of the unknown parameters are denoted by $\phi=(\alpha,\boldsymbol{\beta},\boldsymbol{\gamma})$, the model likelihood can be formally written as

$$L(\phi|Y_k,X_k;\boldsymbol{R},\boldsymbol{Q})=\prod_{i=0,1}\prod_{j=0,1}\prod_{k\in I_{ij}}P(X_k=i,Y_k=j)$$

Bayesian inference consist of summarizing the posterior distributions of the model parameters and requires the joint probability distribution over all random quantities, parameters or observables. The marginal and conditional probability distributions of the vector of unknown parameters are denoted by $Pr(\cdot)$ and $Pr(\cdot|\cdot)$. The ϕ posterior distribution is proportional to the likelihood multiplied by their prior distributions – all assumed non informative as stated in the text. That is

$$Pr(\phi|Y_k,X_k;\boldsymbol{R},\boldsymbol{Q})\propto L(\phi|Y_k,X_k;\boldsymbol{R},\boldsymbol{Q})\times Pr(\phi)$$

Statistical Inference

The simultaneous equations systems (1) define a probability model which has been implemented in OpenBUGS - which makes this approach very appealing even for larger datasets (OpenBUGS program for the simplest model appended). A Bayesian framework for statistical inference using Markov chain Monte Carlo (MCMC) estimation methods naturally accommodates missing

For peer review only

For peer review only - http://bmjopen.bmj.com/site/about/guidelines.xhtml

responses (5%) in the data and allows straightforward assessment of uncertainties in nonlinear models under missing at random assumption. Sensitivities analyses to the missing data (by assuming all positives and all negatives) have been conducted but the results were not altered.

The posterior conditional odds ratios (OR) distributions are presented in Table 2. However, for an easily digestible reading, the summary statistics are presented in terms of means and standard deviations of the predicted joint probabilities by age and stratified by different socio-economic factors that we accounted for.

The MCMC estimation followed 1 million iterations and the corresponding chains (log scale) are displayed in Supplementary Figure 8 – including that of the nuisance parameter. Supplementary Figure 9 display the correlation between the parameters which are all lower than 0.5 in absolute values with the exception of that between the constant and age corresponding to the dengue equations with appear to be highly negatively correlated (-0.87).

Non-informative priors have been used for parameter estimation.

Population adjusting weights used in modelling

We used the 2010 Census data (the latest) adjusted for an average annual growth of in the Ecuador population. All statistical analyses should ultimately serve population predictions. The post-stratification weights, namely calibration, is a technique which corrects the estimation for population misrepresentation which often occurs in ad-hoc surveys or when researchers use already recruited participants. One set of weights are aimed at reducing the effect of Rosa Zarate where most of the data were collected and at inflating the influence of the misrepresented groups (La Union and 10-19 years) on the model likelihood. These weights are associated with each

For peer review only - http://bmjopen.bmj.com/site/about/guidelines.xhtml

individual and are additive for the log scale model formulation of the model (see equations above). The other set of weights are aimed at reducing the effect of those in young group and inflate the effects of older people less represented in the sample. The weights are displayed Supplementary Tables 3 and 4. Each observation is associated with a weight according to individual's age group and one by the household location and their additive effect is accounted for in the predictors (log scale so their effect on the actual odds ratios or odds would be multiplicative).

Goodness of fit

The model is developed using a fully parametric paradigm and inherits complexities including random effects for binary outcomes which are not associated with any classical or formal goodness of fit measures.

However, in reference to the basic independent binary outcomes, the only goodness-of-fit measure following a potential *individual based model* fit to these data is that of Hosmer-Lemeshow statistic. That invokes partition of the data *into groups* (https://thestatsgeek.com/2014/02/16/the-hosmer-lemeshow-goodness-of-fit-test-for-logistic-regression/). That is because the individual level raw observations are either 0 or 1 whilst the individual level predicted values can be anywhere between 0 and 1 and hence a one-to-one observed-predicted plot often used for continuous data loses its meaning.

Hence, our approach alludes to that of basic logistic regression and follows similar lines to those developed in Chis Ster, I. Journal of Applied Statistics, 2012. **39**(11): p. 2523-2542, (*Inference for serological surveys investigating past exposures to infections resulting in long-lasting*

For peer review only - http://bmjopen.bmj.com/site/about/guidelines.xhtml

immunity – an approach using finite mixture models with concomitant information) within a related and yet equally complex modelling framework.

We split the data into groups, by 5 years age-groups and geographical sub-regions, and calculated the average predicted probabilities and their 95%CrIs for the outcomes (((D+, C-), (D-, C+), (D+, C+) and also for all dengue and chikungunya) within each group. Then we calculated the expected number of positives corresponding each group and each outcome component by multiplying the corresponding average predicted probabilities with the total number of people within that group. We plotted the observed numbers vs. the predicted numbers in each group.

Given the full parametric, parsimonious nature of this model, and the average sized data – we deemed our model as a good fit to these data.

OpenBUGS code:

```
model{
    #model's likelihood
       for (i in 1:319){
              log_age[i]<-log(age_final_years[i]);
              log_dist[i]<-log(1+min_dist[i]/250);
              weights_final[i]<-weights[i]+weight_age[i];

              dengue[i]~dbern(p[i,1]);
              chick[i] ~dbern(p[i,2]);
              eta[i,1]<-beta1[1]+beta1[2]*log_age[i]+alpha*chick[i]+beta1[3]*gender[i]+
                              beta1[4]*ses_bin[i]+
                              weights_final[i]+
                              theta[family[i],1]+
                              ward_level[ward_index[i],1];

             eta[i,2]<- beta2[1]+beta2[2]*log_age[i]+alpha*dengue[i]+
                              beta2[3]*gender[i]+
                              beta2[4]*urbanrural[i]+beta2[5]*log_dist[i]+
                              weights_final[i]+
                              theta[family[i],2]+
                              ward_level[ward_index[i],2];
               logit(p[i,1])<-eta[i,1];
              logit(p[i,2])<-eta[i,2];
       }
       for (j in 1:151){
              theta[j,1:2] ~ dmnorm(mu.fam[],R_fam[,]);
        }
    for(j in 1:49){
         ward_level[j,1:2] ~ dmnorm(mu.ward[],R_ward[,]);
    }
    for (k in 1:2){

                    mu.fam[k] <-0.0;
                  mu.ward[k] <-0.0;
       }
```


For peer review only - http://bmjopen.bmj.com/site/about/guidelines.xhtml

```
     R_fam[1:2,1:2] ~dwish(Omega_fam[,],2);
     R_ward[1:2,1:2]~dwish(Omega_ward[,],2);
     Omega_fam[1,1]<-1; Omega_fam[1,2]<-0; Omega_fam[2,1]<-0; Omega_fam[2,2]<-1;
  Omega_ward[1,1]<-1; Omega_ward[1,2]<-0; Omega_ward[2,1]<-0; Omega_ward[2,2]<-1;


    Sigma.fam[1:2, 1:2] <-inverse(R_fam[,]);
    Sigma.ward[1:2, 1:2]<-inverse(R_ward[,]);
    for (j in 1:2){
        sigma.fam[j]<-sqrt(Sigma.fam[j,j]);
        sigma.ward[j]<-sqrt(Sigma.ward[j,j]);
        for(k in 1:j){
                rho_fam[j,k]<-0;
                 rho_ward[j,k]<-0;
         }
        for(k in (j+1):2){
                rho_fam[j,k]<-Sigma.fam[j,k]/(sigma.fam[j]*sigma.fam[k]);
                rho_ward[j,k]<-Sigma.ward[j,k]/(sigma.ward[j]*sigma.ward[k]);
        }
    }
    alpha ~ dnorm( 0.0, 0.00001);
    for (j in 1:4){
        beta1[j] ~ dnorm( 0.0, 0.00001);
        beta2[j] ~ dnorm( 0.0, 0.00001);
        exp_beta1[j]<-exp(beta1[j]);
         exp_beta2[j]<-exp(beta2[j]);
         eff1[j] <-step(beta1[j]);
         eff2[j] <-step(beta2[j]);
    }
           exp_alpha<-exp(alpha);
           beta2[5] ~ dnorm( 0.0, 0.00001);
           exp_beta2[5]<-exp(beta2[5]);
}
```

References

1. Schmidt P, Strauss RP. Estimation of Models with Jointly Dependent Qualitative Variables: A Simultaneous Logit Approach. Econometrica. 1975;43(4):745-55.
2. Ghebremichael M. Joint modeling of correlated binary outcomes: HIV-1 and HSV-2 co-infection. Journal of Applied Statistics. 2015 2015/10/03;42(10):2180-91.
3. Goldstein H. Multilevel Statistical Models. 4th Edition ed: JohnWiley and Sons, Ltd, Chichester; 2011.

For peer review only - http://bmjopen.bmj.com/site/about/guidelines.xhtml

STROBE 2007 (v4) checklist of items to be included in reports of observational studies in epidemiology*
Checklist for cohort, case-control, and cross-sectional studies (combined)

Section/Topic	Item #	Recommendation	Reported on page #
**Title and abstract**	1	(*a*) Indicate the study's design with a commonly used term in the title or the abstract	#1 Title:Age-dependent prevalence
		(*b*) Provide in the abstract an informative and balanced summary of what was done and what was found	#3 Abstract
**Introduction**			
Background/rationale	2	Explain the scientific background and rationale for the investigation being reported	#6 Introduction
Objectives	3	State specific objectives, including any pre-specified hypotheses	#7 Introduction
**Methods**			
Study design	4	Present key elements of study design early in the paper	#8 Materials and methods
Setting	5	Describe the setting, locations, and relevant dates, including periods of recruitment, exposure, follow-up, and data collection	#8 Materials and methods
Participants	6	*Cross-sectional study*—Give the eligibility criteria, and the sources and methods of selection of participants	#8 Materials and methods
		(*b*) *Cohort study*—For matched studies, give matching criteria and number of exposed and unexposed<br>*Case-control study*—For matched studies, give matching criteria and the number of controls per case	
Variables	7	Clearly define all outcomes, exposures, predictors, potential confounders, and effect modifiers. Give diagnostic criteria, if applicable	#8-9 Statistical Methods
Data sources/ measurement	8*	For each variable of interest, give sources of data and details of methods of assessment (measurement). Describe comparability of assessment methods if there is more than one group	#8 Materials and methods
Bias	9	Describe any efforts to address potential sources of bias	#6, #16
Study size	10	Explain how the study size was arrived at	#8 – random sample from an existing cohort
Quantitative variables	11	Explain how quantitative variables were handled in the analyses. If applicable, describe which groupings were chosen and why	#8 Materials and methods
Statistical methods	12	(*a*) Describe all statistical methods, including those used to control for confounding	#8-9 Statistical Methods

For peer review only

		(*b*) Describe any methods used to examine subgroups and interactions	#8-9 Statistical Methods
		(*c*) Explain how missing data were addressed	NA
		(*d*) *Cohort study*—If applicable, explain how loss to follow-up was addressed<br>*Case-control study*—If applicable, explain how matching of cases and controls was addressed<br>*Cross-sectional study*—If applicable, describe analytical methods taking account of sampling strategy	#8-9 Statistical Methods<br>Random sample from an existing cohort.<br>Weighting adjustments to represent the population
		(*e*) Describe any sensitivity analyses	NA
**Results**			
Participants	13*	(a) Report numbers of individuals at each stage of study—eg numbers potentially eligible, examined for eligibility, confirmed eligible, included in the study, completing follow-up, and analysed	319
		(b) Give reasons for non-participation at each stage	NA
		(c) Consider use of a flow diagram	NA
Descriptive data	14*	(a) Give characteristics of study participants (eg demographic, clinical, social) and information on exposures and potential confounders	#18 Table 1
		(b) Indicate number of participants with missing data for each variable of interest	NA
		(c) *Cohort study*—Summarise follow-up time (eg, average and total amount)	NA
Outcome data	15*	*Cohort study*—Report numbers of outcome events or summary measures over time	
		Case-control study—Report numbers in each exposure category, or summary measures of exposure	
		Cross-sectional study—Report numbers of outcome events or summary measures	#18 -19 Table 1, Table 2
Main results	16	(*a*) Give unadjusted estimates and, if applicable, confounder-adjusted estimates and their precision (eg, 95% confidence interval). Make clear which confounders were adjusted for and why they were included	#10 Results
		(*b*) Report category boundaries when continuous variables were categorized	#18 -19 Table 1
		(*c*) If relevant, consider translating estimates of relative risk into absolute risk for a meaningful time period	#10 Results – Supplementary Tables
Other analyses	17	Report other analyses done—eg analyses of subgroups and interactions, and sensitivity analyses	#10 Results<br>#13-16 Discussion
**Discussion**			
Key results	18	Summarise key results with reference to study objectives	#13-16 Discussion
Limitations	19	Discuss limitations of the study, taking into account sources of potential bias or imprecision. Discuss both direction and magnitude of any potential bias	#4 Strength and limitations

For peer review only

			#13-16 Discussion
Interpretation	20	Give a cautious overall interpretation of results considering objectives, limitations, multiplicity of analyses, results from similar studies, and other relevant evidence	#13-16 Discussion
Generalisability	21	Discuss the generalisability (external validity) of the study results	#13-16 Discussion
**Other information**			
Funding	22	Give the source of funding and the role of the funders for the present study and, if applicable, for the original study on which the present article is based	#24 Wellcome Trust (088862/Z/09/Z)

*Give information separately for cases and controls in case-control studies and, if applicable, for exposed and unexposed groups in cohort and cross-sectional studies.

Note: An Explanation and Elaboration article discusses each checklist item and gives methodological background and published examples of transparent reporting. The STROBE checklist is best used in conjunction with this article (freely available on the Web sites of PLoS Medicine at http://www.plosmedicine.org/, Annals of Internal Medicine at http://www.annals.org/, and Epidemiology at http://www.epidem.com/). Information on the STROBE Initiative is available at www.strobe-statement.org.

For peer review only

We would like to thank both reviewers for their detailed comments on the manuscript – they helped to improve the narrative of the paper. Neither reviewer flagged flaws in the science of the manuscript and complimented it on being "*interesting and diligently conducted*" (Reviewer 1). We believe that the interest shown by the reviewers and their detailed analyses of the manuscript likely will translate into significant interest by the readership of your journal, both by scientists and policy makers.

In response to the reviewers' comments, we have done more analyses to support our findings and modified the text in response to reviewers' comments on our resubmission. May we add that we did not agree with all the comments and have provided robust rebuttals where necessary. We feel that some of the comments were not reasonable and have tried to remedy areas of the text and analyses that we feel have not been understood clearly. We now provide further clarity in the text and further detailed analyses in the supplementary files, particularly in response to the goodness of fit questions.

We would also like to reiterate 3 areas of novelty for your attention and consideration that makes the manuscript of likely interest to readers with different scientific backgrounds:

1. There are limited data on dengue and chikungunya virus infections from Ecuador and other Pacific regions of Latin America apart from aggregated data communicated to international bodies (PAHO/WHO/CDC);
2. There are limited methodologies available to address the issue of co-exposures to co-circulating infections with considerable epidemiological and clinical similarities, a criticism highlighted in recent systematic review based on 147 studies (page 5 in Fritzell, C. et al., *Current challenges and implications for dengue, chikungunya and Zika seroprevalence studies worldwide: A scoping review.* PLoS Neglected Tropical Diseases, 2018. **12**(7))
3. Our manuscript also provides a novel solution (i.e. post-stratification weighting) to another criticism emphasised in the above review relating to imperfect sampling of populations used to draw inferences at population level (page 17: "*potential biases such sampling introduces must be considered during the analysis process*").

Below we provide point-by-point responses to the reviewer's comments

For peer review only - http://bmjopen.bmj.com/site/about/guidelines.xhtml

Reviewer 1

Reviewer Name: Lorenzo Cattarino

Institution and Country: Imperial College London, UK

Comment 1**:** *The authors examined patterns of age-dependent seroprevalence of dengue and chikungunya in a tropical district of coastal Ecuador and investigated potential determinants of risk of exposure to the two viruses (sex, urban vs rural area of residence, socio-economic level, etc.). They first conducted a serology surveys of 319 individuals in 151 households using IgG ELISA. They then fitted logistic regression models to individual-level seroprevalence data using different sets of explanatory variables, including sex, area of residence, socio-economic level, a measure of crowding and one of potential human dispersal.*

The authors fitted separate models for data samples tested positive to dengue (tested negative for chikungunya), data samples tested positive for chikungunya (tested negative for dengue) and for data samples tested positive to both viruses.
Overall, ***I find the modelling work interesting, diligently conducted, model code is provided which is always good and the manuscript is well written.*** *Seroprevalence studies like this one are an invaluable source of information to better characterize transmission of arboviral diseases. However, I have a couple of comments related to the modelling approach used and study conclusions.*

Response 1: We thank the reviewer for his positive general comments on the manuscript.

Comment 2: *I wonder whether the model fits the data well, particularly the dengue one (Figure 2 in the manuscript). If I read the paper correctly (and I am not quite sure I fully understand the methodology) the authors fitted the model to the raw, individual-level seroprevalence data (i.e. the fitted response variable is the binary outcome of the serology test of each individual, i.e. 1 or 0). They then asses model fit by comparing model predictions to the observed seroprevalence data, aggregated in 5-year age groups.*

A fairer comparison, in my opinion, would be to compare the individual-level predictions with the individual level observations.

No such plot is provided (a plot of predicted individual-level seropositivity by age with overlaid the observed individual-level seropositivity values), although I would be really interested in seeing it. I suspect that such a plot would look quite noisy (due to variation in mosquito population, climatic conditions, endemic nature of dengue epidemics).

Response 2: To clarify, we did *not* fit two separate logistic models to the data – it is just one model using two mixed effects logistic regressions coupled through their random effects and linked by a nuisance parameter to account for co-exposures to the two viruses, their similarities of transmission and clinical manifestations. As mentioned in the manuscript (page 10, second paragraph), there is no formal goodness-of-fit associated with a complex

2

statistical model that additionally involved mixed effects. If we just take the simplest scenario of a basic logistic regression, the only goodness-of-fit measure following an *individual based model* is that using the Hosmer-Lemeshow statistic. That invokes partition of the data *into groups*. That is because the individual level observations are either 0 or 1 whilst the individual level predictions can be anywhere between 0 and 1 (https://thestatsgeek.com/2014/02/16/the-hosmer-lemeshow-goodness-of-fit-test-for-logistic-regression/). Hence, it is not clear how such a method of goodness-of-fit would, as proposed by the reviewer, might actually work. These one-to-one, observed-predicted comparisons are associated with continuous not with binary data. The goodness-of-fit presented in the manuscript alludes to that of a basic logistic regression (i.e. involving data grouping and calculating the expected number of positives using the average predicted probabilities across groups and, using that and the total number in that group, to calculate the expected number of infections). Our original submission *did* address goodness-of-fit in this manner in supplementary Figure 7 using groups defined by age.

However, to improve clarity we have added now further explanations in both main text and in the supplementary material. Namely, we responded to this reviewer's request in 3 ways:

1. We have added a separate section in which we have explained in detail the goodness of fit methodology as below and which is now on page 7 of the Supplementary Material
2. We have altered Figure 2 as this reviewer requested by also age-group observed bars, also displayed below for your perusal
3. We have added observed-predicted plots across geographical regions (Supplementary Figure 8) and for numbers of all dengue and all chikungunya seropositive participants - also appended below.

1 Goodness of fit

The model is developed using a fully parametric paradigm and inherits complexities including random effects for binary outcomes which are not associated with any classical or formal goodness of fit measures.

However, in reference to the univariate independent binary outcomes, the only goodness-of-fit measure following a potential *individual based model* fit to these data is that of Hosmer-Lemeshow statistic. That invokes partition of the data *into groups* (https://thestatsgeek.com/2014/02/16/the-hosmer-lemeshow-goodness-of-fit-test-for-logistic-regression/). That is because the individual-level raw observations are either 0 or 1 whilst the individual level predicted values can be anywhere between 0 and 1 and hence a one-to-one observed-predicted plot often used for continuous data loses its meaning. Hence, our approach alludes to that of basic logistic regression and follows similar lines to those developed in Chis Ster, I. Journal of Applied Statistics, 2012. **39**(11): p. 2523-2542, (*Inference for serological surveys investigating past exposures to infections resulting in long-lasting immunity – an approach using finite mixture models with concomitant information)* within a related and equally complex modelling framework.

For peer review only - http://bmjopen.bmj.com/site/about/guidelines.xhtml

We split the data into groups, by 5 years age-groups and geographical sub-regions, and calculated the average predicted probabilities and their 95% CrIs for the outcomes ((D+, C-), (D-, C+), (D+, C+) and also for all dengue and chikungunya) within each group. Then we calculated the expected number of positives corresponding each group and each outcome component by multiplying the corresponding average predicted probabilities with the total number of people within that group. We plotted the observed numbers vs. the predicted numbers in each group.

Given the full parametric, parsimonious nature of this model, and the average sized data – we deemed our model as a good fit to these data.

2 The new Figure 2 as requested by the Reviewer has been redone as below:

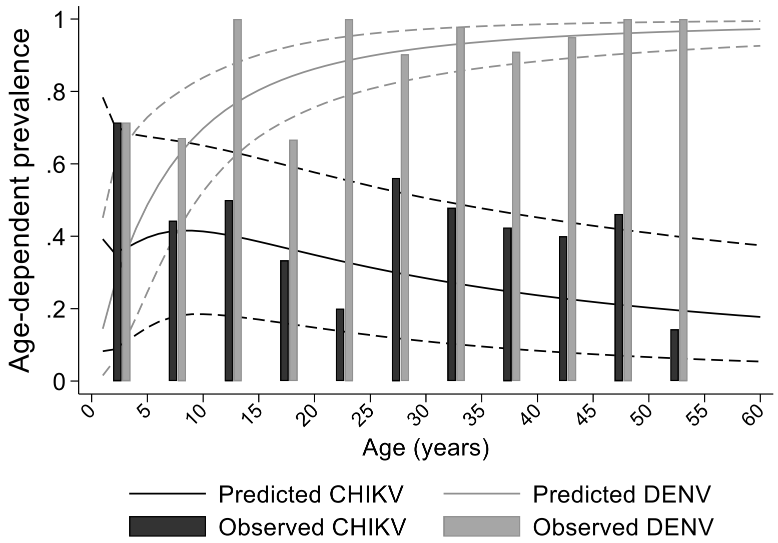

3 We also added new Figures regarding the spatial goodness of fit as requested by the reviewer. The represent the observed vs. expected numbers of all dengue and all chikungunya by geographical sub-region and reflect a reasonable goodness of fit of the model to the data.

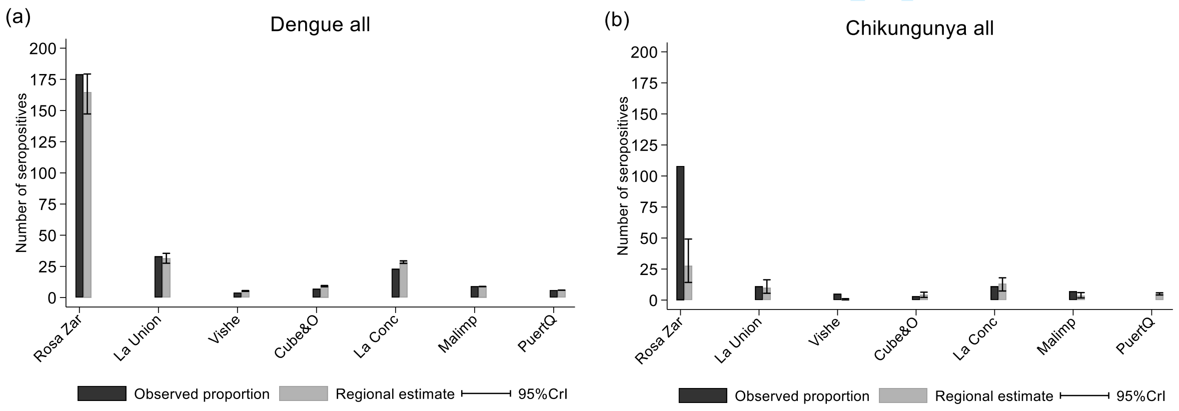

<u>Comment 3</u>: *An alternative to the individual-level fit presented here is to stratify the response by 5-year age group, calculate the proportion of seropositive individuals in each age group, and fit a catalytic model of force of infection to age-stratified seroprevalence data (not individual-level as currently done by the authors). This approach might help smoothing out variations in seroprevalence with age and possibly improving model fit. The approach I am referring to is explained in detail in several papers, including:*

N. Imai, I. Dorigatti, S. Cauchemez, N. M. Ferguson, Estimating Dengue Transmission Intensity from Sero-Prevalence Surveys in Multiple Countries. PLoS neglected tropical diseases 9, e0003719 (2015).

I. Rodriguez-Barraquer, H. Salje, D. Cummings, Opportunities for improved surveillance and control of infectious diseases from age-specific case data: insight from dengue. eLife 8, e45474 (2019).

<u>Response 3</u>: We thank the reviewer for these suggestions. There are, of course, many interesting questions to address regarding dengue and chikungunya and that includes its transmission dynamics which is a different body of work which would make a piece of research on its own. We feel that the question addressed here - which was inspired by the availability of individual detailed data - regarding age-dependent prevalence of past exposure to these co-circulating and related viruses is equally important for the local policy makers and a wide range of scientists. That is another criticism highlighted in the discussion of the December 2018 comprehensive review cited above (Fritzell, C. et al., *Current challenges and implications for dengue, chikungunya and Zika seroprevalence studies worldwide: A scoping review*. PLoS Neglected Tropical Diseases, 2018. **12**(7))

There is certainly value in fitting models to aggregated data - *in the absence of individual level data* and if the grouping correctly reflects the structure of the population to which the inference is made.

However, we feel that in our data set, besides having to ignore valuable individual information (such as further refinement by socio-demographic variables), this strategy will *not* allow interpretation of the results at the individual level (we refer to the *ecological fallacy* concept when inference from aggregated data is wrongly used to draw conclusions at individual level). In our approach, each individual is assigned a population-specific weight according to the age and to the location of their household. Our method uses population level demographics to address the issue of inference at population level in the context of field data collection.

For these reasons, we do not believe that a fit to aggregated data is a preferable approach answers our question or offers a better solution to the goodness of fit. In a previous scientific paper published in an applied statistical journal (Chis Ster, I., *Inference for serological surveys investigating past exposures to infections resulting in long-lasting immunity – an approach using finite mixture models with concomitant information*. Journal of Applied

For peer review only - http://bmjopen.bmj.com/site/about/guidelines.xhtml

Statistics, 2012. 39(11): p. 2523-2542), we used a similar goodness-of-fit approach to address a similar question – our approach was found to be acceptable by the journal after review by three professional statisticians.

One of the papers to which the reviewer refers was included in our original submission (Reference #24, now#18). This and the other paper to which the reviewer refers (now included in our resubmission as Reference #19), addressed the question of global transmission of dengue using available *aggregated not individual* data, highlighted a high level of heterogeneity and *calling for local data collection and analyses*. The 2nd paper (Rodriguez-Barraquer et al) concluded that ***"… the picture provided by serology is very incomplete and limited when trying to characterize transmission across time and space both within and between countries",*** an assertion with which we fully agree. Moreover, we believe that serological data suits better our research question which not related to infection transmission/dynamics but that of age-dependent levels of past exposure in the study population. We also agree with these authors' statement regarding high spatial heterogeneity of dengue transmission. Clearly, climate and geography play crucial roles in the dengue life-cycle and the original supplementary material stated the ward level random effects are meant to capture these unmeasured features.

Our inference, in this study, is confined to a population from coastal Ecuador and can only be generalized to a country level after considering several important limitations that are discussed in the submission. Finally, our manuscript addresses a different question to that of arbovirus transmission – there are no local data on epidemiology of *Aedes* spp. from our study area – but rather we have chosen to focus our research question on a relatively rich and unique source of data at individual level from a birth cohort to understand age-dependent seroprevalence for co-exposures to DENV and CHICKV and their determinants at individual and household levels.

Comment 4: *It would be interesting to see if such a model fits better, or not, the data. Regardless, I think that model goodness of fit should be evaluated between predictions and the actual disaggregated observations used to fit the model.*

Response 4: Please see our Responses to Comments 2 and 3 above, and also our general responses to the reviewer below for further clarification. We feel that there is some confusion in the Reviewers' requests with respect to goodness-of-fit: on one hand the reviewer advocates the use of aggregated data methods to address a different question which are clearly not an optimal approach for our individual level data (which serves to understand patterns in our populations and which the reviewer stated was of interest) and, on the other hand, asks for goodness of fit methods tailored to disaggregated data. We have provided population aggregated data in Table 1. Also, the frequencies of the serological cases can be seen in the goodness of fit Supplementary Figures 8 and 9 where they are plotted by age group and by geographical sub-region.

Comment 5: *I am struggling to appreciate why it is important to fit a combined model (dengue + /chikungunya +). The dengue and chikungunya viruses are known to potentially cross-react and it might be difficult to make useful conclusions from the combined model.*

For peer review only - http://bmjopen.bmj.com/site/about/guidelines.xhtml

Maybe the authors can expand on the rationale for this in the introduction and explain a bit more the limitations of the approach in the discussion. Related to the used of the combined model and to the broader research question, I think the justification for looking at cocirculation of the two arboviruses could be clarified and strengthened in the introduction.

Response 5: There can be issues of cross reactivity due to cross-reactivity of the distinct serum-complexes to which dengue (DENV) and chikungunya (CHIKV) viruses belong. However, for the IgG ELISA assays used in this study this is not considered to be a problem. The DENV assays used purified DENV-2 derived protein as coating which is sufficiently cross-reactive across the 4 principal DENV serotypes as to be useful for measurement of exposure to these serotypes, and which may also cause significant antibody cross-reactivity to other flaviviruses such as Zika virus but not to alphaviruses such as CHIKV (Ohst et al, Adv Exp Med Biol 2018: 1062:19-43). The antigen used for coating CHIKV plates was the recombinant envelope protein 2 of CHIKV which is highly specific for the detection of this virus (Kam et al. PLoS Negl Trop Dis 2015; 9: e3445; Fumagalli et al Virol J 2018; 15: 112). Further there was no evidence of a correlation between OD (optical density) values for IgG responses to DENV and CHIKV (r=-0.0201), illustrated by the quite distinct age-dependent seroprevalence curves for DENV and CHIKV (Figure 2). Further a random sample of 92 plasma samples from the same cohort taken in 2013 (i.e. before the appearance of CHIKV in Ecuador) showed no positive reactions using the same commercial assay, indicating sufficient specificity in reference to DENV. Our data clearly show the differing epidemiology of the two viruses in this population - the endemic nature of DENV as opposed to the 'stochastic' age-seroprevalence profile for CHICKV – justifying the approach to address co-exposures and their epidemiological determinants. This approach allowed us to make observations specific to each virus that, although they share the same vector, differ with respect to numbers of human infections caused year-on-year depending on unknown factors such as vector competence, viral interference within vectors, and the density of susceptible humans.

Comment 6: Previous studies have looked at estimating seroprevalence by age in settings with co-circulating arboviruses (e.g. I. Rodriguez-Barraquer, S. S. Solomon, P. Kuganantham, A. K. Srikrishnan, C. K. Vasudevan, S. H. Iqbal, P. Balakrishnan, S. Solomon, S. H. Mehta, D. A. Cummings, The Hidden Burden of Dengue and Chikungunya in Chennai, India. PLoS neglected tropical diseases 9, e0003906 (2015).).

What is the novel research question this paper is addressing, relative to previous studies? This could be clarified in the manuscript.

Response 6: We thank the reviewer for highlighting this interesting reference which we overlooked in our previous submission. Of course, the study to which the reviewer refers was done in a different population in a different geographic region (Chennai, India) with different infection dynamics. This paper by Rodriguez-Barraquer et al., addressed different study questions (i.e. key infection dynamics parameters such as force of infection and R_0) using serological data. The authors use survey data to fit two *separate* models to the two outcomes (DENV and CHIKV) where there was a clear underlying correlation between the two outcomes. The use of the novel analytic approach described in our manuscript offers clear

advantages over such an approach. However, this study has now been included (Reference 22) in our resubmitted manuscript.

Comment 7: *The results regarding the determinates of risk of past exposure could be given more attention in the paper main text, as I find them quite interesting. Perhaps the authors can add a summary table with odd ratios for each risk factor (e.g. I. Rodriguez-Barraquer, S. S. Solomon, P. Kuganantham, A. K. Srikrishnan, C. K. Vasudevan, S. H. Iqbal, P. Balakrishnan, S. Solomon, S. H. Mehta, D. A. Cummings, The Hidden Burden of Dengue and Chikungunya in Chennai, India. PLoS neglected tropical diseases 9, e0003906 (2015).)*

Response 7: The information which the reviewer requests was provided in Table 2 of the original submission. We thought that these conditional ORs might be difficult to conceptualize for the non-specialist reader and, in addition, provided predicted prevalence across the groups in the population in the supplementary and use that interpretation of the results for the general reader.

Comment 8: *It would also be interesting to see how the predicted seroprevalence varies spatially in the study region. Dengue transmission is highly spatially heterogenous and a map of predicted seroprevalence might give some idea of the sources of this heterogeneity. Please see for example the Rodriguez-Barraquer papers above cited.*

Response 8: We thank the reviewer for this suggestion. We have we added region associated numbers and their corresponding predictions in the last figure associated with the *Response 2* to the *Comment 2* above. as mentioned above. However, the Rodriguez-Barraquer study was done on a much larger geographic area with refined predictions possible given large numbers. We do not believe that such an approach would be appropriate in our own given the much low numbers per spatial location. However, we have added relevant figures corresponding to goodness-of-fit as described in detail above.

Comment 9: *It would be also useful to highlight more the implications of the study for development of control strategies against dengue and chikungunya. How can we use the results of this study (predicted seroprevalence by age) to inform how effective alternative control strategies can be? It might be difficult, due to the observed variation in seroprevalence with age, which is also picked up by the model used by the authors. One advantage of fitting age-stratified data is that it allows to calculate the force of infection (per capita rate at which susceptible individual acquire infection). Force of infection can be used to infer the reproduction number* ***R0 (average number of infections caused by one infectious individual)****, which can be used to predict the potential impact of control strategies, such as vaccination (recently addressed in L. Cattarino, I. Rodriguez-Barraquer, N. Imai, D. A. T. Cummings, N. M. Ferguson, Mapping global variation in dengue transmission intensity. Sci. Transl. Med. 12, (2020).)*

For peer review only - http://bmjopen.bmj.com/site/about/guidelines.xhtml

Response 9: These comments regarding fitting a model to aggregated age-stratified data have been addressed in our previous responses - we reiterate our aim at making full use of our valuable individual-level information.

We agree that the key epidemiological measures to understand infection dynamics is the force of infection – all other measures including transmission parameters are derived from it. We are also aware about the definition and relevance of the R_0 as key dynamic descriptor of an epidemic. However, **dengue is not transmitted between people – this is a well-known vector-transmitted disease and the reviewer's definition of R_0 is *incorrect*** (Estimating R_0 in this context of vector borne transmission involves extra complexities requiring data that are unavailable in our setting such as information on mosquito density and competence in the population).

Summarising: the question of disease dynamics is just different from our study question and we feel that the request to conduct a different body of work which is enough to generate a separate research communication on its own, is unreasonable.

We did add this new reference (Ref no 22 in the current version) stating in the discussion that "*Highlighting areas of high prevalence is important as recent analyses suggested that transmission-blocking interventions such as Wolbachia, even at intermediate efficacy (50% transmission reduction), might reduce global annual disease incidence by up to 90%. The newly licensed Sanofi-Pasteur vaccine, targeting only seropositive recipients, (namely those at risk of secondary infection which can result in severe dengue and subsequent death), might reduce global annual disease incidence by 20 to 30%, with the greatest impact in high-transmission settings*"

Additional comments in response to Reviewer 1:

The request of fitting a model to aggregated data (i.e. data grouped by age) is somewhat at odds with seeing a goodness-of-fit by means of a "*plot of predicted individual-level seropositivity by age with overlaid the observed individual-level seropositivity values*". Individual-level inferences made using models fitted to aggregated data can be deeply flawed as recognised by the *ecological fallacy*.

Given our individual-level information in the data, we feel that an aggregated modelling approach would waste valuable information that allows us to draw inferences.

As discussed in the previous research mentioned by this reviewer (Rodriguez-Barraquer et. al. above), serological data might be the best answer to the question of disease dynamics in this context of vector transmitted virus but we believe that it is very well suited to answer the question of prevalence of past exposure in the population given the long-lasting immunity

For peer review only - http://bmjopen.bmj.com/site/about/guidelines.xhtml

these viruses following exposure. The method developed by our paper is robust, account for the potential effect of unmeasured variables and embeds population-adjusted weighting which allow generalization of the results to the region of coastal Ecuador.

Further, such "*plot of predicted individual-level seropositivity by age with overlaid the observed individual-level seropositivity values*" would not allow us to achieve evidence of goodness of fit as the outcomes are binary with predicted probabilities between 0 and 1. We have clarified this issue by starting with the simplest case of a logistic regression where the Hosmer-Lemeshow measure of goodness of fit involves grouping the data and calculating the expected numbers of positive individuals across groups – however, the classical method applied to independent data no longer holds for hierarchical data. We had addressed goodness of fit in a Hosmer-Lemeshow manner in our original submission by plotting raw numbers for each type of response (D+C-, D-C+, D+C+) against expected numbers by age group, which might, perhaps, have been overlooked by the reviewer. We have added in this resubmission, a section dedicated to goodness of fit in supplementary files with further clarifications, and have more added also observed vs. predicted plots including by geographical region within the study district. We show that our model provides a good fit to the data considering the inherent limitations of a fully parametric parsimonious model fitted to our data set. We have added also group bars using raw data in Figure 2 as requested by the reviewer.

Reviewer 2 generic comments:
Reviewer Name: M. Celeste Torres
Institution and Country: Oswaldo Cruz Institute - FIOCRUZ, Brazil
Please state any competing interests or state 'None declared': None declared

Comment 1: The majority of my comments are included in the attached file. But there are particular main points that I feel are necessary to reinforce. English needs to be improved. Through this, the manuscript comprehension would be significantly facilitated. Spelling must be checked as well.

The objectives and hypotheses are not clearly defined. The methodology is not entirely clear, nor the study population correctly described. The methods and results are very mixed. It needs to be restructured.

For peer review only - http://bmjopen.bmj.com/site/about/guidelines.xhtml

Response 1: We have edited the English in the manuscript to improve clarity as requested and corrected some typos present in the previous version. We also provided a clear description of the hypothesis underlying this analysis in the final paragraph of the Introduction which reads now:

"In this study, we hypothesised that prevalence of past exposure to DENV and CHIKV in the population are associated with age, and individual and household risk factors. We estimated age-dependent seroprevalence of DENV, CHIKV and co-exposures with both arboviruses in a district in coastal Ecuador, and explored individual and household determinants of past exposure status."

A deliberate decision was made to separate the somewhat complex statistical modelling into supplementary files while maintaining the epidemiological question relating to co-exposures to the two arboviruses in the 'core' manuscript. In this way, the non-specialist reader can follow the basic analytic methodology and key results and refer if necessary to supplementary files. The novel but somewhat complex statistical methodology provided in the supplementary files are more likely to be of interest to advanced statistical modellers who may wish to use the methods described in their own research as well as using the code. We have addressed all the reviewer's comments with corresponding changes in a tracked revision of the modified manuscript.

Comment 2: About the serological tests, I am very concerned about the level of confidence that can be achieved, particularly for dengue. As I commented in the manuscript, cross-reaction for flaviviruses is well known and a big issue for these techniques when it comes to diagnosis, for example. There is a commercial kit that crosses even between DENV and CHIKV. At anyway, ZiKV and YFV can be influential for DENV indirect serological tests. Besides, in low-prevalent scenarios (as can be for CHIKV in 2016), the positive predictive value of the test may decrease, leading to a less confident result. In this regard, and considering as well that serological results would be introduced into the modelling, shouldn't the authors be 100% sure that results obtained are true positives?

Response 2. We agree that there is likely to be at least one (of many commercial kits available) for which cross-reactivity between DENV and CHIKV is an issue. Please also see Response 5 to Reviewer 1 above. The commercial assays we used have been validated and show minimal cross-reactivity between flaviviruses and alphaviruses. Particularly for DENV there may be issues of cross-reactivity with other flaviviruses such as Zika and yellow fever. However, the study was done before the emergence of Zika virus in this coastal Province and where there have been no reported human cases of yellow fever for over 50 years. The antigen used for coating plates for DENV was purified Dengue 2 virus proteins from strain 16681, cross-reactive across dengue serotypes and likely also to be cross-reactive with other flaviviruses but not the alphavirus CHIKV. Plates for detection of CHIKV specific-IgG were coated with recombinant envelope protein 2 of CHIKV which appears to be highly specific for the detection of this virus even among other alphaviruses (Kam et al. PLoS Negl Trop Dis 2015; 9: e3445; Fumagalli et al Virol J 2018; 15: 112). Further there was no evidence of a correlation between OD values for the IgG responses to DENV and CHIKV (r=-0.0201),

For peer review only - http://bmjopen.bmj.com/site/about/guidelines.xhtml

illustrated by the quite distinct age-dependent seroprevalence curves for DENV and CHIKV (Figure 2) and limited cross-reactivity. Further, testing of samples from the same cohort from 2013 (i.e. before the appearance of CHIKV in Ecuador) showed no positive tests for CHIKV but a similarly high prevalence of DENV, making significant cross-reactivity between DENV and CHIKV unlikely. Unfortunately, few if any serological tests are 100% specific. There will always be a small proportion of false positives (likely to be low as indicated by the previous observations). Such measurement errors, however, are unlikely to be systematic and will reduce the precision of the measurements and power to show an effect, but they do not invalidate the effects that can be shown.

Comment 3: Concerning the model itself, I could not understand in detail how was it applied. It would be interesting to better explain at least its fundamentals in the methodology section. Besides, being these arboviruses infections multifactorial, I am wondering if this modelling should not also include other variables concerning ecological (climate, vector, etc.) and clinical (fatality rate, asymptomatic estimations, etc.) parameters.

Response 3: We agree that the statistical modelling approach is complex which is why we have provided more complex methods in the supplement (see Response 1). We have changed the presentation in the statistical methods which we hope are now much clearer and have added further clarifications to the supplementary material.

Unmeasured variables that may influence the prevalence of past exposures including climate and vector-related factors are accounted for in the random effects. Unmeasured asymptomatic infections are captured within seroprevalence data given that these viruses induce long-lasting immunity: thus, a strength of using serological data. Most modelling papers on DENV and CHIKV viruses make this assumption given the low figures for case fatality (death ration among positive individuals). Furthermore, fatality ratios are related to severe dengue (references [35,36,40,41] are very informative in this sense). In a study in Brazil, only 1% were diagnosed with severe dengue out of which only 4% died (that is less than 1% of all, i.e. 0.04%).

The assumptions underlying our modelling approach are reasonable and not new in relation to this type of modelling. Moreover, the ability to account for mortality (a rare event) is only possible with data from very large samples. The same is true to a lesser extent of individual clinical characteristics, not of relevance to our specific study question. Only the most important parameters which drive an epidemiological process could be robustly estimated from our data (e.g. prevalence of exposure by age and relevant individual/household). Our data provide useful information for policy makers with respect to, for example, the potential feasibility of dengue vaccination.

Comment 4: The exposed results appear to be in line with those raised in methodology; however, as stated above, it is necessary to make adjustments on them to improve their

For peer review only - http://bmjopen.bmj.com/site/about/guidelines.xhtml

interpretation. Particular comments can be found on the attached file.

Response 4: Please see below our complete responses to the specific comments below and in the resubmitted manuscript.

Comment 5: Although the discussion assessed the main results and limitations of this study, I feel it is necessary to make a more in-depth analysis of the results obtained in this study, including a potential comparison with different serotypes of DENV and other arboviruses in the region (they make a brief mention that is not enough). To base all conclusions just on these two viruses, and not considering DENV as four different serotypes, I do not believe that it correctly reflects what is actually happening. I also feel that they need to be further elaborated on regarding the public health significance of these results and how vaccine implementation could interfere (or not) in the results here presented.

Response 5: We thank the reviewer for acknowledging our discussion of the main results and limitations of this study. We agree that an analysis of DENV-serotype responses would be of interest in this population but such an analysis is beyond the scope of the aims of the present study. The four DENV serotypes have been reported to be present in Ecuador since 2000 (Ramos-Castañeda et al. PLoS Negl Trop Dis 2017), and different outbreaks in space and time are likely to be caused by different serotypes/genotypes. The analysis of serotype-specific responses, not directly relevant to the specific aims of our project, would require a much larger sample to be assessed adequately, a different type of sample relevant to this specific question, and use of more specialized and specific serologic assays (plaque reduction neutralization assays) that are not presently done by any laboratory in Ecuador. Our aim in the present study was to define seroprevalence of DENV irrespective of serotype to allow us to estimate overall exposure to DENV. We are not aware of the circulation of other arboviruses in this region at the time this study was conducted. Zika virus appeared in the region after sample collection was done; the first cases of Mayaro virus infection in coastal Ecuador were identified in 2019 (https://www.salud.gob.ec/se-confirman-los-5-primeros-casos-con-el-virus-mayaro-en-el-pais/); and the first case of Oropouche virus in Esmeraldas Province was identified in 2016 (Wise et al., Emerg Infect Dis 2016). These observations don't indicate significant circulation of other arboviruses in the study area at the time we conducted the study. We address the vaccination issue with additional comments and references. We have appended a new paragraph (below) to the discussion in the light of a novel (2020) publication added to the references list (#22 in the list). The research presented there did not account for different DENV serotype either.

"Highlighting areas of high prevalence is important as recent analyses suggested that transmission-blocking interventions such as *Wolbachia*, even at intermediate efficacy (50% transmission reduction), might reduce global annual disease incidence by up to 90%. The newly licensed Sanofi-Pasteur vaccine, targeting only *seropositive* recipients, (namely those at risk of secondary infection which can result in severe dengue and subsequent death), might

For peer review only - http://bmjopen.bmj.com/site/about/guidelines.xhtml

reduce global annual disease incidence by 20 to 30%, with the greatest impact in high-transmission settings"

Reviewer 2 detailed comments from the pdf file annotated by the reviewer

Please note that the comments are identified by the page number, as labelled by the journal editorial on the top of the page, alternatively on the right/left corner, and line number, as labelled by the journal editorial on the left of that page.

Page 4, Line 19-20:

Authors meant the statistical method?
Response: Yes, we have change as requested – it is now "…the statistical technique"

Page 6, Line 6:

This might not be consistent with the last paragraph of the manuscript. Please, check it up.
Response: We have changed the last statement for consistency with this paragraph. We have now introduced a Patient and Public Involvement statement which reads:

"The cohort was initially set up to understand how early childhood infections affect the development of inflammatory diseases in childhood. Participants were clearly explained the public health benefits of the study prior to recruitment and informed written consent was obtained prior to recruitment and at several stages during the course of this longitudinal study (e.g. at 5 and 8 years also)."

We have also altered the Ethics statement which reads:
"Informed written consent was obtained from participants aged 18 or more, and from a parent or legal representative for a minor. Minor assent was obtained from children aged 8 years or older. The study protocol was approved by the Ethics Committee of the Universidad Internacional del Ecuador, Quito, Ecuador."

Page 7, Line 11:

dengue=disease
DENV= dengue virus
chikungunya=disease
CHIKV= chikungunya virus

It would be interesting to denote as well, that even though they are both arboviruses, they belong to different families and genres.
Response: Thank you – the terminology is important. We have added to the text the viral families to which these arboviruses belong and an additional reference [1]. The paragraph is changed to:

"Dengue virus (DENV) and chikungunya virus (CHIKV), arboviruses belonging to *Flaviviridae* and *Togaviridae* families, respectively, are endemic in many tropical and sub-tropical regions where *Aedes* vectors thrive."

For peer review only - http://bmjopen.bmj.com/site/about/guidelines.xhtml

Page 7, Line 40:

Case notifications may also include cases detected by other diagnosis techniques, or just clinically, etc. Unless serosurveys get implemented and widely applied to a population, I don´t see how they can be comparable to case notifications.

Response: Thank you We hope we added some clarity to this statement. We changed the terminology from case-notification to clinically classified. The paragraph reads now:

"Cross-sectional serological surveys can provide valuable information on arbovirus infection burdens and allow more accurate determination of numbers exposed. Serological surveys, thus, have a number of advantages over clinically classified, self-reported cases or other non-objective clinical diagnoses by providing information that are less sensitive to recall, underreporting, and misdiagnosis due to asymptomatic manifestations of related infections".

Page 8, Line 43:

It is understood from this statement that your particular sub-cohort included children recruited during the period 2006-2009.
Consider re-writing this section. Go from the general to particular... otherwise, it gets really confusing.

Response: We have hopefully clarified the statement adding "children and their households."

Page 9, Line 13:

Please add a different subheading for this paragraph

Response: We have now added a paragraph dedicated to laboratory testing and name it as such, just before the Statistical methods section.

Page 9, Line 13

If they were already abbreviated before, then use DENV and CHIKV

Response: We have addressed that.

Page 9, Line 16-18 (repeated comment)

Can exposure be correlated directly to seropositivity?
What about non-responders? Incubation window?
What about seropositivity due to cross-reaction? Even more considering flaviviruses, zikv infection for exm.

Response: The serum data collection took place in the aftermath of the 2015 epidemics in Ecuador. We have defined seropositivity by the means of presence of viruses' specific antibodies and subsequently assumed that seropositivity indicated past exposure to those viruses. There is a great body of literature in modelling these viruses which are based on the assumptions of long-life immunity following exposure, many of which are cited in this paper.

For peer review only - http://bmjopen.bmj.com/site/about/guidelines.xhtml

Page 9, Line 29-30

Presence/absence of DENV or CHIKV would be referring to the direct detection of the virus, which is not the case. Please be aware of this. I guess authors meant antibodies against DENV or CHIKV, or evidence of past dengue or chikungunya infection. This should be clarified.

Response: This is precisely the case - we have modified the paragraph in the light of this comment and started with the definition of the statistical outcome or the dependent variable as we call it. The beginning of the Statistical Methods section reads now as:

"The presence or absence of DENV and CHIKV antibodies define a bivariate binary outcome indicating past exposure to DENV only (D+C-), to CHICK only (D-C+), to both (D+C+) or to none. The key assumption of the model is that of long-lasting immunity [25] after infection with DENV and CHIKV."

Page 9, Line 29-34

Not clear to me. Which model? The multilevel logistic regression? So, actually, it is an existing model you are adapting to arbovirus infections modelling.

Response: The very basic model we refer to can be found in the reference [26]. Our model is not a simple adaptation of that model. Adding two levels of correlated random effects associated with potentially unmeasured variables at household and ward level, including mosquitos' burden and bites' intensity, water presence around the house, weather, or ward population dynamics in general brings a series of complexities in the approach.

Page 9, Line 41

Which ones? It will be clearer if you present first what are the variables you are taking into account in the model. It is difficult to understand what you are trying to determine using this model otherwise.

Response: We have changed the statistical methods section to quite a greater extent hoping that it flows better. We have moved the variables taken into account in the model after clarifying the definition of the outcome (the dependent variable).

Page 9, Line 50

It is not clear to me, age-dependent prevalence isn´t one of the parameters being estimated, using the serology and household info? How is it that it can infer co-exposures in the past if CHIKV was introduced in 2015?

Moreover, how are you managing the different DENV serotypes infections and the respective serological response?

Response: Age dependent prevalence is a concept in epidemiology which refers to the risk of disease (infection) according to age (can be age groups) in the population. It

For peer review only

usually answers the question of how the disease risk varies with age and what are the factors which confound this potential association between disease and age.

In this particular context, of viral infections which result in long-life immunity, we are talking about the age-dependent prevalence of past exposure to those viruses. This can be thought as the age-dependent prevalence of immunity to those viruses in the population. This is a very important aspect to understand at population level as various control measures can be implemented effectively, i.e. targeting groups at most risk.

Page 10, Line 3-13

It is not clear at all for this reviewer what are you infering from this. You have the serological test result plus patients age information. These are not being infered. Which predictions were plotted?

Response: I think there is some confusion on what inference concept means in statistics. Indeed, we have serological test indicating past exposure to DENV/CHIKV results and patients age information. Our model develops a systematic trend, numerically quantified association between age and the risk of past exposure to these viruses as explained above. Moreover, proper statistical inference assigns uncertainties (the 95% credible intervals) around the numbers which express numerically this association. Accounting for the rest of the variables can change this association and the associated uncertainty.

Predictions are precisely those curves in Figures 2 and 3 - that is how our model infers numerically the risk of past exposure (or the prevalence of immunity) in the population.

Page 10, Line 20

This paragraph is crucial. It should be presented before.

Response: We reshuffled the statistical methods paragraph to a quite great extent.

Page 10, Line 35-39

If applying this model to DENV/CHIKV infection is the key point that authors intend to show, they should consider including the model parameters and the approach employed in the main text, leaving the accessory processing for the supplementary information.

Response: We are not sure if we can make sense of this request. But we think the paper flows better with a lay presentation of the methodology and the mathematics and associated technicalities presented in the Supplementary information.

For peer review only - http://bmjopen.bmj.com/site/about/guidelines.xhtml

Page 10, Line 35-39

This is not the same. To be at risk doesn't necessarily mean to get infected and thus, to seroconvert.

Response: This is not what we state at all, i.e. **to be at risk**. Here we are talking about the risk of past exposure to these viruses which is a very different concept from that of being at risk. The sentence has changed for clarification to: "Given the assumption of life-long immunity, age-dependent seroprevalence and risk of past exposures to DENV and CHICKV, are used interchangeably."

Page 10, Line 52

Please reformulate. There were 2404 children enrolled in the birth cohort, but only some of them participated in this particular study. This sub-group plus their relatives sharing household represent the 319 individuals of this study. Am I right? Population under study is not clear and needs to be better defined. Since the birth cohort is not the main focus of this analysis, I would recommend avoiding its continuous citing, cause it is confusing.

Response: We did reformulate. We think that it is important to cite the background cohort – it is one of the important features of this research. It now reads as "**Error! Reference source not found.** shows the geographical locations of households of children in the background cohort and the locations of those sampled for this analysis (151)."

Page 10, Line 56

Did the authors determine this information? If they did, then how was it determined? If they didn't, then please consider moving it from this section to the introduction, or discussion, and adequately reference it.

Response: This is part of the results and represent the cumulative number derived by summing weekly reported infection using publicly available websites. The numbers are not part of our modelling strategy but it gives the readers perspective into the extent of the 2015 epidemics.

Page 11, Line 8

There is no need to present the information as a table and as a graph. I would strongly recommend choosing one of them.

Response: Table 1 presents cumulative numbers and Figure 1 presents the epidemic curves, i.e. the weekly number of cases. The total (cumulative number) is not informative enough for infections of relatively short duration and short dynamics as it would be in case of chronic diseases outcomes. Weekly incidence is necessary to be reported too so the reader can understand the course of the epidemic.

For peer review only - http://bmjopen.bmj.com/site/about/guidelines.xhtml

Page 11, Line 11

319 children? or 319 children+household contacts?

Response: The question has been addressed above.

Page 11, Line 11

from 49 wards?

Response: yes – that has been clarified in the text.

Page 11, Line 13

The word baseline seemed out of place here. Or do the characteristics change at any point?

Response: We have removed it.

Page 11, Line 18

High proportions of...? Consider removing vague terms. Use instead real numbers.

Response: We have changed it accordingly and referred to numbers too.

Page 11, Line 42-49

Unclear. Please reformulate. By estimations, authors mean the calculated probability? It is still confusing for me what are the authors trying to model.

Response: The ORs (the estimates) are measures of association between the presence of DENV or CHICK antibodies (past exposures to these viruses) with age, sex, etc. The sentence changed to: "*Parameter estimates (conditional odds ratios) which measure the univariate and adjusted associations of the risk of testing positive for DENV, CHIKV and both with each potential risk factor (age, sex, area of residence, socio-economic status and distance to next door neighbour) are shown in Table 2.*"

Page 11, Line 49-55

Difficult to understand.
Besides, why are the authors talking about "predicted seroprevalence"? Didn't they obtain that information directly from the ELISA test?

Response: We think the confusion stems for similar terminology used in laboratory environment and statistics. In this context, the Elisa tests (probably by some prediction methodology which has a completely very different meaning from that of statistical prediction) provided the raw information which defined our binary bivariate outcome or dependent variable as we call it.

Page 11, Line 58

For peer review only - http://bmjopen.bmj.com/site/about/guidelines.xhtml

Abbreviations must be explained the first time they are used. CrI= confidence interval? It is commonly abbreviated as CI, as authors mentioned in the methodology section. Please be consistent, CrI or CI.
On the other side, by average, you meant the mean? The median? How did you get to the percentage value? Did the authors suggest that 84% of the individuals of this cohort tested positive for dengue? If so, the word average is confusing. Consider removing it.
Moreover, which ages define what an infant is? 5 years-intervals stratified ages.
Response: In Bayesian inference, the credible intervals play a similar role as confidence intervals in classical statistical inference – that of assessing the uncertainty of an estimate. Confidence Intervals (usually denoted by CI) are however different concepts from Credible Intervals. We clarified that we referred to credible intervals usually denoted by CrI.

Page 12, Line 6

Not obvious from the graph. Seemed to be before 10.
Response: We have corrected that to around 9 years.

Page 12, Line 11-14

What's the difference between Fig2 and Fig3 D+C- / D-C+??
Response: Figure 2 presents the predicted (from the model) age-dependent prevalence of all DENV and CHIKV past exposures whilst Figure 3 presents the predicted (from the model) age-dependent prevalence for past exposure to DENV only, to CHIKV only and to both only. The y-values on continuous line in Figure 2 are the sum between the y-values between the first and the third panel in Figure 3 (DENV) and the sum between the y-values between the first and the second panel in Figure 3. Both are of epidemiological interest.

Page 12, Line 14-21

I must assume that serological test results were introduced in your model, and then you compare estimations with real serological testing results. However, this is not clear through the manuscript. I would strongly recommend to improve the writing and reformulate the methodology section so to clarify how this model is applied and which is its purpose.
On the other hand, how is it possible to be predicting a probability lower than 3% for D-C+ but between 8-50% for D+C+?
Response: The reviewer is referring essentially to the goodness of fit. We have romped up the goodness of fit tailored to this model, inspired by the basic logistic regression. We add a section in the Supplementary and provided extra clarity on this.

Regarding the second request, the results regarding CHIKV proves that figures associated with past exposure to CHIKV only are actually low. The D+C+ figures of 8-50% reflects the high burden of co-circulation of the two viruses in 2015.

For peer review only - http://bmjopen.bmj.com/site/about/guidelines.xhtml

Page 12, Line 52

vague term

Response: "Less precise" is a term used in statistics when the uncertainty (CrI) is larger. Usually, the larger the number of variables involved in an epidemiological process, the larger the uncertainty. In other words, more information but less precise. The uncertainty associated with an estimated parameter is equally important as the parameter itself.

Page 13, Line 13

Which one?

Response: All of them – the sentence has been changed.

Page 13, Line 17

No y-axis title in suppl fig 8 nor 9.

Response: That has been fixed.

Page 13, Line 42-44

Which ones? Not clear what authors meant to say.

Response: This is a generic statement, i.e. "Nevertheless, the epidemiological and statistical methodologies can straightforwardly accommodate for more multiple concomitant exposures". We did not have to mention which – we wanted to highlight the flexibility of the methodology to extend to outcomes which can reflect past exposures to multiple related viruses.

Page 13, Line 48

This reference doesn't correspond to the stated information

Response: We think it does. The work contains worldwide maps with countries where serological studies have been conducted and the Ecuador map indicates no serological information. (Fritzell, C., et al., *Current challenges and implications for dengue, chikungunya and Zika seroprevalence studies worldwide: A scoping review*. PLoS Neglected Tropical Diseases, 2018. **12**(7))

Page 13, Line 51-54

Seems to be somehow expectable considering that DENV is endemic in Ecuador and 0 to 60 yo is a wide range.

Response: We are glad that our findings reflect those of empirical observations. As mentioned above, estimates and associated uncertainties are important to inform effective control strategies.

For peer review only - http://bmjopen.bmj.com/site/about/guidelines.xhtml

Page 14, Line 13

Interesting. Please cite all corresponding studies.
Response: the references are correctly assigned. We are bounded to a max number of 40 references and already exceeded it. We chose references which provided a comprehensive review of the existing literature.

Page 14, Line 27

This is not the correct reference. Please cite the real ones referencing the mentioned studies.
Response: Please see above

Page 14, Line 50-51

It is expected to find a lower prevalence for CHIKV exposure (compared to DENV), since the first outbreak was evidenced in 2015 and sampling of this study was between May and Sep 2016.

Considering this, how can the authors trust in the results obtained for Chikv+ estimations?
Response: Exposure to CHIKV results in the presence of specific antibodies and therefore a seropositive individual was considered as exposed to CHIKV at some point in time prior to data collection.

Page 14, Line 56

Also, consider that the region under study was defined as a rural area. Thus, it would be expected to detect a difference for male gender due to field activities.
Response: Table 1 indicated quite the contrary, 68% of households were deemed as urban and the rest were rural. We think that the interpretation of the results is informative.

Page 15, Line 10-13

Two comments in this regard:
**Vectorial competence should be considered in this study since it is focusing on a short period when two simultaneous outbreaks were taking place. Mosquitoes populations could have been more competent to transmit CHIKV rather than DENV.*
**On the other side, in the region under study DENV cases were considerable lower than CHIKV ones. A previously immune population would likely difficult or at least decrease DENV transmission (considering same DENV serotype).*
It would be interesting to extend the epidemiological data presented about these two viruses, and other relevant arboviruses as well.

For peer review only - http://bmjopen.bmj.com/site/about/guidelines.xhtml

As an example, while Brazil was experiencing a huge DENV2 outbreak last year, the state of Rio de Janeiro presented just a few cases, but a massive chikungunya outbreak instead. Several studies had proposed/demonstrated how vectorial competence is involved in different arboviruses cycles, together with the immune status of the population.

Response:

Evaluation of vectorial competence would require collections of vectors and molecular analyses during the outbreaks for the presence of the two viruses, as well as more sophisticated laboratory-based studies using A. aegypti. In this analysis, we used a study sample with blood collection after and not during the outbreaks. We measured levels of IgG that, after a first exposure, would require a 30-days or so to reach maximal levels and which would tell us about past exposure. Although we agree that studies of vector competence during simultaneous outbreaks of DENV and CHIKV are of scientific interest, they are beyond the scope of the present analysis and study sampling. We have added a statement to the Discussion on the value of studies of vector competence t our understanding of the evolution of two infection occurring simultaneously in time and through the same vector.

Page 15, Line 46

This is a strong statement.

Response: Replaced “unreliable” with “uncertain”.

Page 15, Line 48-53

Authors should take care with this kind of statement, and consider toning it down since serological surveys are extremely useful to determine the prevalence of a disease. Still, they may not entirely represent the impact/burden that the disease may represent. To start with, dengue, for example, presents high levels of subclinical infection. Moreover, a vaccine against DENV was implemented in different countries, which can raise the population seropositivity.

Otherwise, please provide justifying evidence.

Response: We are not entirely sure if we understand this comment. We stand by our original statement which advocates the usefulness of serological surveys as a powerful tool to understand population past exposure to viruses. In case of subsequent long-lasting immunity, they provide a measure of immunity in the population.

On the other hand, to our best knowledge there has been a long debate regarding most effective strategies for DENV control including vaccination. There are very new guidelines in this sense – two very novel and informative papers [20, 21] published in 2019 and 2020 are cited here. One of them (The Lancet) was cited in the original submission.

Page 16, Line 42-53

This information should be presented in the methodology.

For peer review only - http://bmjopen.bmj.com/site/about/guidelines.xhtml

The authors mentioned that this modelling would contribute to these arboviruses burden estimations. However, if the fatality rate is dismissed even for denv infection, wouldn´t the final predictions be biased, or underestimated?
It seems to me that both asymptomatic infections and fatality cases should be taken into account.

Response: The case fatality ratios appear to be low in the population as presented in references [35, 36]. Most modelling papers on these viruses made this assumption of negligible mortality given these low figures for case fatality ratios. Furthermore, fatality ratios are related to severe dengue (references [40,41] are very informative in this sense). In a study in Brazil, only 1% were diagnosed with severe dengue out of which only 4% died (that is less than 1% of all, i.e. 0.04%)

We clearly set out our assumptions which are reasonable and not new in relation to this type of modelling.

Having said this, unless operating with very large numbers of individual data, it is impossible to account for mortality, DENV serotypes and all otherwise important biological aspects. No matter how complex an epidemiological process in a population, only the most important parameters which drive that process can be robustly estimated from the data. In our research setting, that is the prevalence of past exposure by age of two important co-circulating viruses and a series of important population characteristics which would inform policy makers for effective vaccination.

Page 16, Line 53

Even more, I feel that the four serotypes should be modelled separately. Perhaps, immune response for heterotypic denv infections should be considered too, since non-neutralizing antibodies when in a certain titer, may cause ADE. The following publications could be helpful:
**Katzelnick LC, Gresh L, Halloran ME, et al. Antibody-dependent enhancement of severe dengue disease in humans. Science. 2017;358(6365):929–932. DOI:10.1126/science.aan6836*
**Salje H, Cummings DAT, Rodriguez-Barraquer I, et al. Reconstruction of antibody dynamics and infection histories to evaluate dengue risk. Nature. 2018;557(7707):719–723. DOI:10.1038/s41586-018-0157-4*

Response: This question has been addressed above to some extent. Population studies are different from laboratory studies. Serological studies combine them but it is impossible to accurately identify patterns in the populations accounting for all biological aspects in the lab. Large amounts of detailed laboratory data can be logistically difficult particularly in low-middle income countries. But this is what statistics do – from a relatively small sample which represents a population, through appropriate modelling, derives conclusions valid for that population. Only the most

For peer review only - http://bmjopen.bmj.com/site/about/guidelines.xhtml

important questions can be estimated with reasonable precision. For example, some estimate of a proportion of 0.5 with a CI between 0.01 and 0.99 is equivalent with *no information* at population level on what that proportion can represent.

Page 16, Line 58-60

Not clear.

Response: The reference [40] is very informative for case fatality ratios.

Page 17, Line 17-20

This is not a minor issue. I strongly recommend the authors to include epidemiological data of ZIKV in Ecuador; even better if there is any from the region under study. Serological cross-reaction is one of the main concerns about these tests. It should be taking into account. Mostly for flaviviruses. What about YFV?

Response: Our methodology accommodates for past exposure to a third related virus. At the time we started analyses of this data we did not hold serological results associated with Zika virus. Having said this – it is not straightforward to analyse samples in the lab and to re-analyse the data. It would be another year of work at least. But we do have plans in these regards.

Page 17, Line 30-34

But this information comes from the serological tests + patient's data and seems to be representing the mere epidemiology of both, DENV and CHIKV infections.

Response: In the light of the new vaccination guidelines, simply said, we put numbers to assess this dependence. This is always important control strategies may be costly and in the absence of population level predictions choosing the most effective strategy is impossible.

Page 18, Line 34

Please adjust font and size to correlate with Age column

Response: We fixed that.

Page 18, Line 54

How did the authors calculate this? Which is the reference point? Total n should not be 151? Didn't the authors take each house as a reference and measure the distance to the next most proximal one?

Response: We made the necessary corrections.

Page 20, Line 6 (in the Table 2)

For peer review only - http://bmjopen.bmj.com/site/about/guidelines.xhtml

What does power mean?

Response: We added clarity in the Table 2 legend. The power is a parameter to be estimated of the age ^{power} component of the model. For example, that can be age ^{2}. We estimated the power as 7.6 – that is translated in a very fast increase of past exposure to DENV with age - see Figures and Table 2.

Page 20, Line 56

this would be the prevalence....

Response: We disagree on the relevance of a proportion of cases on a particular year associated with this type of infections.

Page 22, Line 4

I would recommend to re-write this figure title
Response: Spatial distributions of the household locations of the background population (ECUAVIDA) and the serological sample.

Page 22, Line 31-36

17-20

Response: We left each reference in case the original order is changed.

Page 25, Line 8-9

Patient's samples and data are involved. Do you have a written consent from them? Since this study includes children, a legal representant must allow for using all this information. If waived, please inform it. Also authors would need to include a statement referring to the ethical comitee aproval.
Response: We have now introduced a Patient and Public Involvement statement which reads:

"The cohort was initially set up to understand how early childhood infections affect the development of inflammatory diseases in childhood. Participants were clearly explained the public health benefits of the study prior to recruitment and informed written consent was obtained prior to recruitment and at several stages during the course of this longitudinal study (e.g. at 5 and 8 years also)."

We have also altered the Ethics statement which reads:
"Informed written consent was obtained from participants aged 18 or more, and from a parent or legal representative for a minor. Minor assent was obtained from children aged 8 years or older. The study protocol was approved by the Ethics Committee of the Universidad Internacional del Ecuador, Quito, Ecuador."

Page 34 (Figure)
y axis?

For peer review only - http://bmjopen.bmj.com/site/about/guidelines.xhtml

Response: We fixed that

Page 38 (Figure)

x axis?

Response: We fixed that

Page 47, Line 29

variables need to be define at first instance.... what is R, what is K, etc. What are they representing?

Response: We think that the supplementary information reads very well – there are clear statements on what R, K represent.

For peer review only

BMJ Open

Age-dependent seroprevalence of dengue and chikungunya: inference from a cross-sectional analysis in Esmeraldas Province in coastal Ecuador

Journal:	*BMJ Open*
Manuscript ID	bmjopen-2020-040735.R2
Article Type:	Original research
Date Submitted by the Author:	05-Sep-2020
Complete List of Authors:	Chis Ster, Irina; St George's Hospital Medical School, Institute of Infection and Immunity<br>Rodriguez, Alejandro ; International University of Ecuador<br>Romero, Natalia; International University of Ecuador<br>Lopez, Andrea; International University of Ecuador<br>Chico, Martha; International University of Ecuador<br>Montgomery, Joel ; CDC<br>Cooper, Philip; St George's Hospital Medical School, Institute of Infection and Immunity; International University of Ecuador
**Primary Subject Heading**:	Epidemiology
Secondary Subject Heading:	Epidemiology, Infectious diseases
Keywords:	Epidemiology < TROPICAL MEDICINE, STATISTICS & RESEARCH METHODS, PUBLIC HEALTH

SCHOLARONE™
Manuscripts

I, the Submitting Author has the right to grant and does grant on behalf of all authors of the Work (as defined in the below author licence), an exclusive licence and/or a non-exclusive licence for contributions from authors who are: i) UK Crown employees; ii) where BMJ has agreed a CC-BY licence shall apply, and/or iii) in accordance with the terms applicable for US Federal Government officers or employees acting as part of their official duties; on a worldwide, perpetual, irrevocable, royalty-free basis to BMJ Publishing Group Ltd ("BMJ") its licensees and where the relevant Journal is co-owned by BMJ to the co-owners of the Journal, to publish the Work in this journal and any other BMJ products and to exploit all rights, as set out in our licence.

The Submitting Author accepts and understands that any supply made under these terms is made by BMJ to the Submitting Author unless you are acting as an employee on behalf of your employer or a postgraduate student of an affiliated institution which is paying any applicable article publishing charge ("APC") for Open Access articles. Where the Submitting Author wishes to make the Work available on an Open Access basis (and intends to pay the relevant APC), the terms of reuse of such Open Access shall be governed by a Creative Commons licence – details of these licences and which Creative Commons licence will apply to this Work are set out in our licence referred to above.

Other than as permitted in any relevant BMJ Author's Self Archiving Policies, I confirm this Work has not been accepted for publication elsewhere, is not being considered for publication elsewhere and does not duplicate material already published. I confirm all authors consent to publication of this Work and authorise the granting of this licence.

For peer review only

Age-dependent seroprevalence of dengue and chikungunya: inference from a cross-sectional analysis in Esmeraldas Province in coastal Ecuador

Irina Chis Ster[1], Alejandro Rodriguez[2], Natalia Romero[2,5], Andrea Lopez[2], Martha E Chico[3], Joel Montgomery[4], Philip J Cooper[1-3].

[1]Institute of Infection and Immunity, *St George's University of London, London SW17 ORE*, United Kingdom; [2]School of Medicine, *Universidad Internacional del Ecuador, Quito, Ecuador*; [3]*Fundacion Ecuatoriana Para Investigacion en Salud, Quito, Ecuador*; [4]*Centers for Disease Control and Prevention*, Atlanta, Georgia, USA; [5]*Grups* de *Recerca d'America* i *Africa Llatines*.

Conflict of Interests: NONE declared.

Correspondence: Dr Irina Chis Ster, (ichisste@sgul.ac.uk)

Dr Irina Chis Ster

Senior Lecturer in Biostatistics

Institute of Infection and Immunity, St George's University of London

For peer review only - http://bmjopen.bmj.com/site/about/guidelines.xhtml

Dengue and Chikungunya in Ecuador

Cranmer Terrace | Tooting | London | SW17 0RE

Tel: +44 (0)208 725 4937

Email: i.chisster@sgul.ac.uk

Contributors:

Contributors: PJC and ICS designed the study. PJC and MEC supervised data and sample collection. AL did the laboratory assays. ICS developed and implemented the statistical methodology and conducted the literature research. AR provided data and contributed to spatial data analyses. ICS and PJC drafted the manuscript. NR and JM reviewed and provided critical comments on the manuscript. All authors reviewed and approved the final version of the manuscript. ICS is the paper guarantor.

For peer review only

Abstract

Objectives: There are few population-based estimates for prevalence of past exposure to dengue and chikungunya viruses despite common epidemiological features. Here, we have developed a novel statistical method to study patterns of age-dependent prevalence of immunity in a population following exposures to two viruses which share similar epidemiological features including mode of transmission and induce long-lasting immunity. This statistical technique accounted for socio-demographic characteristics associated with individuals and households.

Settings: The data consist of a representative sample from an ongoing longitudinal birth cohort set up in a tropical District in coastal Ecuador (Esmeraldas).

Participants: We collected data and blood samples from 319 individuals belonging to 152 households following epidemics of the infections in 2015 in Latin America.

Primary outcome: Plasma was tested for the presence of specific IgG antibodies to dengue and chikungunya viruses by commercial enzyme-linked immunosorbent assays and defined a bivariate binary outcome indicating individuals' past exposure status to dengue and chikungunya (i.e. presence/absence of IgG antibodies to dengue or chikungunya or both).

Results: Dengue seroprevalence increased rapidly with age reaching 97% (95%CrI 93%-99%) by 60 years. Chikungunya seroprevalence peaked at 42% (95%CrI 18%-66%) around 9 years of age and averaged 27% (95%CrI 8.7%-51.6%) for all ages. Rural areas were more likely to be associated with dengue only exposure while urban areas and shorter distance to the nearest household were associated with exposures to both. Females living in urban settings were more likely to be chikungunya seropositive while rural males were more likely to be dengue seropositive.

For peer review only - http://bmjopen.bmj.com/site/about/guidelines.xhtml

Conclusion: Dengue seroprevalence was strongly age-dependent consistent with endemic exposure while that of chikungunya peaked in childhood consistent with the recent emergence of the virus in the study area. Our findings will inform control strategies for the two arboviruses in Ecuador including recommendations by WHO on dengue vaccination.

Strengths and limitations of this study

- This is the first serological study presenting population-based estimates on age-dependent prevalence of dengue and chikungunya in a region of Ecuador.
- The paper describes a novel statistical method and associated OpenBUGS code.
- The estimates account for individual and household characteristics
- The paper responds to recent WHO call for understanding local epidemiology of dengue prior to designing efficient control strategies.
- More research is needed to assess the impact of various control strategies in the community using these estimates and their uncertainties.

Ethics

Informed written consent was obtained from participants aged 18 or more, and from a parent or legal representative for a minor. Minor assent was obtained from children aged 8 years or older. The study protocol was approved by the Ethics Committee of the Universidad Internacional del Ecuador, Quito, Ecuador.

Acknowledgements

We thank to all participants and their families who agreed to participate in the Ecuavida cohort.

For peer review only - http://bmjopen.bmj.com/site/about/guidelines.xhtml

For peer review only

For peer review only - http://bmjopen.bmj.com/site/about/guidelines.xhtml

Introduction

Dengue virus (DENV) and chikungunya virus (CHIKV), arboviruses belonging to *Flaviviridae* and *Togaviridae* families, respectively, are endemic in many tropical and sub-tropical regions where *Aedes* vectors thrive. Although the two viruses belong to different arbovirus families *[1]*, human infections are associated with similar clinical manifestations and are a major cause of morbidity *[2-4]*. Over the past 15 years, the number of dengue cases reported in Latin America has increased dramatically *[5]* while chikungunya, which emerged in the Caribbean region in 2013, has spread rapidly through the region *[6]* causing an estimated 2.9 million cases *[7-9]*.

Global estimates of the burden of dengue and chikungunya are based on aggregate numbers of reported cases and likely to be biased by underreporting because of limited surveillance, high rates of asymptomatic infections, and non-specific clinical presentation *[10]*. Asymptomatic dengue, for example, can inflate the total number of cases by up to 40-fold *[11-13]*.

Cross-sectional serological surveys can provide valuable information on arbovirus infection burdens and allow more accurate determination of numbers exposed. Serological surveys, thus, have a number of advantages over clinically classified, self-reported cases or other non-objective clinical diagnoses by providing information that are less sensitive to recall, underreporting, and misdiagnosis due to asymptomatic manifestations of related infections *[6, 12-14]*.

Ecuador is a middle-income country in Latin America where dengue has been endemic since the late 1980s *[15]*: dengue is thought to have re-emerged around 1988 following re-colonisation with *A. aegypti* after a thirty-year absence *[16, 17]*. Chikungunya first appeared

For peer review only - http://bmjopen.bmj.com/site/about/guidelines.xhtml

in the Caribbean region in 2013, reaching Ecuador in 2014 causing a large outbreak the following year *[17] [18]*.

Dengue transmission dynamics has been estimated from aggregated data in a series of studies *[19, 20]* with the latter suggesting that the picture provided by serology is very incomplete and limited when trying to characterize transmission across time and space both within and between countries and calling for local data analyses.

Most seroprevalence studies for DENV and CHIKV have examined exposures to each in isolation despite evidence *[21]* of co-circulation and shared risk factors *[6, 22, 23]*. There are no published serological surveys of dengue and chikungunya in Ecuador or studies of co-exposures to the two viruses from the Latin American region *[6]*, and limited information on the determinants of exposure risk. Such data are critical to estimate the burden of infection and inform public health strategies for control. The first licenced dengue vaccine (CYD-TDV) is now available in the Latin American region. Current WHO guidelines advise the implementation of cost-effective prevention strategies that consider local arbovirus epidemiology *[24]*. Recent studies indicated that targeting only seropositive individuals for vaccination might reduce global annual disease incidence by 20 to 30%, with the greatest impact in high-transmission settings *[25]*. Hence, understanding past exposure or seropositivity patterns in affected populations are key to implementing cost-effective control strategies.

In this study, we hypothesised that prevalence of past exposure to DENV and CHIKV in the population are associated with age, and individual and household risk factors. We estimated age-dependent seroprevalence of DENV, CHIKV and co-exposures with both arboviruses in a district in coastal Ecuador, and explored individual and household determinants of past exposure status.

For peer review only - http://bmjopen.bmj.com/site/about/guidelines.xhtml

Materials and methods

Population and study sample

The data consist of a representative sample of households including children (and other household members) recruited into the ECUAVIDA birth cohort between 2005 and 2009 in the town of Quininde, Esmeraldas Province [26]. The District is largely rural in a tropical region of coastal Ecuador with a population of 88,000 living below 150 m altitude with mean annual temperature of 30ºC and relative humidity of 80%. Main sources of income are cultivation of palm oil and tropical fruits, and timber extraction. Children (and all other members of the household to which they belonged) due to attend the cohort clinic in Quininde for routine follow up visits between October 2015 and February 2016 were invited to participate in this study according to the dates of their scheduled attendance. This approach resulted in the selection of a representative sample of ECUAVIDA cohort households (*Supplementary Table 1*). As is frequent for such ad-hoc surveys, post-stratification weightings [27] using the census population distributions were used to adjust the estimates to population-generalizable values (details in supplementary information and weights shown in *Supplementary Table 2.*

Venous blood was collected into heparinized tubes (Vaccutainer, BD Systems), centrifuged and plasma was stored at -20°C. Data on individual and household factors were collected from the child's mother by an investigator-administered questionnaire and the 2010 census population data was used to derive post-stratification weights such that the resulting estimates would represent those of the population *[27]*. Each household was visited and location determined by GPS (Garmin eTrex, KS, USA).

For peer review only - http://bmjopen.bmj.com/site/about/guidelines.xhtml

Laboratory testing

Plasma was analysed for specific IgG antibodies to DENV and CHIKV viruses using commercial ELISA kits (Human anti-Dengue IgG and human anti-Chikungunya IgG ELISA kits, Abcam, Cambridge UK) following manufacturer's instructions. Past exposure or seropositivity to DENV and CHIKV was defined by the presence of specific IgG antibodies.

Statistical methods

The presence or absence of DENV and CHIKV antibodies define a bivariate binary outcome indicating past exposure to DENV only denoted by D+C-, to CHICK only denoted by D-C+, to both, D+C+, or to none. The key assumption of the model is that of long-lasting immunity *[19]* after infection with DENV and CHIKV.

We used two simultaneous multilevel logistic regression equations with shared random effects to model the presence/absence of antibodies for DENV or CHIKV or both. The basic idea of this model was developed initially in econometrics *[28]* and more recently in the context of two binary correlated outcomes indicating infections but using a distinct approach from the one used here *[29]*. Each equation modelled the presence of one arbovirus conditioned on the other, making the presence of DENV more or less likely in the presence of CHIKV (and vice versa). We explored the effects of age, sex, area of residence (urban vs. rural), socioeconomic level (grouped as low, medium, and high derived from tertiles of the first component of principal components analysis of 7 socio-economic variables as described in *[30]*), crowding (number of persons per sleeping room) and household dispersal (Euclidian distance to nearest household using GPS coordinates) on our bivariate binary outcome (dependent variable) defined above. The simplest model started with investigating the effect of age on the outcome which provided answers to the main question of interest, i.e. how the probabilities of past exposures to (D+C-, D-C+ and D+C+) varied with age in this population. The systematic trends with age in these probabilities are called *age-dependent prevalence of past exposure* to D+C-, D-C+ and D+C+.

For peer review only - http://bmjopen.bmj.com/site/about/guidelines.xhtml

They are also predicted probabilities of past exposures. We then refined these findings by exploring the extent to which these overall associations between past exposure to D+C-, D-C+ and D+C+ and age are altered by the other variables – alone and in a multivariable model.

The model (*Supplementary file*) also embedded post-stratification weights, allowing estimates to be generalizable to the population from which the sample was derived *[27]*. Hierarchical structure *[31]* of the data (individuals within families within wards) was represented by shared random effects, accounting for unmeasured epidemiological factors such as biting rates or climate conditions. Because of the assumption of long-life immunity, the age-dependent prevalence of past specific or co-exposures *[14]* can be also regarded as measures of immunity to these viruses in the population.

Bayesian inference was used for parameter estimation and to predict age-dependent probabilities of seropositivity and 95% credible intervals (CrI). In the absence of any formal goodness-of-fit measure, predicted age dependent prevalence was plotted against the observed proportions of positives within the same 5-year age group. Moreover, we also plotted the observed numbers of seropositive individuals across 5-year groupings and across geographical regions against expected numbers predicted by the model.

Detailed information on the statistical approach used with mathematical models, framework for statistical inference, estimated parameters and goodness of fit are provided as Supplementary files. Aggregated data at national and provincial levels for case reports of dengue/chikungunya from 2015 were retrieved from various sources *[16, 17, 32, 33]*. Given the assumption of life-long immunity, age-dependent seroprevalence and risk or probability of past exposures to DENV and CHICKV, are used interchangeably.

For peer review only - http://bmjopen.bmj.com/site/about/guidelines.xhtml

Results

Figure 1 shows the geographical locations of households of children in the background cohort and the locations of those sampled for this analysis (151). There was an almost 3-fold increase in the number of reported DENV cases (42,499 cases) in 2015 compared to 2014 (15,584) and 2016 (13,612). An outbreak of CHIKV of comparable size (34,101 cases) emerged in 2015 after no previously documented history of infection in the country before 2014: Esmeraldas province accounted for a third (10,477) of CHIKV cases in 2015 reported nationally (*Supplementary Table 3* and *Supplementary Figure 1* [showing monthly time-series of cases]). Subsequent (2016) Zika epidemic cumulative numbers are also presented for reference.

Plasma from blood samples collected between September and December 2015 from 319 individuals (including children) belonging to 151 households from 49 wards (electoral sub-divisions at parish level) were analysed. Characteristics for subjects and their households are provided in Table 1. Of 319 individuals, 245 (76.8%) were seropositive for DENV and 145 (45%) were seropositive for CHIKV. High numbers/proportions were positive for both arboviruses: 129 (40%) individuals belonging to 86 (57%) families living in 24 (49%) wards. Proportions of households and wards with at least one seropositive individual were: 143 (95%) households in 46 (94%) wards for DENV and 93 (62%) households in 27 (55%) wards for CHIKV.

The sub-sample of the cohort used in this analysis appeared to represent the overall cohort well in terms of socioeconomic status and area of residence *(Supplementary Table 1*). Distributions by parish and age for the census population of Esmeraldas Province, cohort, and serological sample are shown in *Supplementary Table 2* and *Supplementary Table 3*, respectively, along with stratum-specific weights subsequently used in modelling for population-level

For peer review only - http://bmjopen.bmj.com/site/about/guidelines.xhtml

representativeness. Parameter estimates (conditional odds ratios) which measure the univariate and adjusted associations of the risk of testing positive for DENV, CHIKV and both with each potential risk factor (age, sex, area of residence, socio-economic status and distance to next door neighbour) are shown in Table 2. Given the relative complexity of this non-linear modelling, the results are best understood through a predicted average seroprevalence across the population, with reference to trends and patterns by age and household characteristics (*Supplementary Table 4*).

Average overall DENV seroprevalence (including those seropositive for both viruses) was 84% (95% CrI 75%-91%) but increased rapidly with age from 14% (95% CrI 1.5-45%) in infants to 97% (95% CrI 93-99%) in adults aged 60 years (*Figure 2*). The overall average seroprevalence of CHIKV was 27% (95% CrI 8.7%-51.6%), peaked at 42% (95% CrI 18%-66%) in children aged 9 years, and declined to 18% (95% CrI (5%-38%) by 60 years (*Figure 2*).

Age-dependent prevalence curves for D+C-, D-C+, D+C+ and actual proportions from raw data (bars) for 5-year age groupings are shown *Figure 3*. The predicted curves fitted observed data well: raw proportions of positives were generally close to the 95% CrI predicted curves. Average seroprevalence of D+C- was 58% (95% CrI 38-74%), that of D-C+ was 1% (95% CrI 0-3%) and that of D+C+ was 26% (95% CrI 8-50%) (*Supplementary Table 5*). Seroprevalence increased rapidly following a power law (i.e. faster than exponential) with age for D+C-, declined rapidly for D-C+, and increased rapidly to a peak around 9 years (40%, 95% CrI 18%-64%) for D+C+, followed by a steady decline to 18% (95% CrI 6-37%) by 60 years.

Average seroprevalence by groups defined by co-exposures including sex, area of residence, and socioeconomic status are shown in *Supplementary Table 5* and their sole effects on age-specific prevalence in Table 2 and *Supplementary Figure 2.* Females were more likely to be seropositive for CHIKV (D+C+ and D-C+) but less likely to be singly positive to DENV

For peer review only - http://bmjopen.bmj.com/site/about/guidelines.xhtml

(D+C). The difference in D+C- seroprevalence between males and females was greatest in adolescence while that for D+C+ was greatest in childhood (*Supplementary Figure 3*). Individuals resident in rural areas were more likely to be D+C- while D+C+ was more frequent in urban areas; while shorter distance to nearest household (i.e. household dispersal) appeared to be a risk factor for D+C+.

Strength of associations were preserved in the multivariable model but estimates were less precise as more information was extracted, i.e. the CrIs were wider (Final Model in Table 2). Males remained more likely to be D+C- and less likely to be D+C+ (*Supplementary Figure 4*); individuals living in rural areas were more likely to be D+C- while those living in urban areas were more likely to be D+C+ (*Supplementary Figure 5*); and individuals of lower economic status appeared to be more likely to have all outcomes across all ages although differences were greater among those aged less than 20 years (*Supplementary Figure 6*). *Supplementary Figure 7* and *Supplementary Figure 8* show further evidence for a good fit of the model to the numbers in the data rather than to proportions: group-specific defined by age and geographical regions predicted number of seropositive individuals were derived by averaging the age-specific proportions in that group and used to predict approximate average number of exposed individuals. All the parameter estimates MCMC traces for the most complex model (Table 2) and their correlations are presented in *Supplementary Figure 9 Supplementary Figure 9* and *Supplementary Figure 10*, respectively. The estimated variance-covariance matrices corresponding to the random effects associated with both ward and family structures can also be viewed in *Supplementary Figure 11*.

Discussion

Arbovirus infections are a growing public health threat in Latin America where DENV is endemic in tropical and sub-tropical regions, and CHIKV and Zika virus infections have recently emerged *[6]*. Despite evidence for co-circulation within the same geographic regions

For peer review only - http://bmjopen.bmj.com/site/about/guidelines.xhtml

of more than one type of arbovirus, and in many regions all 3 viruses *[3, 9, 34]* that share the same vectors and cause similar symptomatology *[35]*, there are no studies addressing the epidemiology of co-exposures. Nevertheless, the epidemiological and statistical methodologies developed here can be made to accommodate relatively easily more than two concomitant exposures. Although DENV has been endemic in Ecuador since 1980, there are no previous seroprevalence estimates from Ecuador for either arbovirus *[6]*. Recent research in Machala in southern coastal Ecuador has highlighted the need for integrated surveillance [13] and predictions in the same region have been discussed in the context of El Niño Southern Oscillation climatic phenomena [36, 37].In the present study, we estimated that 84% (95%CrI 75%-91%) of individuals aged 0 to 60 years had been exposed to DENV in a tropical region of coastal Ecuador. However, DENV seroprevalence increased rapidly with age reaching 97% (95% CrI 93%-99%) by 60 years, reflecting cumulative exposures over a 30-year period since DENV re-emerged in the region in the late 1980s *[18]*. Placing this estimate within a global context, it concurs with a previous systematic review *[6]* indicating a high average seroprevalence in the Caribbean and Latin American regions (~65%) compared to Asia (46%) and Africa (18%).

Mean chikungunya seroprevalence was 27% (95% CrI 8.7%-51.6%) in this population and is within the range of seroprevalence estimates from seven previous studies from Latin America: Puerto Rico (23.5%), Guadeloupe (48.1%), Martinique (41.9%), Saint Martin (16.9%), Nicaragua (32.8%) and Brazil (20%) *[38]*. A study of two urban populations in Brazil estimated CHIKV seroprevalence at 57.1% and 45.7%, respectively *[38]* while seroprevalence in a rural area of Chapada district (Brazil) was estimated at 20% (95% CrI, 15.4–35%) *[39]*. A seroprevalence of CHIKV of 75% was observed in urban children in Haiti and French Polynesia *[6]*. Our data showed a much flatter seroprevalence pattern by age with a peak around 9 years, likely reflecting a single intense exposure period during 2015.

For peer review only - http://bmjopen.bmj.com/site/about/guidelines.xhtml

In the present analysis, we developed statistical methodology to address analysis of exposures to co-circulating viruses which share strong epidemiological similarities. This method was applied to a bivariate binary outcome defined by the presence/absence of specific IgG antibodies in sera from a population sample weighted to represent the source population, and aimed at understanding how patterns of age-dependent seroprevalence vary by individual and contextual factors. The model allowed prediction of 4 possible outcomes (i.e. D+C-, D-C+, D+C-, and D-C- [used as reference group]). D+C- was the most prevalent group in this population followed by D+C+ and D-C+ (*Figure 3*). The latter group was small and young with imprecise estimates. This statistical methodology show here can be applied relatively easily to more than two co-exposures in future studies.

Males were more likely to have been exposed to DENV while females had greater exposure to CHIKV, effects that were observed across all ages. Differences in exposure by sex could reflect temporal changes in transmission sources related to accelerating urbanisation in the context of women maintaining traditional roles as home keepers (i.e. transmission is largely within and between households, particularly in an urbanizing/urban setting). The observation that CHIKV seroprevalence was inversely associated with distance between households (e.g. a marker of greater population densities and urbanism) suggest that CHIKV exposures are more intense in crowded places, an observation not seen for DENV in this study. These data are consistent with an outbreak in 2012 in Bangladesh that showed a reduced probability of transmission of CHIKV with increasing distance between households *[40]*. An increased risk of infection among females from the same study was attributed to greater time spent around the house *[40]*. The primary vector, *Ae. aegypti*, is well adapted to urban living and breeds wherever clean water is present, particularly in open recipients around households.

The non-linear age-dependence of DENV seroprevalence is not unexpected given the period over which exposures are likely to have occurred. Our data indicate that by 60 years of age

For peer review only - http://bmjopen.bmj.com/site/about/guidelines.xhtml

almost everyone has been infected at least once with a dengue serotype. In contrast, exposure to CHIKV, which first appeared in Ecuador in 2014, possibly after a long absence *[7]*, was shorter: CHIKV caused a large outbreak in 2015 in coastal Ecuador, particularly in Esmeraldas Province. Thus, the age-seroprevalence profile for chikungunya, unlike dengue for which age can be viewed as a measure of length of exposure, may reflect age-dependent differences in exposures to *Aedes* vectors or susceptibility to infection.

Current global estimates of DENV and CHIKV prevalence are uncertain, being largely based on case reports to public health authorities often by other diagnostic mechanisms than laboratory analyses. Our data support the usefulness of serological surveys to inform evaluations of burden of infection within countries, particularly those with limited health resources. Further, local serological surveys will be useful for planning novel population-level interventions against arbovirus transmission such as vaccination. Dengvaxia is only currently registered dengue vaccine that might be used in endemic populations *[24]* and it is not licensed for use in Ecuador. There has been extensive debate over dengue vaccination planning in the endemic countries: either vaccination of populations with dengue seroprevalence rates above 80% or screening for dengue-specific antibodies and vaccinating only seropositive individuals. The WHO scientific advisory working group concluded that both “mass vaccination based on population seroprevalence criteria” and “pre-vaccination screening” are difficult to implement and neither can achieve herd immunity but favours a pre-vaccination strategy in which only seropositive persons are vaccinated. A better understanding of the local epidemiology of dengue will be essential for the evaluation of cost-effectiveness of such measures *[24]*. Recent research suggested that highlighting areas of high prevalence is important as recent analyses suggested that transmission-blocking interventions such as Wolbachia, even at intermediate efficacy (50% transmission reduction), might reduce global annual disease incidence by up to 90%. The newly licensed Sanofi-

For peer review only - http://bmjopen.bmj.com/site/about/guidelines.xhtml

Pasteur vaccine, targeting only seropositive recipients, (namely those at risk of secondary infection which can result in severe dengue and subsequent death), might reduce global annual disease incidence by 20 to 30%, with the greatest impact in high-transmission settings [25].

Limitations of seroprevalence estimates from the literature are small sample size and poorly representative samples *[6]*. A recent review of seroprevalence studies for DENV, CHIKV, and Zika identified 185 studies of which the majority were specific populations or age sub-groups (60%), and just over a third (38.9%) were samples from the general population *[6]* although few were representative samples. About half (50.3%) of the 185 studies had sample sizes of less than 500 subjects *[6]*. The sample size in this study likely limited our ability to detect effects of individual and contextual factors. We have minimized the potential bias in using a sample from a birth cohort rather than a general population sample, by assigning post-stratification weights based on the 2010 census *[27]*.

Our modelling strategy was based on assumptions that: 1) mortality caused by either infection is negligible- case fatality rate in the America region for dengue is approximately 2.5% annually while that of chikungunya is less than 0.1% *[41, 42]*; and 2) both arboviruses induce long-lasting immunity *[43]* - while likely to be true for CHIKV, relevance to DENV is less clear given that immunity is strain-specific and infections with 4 different strains are possible *[44]*. Molecular analysis of dengue strains in Ecuador have shown all 4 strains to be circulating since before 2013 *[45]*. Further, our data can shed little light on vector competence which may affect the transmission of one or other arbovirus (or DENV serotype/genotype) if the two are circulating in the same population at the same point in time – there is some evidence for adaptation of CHIKV to *A. aegypti* and for displacement of one arbovirus by another within populations of *A. aegypti [46]*. Seroprevalence estimates here likely reflect cumulative exposures to all four DENV strains. Given relatively low case

For peer review only - http://bmjopen.bmj.com/site/about/guidelines.xhtml

fatality rates of 2.5% for DENV (mostly related to severe dengue) and 0.1% for CHIKV *[43, 47]*, this assumption is reasonable. Although the serological tests used were highly sensitive, sensitivity may vary depending on whether infection is primary or secondary *[48]*. In the context of the present study, there was limited evidence of reduced sensitivity in older subjects (more likely to have secondary infections) among whom seroprevalence to DENV reached almost 100% by 60 years. The serological test used for CHIKV and DENV were highly specific: the study was done before the emergence of Zika virus which first appeared in Ecuador in 2016 and in the study area in May 2016 with which serological tests for DENV share significant serological cross-reactivity [49].

Conclusions

Our data, from a household sample weighted to represent the general population, showed strong age-dependence of DENV seroprevalence but not CHIKV which emerged only in 2015 in the study population in a tropical region of coastal Ecuador. Our study will inform policy makers on relevant control strategies in Ecuador and decisions on cost–effectiveness of vaccination strategies for dengue *[24]*. Highlighting areas of high prevalence is important as recent analyses suggested that transmission-blocking interventions such as *Wolbachia*, even at intermediate efficacy (50% transmission reduction), might reduce global annual disease incidence by up to 90% *[25]*. The newly licensed Sanofi-Pasteur vaccine, targeting only *seropositive* recipients, (namely those at risk of secondary infection which can result in severe dengue and subsequent death), might reduce global annual disease incidence by 20 to 30%, with the greatest impact in high-transmission settings *[25]*.

For peer review only - http://bmjopen.bmj.com/site/about/guidelines.xhtml

MODEL	DENGUE CONDITIONED ON CHIKUNGUNYA						CHIKUNGUNYA CONDITIONED ON DENGUE					
	MEAN	SD	MEDIAN	P025	P975	P(OR<=1)/P(OR>1)	MEAN	SD	MEDIAN	P025	P975	P(OR<=1)/P(OR>1)
1st MODELS												
AGE ((POWER*)	7.671	3.123	7.023	3.616	15.450		0.357	0.097	0.346	0.202	0.579	
2nd MODELS												

Tables

Characteristic	N (%)
**Individual characteristics**	**N=319**
Sex	
Male	117(37%)
Female	202(63%)
Age (years)	
0-5	7(2%)
6-10	140(44%)
11-15	4(1%)
16-20	3(1%)
21-25	12(4%)
26-30	45(14%)
31-35	37(12%)
36-40	41(13%)
41-45	15(5%)
46-50	9(3%)
>50	6(2%)
Dengue seropositive	
No	74 (32.2%)
Yes	245 (76.8%)
Chikungunya seropositive	
No	174 (54.5%)
Yes	145 (45.5%)
**Household characteristics**	**N=151**
Area of residence	
Rural	47 (32%)
Urban	104 (68%)
Socioeconomic status	
Low and medium	106 (70%)
High	45 (30%)
Household dispersal (metres)	
<50	13 (8.6%)
51-250	63(41.7%)
251-500	34(22.5%)
501-1,000	12(8%)
>1,000	29(19%)

Table 1. Characteristics of 319 individuals from 151 households in the region of the District of Quininde, Esmeraldas province.

For peer review only

FEMALE vs. MALE	0.728	0.373	0.650	0.248	1.665	**0.822/0.178**	2.971	1.587	2.614	1.030	7.004	**0.017/0.983**
RURAL vs. URBAN	1.490	1.431	1.099	0.260	5.020	0.441/0.559	0.252	0.337	0.159	0.023	1.040	**0.975/0.025**
CROWDING	1.217	0.744	1.042	0.348	3.118	0.476/0.524	0.916	0.759	0.718	0.179	2.808	0.679/0.321
LOW vs. HIGH	3.302	2.472	2.671	0.828	9.518	**0.052/0.948**	0.916	0.858	0.675	0.142	3.148	0.684/0.316
HOUSEHOLD	0.825	0.314	0.772	0.380	1.585	0.771/0.229	0.711	0.384	0.628	0.225	1.685	**0.826/0.174**
FINAL MODELS												
AGE (POWER)	9.980	4.623	8.955	4.290	21.760		0.263	0.085	0.251	0.132	0.460	
FEMALE vs. MALE	0.651	0.347	0.577	0.210	1.528	**0.87/0.13**	3.804	2.356	3.238	1.152	9.831	**0.01/0.99**
RURAL vs. URBAN							0.218	0.479	0.099	0.008	1.167	**0.98/0.02**
LOW vs. HIGH	3.028	1.992	2.536	0.827	8.085	**0.05/0.95**						
HOUSEHOLD							0.794	0.561	0.657	0.186	2.202	0.76/0.24

Table 2. Parameter estimates derived after fitting simultaneous logistic models with increasing number of explanatory variables. 1st Models have age only as predictor. 2nd Models show effects of each of age, sex, area of residence, socio-economic status, household overcrowding and household dispersal on the Odds Ratio of past exposure vs. no exposure to the viruses. Final models included all variables for which the effect in the simpler models, measured as P(OR>1) (or P(OR<1)), was greater than 0.80 or less than 0.20 (shown in bold). Age-dependence is parameterized according to a power law (i.e. age^{power}) and the corresponding estimate represented the *power* of the age^{power} component.

Supplementary Tables

For peer review only - http://bmjopen.bmj.com/site/about/guidelines.xhtml

For peer review only

Supplementary Table 1. Comparison between serological sample and cohort with respect to area of residence and socioeconomic status of households.

Supplementary Table 2. Geographical distribution of households by parish across region of Esmeraldas (census), cohort and data sample. The data sample distribution is similar to that of the cohort and the post-stratification weights adjusted for a fair representation of the Esmeraldas Province. The weights represent essentially the ratios between the census proportions and data proportions across each region. This calibration also adjusted for urban/rural area of residence.

Supplementary Table 3. Total number of reported cases of dengue and chikungunya in Ecuador and Esmeraldas province in 2015 compared to the 2014 and 2016. Overall proportions of cases were calculated using Ecuador and Esmeraldas population total as denominator, respectively. Ecuador census data for 2010 were used and the totals were adjusted according to the annual predicted population growth.

Supplementary Table 4. Age distributions of Esmeraldas census population and serological sample from cohort. The weights used in modelling were the ratios between Esmeraldas census population distribution proportions and age-group proportions in the serological sample.

Supplementary Table 5 Average predicted seroprevalence (proportions) for each exposure category and their 95%Credible Intervals (CrI) for dengue and chikungunya and seroprevalence difference by sex (female [F] vs. males [M]), area of residence (urban [U] vs. rural [R]), and socioeconomic level (low [L] vs. high [H]). Exposure categories are: DENV+CHIKV- [D+C-]; DENV-CHIKV+ [D-C+]; and DENV+CHIKV+ [D+C+].

Figures

For peer review only - http://bmjopen.bmj.com/site/about/guidelines.xhtml

Figure 1. Geographic distribution of the household locations of the background population (ECUAVIDA) and the serological sample.

Figure 2. Predicted age-dependent seroprevalence for dengue and chikungunya. Dashed lines represent the 95% Credible Intervals and the bars represent the observe proportions of seropositive participants.

Figure 3. Predicted age-dependent prevalence for exposure categories to dengue and chikungunya in the sample population plotted against observed proportions (in 5-year age groupings). Dashed lines represent 95% Credible Intervals.

Supplementary Figure 1. Reported clinical cases of dengue and chikungunya in 2015 in Ecuador nationally and in the Province of Esmeraldas. Time-series graphs were derived from data publicly accessible (17, 18, 19, 20)

Supplementary Figure 2. Predicted age-dependent seroprevalence for exposure groups to dengue and chikungunya for categories of area of residence, sex, and household dispersal in study sample. Exposure categories are: DENV+CHIKV- [D+C-]; DENV-CHIKV+ [D-C+]; and DENV+CHIKV+ [D+C+]. Dashed lines represent the 95% Credible Intervals.

Supplementary Figure 3. Predicted age-dependent differences for exposure categories by sex (A), area of residence (B), and socioeconomic status (C). Exposure categories were

For peer review only - http://bmjopen.bmj.com/site/about/guidelines.xhtml

DENV+CHIKV- [D+C-]; DENV-CHIKV+ [D-C+]; and DENV+CHIKV+ [D+C+]. Dashed lines represent 95% Credible Intervals.

Supplementary Figure 4 Age-dependent seroprevalence for exposure groups to dengue and chikungunya stratified by sex, socioeconomic status, and area of residence. Exposure categories were DENV+CHIKV- [D+C-]; DENV-CHIKV+ [D-C+]; and DENV+CHIKV+ [D+C+]. Dashed lines represent the 95% Credible Intervals of the predictions derived from the multivariable model

Supplementary Figure 5. Age-dependent seroprevalence for exposure groups to dengue and chikungunya stratified by area of residence, sex, and socioeconomic status. Exposure categories were DENV+CHIKV- [D+C-]; DENV-CHIKV+ [D-C+]; and DENV+CHIKV+ [D+C+]. Dashed lines represent the 95% Credible Intervals of the predictions derived from the multivariable model (Table 2).

Supplementary Figure 6. Age-dependent seroprevalence for exposure groups to dengue and chikungunya stratified by socioeconomic status, sex, and area of residence. Exposure categories were DENV+CHIKV- [D+C-]; DENV-CHIKV+ [D-C+]; and DENV+CHIKV+ [D+C+]. Dashed lines represent the 95% Credible Intervals of the predictions derived from the multivariable model (Table 2).

Supplementary Figure 7. Model goodness of fit: the observed seropositive numbers in each 5-year age group vs. the expected numbers calculated using average age-specific MCMC

For peer review only - http://bmjopen.bmj.com/site/about/guidelines.xhtml

For peer review only

predictions and the total number in the corresponding age group: (a) for D+C-, (b) for D-C+ and (c) for D+C+.

Supplementary Figure 8 Model goodness of fit: the observed seropositive numbers in groups defined by geographical regions vs. the corresponding expected numbers calculated using MCMC predicted prevalence in that group and its total numbers: (a) for all DENV and (b) for all CHIKV.

Supplementary Figure 9. Parameter estimates for the most complex models - the MCMC traces for 100000 iterations.

Supplementary Figure 10. Parameter correlations based on the MCMC traces for 100000 iterations.

Supplementary Figure 11 Variance-covariance components (random effects) estimation associated with hierarchical structure of the data (family and ward).

For peer review only

Financial support

The project was supported by the Wellcome Trust (088862/Z/09/Z) and the Universidad Internacional del Ecuador.

Data sharing statement

The data were from a longitudinal birth cohort study and are available upon request to pcooper@sgul.ac.uk

For peer review only - http://bmjopen.bmj.com/site/about/guidelines.xhtml

Patient and Public Involvement statement

Patients were not involved in design or conduct of this study and there are no plans to disseminate the results to study participants.

For peer review only

For peer review only - http://bmjopen.bmj.com/site/about/guidelines.xhtml

References

1. Torres, M.C., et al., *Re-introduction of dengue virus serotype 2 in the state of Rio de Janeiro after almost a decade of epidemiological silence.* PLOS ONE, 2019. **14**(12): p. e0225879.
2. Brathwaite Dick, O., et al., *The History of Dengue Outbreaks in the Americas.* The American Journal of Tropical Medicine and Hygiene, 2012. **87**(4): p. 584-593.
3. Ding, F., et al., *Mapping the spatial distribution of Aedes aegypti and Aedes albopictus.* Acta Tropica, 2018. **178**: p. 155-162.
4. Kraemer, M.U.G., et al., *The global distribution of the arbovirus vectors Aedes aegypti and Ae. albopictus.* eLife, 2015. **4**: p. e08347.
5. Brady, O.J., et al., *Refining the Global Spatial Limits of Dengue Virus Transmission by Evidence-Based Consensus.* PLOS Neglected Tropical Diseases, 2012. **6**(8): p. e1760.
6. Fritzell, C., et al., *Current challenges and implications for dengue, chikungunya and Zika seroprevalence studies worldwide: A scoping review.* PLoS Neglected Tropical Diseases, 2018. **12**(7).
7. Scott, B.H., *Reappearance of Chikungunya, Formerly Called Dengue, in the Americas.* Emerging Infectious Disease journal, 2015. **21**(4): p. 561.
8. Yactayo, S., et al., *Epidemiology of Chikungunya in the Americas.* The Journal of infectious diseases, 2016. **214**(suppl 5): p. S441-S445.
9. Wahid, B., et al., *Global expansion of chikungunya virus: mapping the 64-year history.* International Journal of Infectious Diseases, 2017. **58**: p. 69-76.
10. Romero-Sandoval, N., et al., *Critical analysis of the definition of severe dengue, based on the Ecuador outbreak in 2012*. Vol. 04. 2014. 36-38.
11. Balmaseda, A., et al., *Trends in patterns of dengue transmission over four years of a pediatric cohort study in Nicaragua.* The Journal of infectious diseases, 2010. **201**(1): p. 5-14.
12. Morrison, A.C., et al., *Epidemiology of Dengue Virus in Iquitos, Peru 1999 to 2005: Interepidemic and Epidemic Patterns of Transmission.* PLOS Neglected Tropical Diseases, 2010. **4**(5): p. e670.
13. Vitale, M., et al., *A comparison of passive surveillance and active cluster-based surveillance for dengue fever in southern coastal Ecuador.* BMC Public Health, 2020. **20**(1): p. 1065.
14. Chis Ster, I., *Inference for serological surveys investigating past exposures to infections resulting in long-lasting immunity – an approach using finite mixture models with concomitant information.* Journal of Applied Statistics, 2012. **39**(11): p. 2523-2542.
15. CDC. *International Notes Dengue Epidemic -- Ecuador*. 1988; Available from: https://www.cdc.gov/mmwr/preview/mmwrhtml/00001411.htm.
16. PÚBLICA, S.N.D.V.-M.D.S. *Dengue in Ecuador*. Available from: https://www.salud.gob.ec/wp-content/uploads/2013/02/Gecata-Dengue-SE-15.pdf.
17. PÚBLICA, S.N.D.V.-M.D.S. *Chickungunya in Ecuador*. Available from: https://www.salud.gob.ec/wp-content/uploads/2017/07/GACETA-VECTORES-SE-1.pdf.
18. Stewart-Ibarra, A.M., et al., *The high burden of dengue and chikungunya in southern coastal Ecuador: Epidemiology, clinical presentation, and phylogenetics from a prospective study in Machala in 2014 and 2015.* bioRxiv, 2017.
19. Imai, N., et al., *Estimating Dengue Transmission Intensity from Sero-Prevalence Surveys in Multiple Countries.* PLOS Neglected Tropical Diseases, 2015. **9**(4): p. e0003719.
20. Rodriguez-Barraquer, I., H. Salje, and D.A. Cummings, *Opportunities for improved surveillance and control of dengue from age-specific case data.* eLife, 2019. **8**: p. e45474.
21. Paixão, E.S., M.G. Teixeira, and L.C. Rodrigues, *Zika, chikungunya and dengue: the causes and threats of new and re-emerging arboviral diseases.* BMJ Global Health, 2018. **3**(Suppl 1): p. e000530.
22. Beltrán-Silva, S.L., et al., *Clinical and differential diagnosis: Dengue, chikungunya and Zika.* Revista Médica del Hospital General de México, 2016.

For peer review only - http://bmjopen.bmj.com/site/about/guidelines.xhtml

23. Rodríguez-Barraquer, I., et al., *The Hidden Burden of Dengue and Chikungunya in Chennai, India.* PLoS Negl Trop Dis, 2015. **9**(7): p. e0003906.
24. Wilder-Smith, A., et al., *Deliberations of the Strategic Advisory Group of Experts on Immunization on the use of CYD-TDV dengue vaccine.* The Lancet Infectious Diseases, 2019. **19**(1): p. e31-e38.
25. Cattarino, L., et al., *Mapping global variation in dengue transmission intensity.* Science Translational Medicine, 2020. **12**(528): p. eaax4144.
26. Cooper, P.J., et al., *Cohort Profile: The Ecuador Life (ECUAVIDA) study in Esmeraldas Province, Ecuador.* International Journal of Epidemiology, 2015. **44**(5): p. 1517-1527.
27. Little, R.J.A., *Post-Stratification: A Modeler's Perspective.* Journal of the American Statistical Association, 1993. **88**(423): p. 1001-1012.
28. Schmidt, P. and R.P. Strauss, *Estimation of Models with Jointly Dependent Qualitative Variables: A Simultaneous Logit Approach.* Econometrica, 1975. **43**(4): p. 745-755.
29. Ghebremichael, M., *Joint modeling of correlated binary outcomes: HIV-1 and HSV-2 co-infection.* Journal of Applied Statistics, 2015. **42**(10): p. 2180-2191.
30. Menzies, S.K., et al., *Risk factors for soil-transmitted helminth infections during the first 3 years of life in the tropics; findings from a birth cohort.* PLoS Negl Trop Dis, 2014. **8**(2): p. e2718.
31. Goldstein, H., *Multilevel Statistical Models*. 4th Edition ed. 2011: JohnWiley and Sons, Ltd, Chichester.
32. PÚBLICA, S.N.D.V.-M.D.S. *Dengue in Ecuador - Esmeraldas*. Available from: https://www.salud.gob.ec/wp-content/uploads/2013/02/DENGUE-GACETA-SE28.pdf.
33. PÚBLICA, S.N.D.V.-M.D.S. *Chikungunya in Ecuador - Esmeraldas*. Available from: https://www.salud.gob.ec/wp-content/uploads/2014/09/GACETA-CHIKUNGUNYA-SE-52.pdf.
34. Leta, S., et al., *Global risk mapping for major diseases transmitted by Aedes aegypti and Aedes albopictus.* International journal of infectious diseases : IJID : official publication of the International Society for Infectious Diseases, 2018. **67**: p. 25-35.
35. Mardekian, S.K. and A.L. Roberts, *Diagnostic Options and Challenges for Dengue and Chikungunya Viruses.* BioMed research international, 2015. **2015**: p. 834371-834371.
36. Petrova D, R.X., Sippy R, Ballester J, Mejia R, Beltran-Ayala E, Borbor-Cordova MJ, Vallejo GM, Olmedo AA, Stewart-Ibarra AM, Lowe R. , *he 2018-2019 weak El Niño: predicting the risk of a dengue outbreak in Machala, Ecuador.* International Journal of Climatology, 2020. **In Press**.
37. Petrova, D., et al., *Sensitivity of large dengue epidemics in Ecuador to long-lead predictions of El Niño.* Climate Services, 2019. **15**: p. 100096.
38. Dias, J.P., et al., *Seroprevalence of Chikungunya Virus after Its Emergence in Brazil.* Emerging infectious diseases, 2018. **24**(4): p. 617-624.
39. Cunha, R.V., et al., *Seroprevalence of Chikungunya Virus in a Rural Community in Brazil.* PLOS Neglected Tropical Diseases, 2017. **11**(1): p. e0005319.
40. Salje, H., et al., *How social structures, space, and behaviors shape the spread of infectious diseases using chikungunya as a case study.* Proc Natl Acad Sci U S A, 2016. **113**(47): p. 13420-13425.
41. WHO. *Dengue and severe dengue*. Available from: https://www.who.int/news-room/fact-sheets/detail/dengue-and-severe-dengue.
42. WHO. *Chikungunya*. Available from: https://www.who.int/news-room/fact-sheets/detail/chikungunya.
43. Torres, J.R., et al., *Epidemiological Characteristics of Dengue Disease in Latin America and in the Caribbean: A Systematic Review of the Literature.* Journal of tropical medicine, 2017. **2017**: p. 8045435-8045435.

For peer review only - http://bmjopen.bmj.com/site/about/guidelines.xhtml

44. Andraud, M., et al., *Dynamic Epidemiological Models for Dengue Transmission: A Systematic Review of Structural Approaches.* PLOS ONE, 2012. **7**(11): p. e49085.
45. Ramos-Castañeda, J., et al., *Dengue in Latin America: Systematic Review of Molecular Epidemiological Trends.* PLoS neglected tropical diseases, 2017. **11**(1): p. e0005224-e0005224.
46. Souza-Neto, J.A., J.R. Powell, and M. Bonizzoni, *Aedes aegypti vector competence studies: A review.* Infection, Genetics and Evolution, 2019. **67**: p. 191-209.
47. Moraes, G.H., E. de Fátima Duarte, and E.C. Duarte, *Determinants of mortality from severe dengue in Brazil: a population-based case-control study.* The American journal of tropical medicine and hygiene, 2013. **88**(4): p. 670-676.
48. Simmons, C.P., et al., *Dengue.* New England Journal of Medicine, 2012. **366**(15): p. 1423-1432.
49. Priyamvada, L., et al., *Human antibody responses after dengue virus infection are highly cross-reactive to Zika virus.* Proc Natl Acad Sci U S A, 2016. **113**(28): p. 7852-7.

For peer review only

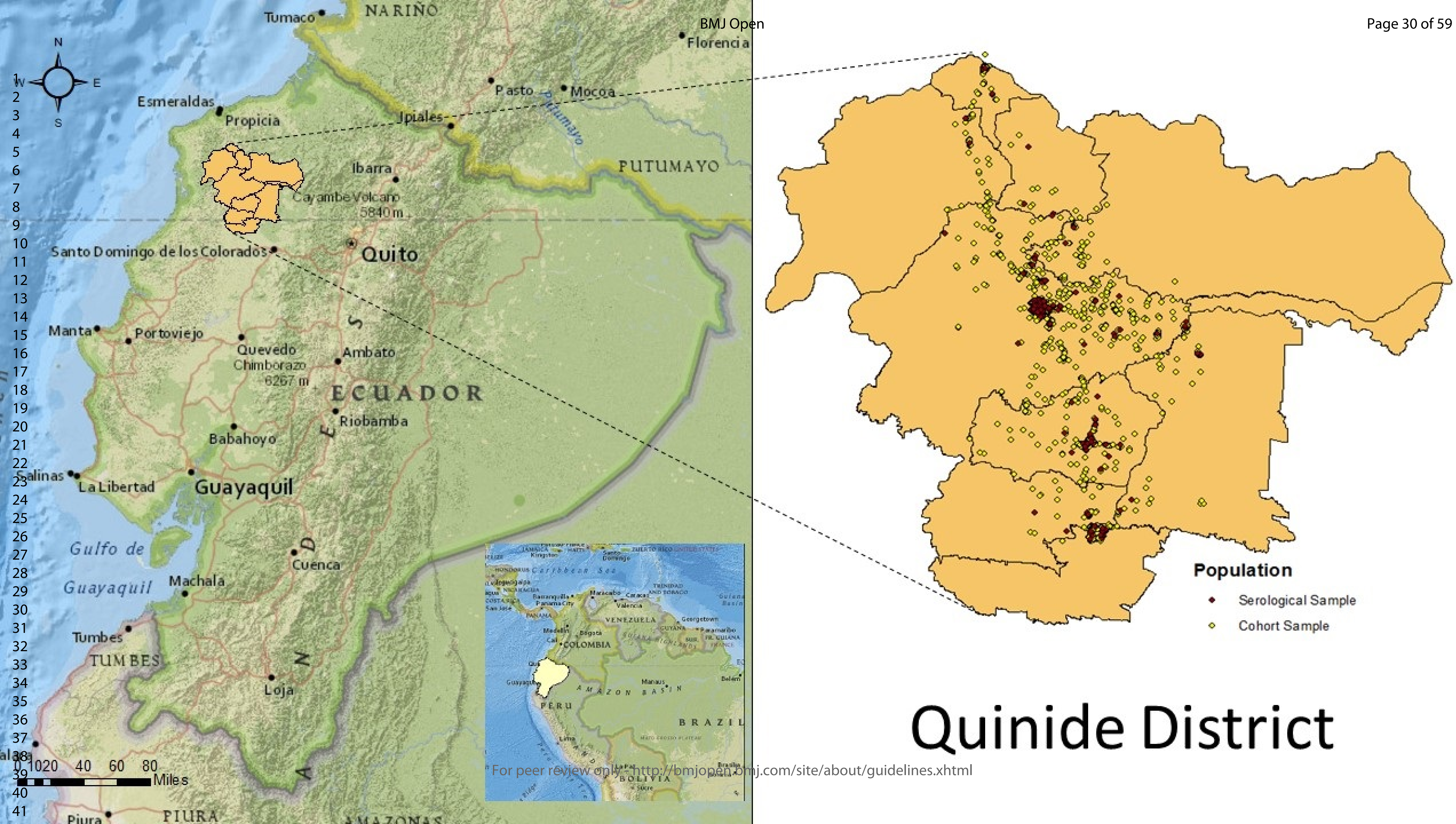
Tumaco
NARIÑO
Florencia
N
W
E
S
Esmeraldas
Propicia
Pasto
Mocoa
Putumayo
Ipiales
PUTUMAYO
Ibarra
Cayambe Volcano
5840 m
Santo Domingo de los Colorados
Quito
Manta
Portoviejo
Quevedo
Chimborazo
6267 m
Ambato
ECUADOR
Riobamba
Babahoyo
Salinas
La Libertad
Guayaquil
Gulfo de
Guayaquil
Machala
Cuenca
Tumbes
TUMBES
Loja
PIURA
Piura
AMAZONAS
0 10 20 40 60 80
Miles
COLOMBIA
VENEZUELA
PERU
BRAZIL
AMAZON BASIN
Population
Serological Sample
Cohort Sample
Quinide District

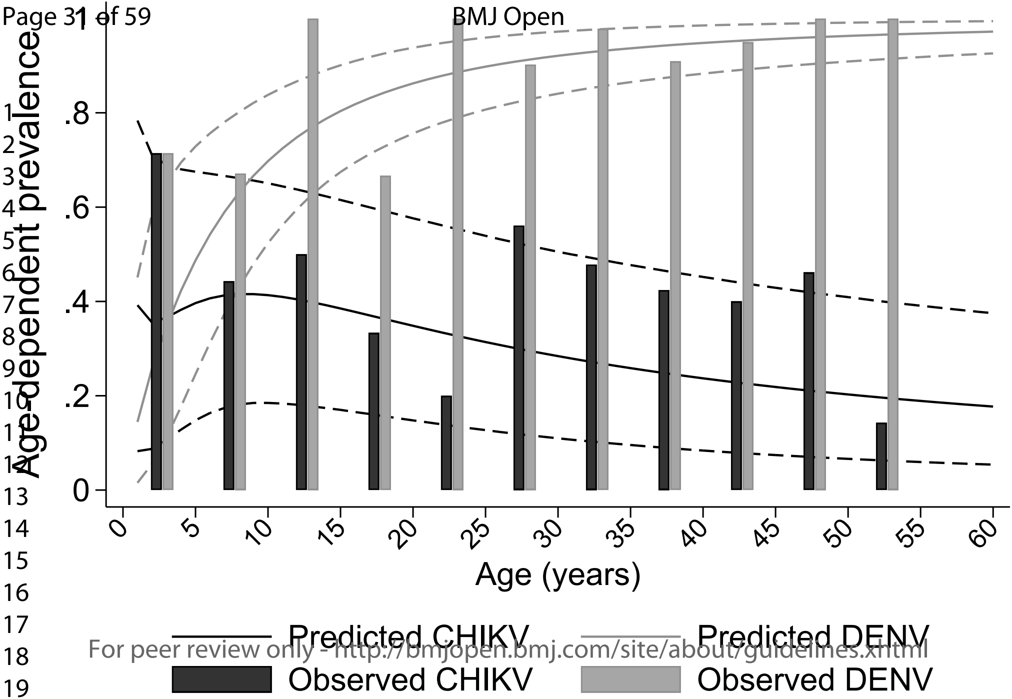

For peer review only - http://bmjopen.bmj.com/site/about/guidelines.xhtml

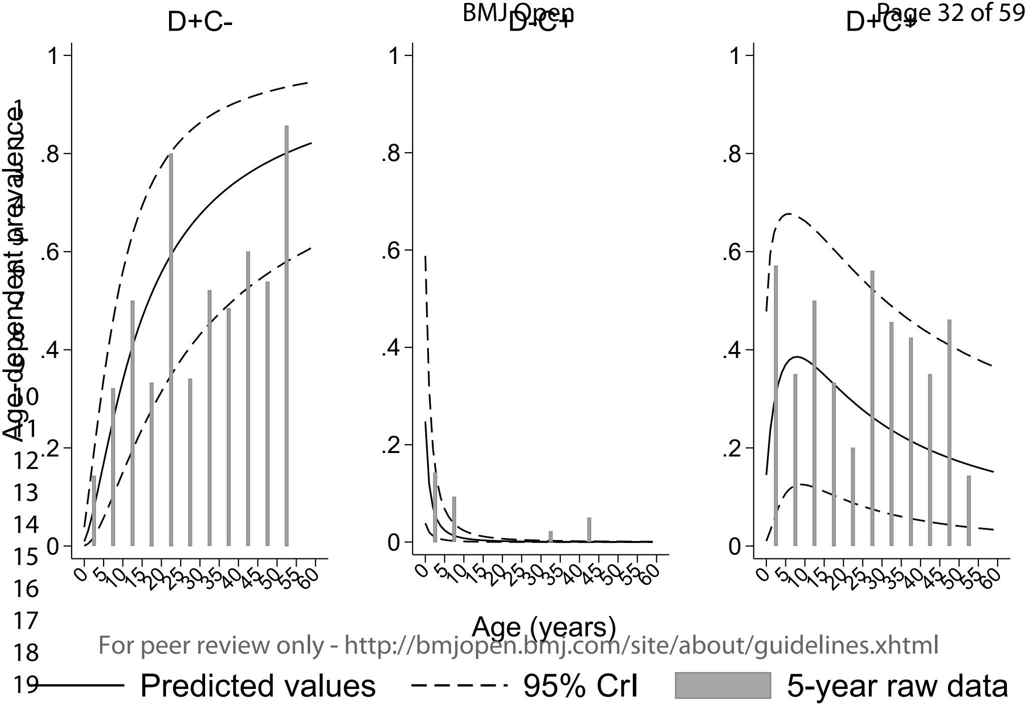

For peer review only - http://bmjopen.bmj.com/site/about/guidelines.xhtml

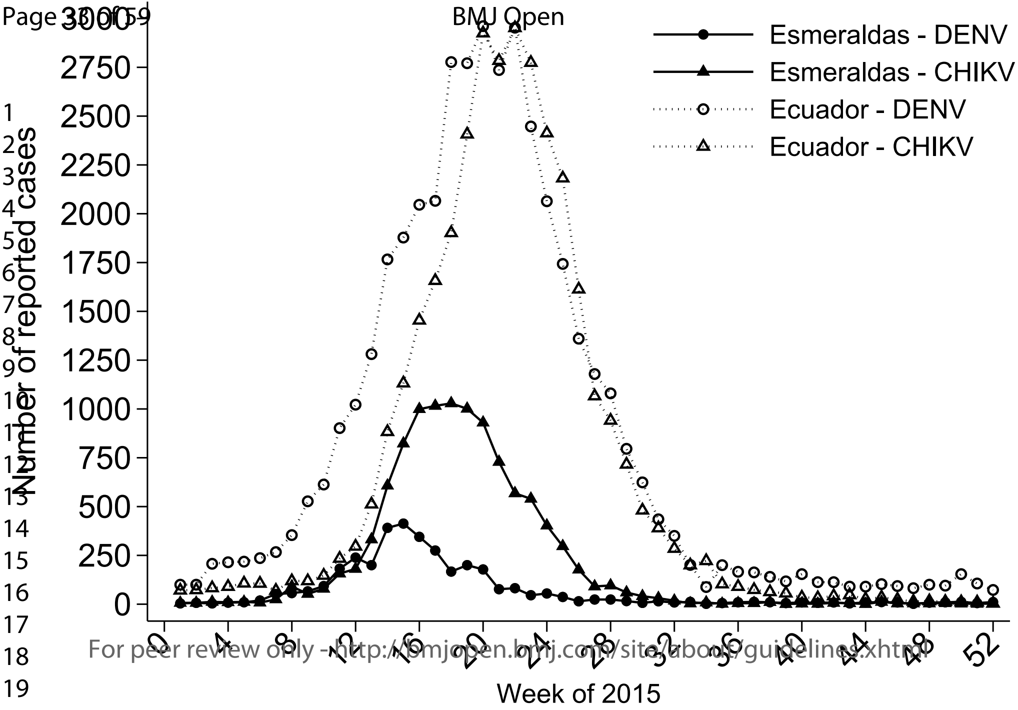

For peer review only - http://bmjopen.bmj.com/site/about/guidelines.xhtml

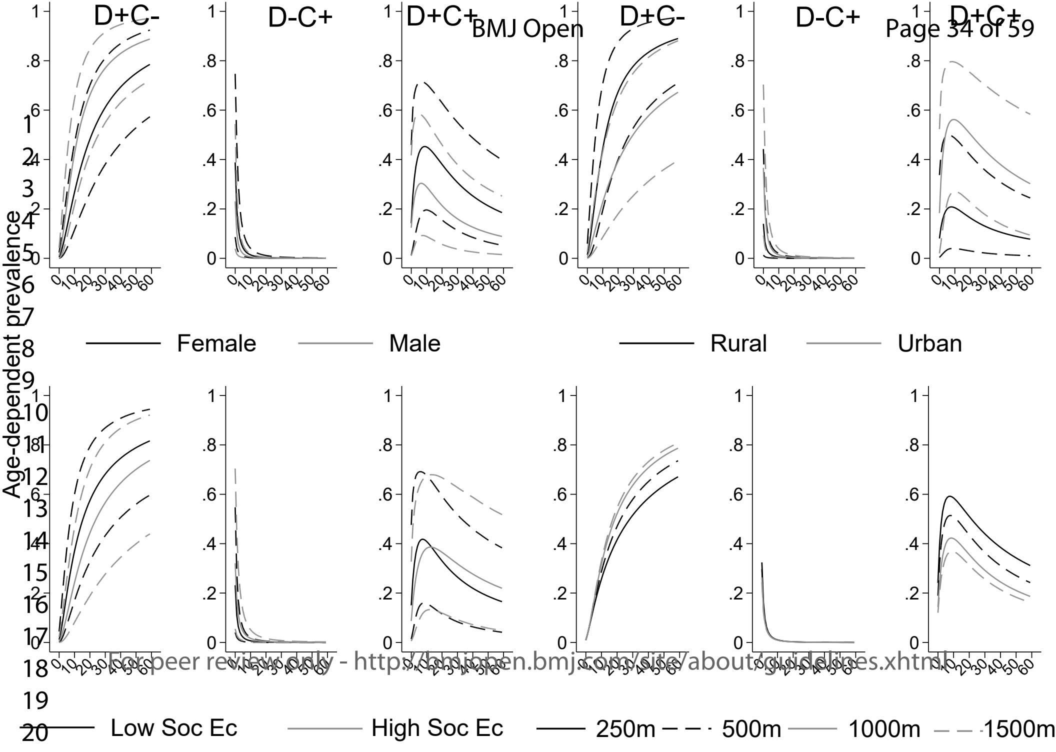

For peer review only - http://bmjopen.bmj.com/site/about/guidelines.xhtml

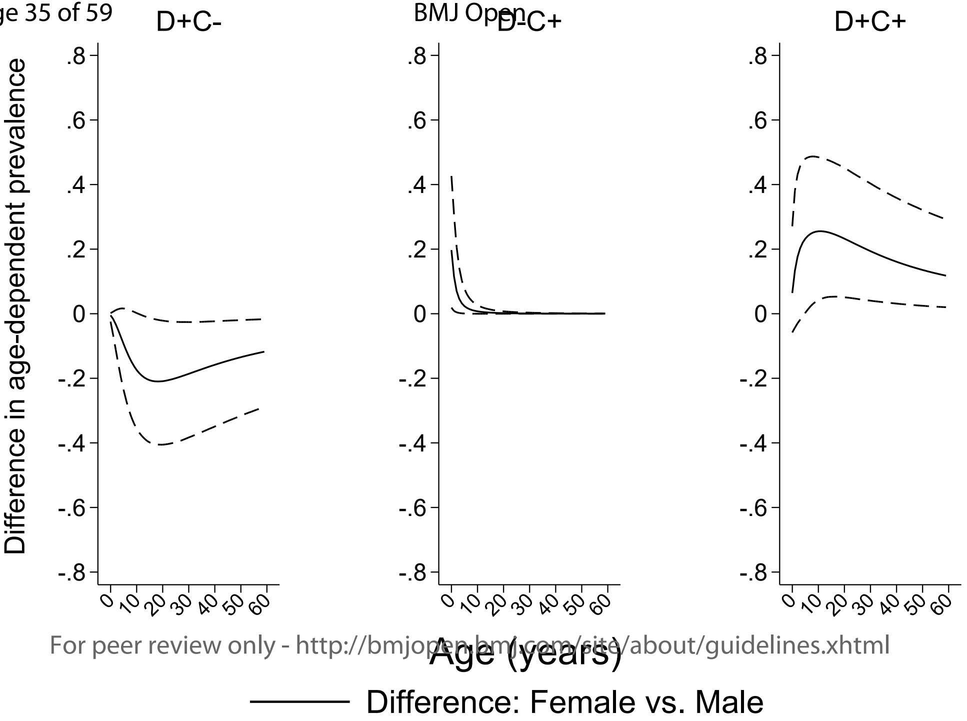

For peer review only - http://bmjopen.bmj.com/site/about/guidelines.xhtml

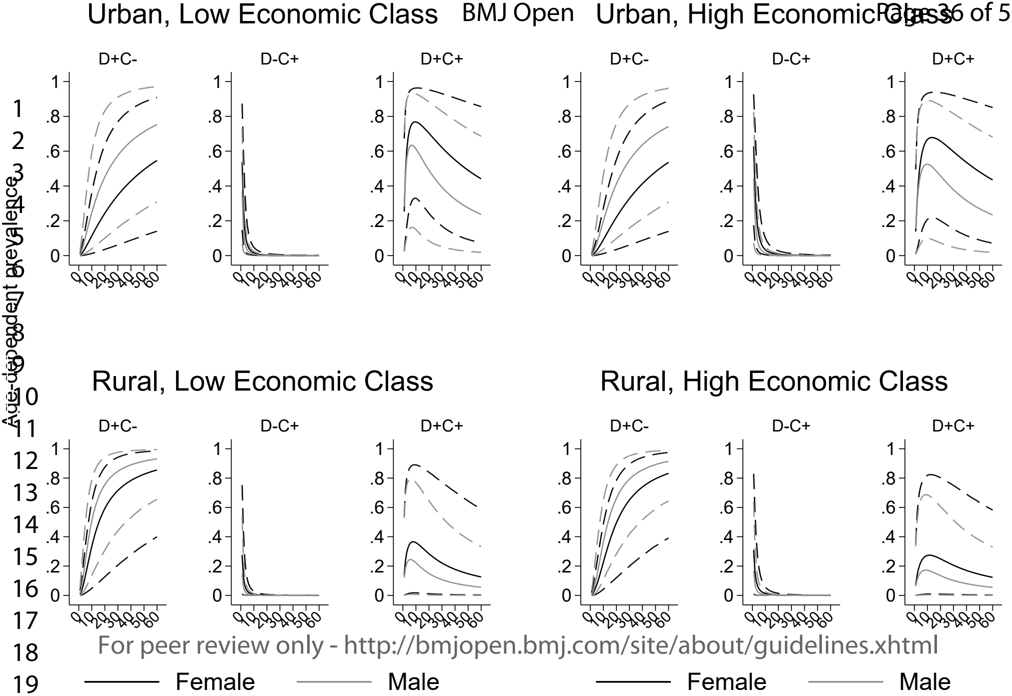

For peer review only - http://bmjopen.bmj.com/site/about/guidelines.xhtml

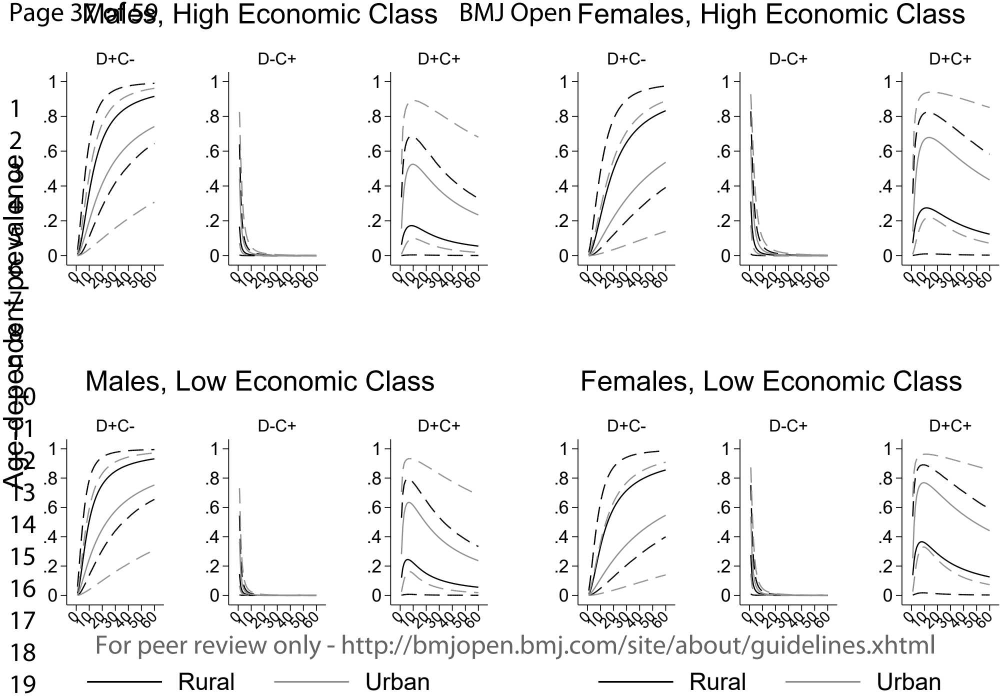

For peer review only - http://bmjopen.bmj.com/site/about/guidelines.xhtml

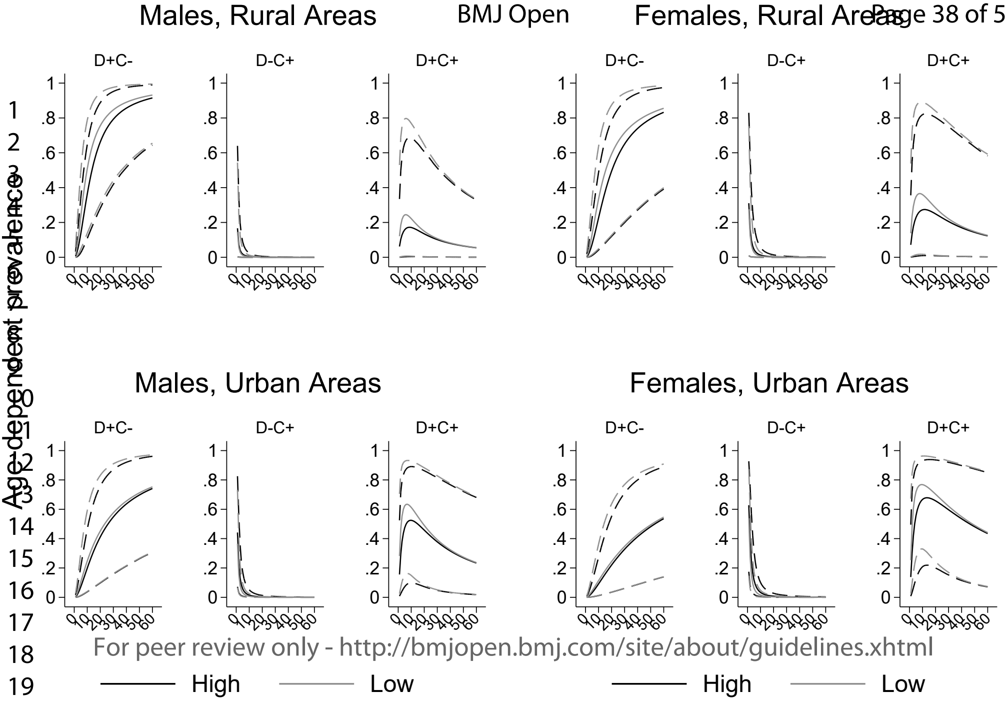
Males, Rural Areas
Females, Rural Areas
Males, Urban Areas
Females, Urban Areas
D+C-
D-C+
D+C+
Age-dependent prevalence
0
10
20
30
40
50
60
0
.2
.4
.6
.8
1
High
Low

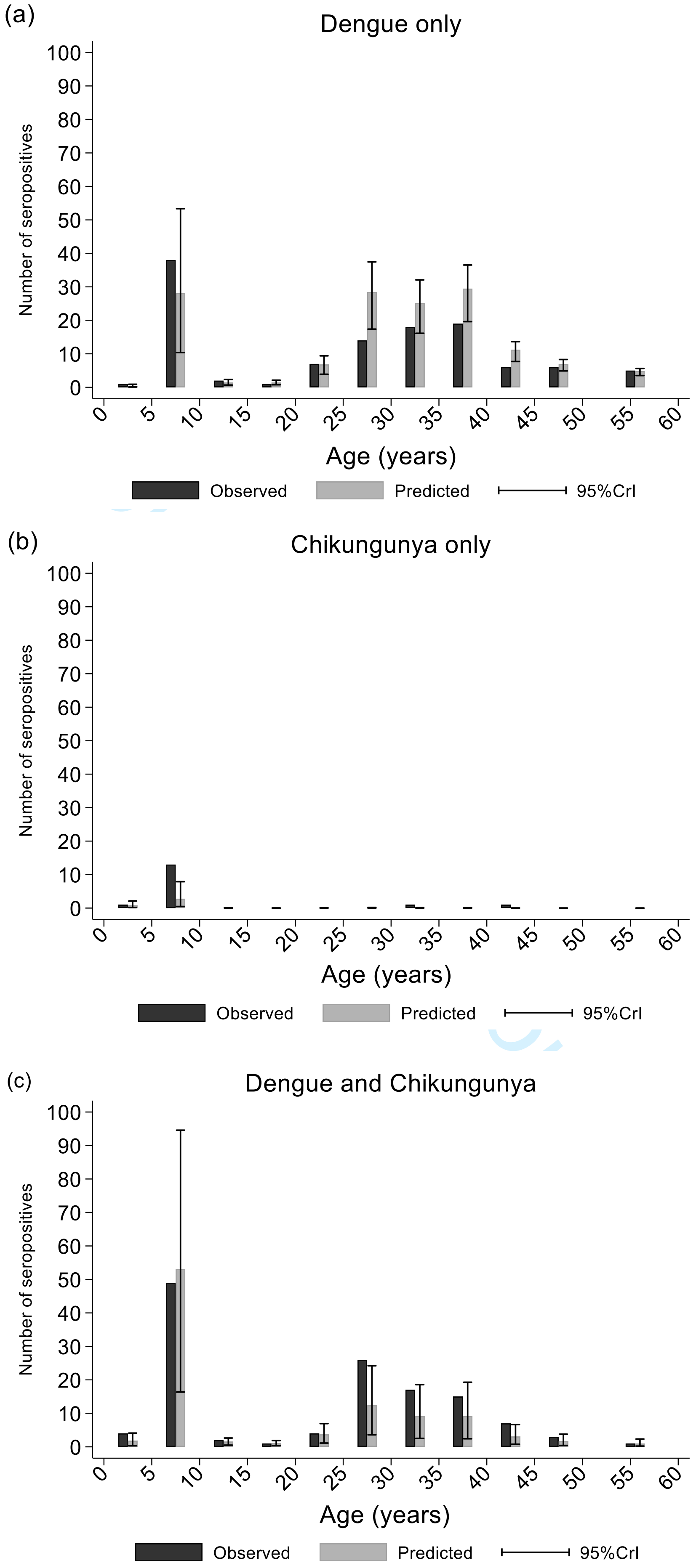
(a)
Dengue only
Number of seropositives
0
10
20
30
40
50
60
70
80
90
100
0 5 10 15 20 25 30 35 40 45 50 55 60
Age (years)
Observed
Predicted
95%CrI
(b)
Chikungunya only
Number of seropositives
Age (years)
Observed
Predicted
95%CrI
(c)
Dengue and Chikungunya
Number of seropositives
Age (years)
Observed
Predicted
95%CrI

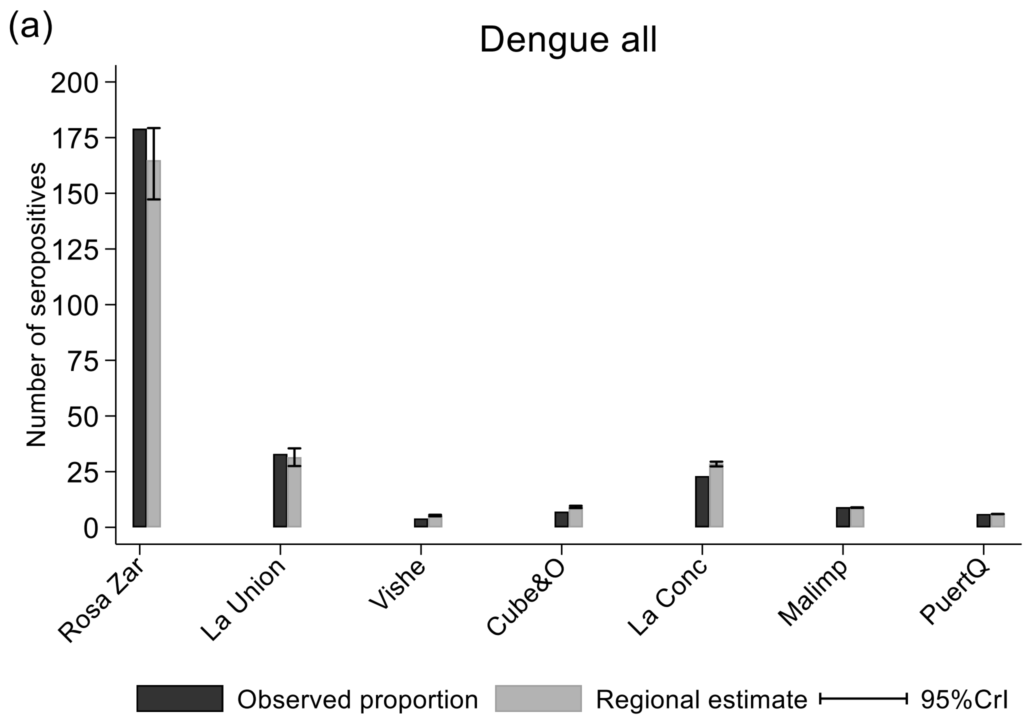
(a)
Dengue all
Number of seropositives
200
175
150
125
100
75
50
25
0
Rosa Zar
La Union
Vishe
Cube&O
La Conc
Malimp
PuertQ
Observed proportion
Regional estimate
95%CrI

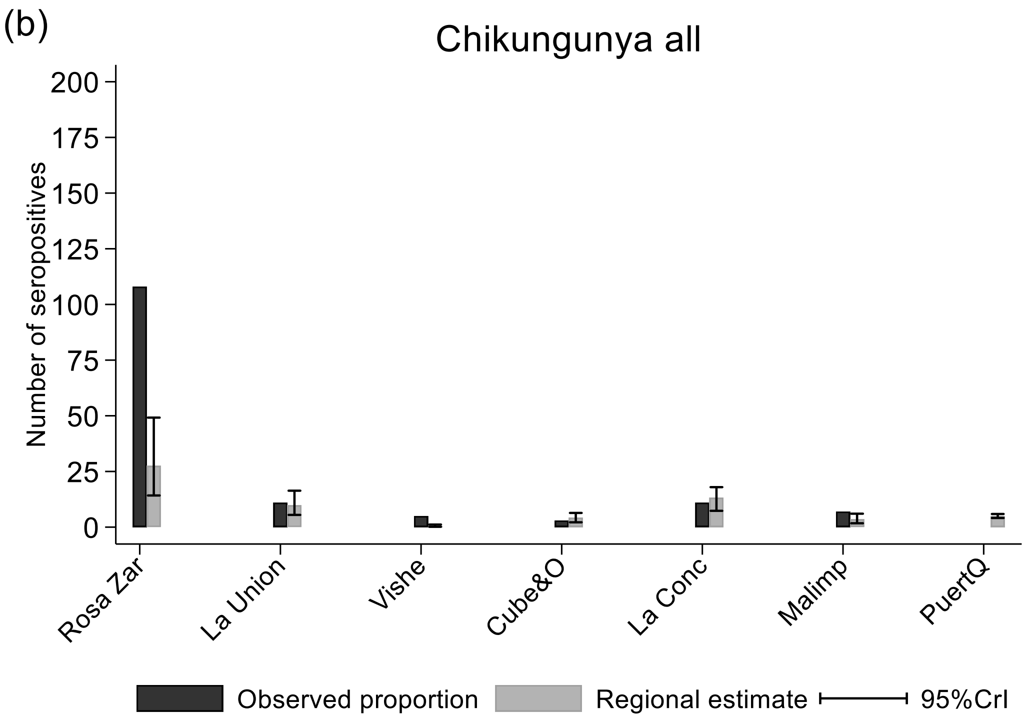
(b)
Chikungunya all
Number of seropositives
200
175
150
125
100
75
50
25
0
Rosa Zar
La Union
Vishe
Cube&O
La Conc
Malimp
PuertQ
Observed proportion
Regional estimate
95%CrI

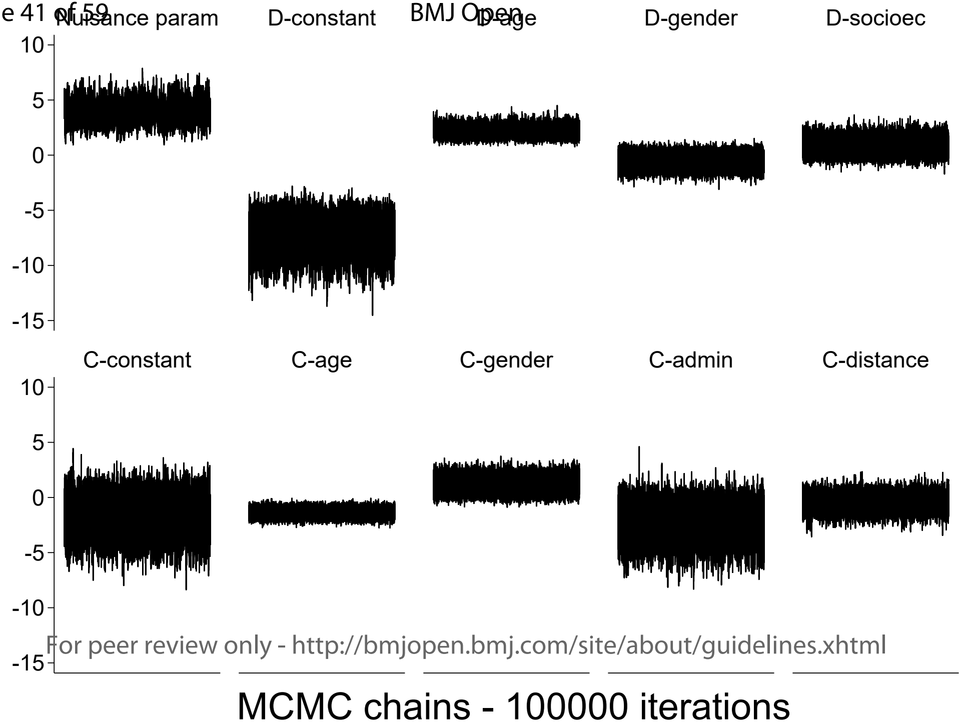

For peer review only - http://bmjopen.bmj.com/site/about/guidelines.xhtml

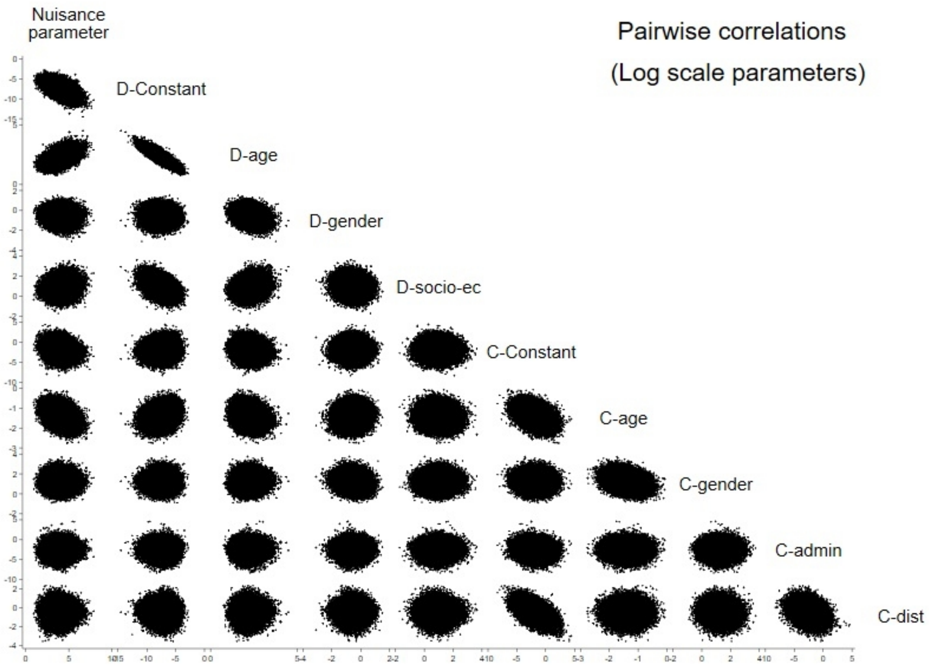

224x162mm (96 x 96 DPI)

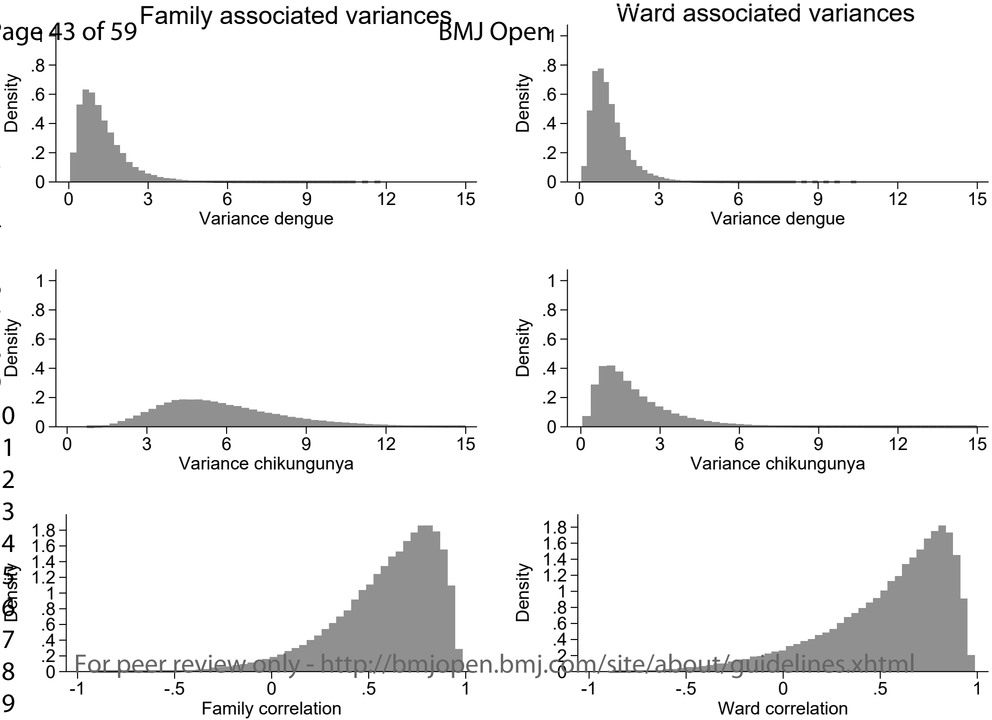

For peer review only - http://bmjopen.bmj.com/site/about/guidelines.xhtml

Variable	Category	Serological cohort 151 households Number/Prop	Ecuavida 2404 households Number/Prop	Serological cohort vs Ecuavida One-sample test
Administrative location	Urban	115 (76.2%)	1,796 (75%)	p=0.74
	Rural	36 (23.8%)	608 (25%)	
Socio Economic Status	High	45 (29.8%)	797 (33.2%)	P=0.46
	Low	106 (70.2%)	1,607(66.8%)	

Supplementary Table 1

For peer review only

	ALL	ECUAVIDA	SAMPLE	Prop of ALL	Prop of ECUAVIDA	Prop of SAMPLE	PROP ECUAVIDA / PROP ALL	PROP SAMPLE / PROP ECUAVIDA	RATIO OF THE LAST	WEIGHTS= 1/RATIO
**Rosa Zarate**	16342	1578	102	0.353	0.656	0.675	1.862	1.029	1.916	**0.522**
**La Concord**	10662	217	13	0.230	0.090	0.086	0.392	0.954	0.374	**2.672**
**PuertoQuito**	5037	27	3	0.109	0.011	0.020	0.103	1.769	0.183	**5.469**
**La Unión**	4963	351	21	0.107	0.146	0.139	1.364	0.953	1.299	**0.770**
**Cube+Others**	4163	49	6	0.090	0.020	0.040	0.227	1.949	0.442	**2.260**
**Malimpia**	4004	81	3	0.086	0.034	0.020	0.390	0.590	0.230	**4.348**
**Viche**	1185	101	3	0.026	0.042	0.020	1.644	0.473	0.777	**1.287**
**Total**	46356	2404	151	1	1	1				

Supplementary Table 2

For peer review only

Year	Population		Dengue		Chickungunya		Zika	
	**Ecuador**	**Esmeraldas**	**Ecuador**	**Esmeraldas**	**Ecuador**	**Esmeraldas**	**Ecuador**	**Esmeraldas**
2014	15,944,275	187,880	15, 584 (0.10%)	984 (0.5%)	39 (~0%)	0 (0%)	0 (0%)	0 (0%)
2015	16,144,368	196,350	42, 499 (0.26%)	3,482 (1.8%)	34,101 (0.2%)	10,477 (5.3%)	1 (0%)	0 (0%)
2016	16,385,068	204,820	13,612 (0.08%)	1,311 (0.6%)	1, 860 (0.01%)	301 (0.15%)	2946(0.02%)	192(0.09%)

Supplementary Table 3

For peer review only

Age Group	Age distribution of census population in Esmeraldas		Age distribution of serological sample		Age-adjusted weights=
	Numbers	Proportions	Numbers	Proportions	
0-9	32452	0.211	147	0.461	**0.458**
10-19	33798	0.219	7	0.022	**9.999**
20-29	26507	0.172	46	0.144	**1.193**
30-39	20092	0.130	79	0.248	**0.527**
40-49	16477	0.107	33	0.103	**1.034**
50-59	12279	0.080	4	0.013	**6.357**
60+	12430	0.081	3	0.009	**8.581**

Supplementary Table 4

For peer review only

OUTCOME	CATEG	MEAN	SD	LOWER 95% CrI	MEDIAN	UPPER 95% CrI
				**ALL**		
		MEAN	SD	P025	MEDIAN	P975
**D+C-**	**ALL**	0.58	0.09	0.38	0.59	0.74
**D-C+**		0.01	0.01	0.00	0.01	0.03
**D+C+**		0.26	0.11	0.08	0.25	0.50
**D+**		0.84	0.04	0.75	0.84	0.91
**C+**		0.27	0.11	0.09	0.27	0.52
				**BY SEX**		
**D+C-**	**MALE**	0.69	0.08	0.51	0.70	0.81
	**FEMALE**	0.53	0.10	0.32	0.54	0.72
**D-C+**	**MALE**	0.008	0.01	0.001	0.01	0.023
	**FEMALE**	0.018	0.01	0.004	0.02	0.04
**D+C+**	**MALE**	0.13	0.09	0.03	0.12	0.33
	**FEMALE**	0.32	0.12	0.11	0.31	0.57
				**SEX DIFFERENCE**		
**D+C-**	**F - M**	-0.15	0.07	-0.31	-0.15	-0.018
**D-C+**		0.01	0.007	0.00	0.009	0.03
**D+C+**		0.18	0.09	0.04	0.18	0.37
				**BY AREA OF RESIDENCE**		
**D+C-**	**URBAN**	0.46	0.12	0.23	0.47	0.68
	**RURAL**	0.68	0.09	0.39	0.70	0.86
**D-C+**	**URBAN**	0.017	0.01	0.004	0.015	0.04
	**RURAL**	0.006	0.005	0.00	0.005	0.02
**D+C+**	**URBAN**	0.39	0.14	0.13	0.38	0.68
	**RURAL**	0.21	0.13	0.03	0.18	0.53
				**AREA OF RESIDENCE DIFFERENCES**		
**D+C-**	**U - R**	-0.21	0.16	-0.52	-0.22	0.13
**D-C+**		0.01	0.01	-0.004	0.01	0.03
**D+C+**		0.18	0.18	-0.21	0.18	0.53
				**BY SOCIOECONOMIC STATUS**		
**D+C-**	**LOW**	0.61	0.11	0.37	0.63	0.79
	**HIGH**	0.51	0.13	0.24	0.52	0.73
**D-C+**	**LOW**	0.009	0.006	0.001	0.007	0.03
	**HIGH**	0.016	0.01	0.002	0.013	0.04
**D+C+**	**LOW**	0.26	0.12	0.07	0.24	0.52
	**HIGH**	0.28	0.15	0.06	0.26	0.61
				**SOCIOECONOMIC STATUS DIFFERENCES**		
**D+C-**	**L-H**	0.10	0.14	-0.18	-0.10	0.39
**D-C+**		-0.006	0.01	-0.07	0.00	0.008
**D+C+**		-0.017	0.16	-0.35	0.02	0.29

Supplementary Table 5

For peer review only

Supplementary: The statistical model and inference

The model

This is a model borrowed from econometrics (1) and recently used in the context of infectious diseases which occur simultaneously or are inherently influenced by correlated binary outcomes. We adapted the methodology in the framework of these two binary highly correlated outcomes which can be associated with a similar (or not) set of covariates and the presence of one is highly correlated with the other. A lay description of the approach is presented in the main text. Here, we give details on the mathematical equations and the statistical inference.

Let us denote by X and Y the two binary random variables indicating the presence/absence of an individual to DENV and CHIKV, respectively. This pair of responses define a binary bivariate outcome denoted by (X, Y).

Let us also denote

$R_i = 1 \times \boldsymbol{K}_X$ vector of variables associated with X

$\boldsymbol{\beta} = \boldsymbol{K}_X \times 1$ vector of unknown parameters which quantify the effect of variables associated with X

$Q_i = 1 \times \boldsymbol{K}_Y$ vector of variables associated with Y

$\boldsymbol{\gamma} = \boldsymbol{K}_Y \times 1$ vector of unknown parameters which quantify the effect of variables associated with Y,

$i = \overline{1,319}\,,\ j = \overline{1,151},\ k = \overline{1{,}49}$

NB: We deliberately keep the generality of the model specification although in our scenario $\boldsymbol{K}_X = \boldsymbol{K}_Y$

For peer review only - http://bmjopen.bmj.com/site/about/guidelines.xhtml

Then, the two simultaneous logistic equations which depict the nonlinear association with age and the rest of the other variables are written in terms of a system of conditional probabilities on the potential explanatory variables and their random effects:

$$\begin{cases} log\left(\frac{P(X=1|Y)}{P(X=0|Y)}\right)_{ijk} = \beta_{age} \times \log(\text{age}_{ijk}) + \boldsymbol{\beta} \times R_{ijk} + \boldsymbol{\alpha} \times Y_{ijk} + w_{ijk}^{age} + w_k^{ward} + v_{0k}^1 + u_{0jk}^1 \\ log\left(\frac{P(Y=1|X)}{P(Y=0|X)}\right)_{ijk} = \gamma_{age} \times \log(\text{age}_{ijk}) + \boldsymbol{\gamma} \times Q_{ijk} + \boldsymbol{\theta} \times X_{ijk} + w_{ijk}^{age} + w_k^{ward} + v_{0k}^2 + u_{0jk}^2 \end{cases}$$

(1)

The triple index (ijk) – denotes individual i in the family j and the ward k

w_{ijk}^{age} denote the age-group constants associated weights (see the subsequent section on Population adjusting weights)

w_k^{ward} denote the ward constants associated weights (see the subsequent section on Population adjusting weights)

$v_{0k}^{1,2}$ denote the random effect associated with the ward

$u_{0jk}^{1,2}$ denote the random effect associated with the family

The latter two sets of random effects are assumed to be drawn from a two normal bivariate normal distributions (MVN) which model the shared and unshared similarities and account for unmeasured aspects such as mosquitos' burden around the households or weather patterns for instance.

$$\begin{pmatrix} v_{0k}^1 \\ v_{0k}^2 \end{pmatrix} \sim MVN\left(\begin{pmatrix} 0 \\ 0 \end{pmatrix}, \sum \begin{pmatrix} (v_0^1)^2 & \rho_v v_0^1 v_0^2 \\ \rho_v v_0^1 v_0^2 & (v_0^2)^2 \end{pmatrix}\right)$$

For peer review only - http://bmjopen.bmj.com/site/about/guidelines.xhtml

$$\begin{pmatrix} u_{0jk}^{1} \\ u_{0jk}^{2} \end{pmatrix} \sim MVN\left(\begin{pmatrix} 0 \\ 0 \end{pmatrix}, \sum \begin{pmatrix} (u_0^1)^2 & \rho_u u_0^1 u_0^2 \\ \rho_u u_0^1 u_0^2 & (u_0^2)^2 \end{pmatrix} \right)$$

On one hand side, from the first simultaneous equation specification (1):

$$\boldsymbol{\alpha} = log\left[\frac{P(X=1|Y=1)}{P(X=0|Y=1)}\right] - log\left[\frac{P(X=1|Y=0)}{P(X=0|Y=0)}\right] = log\frac{P(X=1,Y=1)/P(Y=1)}{P(X=0,Y=1)/P(Y=1)} - log\frac{P(X=1,Y=0)/P(Y=0)}{P(X=0,Y=0)/P(Y=0)} =$$

$$log\left[\frac{P(X=1,Y=1)\times P(X=0,Y=0)}{P(X=0,Y=1)\times P(X=1,Y=0)}\right] \quad (2)$$

From the second simultaneous equation specification:

$$\boldsymbol{\theta} = log\left[\frac{P(Y=1|X=1)}{P(Y=0|X=1)}\right] - log\left[\frac{P(Y=1|X=0)}{P(Y=0|X=0)}\right] = log\frac{P(X=1,Y=1)/P(X=1)}{P(X=0,Y=1)/P(X=1)} - log\frac{P(X=1,Y=0)/P(X=0)}{P(X=0,Y=0)/P(X=0)} =$$

$$log\left[\frac{P(X=1,Y=1)\times P(X=0,Y=0)}{P(X=0,Y=1)\times P(X=1,Y=0)}\right] \quad (3)$$

Hence, using the key assumption that $\boldsymbol{\alpha} = \boldsymbol{\theta}$ and keeping the fixed effects only for the sake of simplicity, the system can be written as

$$\begin{cases} log\left(\dfrac{P(X=1|Y)}{P(X=0|Y)}\right)_i = \boldsymbol{\beta} \times R_i + \boldsymbol{\alpha} \times Y_i \\ log\left(\dfrac{P(Y=1|X)}{P(Y=0|X)}\right)_i = \boldsymbol{\gamma} \times Q_i + \boldsymbol{\alpha} \times X_i \end{cases}$$

This implies that, if the occurrence of dengue makes more likely (or less likely) the occurrence of chikungunya, the converse is also necessarily true and $\boldsymbol{\alpha}$ is deemed as a nuisance parameter

This crucial assumption ($\boldsymbol{\alpha} = \theta$) and elementary rules of conditioned probabilities leads to the following four statements derived from

For peer review only - http://bmjopen.bmj.com/site/about/guidelines.xhtml

$$\begin{cases} \dfrac{P(X=1|Y=0)}{P(X=0|Y=0)} = \dfrac{P(X=1,Y=0)}{P(X=0,Y=0)} = exp(\boldsymbol{\beta} \times \boldsymbol{R}) & (4) \\ \dfrac{P(X=1|Y=1)}{P(X=0|Y=1)} = \dfrac{P(X=1,Y=1)}{P(X=0,Y=1)} = exp(\boldsymbol{\beta} \times \boldsymbol{R} + \alpha) & (5) \end{cases}$$

$$\begin{cases} \dfrac{P(Y=1|X=0)}{P(Y=0|X=0)} = \dfrac{P(Y=1,X=0)}{P(Y=0,X=0)} = exp(\boldsymbol{\gamma} \times \boldsymbol{Q}) & (6) \\ \dfrac{P(Y=1|X=1)}{P(Y=0|X=1)} = \dfrac{P(Y=1,X=1)}{P(Y=0,X=1)} = exp(\boldsymbol{\gamma} \times \boldsymbol{Q} + \alpha) & (7) \end{cases}$$

Hence, combining either (4) + (7) (or (5) + (6)) by multiplication

$$\frac{P(X=1,Y=0)}{P(X=0,Y=0)} \times \frac{P(Y=1,X=1)}{P(Y=0,X=1)} =$$

$$\frac{P(Y=1,X=1)}{P(Y=0,X=0)} = exp(\boldsymbol{\beta} \times \boldsymbol{R}) \times exp(\boldsymbol{\gamma} \times \boldsymbol{Q} + \alpha) = exp(\boldsymbol{\beta} \times \boldsymbol{R} + \boldsymbol{\gamma} \times \boldsymbol{Q} + \alpha)$$

But all four probabilities situations ((1, 0), (0, 1), (1, 1), (0,0)) sum to 1:

$$P(X=1,Y=0) + P(X=0,Y=1) + P(Y=0,X=0) + P(Y=1,X=1) = 1$$

$$P(X=0,Y=0) + P(X=0,Y=0) \times exp(\boldsymbol{\gamma} \times Q) + P(Y=0,X=0) \times exp(\boldsymbol{\beta} \times \boldsymbol{R})$$
$$+ P(Y=0,X=0) \times exp(\boldsymbol{\beta} \times \boldsymbol{R} + \boldsymbol{\gamma} \times \boldsymbol{Q} + \alpha) = 1$$

So

$$P(X=0,Y=0) \times \left(1 + exp(\boldsymbol{\gamma} \times \boldsymbol{Q}) + exp(\boldsymbol{\beta} \times \boldsymbol{R}) + exp(\boldsymbol{\beta} \times \boldsymbol{R} + \boldsymbol{\gamma} \times \boldsymbol{Q} + \alpha)\right) = 1$$

Therefore, the predicted joint probabilities can be derived as it follows:

$$P(X=0,Y=0) = \frac{1}{1 + exp(\boldsymbol{\gamma} \times \boldsymbol{Q}) + exp(\boldsymbol{\beta} \times \boldsymbol{R}) + exp(\boldsymbol{\beta} \times \boldsymbol{R} + \boldsymbol{\gamma} \times \boldsymbol{Q} + \alpha)}$$

$$P(X=0,Y=1) = \frac{exp(\boldsymbol{\gamma} \times \boldsymbol{Q})}{1 + exp(\boldsymbol{\gamma} \times \boldsymbol{Q}) + exp(\boldsymbol{\beta} \times \boldsymbol{R}) + exp(\boldsymbol{\beta} \times \boldsymbol{R} + \boldsymbol{\gamma} \times \boldsymbol{Q} + \alpha)}$$

For peer review only - http://bmjopen.bmj.com/site/about/guidelines.xhtml

$$P(X=1,Y=0)=\frac{exp(\boldsymbol{\beta}\times\boldsymbol{R})}{1+exp(\boldsymbol{\gamma}\times\boldsymbol{Q})+exp(\boldsymbol{\beta}\times\boldsymbol{R})+exp(\boldsymbol{\beta}\times\boldsymbol{R}+\boldsymbol{\gamma}\times\boldsymbol{Q}+\alpha)}$$

$$P(X=1,Y=1)=\frac{exp(\boldsymbol{\beta}\times\boldsymbol{R}+\boldsymbol{\gamma}\times\boldsymbol{Q}+\alpha)}{1+exp(\boldsymbol{\gamma}\times\boldsymbol{Q})+exp(\boldsymbol{\beta}\times\boldsymbol{R})+exp(\boldsymbol{\beta}\times\boldsymbol{R}+\boldsymbol{\gamma}\times\boldsymbol{Q}+\alpha)}$$

Denoting by

$$I_{ij}=\{k|X_k=i,Y_k=j\}\ where\ \ i,j=0,1$$

If the vector of the unknown parameters are denoted by $\phi=(\alpha,\boldsymbol{\beta},\boldsymbol{\gamma})$**,** the model likelihood can be formally written as

$$L(\phi|Y_k,X_k;\boldsymbol{R},\boldsymbol{Q})=\prod_{i=0,1}\prod_{j=0,1}\prod_{k\in I_{ij}}P(X_k=i,Y_k=j)$$

Bayesian inference consist of summarizing the posterior distributions of the model parameters and requires the joint probability distribution over all random quantities, parameters or observables. The marginal and conditional probability distributions of the vector of unknown parameters are denoted by $Pr(\cdot)$ and $Pr(\cdot\mid\cdot)$. The ϕ posterior distribution is proportional to the likelihood multiplied by their prior distributions – all assumed non informative as stated in the text. That is

$$Pr(\phi|Y_k,X_k;\boldsymbol{R},\boldsymbol{Q})\propto L(\phi|Y_k,X_k;\boldsymbol{R},\boldsymbol{Q})\times Pr(\phi)$$

Statistical Inference

The simultaneous equations systems (1) define a probability model which has been implemented in OpenBUGS - which makes this approach very appealing even for larger datasets (OpenBUGS program for the simplest model appended). A Bayesian framework for statistical inference using Markov chain Monte Carlo (MCMC) estimation methods naturally accommodates missing

For peer review only

For peer review only - http://bmjopen.bmj.com/site/about/guidelines.xhtml

responses (5%) in the data and allows straightforward assessment of uncertainties in nonlinear models under missing at random assumption. Sensitivities analyses to the missing data (by assuming all positives and all negatives) have been conducted but the results were not altered.

The posterior conditional odds ratios (OR) distributions are presented in Table 2. However, for an easily digestible reading, the summary statistics are presented in terms of means and standard deviations of the predicted joint probabilities by age and stratified by different socio-economic factors that we accounted for.

The MCMC estimation followed 1 million iterations and the corresponding chains (log scale) are displayed in Supplementary Figure 8 – including that of the nuisance parameter. Supplementary Figure 9 display the correlation between the parameters which are all lower than 0.5 in absolute values with the exception of that between the constant and age corresponding to the dengue equations with appear to be highly negatively correlated (-0.87).

Non-informative priors have been used for parameter estimation.

Population adjusting weights used in modelling

We used the 2010 Census data (the latest) adjusted for an average annual growth of in the Ecuador population. All statistical analyses should ultimately serve population predictions. The post-stratification weights, namely calibration, is a technique which corrects the estimation for population misrepresentation which often occurs in ad-hoc surveys or when researchers use already recruited participants. One set of weights are aimed at reducing the effect of Rosa Zarate where most of the data were collected and at inflating the influence of the misrepresented groups (La Union and 10-19 years) on the model likelihood. These weights are associated with each individual and are additive for the log scale model formulation of the model (see equations

For peer review only - http://bmjopen.bmj.com/site/about/guidelines.xhtml

above). The other set of weights are aimed at reducing the effect of those in young group and inflate the effects of older people less represented in the sample. The weights are displayed Supplementary Tables 3 and 4. Each observation is associated with a weight according to individual's age group and one by the household location and their additive effect is accounted for in the predictors (log scale so their effect on the actual odds ratios or odds would be multiplicative).

Goodness of fit

The model is developed using a fully parametric paradigm and inherits complexities including random effects for binary outcomes which are not associated with any classical or formal goodness of fit measures.

However, in reference to the basic independent binary outcomes, the only goodness-of-fit measure following a potential *individual based model* fit to these data is that of Hosmer-Lemeshow statistic. That invokes partition of the data *into groups* (https://thestatsgeek.com/2014/02/16/the-hosmer-lemeshow-goodness-of-fit-test-for-logistic-regression/). That is because the individual level raw observations are either 0 or 1 whilst the individual level predicted values can be anywhere between 0 and 1 and hence a one-to-one observed-predicted plot often used for continuous data loses its meaning.

Hence, our approach alludes to that of basic logistic regression and follows similar lines to those developed in Chis Ster, I. Journal of Applied Statistics, 2012. **39**(11): p. 2523-2542, (*Inference for serological surveys investigating past exposures to infections resulting in long-lasting immunity – an approach using finite mixture models with concomitant information)* within a

For peer review only - http://bmjopen.bmj.com/site/about/guidelines.xhtml

related and yet equally complex modelling framework.

We split the data into groups, by 5 years age-groups and geographical sub-regions, and calculated the average predicted probabilities and their 95%CrIs for the outcomes (((D+, C-), (D-, C+), (D+, C+) and also for all dengue and chikungunya) within each group. Then we calculated the expected number of positives corresponding each group and each outcome component by multiplying the corresponding average predicted probabilities with the total number of people within that group. We plotted the observed numbers vs. the predicted numbers in each group.

Given the full parametric, parsimonious nature of this model, and the average sized data – we deemed our model as a good fit to these data.

For peer review only

OpenBUGS code:

```
model{
    #model's likelihood
       for (i in 1:319){
              log_age[i]<-log(age_final_years[i]);
              log_dist[i]<-log(1+min_dist[i]/250);
              weights_final[i]<-weights[i]+weight_age[i];

              dengue[i]~dbern(p[i,1]);
              chick[i] ~dbern(p[i,2]);
              eta[i,1]<-beta1[1]+beta1[2]*log_age[i]+alpha*chick[i]+beta1[3]*gender[i]+
                               beta1[4]*ses_bin[i]+
                               weights_final[i]+
                               theta[family[i],1]+
                               ward_level[ward_index[i],1];

             eta[i,2]<- beta2[1]+beta2[2]*log_age[i]+alpha*dengue[i]+
                               beta2[3]*gender[i]+
                               beta2[4]*urbanrural[i]+beta2[5]*log_dist[i]+
                               weights_final[i]+
                               theta[family[i],2]+
                               ward_level[ward_index[i],2];
               logit(p[i,1])<-eta[i,1];
              logit(p[i,2])<-eta[i,2];
       }
       for (j in 1:151){
              theta[j,1:2] ~ dmnorm(mu.fam[],R_fam[,]);
        }
    for(j in 1:49){
         ward_level[j,1:2] ~ dmnorm(mu.ward[],R_ward[,]);
    }
    for (k in 1:2){

                    mu.fam[k] <-0.0;
                  mu.ward[k] <-0.0;
       }

    R_fam[1:2,1:2] ~dwish(Omega_fam[,],2);
    R_ward[1:2,1:2]~dwish(Omega_ward[,],2);
    Omega_fam[1,1]<-1; Omega_fam[1,2]<-0; Omega_fam[2,1]<-0; Omega_fam[2,2]<-1;
 Omega_ward[1,1]<-1; Omega_ward[1,2]<-0; Omega_ward[2,1]<-0; Omega_ward[2,2]<-1;
```

```
    Sigma.fam[1:2, 1:2] <-inverse(R_fam[,]);
    Sigma.ward[1:2, 1:2]<-inverse(R_ward[,]);
    for (j in 1:2){
        sigma.fam[j]<-sqrt(Sigma.fam[j,j]);
        sigma.ward[j]<-sqrt(Sigma.ward[j,j]);
        for(k in 1:j){
                rho_fam[j,k]<-0;
                 rho_ward[j,k]<-0;
         }
        for(k in (j+1):2){
               rho_fam[j,k]<-Sigma.fam[j,k]/(sigma.fam[j]*sigma.fam[k]);
               rho_ward[j,k]<-Sigma.ward[j,k]/(sigma.ward[j]*sigma.ward[k]);
        }
    }
    alpha ~ dnorm( 0.0, 0.00001);
    for (j in 1:4){
        beta1[j] ~ dnorm( 0.0, 0.00001);
        beta2[j] ~ dnorm( 0.0, 0.00001);
        exp_beta1[j]<-exp(beta1[j]);
         exp_beta2[j]<-exp(beta2[j]);
         eff1[j] <-step(beta1[j]);
         eff2[j] <-step(beta2[j]);
    }
           exp_alpha<-exp(alpha);
           beta2[5] ~ dnorm( 0.0, 0.00001);
           exp_beta2[5]<-exp(beta2[5]);
}
```

References

1. Schmidt P, Strauss RP. Estimation of Models with Jointly Dependent Qualitative Variables: A Simultaneous Logit Approach. Econometrica. 1975;43(4):745-55.
2. Ghebremichael M. Joint modeling of correlated binary outcomes: HIV-1 and HSV-2 co-infection. Journal of Applied Statistics. 2015 2015/10/03;42(10):2180-91.
3. Goldstein H. Multilevel Statistical Models. 4th Edition ed: JohnWiley and Sons, Ltd, Chichester; 2011.

For peer review only - http://bmjopen.bmj.com/site/about/guidelines.xhtml

STROBE 2007 (v4) checklist of items to be included in reports of observational studies in epidemiology*
Checklist for cohort, case-control, and cross-sectional studies (combined)

Section/Topic	Item #	Recommendation	Reported on page #
**Title and abstract**	1	(*a*) Indicate the study's design with a commonly used term in the title or the abstract	#1 Title:Age-dependent prevalence
		(*b*) Provide in the abstract an informative and balanced summary of what was done and what was found	#3 Abstract
**Introduction**			
Background/rationale	2	Explain the scientific background and rationale for the investigation being reported	#6 Introduction
Objectives	3	State specific objectives, including any pre-specified hypotheses	#7 Introduction
**Methods**			
Study design	4	Present key elements of study design early in the paper	#8 Materials and methods
Setting	5	Describe the setting, locations, and relevant dates, including periods of recruitment, exposure, follow-up, and data collection	#8 Materials and methods
Participants	6	*Cross-sectional study*—Give the eligibility criteria, and the sources and methods of selection of participants	#8 Materials and methods
		(*b*) *Cohort study*—For matched studies, give matching criteria and number of exposed and unexposed<br>*Case-control study*—For matched studies, give matching criteria and the number of controls per case	
Variables	7	Clearly define all outcomes, exposures, predictors, potential confounders, and effect modifiers. Give diagnostic criteria, if applicable	#8-9 Statistical Methods
Data sources/ measurement	8*	For each variable of interest, give sources of data and details of methods of assessment (measurement). Describe comparability of assessment methods if there is more than one group	#8 Materials and methods
Bias	9	Describe any efforts to address potential sources of bias	#6, #16
Study size	10	Explain how the study size was arrived at	#8 – random sample from an existing cohort
Quantitative variables	11	Explain how quantitative variables were handled in the analyses. If applicable, describe which groupings were chosen and why	#8 Materials and methods
Statistical methods	12	(*a*) Describe all statistical methods, including those used to control for confounding	#8-9 Statistical Methods

For peer review only

		(*b*) Describe any methods used to examine subgroups and interactions	#8-9 Statistical Methods
		(*c*) Explain how missing data were addressed	NA
		(*d*) *Cohort study*—If applicable, explain how loss to follow-up was addressed<br>*Case-control study*—If applicable, explain how matching of cases and controls was addressed<br>*Cross-sectional study*—If applicable, describe analytical methods taking account of sampling strategy	#8-9 Statistical Methods<br>Random sample from an existing cohort.<br>Weighting adjustments to represent the population
		(*e*) Describe any sensitivity analyses	NA
**Results**			
Participants	13*	(a) Report numbers of individuals at each stage of study—eg numbers potentially eligible, examined for eligibility, confirmed eligible, included in the study, completing follow-up, and analysed	319
		(b) Give reasons for non-participation at each stage	NA
		(c) Consider use of a flow diagram	NA
Descriptive data	14*	(a) Give characteristics of study participants (eg demographic, clinical, social) and information on exposures and potential confounders	#18 Table 1
		(b) Indicate number of participants with missing data for each variable of interest	NA
		(c) *Cohort study*—Summarise follow-up time (eg, average and total amount)	NA
Outcome data	15*	*Cohort study*—Report numbers of outcome events or summary measures over time	
		Case-control study—Report numbers in each exposure category, or summary measures of exposure	
		Cross-sectional study—Report numbers of outcome events or summary measures	#18 -19 Table 1, Table 2
Main results	16	(*a*) Give unadjusted estimates and, if applicable, confounder-adjusted estimates and their precision (eg, 95% confidence interval). Make clear which confounders were adjusted for and why they were included	#10 Results
		(*b*) Report category boundaries when continuous variables were categorized	#18 -19 Table 1
		(*c*) If relevant, consider translating estimates of relative risk into absolute risk for a meaningful time period	#10 Results – Supplementary Tables
Other analyses	17	Report other analyses done—eg analyses of subgroups and interactions, and sensitivity analyses	#10 Results<br>#13-16 Discussion
**Discussion**			
Key results	18	Summarise key results with reference to study objectives	#13-16 Discussion
Limitations	19	Discuss limitations of the study, taking into account sources of potential bias or imprecision. Discuss both direction and magnitude of any potential bias	#4 Strength and limitations

For peer review only

			#13-16 Discussion
Interpretation	20	Give a cautious overall interpretation of results considering objectives, limitations, multiplicity of analyses, results from similar studies, and other relevant evidence	#13-16 Discussion
Generalisability	21	Discuss the generalisability (external validity) of the study results	#13-16 Discussion
**Other information**			
Funding	22	Give the source of funding and the role of the funders for the present study and, if applicable, for the original study on which the present article is based	#24 Wellcome Trust (088862/Z/09/Z)

*Give information separately for cases and controls in case-control studies and, if applicable, for exposed and unexposed groups in cohort and cross-sectional studies.

Note: An Explanation and Elaboration article discusses each checklist item and gives methodological background and published examples of transparent reporting. The STROBE checklist is best used in conjunction with this article (freely available on the Web sites of PLoS Medicine at http://www.plosmedicine.org/, Annals of Internal Medicine at http://www.annals.org/, and Epidemiology at http://www.epidem.com/). Information on the STROBE Initiative is available at www.strobe-statement.org.

For peer review only
